# Clinical characteristics and factors relating to poor outcome in patients with aneurysmal subarachnoid hemorrhage in Vietnam: A multicenter prospective cohort study

**DOI:** 10.1371/journal.pone.0256150

**Published:** 2021-08-13

**Authors:** Chinh Quoc Luong, Hung Manh Ngo, Hai Bui Hoang, Dung Thi Pham, Tuan Anh Nguyen, Tuan Anh Tran, Duong Ngoc Nguyen, Son Ngoc Do, My Ha Nguyen, Hung Dinh Vu, Hien Thi Thu Vuong, Ton Duy Mai, Anh Quang Nguyen, Kien Hoang Le, Phuong Viet Dao, Thong Huu Tran, Luu Dang Vu, Linh Quoc Nguyen, Trang Quynh Pham, He Van Dong, Hao The Nguyen, Chi Van Nguyen, Anh Dat Nguyen

**Affiliations:** 1 Center for Emergency Medicine, Bach Mai Hospital, Hanoi, Vietnam; 2 Department of Emergency and Critical Care Medicine, Hanoi Medical University, Hanoi, Vietnam; 3 Faculty of Medicine, University of Medicine and Pharmacy, Vietnam National University, Hanoi, Vietnam; 4 Department of Neurosurgery II, Neurosurgery Center, Vietnam-Germany Friendship Hospital, Hanoi, Vietnam; 5 Emergency and Critical Care Department, Hanoi Medical University Hospital, Hanoi Medical University, Hanoi, Vietnam; 6 Department of Nutrition and Food Safety, Faculty of Public Health, Thai Binh University of Medicine and Pharmacy, Thai Binh, Vietnam; 7 Radiology Centre, Bach Mai Hospital, Hanoi, Vietnam; 8 Department of Radiology, Hanoi Medical University, Hanoi, Vietnam; 9 Department of Health Organization and Management, Faculty of Public Health, Thai Binh University of Medicine and Pharmacy, Thai Binh, Vietnam; 10 Emergency Department, Vietnam–Czechoslovakia Friendship Hospital, Hai Phong, Vietnam; 11 Stroke Center, Bach Mai Hospital, Hanoi, Vietnam; 12 Department of Neurosurgery, Bach Mai Hospital, Hanoi, Vietnam; 13 Department of Neurosurgery I, Neurosurgery Center, Vietnam-Germany Friendship Hospital, Hanoi, Vietnam; Hospital Dr. Rafael A. Calderón Guardia, CCSS, COSTA RICA

## Abstract

**Background:**

The prevalence of risk factors for poor outcomes from aneurysmal subarachnoid hemorrhage (SAH) varies widely and has not been fully elucidated to date in Vietnam. Understanding the risk and prognosis of aneurysmal SAH is important to reduce poor outcomes in Vietnam. The aim of this study, therefore, was to investigate the rate of poor outcome at 90 days of ictus and associated factors from aneurysmal SAH in the country.

**Methods:**

We performed a multicenter prospective cohort study of patients (≥18 years) presenting with aneurysmal SAH to three central hospitals in Hanoi, Vietnam, from August 2019 to August 2020. We collected data on the characteristics, management, and outcomes of patients with aneurysmal SAH and compared these data between good (defined as modified Rankin Scale (mRS) of 0 to 3) and poor (mRS, 4–6) outcomes at 90 days of ictus. We assessed factors associated with poor outcomes using logistic regression analysis.

**Results:**

Of 168 patients with aneurysmal SAH, 77/168 (45.8%) were men, and the median age was 57 years (IQR: 48–67). Up to 57/168 (33.9%) of these patients had poor outcomes at 90 days of ictus. Most patients underwent sudden-onset and severe headache (87.5%; 147/168) and were transferred from local to participating central hospitals (80.4%, 135/168), over half (57.1%, 92/161) of whom arrived in central hospitals after 24 hours of ictus, and the initial median World Federation of Neurological Surgeons (WFNS) grading score was 2 (IQR: 1–4). Nearly half of the patients (47.0%; 79/168) were treated with endovascular coiling, 37.5% (63/168) were treated with surgical clipping, the remaining patients (15.5%; 26/168) did not receive aneurysm repair, and late rebleeding and delayed cerebral ischemia (DCI) occurred in 6.1% (10/164) and 10.4% (17/163) of patients, respectively. An initial WFNS grade of IV (odds ratio, OR: 15.285; 95% confidence interval, CI: 3.096–75.466) and a grade of V (OR: 162.965; 95% CI: 9.975–2662.318) were independently associated with poor outcomes. Additionally, both endovascular coiling (OR: 0.033; 95% CI: 0.005–0.235) and surgical clipping (OR: 0.046; 95% CI: 0.006–0.370) were inversely and independently associated with poor outcome. Late rebleeding (OR: 97.624; 95% CI: 5.653–1686.010) and DCI (OR: 15.209; 95% CI: 2.321–99.673) were also independently associated with poor outcome.

**Conclusions:**

Improvements are needed in the management of aneurysmal SAH in Vietnam, such as increasing the number of aneurysm repairs, performing earlier aneurysm treatment by surgical clipping or endovascular coiling, and improving both aneurysm repairs and neurocritical care.

## Introduction

Subarachnoid hemorrhage (SAH) is often a devastating event with high mortality, morbidity, and burden of healthcare [1, 2]. The mortality rate is approximately 50% in population-based studies with a trend towards gradual improvement [3–5]. This mortality rate includes 10–18% of all patients with aneurysmal SAH who die at home or during transportation to the hospital [6, 7]. Among patients who reach the hospital alive, subsequent early death is caused by the common complications of aneurysmal SAH related to initial bleeding, rebleeding, delayed cerebral ischemia (DCI), hydrocephalus, increased intracranial pressure (ICP), seizures, and cardiac complications [8–10]. Additionally, patients with aneurysmal SAH who are discharged alive from the hospital have an increased long-term mortality rate compared with the general population [11–15]. Patients who are discharged alive from the hospital also have high rates of memory and neurocognitive impairment [16, 17]. At three months after aneurysmal clipping, global impairment was present in approximately 20% of all patients who were discharged alive from the hospital and in 16% of those with excellent preoperative conditions [18]. The location of the aneurysm responsible for aneurysmal SAH does not appear to influence the cognitive outcome compared to the occurrence of DCI and other complications [15, 19, 20].

Advances in diagnostic and treatment strategies for aneurysmal SAH, through the introduction of computed tomography (CT) angiography with early detection of aneurysms, the use of nimodipine, specialist care for patients, and endovascular coiling of ruptured aneurysms, have substantially improved the outcomes of hospitalized patients [21–24]. Despite these improvements, aneurysmal SAH continues to extract a high economic and social cost [25]. It remains a disease of relatively young people and causes a loss of productive life-years similar to that of ischemic stroke.

Economic and political reforms have spurred rapid economic growth in Vietnam [26]. However, medical providers still have difficulty caring for patients with aneurysmal SAH in local settings because of low resources and a lack of advanced diagnostic and treatment strategies [27, 28]. Furthermore, although national health insurance was established in 1992 to improve access to health care and mitigate the negative impact of user fees introduced in 1989, advances in diagnosis and treatment are incompletely covered by health insurance. At the same time, the medical staff may not be sufficiently well trained or experienced to be able to recognize aneurysmal SAH and other severe conditions in their patients and provide the required care [27–29]. Additionally, within the healthcare system in Vietnam, central hospitals are responsible for receiving patients who have difficulty being treated in local hospital settings [30]. Therefore, the initiation of aneurysm treatment and appropriate supportive care in patients with aneurysmal SAH is often delayed [27, 28].

Understanding the country-specific causes, risks, and prognosis of aneurysmal SAH is important to reduce poor outcomes and mortality in Vietnam. The aim of this study, therefore, was to investigate the rate of poor outcomes and associated factors from aneurysmal SAH in the country.

## Methods

### Study design and setting

We performed a multicenter prospective observational cohort study that included all patients with aneurysmal SAH consecutively admitted to the emergency departments (EDs) of the three national tertiary hospitals (Vietnam-Germany Friendship, Bach Mai, and Hanoi Medical University Hospital) in Hanoi, Vietnam, between August 2019 and August 2020. These hospitals were designated central hospitals (level I) in northern Vietnam by the Ministry of Health (MOH) of Vietnam [29, 30], of which the first is a surgical hospital with 1,500 beds, the second is a larger general hospital with 3,200 beds, and the last is a smaller general hospital with 580 beds. In the healthcare system of Vietnam, central hospitals are responsible for training hospital staff and treating patients who are unable to be adequately treated in local hospital settings, including provincial and district hospitals (levels II and III, according to the MOH of Vietnam).

### Participants and treatments

This study included all patients (aged 18 years or older) presenting with aneurysmal SAH to the EDs of the three central hospitals within 4 days of ictus. We defined a case of aneurysmal SAH as a person who had the presence of blood on head CT scan (or in case CT scan was negative on the presence of xanthochromia in cerebral spinal fluid) in combination with an aneurysm confirmed on CT or digital subtraction angiography (DSA) [16]. We excluded patients for whom the Glasgow coma scale (GCS) on admission was unable to score (e.g., the patients intubated before arrival in the central hospital) or neurological functional outcome was unknown at 90 days of ictus.

All patients were managed following the American Heart Association (AHA)/American Stroke Association (ASA) guidelines for the management of aneurysmal SAH [16]. Aneurysm repair with surgical clipping or endovascular coiling was performed as early as possible and immediately if rebleeding did occur. The decision to treat cerebral aneurysms was at the discretion of the physician in charge of the patients and the availability of neurosurgical clipping or endovascular coiling, which depended on the participating hospital or the financial situation (either insurance or patient self-pay).

### Data collection

The data for each study patient were recorded from the same unified samples (case record form). A case record form was adopted across the study sites to collect common variables. Data were entered into the study database by EpiData Entry software, which was used for simple or programmed data entry and data documentation that could prevent data entry errors or mistakes. Patient identifiers were not entered in the database to protect patients’ confidentiality.

### Variables

We included variables based on unruptured intracranial aneurysm (UIA) and SAH work group (WG) recommendations [31], such as information on:

- Assessments and examinations, including histories (e.g., stroke, UIA, etc.); clinical presentation (e.g., GCS and focal neurological signs); SAH grading scales such as the WFNS grading scale ranging from grade I (GCS score of 15) to V (GCS scores of 3 to 6) of which focal deficits making up 1 additional grade for patients with a GCS score of 14 or 13 [32] and the Hunt and Hess scale also consists of five grades ranging from minimally symptomatic to coma [33].
- Laboratory, such as coagulation tests (e.g., platelets, prothrombin time and international normalized ratio) and other tests.
- Neuroimaging, such as admission CT scan (e.g., presence of SAH, IVH or ICH, and Fisher scale) and follow-up CT scan during hospitalization (e.g., presence of SAH, IVH or ICH) or at 30 and 90 days of ictus (e.g., presence of chronic hydrocephalus).
- Management, including surgical and endovascular interventions (i.e., surgical clipping or endovascular coiling); rescue therapies (e.g., surgical hematoma evacuation, decompressive craniectomy, EVD placement, VP shunt); and intensive care unit (ICU) therapies (e.g., mechanical ventilation).
- Neurological complications, including early and late rebleeding, which included bleeding into the subarachnoid space, intracerebral, intraventricular, or subdural compartments and catheter-induced hemorrhages; DCI; acute hydrocephalus, which was defined as marked symmetrical dilatation of the ventricles with an Evans ratio of at least 0.3 on admission [34].
- Hospital course and outcomes, including length of hospitalization, discharge status (e.g., hospital discharge, transfer to another hospital, “discharged to die” decision in which almost all patients were in grave condition or dying and classified with a modified Rankin Scale (mRS) score of 5 (severe disability) at the time of discharge [35], and death in hospital); functional outcomes at 30 and 90 days of ictus, such as mRS scores ranging from 0 (no disability) to 6 (death) [36]; and death at 30 and 90 days of ictus.

We also collected data on behavioral history (e.g., cigarette smoking, alcohol drinking), demographics (i.e., sex, age), and social status (e.g., health insurance, occupations, highest education levels, annual income).

### Outcomes

The primary outcome of the study was poor neurological function (poor outcome) at 90 days of ictus, which was defined as mRS scores of 4 (moderately severe disability) to 6 (death) [35, 37]. We also examined the following secondary outcomes: poor outcome at 30 days of ictus, mortality rate at 30 and 90 days of ictus, and incidence of complications.

### Data analyses

We used IBM^®^ SPSS^®^ Statistics 22.0 (IBM Corp., Armonk, United States of America) for data analysis. We report data as numbers and percentages for categorical variables and medians and interquartile ranges (IQRs) or means and standard deviations (SDs) for continuous variables. Comparisons were made among methods of aneurysm repair, between poor and good outcomes, and between alive and dead patients for each variable using the χ2 test or Fisher exact test for categorical variables and the Mann–Whitney U test, Kruskal–Wallis test, one-way analysis of variance for continuous variables. We assessed factors associated with poor outcome or death using logistic regression analysis and included independent variables related to the patient (age, sex, risk factors for aneurysmal SAH, comorbidities), the SAH event (onset symptoms, presentation on admission, and SAH severity), therapy provided (e.g., no aneurysm repair, endovascular coiling, and surgical clipping) and complications (e.g., DCI) if the P-value was < 0.05 in the bivariate analysis between poor and good outcomes and between alive and dead patients. We used a stepwise method to select variables and then used the backward method with these variables. We present odds ratios (ORs) and 95% confidence intervals (CIs). For all analyses, significance levels were two-tailed, and we considered P < 0.05 to be statistically significant.

### Ethical issues

The Hanoi Medical University (Approval number: 3335/QĐ-ĐHYHN), Vietnam-Germany Friendship Hospital (Approval number: 818/QĐ-VĐ; Research code: KH04.2020), and Bach Mai Hospital (Approval number: 3288/QĐ-BM; Research code: BM_2020_1247) Scientific and Ethics Committees approved this study. The study was conducted according to the principles of the Declaration of Helsinki. The Vietnam-Germany Friendship Hospital Scientific and Ethics Committees waived written informed consent for this noninterventional study, and public notification of the study was made by public posting. The authors who performed the data analysis kept the data sets in password-protected systems, and we present anonymized data.

## Results

### General characteristics, treatment, and outcomes

**Fig 1:**
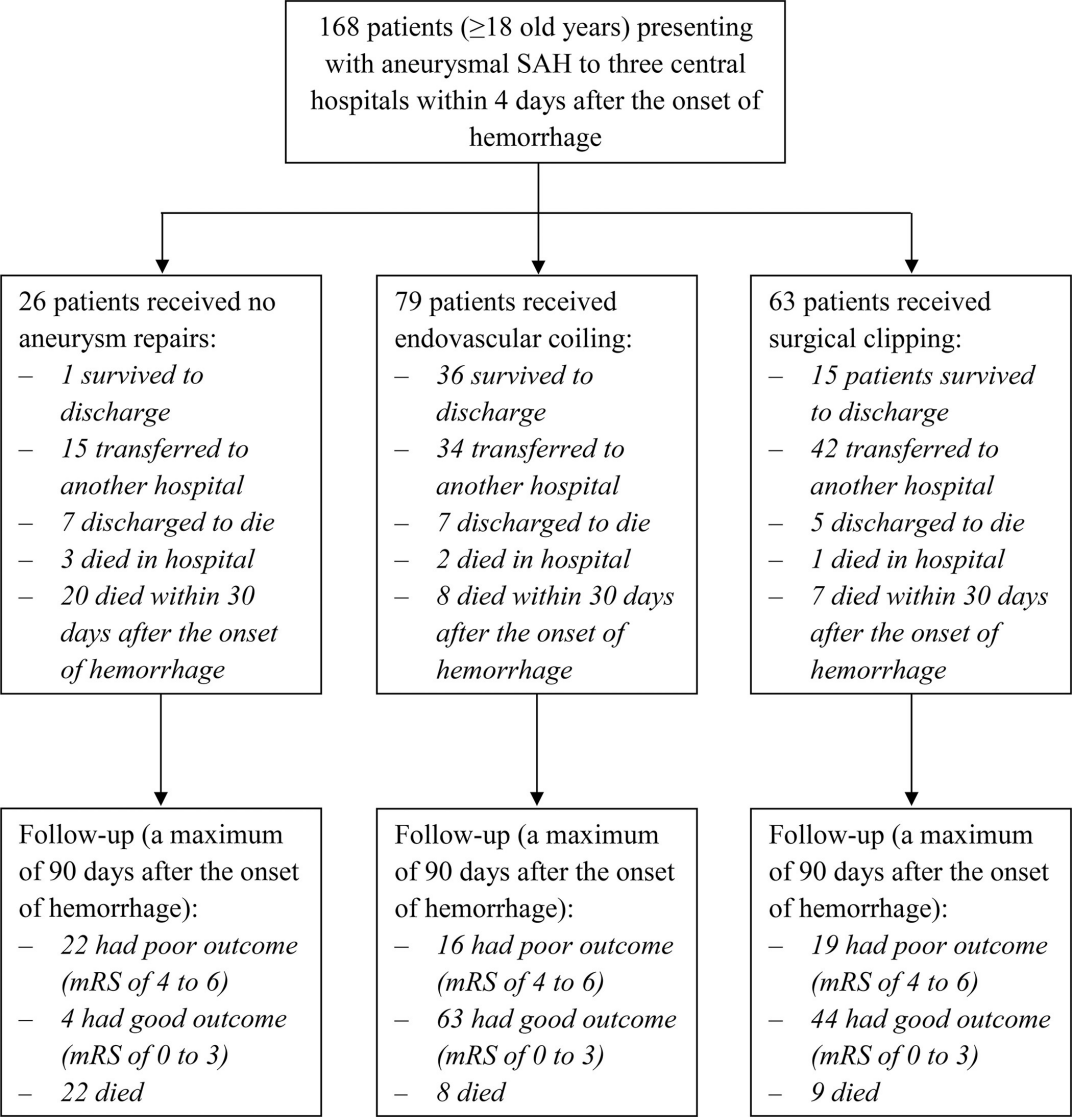
Flowchart of aneurysm treatment and outcome of patients with aneurysmal subarachnoid hemorrhage included in the study (mRS, modified Rankin Scale; SAH, subarachnoid hemorrhage).

During the observation period, a total of 168 patients presented to the study sites with aneurysmal SAH (Fig 1). Among these patients, 33.9% (57/168) of patients had a poor outcome, and 23.2% (39/168) died at 90 days of ictus. In addition, intracerebral (ICH) and intraventricular hemorrhages (IVH) were also detected on the admission CT scan in 24.4% (41/168) and 63.7% (107/168) of patients, respectively. Of the 168 patients with aneurysm SAH, 10/164 (6.1%) had late rebleeding of aneurysmal SAH, and DCI occurred in 17/163 (10.4%) patients. The characteristics, complications, clinical time course, and outcomes of patients were compared between methods of aneurysm treatment, as shown in Table 1.

**Table 1:** Demographic and baseline characteristics, management and outcomes of patients with aneurysmal subarachnoid hemorrhage according to methods of aneurysm treatment.

Variable	All cases	No aneurysm repair	Endovascular coiling	Surgical clipping	P^a^
	(n = 168)				
			(n = 79)	(n = 63)	
		(n = 26)			
Transferred from local hospitals, no. (%)	135 (80.4)	17 (65.4)	62 (78.5)	56 (88.9)	0.034
**Demographics**					
Age (year), median (IQR)	57 (48–67)	59.5 (50–70.5)	58 (49–67)	54 (46–67)	0.437
Gender (male), no. (%)	77 (45.8)	12 (46.2)	31 (39.2)	34 (54.0)	0.216
**Risk factors for aneurysmal subarachnoid hemorrhage**					
Cigarette smoking, no. (%)	63 (37.5)	9 (34.6)	25 (31.6)	29 (46.0)	0.201
Hypertension, no. (%)	104 (61.9)	14 (53.8)	46 (58.2)	44 (69.8)	0.240
Genetic risk, no. (%)	6 (3.6)	1 (3.8)	3 (3.8)	2 (3.2)	>0.999
Alcohol consumption, no. (%)	81 (48.2)	13 (50.0)	33 (41.8)	35 (55.6)	0.258
Sympathomimetic drugs, no. (%), n = 167	2 (1.2)	0 (0.0)	2 (2.5)	0 (0.0)	0.647
Estrogen deficiency, no. (%), n = 89	32 (36.0)	3 (21.4)	15 (32.6)	14 (48.3)	0.181
Antithrombotic therapy, no. (%)	3 (1.8)	0 (0.0)	2 (2.5)	1 (1.6)	>0.999
Elevated total cholesterol, no. (%)	8 (4.8)	0 (0.0)	5 (6.3)	3 (4.8)	0.556
**Comorbidities**					
Cerebrovascular disease, no. (%)	3 (1.8)	1 (3.8)	1 (1.3)	1 (1.6)	0.551
Chronic cardiac failure, n (%)	3 (1.8)	0 (0.0)	2 (2.5)	1 (1.6)	>0.999
Coronary artery disease/MI, no. (%)	3 (1.8)	0 (0.0)	2 (2.5)	1 (1.6)	>0.999
Active neoplasm, no. (%)	3 (1.8)	0 (0.0)	1 (1.3)	2 (3.2)	0.750
Chronic renal failure, no. (%)	2 (1.2)	1 (3.8)	1 (1.3)	0 (0.0)	0.426
Diabetes mellitus, n (%)	15 (8.9)	1 (3.8)	7 (8.9)	7 (11.1)	0.550
Hematological disease, no. (%)	2 (1.2)	0 (0.0)	2 (2.5)	0 (0.0)	0.645
**Onset symptoms**					
Sudden-onset, severe headache, no. (%)	147 (87.5)	19 (73.1)	67 (84.8)	61 (96.8)	0.005
Vomiting, no. (%)	102 (60.7)	12 (46.2)	56 (70.9)	34 (54.0)	0.031
Neck pain or stiffness, no. (%)	66 (39.3)	8 (30.8)	27 (34.2)	31 (49.2)	0.119
Photophobia, no. (%)	6 (3.6)	1 (3.8)	3 (3.8)	2 (3.2)	>0.999
Blurred or double vision, no. (%)	4 (2.4)	1 (3.8)	1 (1.3)	2 (3.2)	0.499
Brief loss of consciousness, no. (%)	70 (41.7)	21 (80.8)	26 (32.9)	23 (36.5)	<0.001
Seizures, no. (%)	9 (5.4)	2 (7.7)	2 (2.5)	5 (7.9)	0.260
**Clinical presentation on admission**					
GCS score, median (IQR)	14 (9–15)	6.5 (5.75–8.25)	15 (11–15)	15 (12–15)	<0.001
Focal neurological deficits, no. (%)	99 (58.9)	19 (73.1)	41 (51.9)	39 (61.9)	0.136
**Head imaging findings on admission**					
Blood filling the subarachnoid space, no. (%)					
Basal cistern, n = 165	79 (47.9)	15 (57.7)	44 (57.1)	20 (32.3)	0.008
Sylvian fissure, n = 167	157 (94.0)	25 (96.2)	77 (98.7)	55 (87.3)	0.017
Interhemispheric fissure, n = 166	84 (50.6)	16 (61.5)	46 (59.7)	22 (34.9)	0.007
Interpeduncular fossa, n = 166	83 (50.0)	18 (69.2)	34 (44.2)	31 (49.2)	0.086
Suprasellar cistern, n = 166	90 (54.2)	17 (65.4)	40 (51.9)	33 (52.4)	0.460
Ambient cistern, n = 166	81 (48.8)	18 (69.2)	34 (44.2)	29 (46.0)	0.074
Quadrigeminal cistern, n = 166	27 (16.3)	11 (42.3)	13 (16.9)	3 (4.8)	<0.001
IVH, no. (%)	107 (63.7)	22 (74.6)	48 (60.8)	37 (58.7)	0.053
ICH, no. (%)	41 (24.4)	11 (42.3)	9 (11.4)	21 (33.3)	0.001
ICH volume (mL), mean (SD), n = 41	29.29 (28.21)	52.30 (30.79)	5.71 (4.73)	27.34 (23.74)	<0.001
Subdural hemorrhage, no. (%)	10 (6.0)	1 (3.8)	3 (3.8)	6 (9.5)	0.341
Evans’ index, mean (SD), n = 145	0.30 (0.06)	0.30 (0.07)	0.29 (0.06)	0.32 (0.07)	0.461
**Severity of aneurysmal subarachnoid hemorrhage on admission**					
WFNS score, median (IQR)	2 (1–4)	4 (3–4)	1 (1–4)	1 (1–4)	<0.001
HH score, median (IQR)	2.5 (2–4)	5 (4.7–5)	2 (2–3)	2 (1–4)	<0.001
Fisher score, median (IQR)	4 (3–4)	4 (4–4)	4 (3–4)	4 (3–4)	0.088
**Laboratory investigations on admission**					
Platelets (G/L), mean (SD)	247.21 (76.12)	244.60 (73.26)	244.16 (83.08)	251.89 (69.20)	0.905
PT-INR, mean (SD)	1.00 (0.09)	1.05 (0.13)	0.99 (0.09)	1.00 (0.07)	0.096
**Complications**					
Late rebleeding, no. (%), n = 164	10 (6.1)	0	3 (3.8)	7 (11.9)	0.060
DCI, no. (%), n = 163	17 (10.4)	2 (7.7)	4 (5.3)	11 (18.0)	0.046
Acute hydrocephalus, no. (%)	76 (45.2)	14 (53.8)	34 (43.0)	28 (44.4)	0.622
Hyponatremia, no. (%)	34 (20.2)	3 (11.5)	20 (25.3)	11 (17.5)	0.249
Seizures, no. (%)	44 (26.2)	5 (19.2)	14 (17.7)	25 (39.7)	>0.009
Chronic hydrocephalus, no. (%), n = 83	5 (6.0)	0 (0.0)	3 (7.1)	2 (5.6)	>0.999
Ventriculitis, no. (%), n = 126	8 (6.3)	0 (0.0)	5 (7.7)	3 (7.5)	0.553
Pneumonia, no. (%)	29 (17.3)	1 (3.8)	16 (20.3)	12 (19.0)	0.141
Urinary tract infection, n (%)	3 (1.8)	0 (0.0)	3 (3.8)	0 (0.0)	0.281
**Clinical time course**					
Ictus to hospital arrival (hours), no. (%), n = 161					0.603
≤ 24 hours	69 (42.9)	13 (54.2)	30 (39.5)	26 (42.6)	
>24–72 hours	91 (56.5)	11 (45.8)	45 (59.2)	35 (57.4)	
>72 hours	1 (0.6)	0 (0.0)	1 (1.3)	0 (0.0)	
Length of hospitalization (days), mean (SD)	10.85 (9.40)	6.31 (10.56)	10.38 (9.98)	13.32 (7.27)	<0.001
**Clinical outcomes**					
Hospital discharge, no. (%)	52 (31.0)	1 (3.8)	36 (45.6)	15 (23.8)	<0.001
Transferred to another hospital, no. (%)	91 (54.2)	15 (57.7)	34 (43.0)	42 (66.7)	0.018
Discharged to die, no. (%)	19 (11.3)	7 (26.9)	7 (8.9)	5 (7.9)	0.023
*Deaths*:					
Died at 30 days of ictus, no. (%)	35 (20.8)	20 (76.9)	8 (10.1)	7 (11.1)	<0.001
Died at 90 days days of ictus, no. (%)	39 (23.2)	22 (84.6)	8 (10.1)	9 (14.3)	<0.001
*Neurological function*:					
mRS at 30 days of ictus, no. (%)					<0.001
Good (mRS of 0 to 3)	110 (65.5)	3 (11.5)	63 (79.7)	44 (69.8)	
Poor (mRS of 4 to 6)	58 (34.5)	23 (88.5)	16 (20.3)	19 (30.2)	
mRS at 90 days of ictus, no. (%)					<0.001
Good (mRS of 0 to 3)	111 (66.1)	4 (15.4)	63 (79.7)	44 (69.8)	
Poor (mRS of 4 to 6)	57 (33.9)	22 (84.6)	16 (20.3)	19 (30.2)	

^a^ Comparison between no aneurysm repair, endovascular coiling and surgical clipping.

Abbreviations: **COPD**, chronic obstructive pulmonary disease; **DCI**, delayed cerebral ischemia; **GCS**, Glasgow coma scale; **HH**, Hunt and Hess; **ICH**, intracerebral hemorrhage; **IQR**, interquartile range; **IVH**, intraventricular hemorrhage; **MI**, myocardial ischemia; **mRS**, modified Rankin scale; **SD**, standard deviation; **WFNS**, World Federation of Neurological Surgeons.

The median age of patients who had a good outcome (56 years; IQR: 46–65) was younger than that of patients who had a poor outcome (63 years; IQR: 53.5–71.5; p<0.001). There was a significant difference (p<0.001) between patients who had a good outcome and patients who had a poor outcome according to the severity of aneurysmal SAH (e.g., WFNS grading scale, Fisher scale, etc.). In addition, ICH (19.8% [22/111] vs. 33.3% [19/57], p = 0.054) and IVH (55.0% [61/111] vs. 80.7% [46/57], p = 0.001) were less commonly detected on the admission CT scan of patients who had good outcomes than those of patients who had poor outcomes. Although no difference was observed in surgical clipping between patients who had good outcomes (39.6%; 44/111) and patients who had poor outcomes (33.3%; 19/57; p = 0.424), there was a higher rate of endovascular coiling in patients who had good outcomes (56.8%; 63/111) than in patients who had poor outcomes (28.1%; 16/57; p<0.001). Both late rebleeding of aneurysmal SAH (0.9% [1/110] vs. 16.7% [9/54], p<0.001) and DCI (4.6% [5/108] vs. 21.8% [12/55], p = 0.001) were less common in patients who had good outcomes than in patients who had poor outcomes. The characteristics, treatment, and outcomes of patients were compared between patients who had a good outcome and patients who had a poor outcome, as shown in Table 2.

**Table 2:** Demographic and baseline characteristics, management and outcomes of patients with aneurysmal subarachnoid hemorrhage according to neurologic function at 90 days after the onset of hemorrhage.

Variable	All cases	mRS of 0 to 3	mRS of 4 to 6	P^a^
	(n = 168)	(n = 111)	(n = 57)	
Transferred from local hospitals, no. (%)	135 (80.4)	91 (82.0)	44 (77.2)	0.459
**Demographics**				
Age (year), median (IQR)	57 (48–67)	56 (46–65)	63 (53.5–71.5)	<0.001
Gender (male), no. (%)	77 (45.8)	51 (45.9)	26 (45.6)	0.967
**Risk factors for aneurysmal subarachnoid hemorrhage**				
Cigarette smoking, no. (%)	63 (37.5)	41 (36.9)	22 (38.6)	0.833
Hypertension, no. (%)	64 (38.1)	31 (27.9)	33 (57.9)	<0.001
Genetic risk, no. (%)	6 (3.6)	5 (4.5)	1 (1.8)	0.665
Alcohol consumption, no. (%)	81 (48.2)	53 (47.7)	28 (49.1)	0.866
Sympathomimetic drugs, no. (%), n = 167	2 (1.2)	2 (1.8)	0 (0.0)	0.548
Estrogen deficiency, no. (%), n = 89	32 (36.0)	19 (32.8)	13 (41.9)	0.488
Antithrombotic therapy, no. (%)	3 (1.8)	1 (0.9)	2 (3.5)	0.266
Elevated total cholesterol, no. (%)	8 (4.8)	5 (4.5)	3 (5.3)	>0.999
**Comorbidities**				
Cerebrovascular disease, no. (%)	3 (1.8)	2 (1.8)	1 (1.8)	>0.999
Chronic cardiac failure, no. (%)	3 (1.8)	1 (0.9)	2 (3.5)	0.266
Coronary artery disease/MI, no. (%)	3 (1.8)	1 (0.9)	2 (3.5)	0.266
Active neoplasm, no. (%)	3 (1.8)	2 (1.8)	1 (1.8)	>0.999
Chronic renal failure, no. (%)	2 (1.2)	1 (0.9)	1 (1.8)	>0.999
Diabetes mellitus, no. (%)	15 (8.9)	8 (7.2)	7 (12.3)	0.275
Hematological disease, no. (%)	2 (1.2)	1 (0.9)	1 (1.8)	>0.999
**Onset symptoms**				
Sudden-onset, severe headache, no. (%)	147 (87.5)	102 (91.9)	45 (78.9)	0.016
Vomiting, no. (%)	102 (60.7)	71 (61.0)	31 (54.4)	0.229
Neck pain or stiffness, no. (%)	66 (39.3)	43 (38.7)	23 (40.4)	0.839
Photophobia, no. (%)	6 (3.6)	5 (4.5)	1 (1.8)	0.665
Blurred or double vision, no. (%)	4 (2.4)	3 (2.7)	1 (1.8)	>0.999
Brief loss of consciousness, no. (%)	70 (41.7)	32 (28.8)	38 (66.7)	<0.001
Seizures, no. (%)	9 (5.4)	6 (5.4)	3 (5.3)	>0.999
**Clinical presentation on admission**				
GCS score, median (IQR)	14 (9–15)	15 (13–15)	8 (6.5–12)	<0.001
Focal neurological deficits, no. (%)	99 (58.9)	61 (55.0)	38 (66.7)	0.144
**Head imaging findings on admission**				
Blood filling the subarachnoid space, no. (%)				
Basal cistern, n = 165	79 (47.9)	43 (39.1)	36 (65.5)	0.001
Sylvian fissure, n = 167	157 (94.0)	103 (93.6)	54 (94.7)	>0.999
Interhemispheric fissure, n = 166	84 (50.6)	51 (46.4)	33 (58.9)	0.126
Interpeduncular fossa, n = 166	83 (50.0)	44 (40.0)	39 (69.6)	<0.001
Suprasellar cistern, n = 166	90 (54.2)	56 (50.9)	34 (60.7)	0.231
Ambient cistern, n = 166	81 (48.8)	43 (39.1)	38 (67.9)	<0.001
Quadrigeminal cistern, n = 166	27 (16.3)	10 (9.1)	17 (30.4)	<0.001
IVH, no. (%)	107 (63.7)	61 (55.0)	46 (80.7)	0.001
ICH, no. (%)	41 (24.4)	22 (19.8)	19 (33.3)	0.054
ICH volume (mL), mean (SD), n = 41	29.29 (28.21)	21.62 (21.95)	38.17 (32.43)	0.117
Subdural hemorrhage, no. (%)	10 (6.0)	5 (4.5)	5 (8.8)	0.310
Evans’ index, mean (SD), n = 145	0.30 (0.06)	0.29 (0.05)	0.32 (0.08)	0.018
**Severity of aneurysmal subarachnoid hemorrhage on admission**				
WFNS score, median (IQR)	2 (1–4)	1 (1–3)	4 (4–5)	<0.001
HH score, median (IQR)	2.5 (2–4)	2 (1–3)	5 (3–5)	<0.001
Fisher score, median (IQR)	4 (3–4)	4 (3–4)	4 (4–4)	<0.001
**Laboratory investigations on admission**				
Platelets (G/L), mean (SD)	247.21 (76.12)	247.68 (72.41)	246.32 (83.42)	0.939
PT-INR), mean (SD)	1.00 (0.09)	1.00 (0.09)	1.02 (0.10)	0.889
**Aneurysm repairs and other treatments**				
No aneurysm repair, no. (%)	26 (15.5)	4 (3.6)	22 (38.6)	<0.001
Endovascular coiling, no. (%)	79 (47.0)	63 (56.8)	16 (28.1)	<0.001
Surgical clipping, n (%)	63 (37.5)	44 (39.6)	19 (33.3)	0.424
Surgical hematoma evacuation or decompressive craniotomy, no. (%)	7 (4.2)	1 (0.9)	6 (10.5)	0.007
EVD, no. (%), n = 167	26 (15.6)	9 (8.2)	17 (29.8)	<0.001
IVF, no. (%), n = 168	3 (1.8)	1 (0.9)	2 (3.5)	0.266
**Complications**				
Late rebleeding, no. (%), n = 164	10 (6.1)	1 (0.9)	9 (16.7)	<0.001
DCI, no. (%), n = 163	17 (10.4)	5 (4.6)	12 (21.8)	0.001
Acute hydrocephalus, no. (%)	76 (45.2)	39 (35.1)	37 (64.9)	<0.001
Hyponatremia, no. (%)	34 (20.2)	19 (17.1)	15 (26.3)	0.160
Seizures, no. (%)	44 (26.2)	33 (29.7)	11 (19.3)	0.145
Chronic hydrocephalus, no. (%), n = 83	5 (6.0)	3 (4.3)	2 (15.4)	0.173
Ventriculitis, no. (%), n = 126	8 (6.3)	4 (5.0)	4 (8.7)	0.462
Pneumonia, no. (%)	29 (17.3)	10 (9.0)	19 (33.3)	<0.001
Urinary tract infection, no. (%)	3 (1.8)	3 (2.7)	0 (0.0)	0.552
**Clinical time course**				
Ictus to hospital arrival (hours), no. (%), n = 161				0.034
≤ 24 hours	69 (42.9)	38 (36.2)	31 (55.4)	
>24–72 hours	91 (56.5)	66 (62.9)	25 (44.6)	
>72 hours	1 (0.6)	1 (1.0)	0 (0.0)	
Length of hospitalization (days), mean (SD)	10.85 (9.40)	11.68 (9.42)	9.23 (9.24)	0.017
**Clinical outcomes**				
Hospital discharge, no. (%)	52 (31.0)	49 (44.1)	3 (5.3)	<0.001
Transferred to another hospital, no. (%)	91 (54.2)	61 (55.0)	30 (52.6)	0.775
Discharged to die, no. (%)	19 (11.3)	1 (0.96)	18 (31.6)	<0.001
*Deaths*:				
Died in hospital, no. (%)	6 (3.6)	0 (0.0)	6 (10.5)	0.001
Died within 30 days of ictus, no. (%)	35 (20.8)	0 (0.0)	35 (61.4)	
*Neurological function*:				
mRS score at 30 days of ictus, median (IQR)	1 (0–5)	1 (0–1)	6 (5–6)	<0.001
mRS at 30 days of ictus, no. (%)				<0.001
Good (mRS of 0 to 3)	110 (65.5)	110 (99.1)	0 (0.0)	
Poor (mRS of 4 to 6)	58 (34.5)	1 (0.9)	57 (100.0)	

^a^ Comparison between mRS of 0 to 3 and mRS of 4 to 6.

Abbreviations: **COPD**, chronic obstructive pulmonary disease; **DCI**, delayed cerebral ischemia; **EVD**, external ventricular drain; **GCS**, Glasgow coma scale; **HH**, Hunt and Hess; **ICH**, intracerebral hemorrhage; **IQR**, interquartile range; **IVF**: Intraventricular fibrinolysis; **IVH**, intraventricular hemorrhage; **MI**, myocardial ischemia; **mRS**, modified Rankin scale; **SD**, standard deviation; **WFNS**, World Federation of Neurological Surgeons.

### Factors associated with poor outcome and mortality

Several factors were independently associated with poor outcome on patients with aneurysm SAH at both 30 and 90 days of ictus, including blood filling the basal cistern (OR: 3.736, 95% CI: 1.082–12.899 and OR: 4.062, 95% CI: 1.102–14.981, respectively); admission WFNS grade of IV (OR: 14.367, 95% CI: 3.155–65.429 and OR: 15.285, 95% CI: 3.096–75.466, respectively) and grade of V (OR: 54.391, 95% CI: 4.831–612.362 and OR: 162.965, 95% CI: 9.975–2662.318, respectively); endovascular coiling (OR: 0.032, 95% CI: 0.005–0.194 and OR: 0.033, 95% CI: 0.005–0.235, respectively); surgical clipping (OR: 0.044, 95% CI: 0.007–0.291 and OR: 0.046, 95% CI: 0.006–0.370, respectively); late rebleeding (OR: 71.142, 95% CI: 4.915–1029.672 and OR: 97.624, 95% CI: 5.653–1686.010, respectively); and DCI (OR: 11.581, 95% CI: 1.897–70.698 and OR: 15.209, 95% CI: 2.321–99.673, respectively). Further analysis also showed that late rebleeding (OR: 10.153, 95% CI: 1.557–66.219 and OR: 22.588, 95% CI: 3.619–141.000) was independently associated with mortality at both 30 and 90 days of ictus. Factors associated with poor outcome and mortality are shown in Table 3.

**Table 3:** Factors associated with poor outcome and mortality of patients with aneurysmal subarachnoid hemorrhage: Multivariate logistic regression analyses.

Factor	Unit	OR	95.0% CI for OR		p-value
			Lower	Upper	
**Factors associated with poor outcome at 90 days of ictus**					
Age (years)					
20–39	%	-	-	-	0.052
40–59	%	16.272	0.858	308.485	0.063
≥ 60	%	39.045	1.938	786.699	0.017
Hypertension	%	3.842	0.874	16.881	0.075
Blood filling the subarachnoid space					
Basal cistern	%	4.062	1.102	14.981	0.035
WFNS scale					
Grade I	%	-	-	-	0.002
Grade II	%	3.744	0.338	41.441	0.282
Grade III	%	1.480	0.036	60.288	0.836
Grade IV	%	15.285	3.096	75.466	0.001
Grade V	%	162.965	9.975	2662.318	<0.001
Aneurysm repairs					
No aneurysm repair	%	-	-	-	0.003
Endovascular coiling	%	0.033	0.005	0.235	0.001
Surgical clipping	%	0.046	0.006	0.370	0.004
EVD	%	5.016	1.000	25.158	0.050
Late rebleeding	%	97.624	5.653	1686.010	0.002
DCI	%	15.209	2.321	99.673	0.005
Constant	%	0.005			0.002
**Factors associated with mortality at 90 days of ictus**					
Hypertension	%	4.707	1.224	18.107	0.024
Blood filling the subarachn-oid space					
Quadrigeminal cistern	%	4.279	0.760	24.086	0.099
WFNS scale					
Grade I	%	-	-	-	0.068
Grade II	%	4.632	0.317	67.657	0.263
Grade III	%	10.140	0.570	180.475	0.115
Grade IV	%	14.038	2.294	85.911	0.004
Grade V	%	14.021	1.299	151.332	0.030
Aneurysm repairs					
No aneurysm repair	%	-	-	-	<0.001
Endovascular coiling	%	0.022	0.004	0.128	<0.001
Surgical clipping	%	0.024	0.004	0.155	<0.001
Late rebleeding	%	22.588	3.619	141.000	0.001
Constant		0.197			0.143
**Factors associated with poor outcome at 30 days of ictus**					
Hypertension	%	5.822	1.588	21.342	0.008
Blood filling the subarachn-oid space					
Basal cistern	%	3.736	1.082	12.899	0.037
WFNS scale					
Grade I	%	-	-	-	0.003
Grade II	%	1.842	0.209	16.256	0.583
Grade III	%	1.740	0.073	41.440	0.732
Grade IV	%	14.367	3.155	65.429	0.001
Grade V	%	54.391	4.831	612.362	0.001
Aneurysm repairs					
No aneurysm repair	%	-	-	-	0.001
Endovascular coiling	%	0.032	0.005	0.194	<0.001
Surgical clipping	%	0.044	0.007	0.291	0.001
EVD	%	4.202	1.006	17.556	0.049
Late rebleeding	%	71.142	4.915	1029.672	0.002
DCI	%	11.581	1.897	70.698	0.008
Constant	%	0.143			0.070
**Factors associated with mortality at 30 days of ictus**					
Blood filling the subarachn-oid space					
Quadrigeminal cistern	%	5.958	1.155	30.730	0.033
WFNS scale					
Grade I	%	-	-	-	0.098
Grade II	%	20.290	1.218	338.093	0.036
Grade III	%	30.342	1.055	872.794	0.046
Grade IV	%	26.787	2.599	276.094	0.006
Grade V	%	24.131	1.705	341.556	0.019
Aneurysm repairs					
No aneurysm repair	%	-	-	-	<0.001
Endovascular coiling	%	0.045	0.009	0.228	<0.001
Surgical clipping	%	0.038	0.007	0.227	<0.001
Late rebleeding	%	10.153	1.557	66.219	0.015
DCI	%	6.602	1.127	38.673	0.036
Constant	%	0.082			0.055

Abbreviations: **CI**, confidence interval; **DCI**, delayed cerebral ischemia; **EVD**, external ventricular drain; **OR**, odds ratio; **WFNS**, World Federation of Neurological Surgeons.

## Discussion

In this study, most of the patients with aneurysmal SAH (80.4%) were transferred from local hospitals to participating hospitals (Table 1). Participating hospitals are the central hospital in northern Vietnam and are responsible for treating patients with severe conditions from lower-level hospitals as well as educating and training medical providers at local hospitals [30]. This is one of the main reasons for the present study, namely, to examine the outcome of patients transferred from local hospitals.

Our study shows that poor outcomes at 30 and 90 days of ictus were observed in over a third (34.5% and 33.9%, respectively) of patients with aneurysmal SAH; death at 30 and 90 days of ictus was observed in over a fifth (20.8% and 23.2%, respectively) of patients (Table 1). The mortality rates of our patients at 30 and 90 days of ictus were lower than the rates reported in previous studies (22–25% and 25–29%, respectively) [5, 38]. These differences might be because our cohort is likely to be selected, as many patients with aneurysmal SAH in Vietnam are not transferred to the central hospital and might die in the local hospital, as well as die outside of the hospital [39], and might also be attributed to our study only including patients presenting to the participating hospitals within 4 days of ictus and excluding patients for whom admission GCS was unable to score (e.g., in the intubated patients). Therefore, our cohort may not reflect all aneurysmal SAHs in the region.

In our study, the proportions of ICH and IVH detected on admission CT scan were higher than the rates reported in a previous study (20.9%, 1120/5362; 49.7%, 2628/5362, respectively) [40]. While most rebleeding occurs in the subarachnoid space, bleeding can also occur in the intraparienchymal, intraventricular, or subdural compartments. After aneurysmal SAH, the risk of early rebleeding is 4 to 14% in the first 24 hours [1, 16]. Our study shows that over half of patients arrived in participating hospitals after 24 hours of ictus, and nearly half of patients who did not receive aneurysm repair took over 24 hours from ictus to participating hospital arrival (Table 1). Therefore, these differences in terms of ICH and IVH rates might be attributed to early rebleeding in our study. Additionally, a unique characteristic of patients with aneurysmal SAH or other clinical conditions in Vietnam is that many of these patients are diagnosed with SAH in local hospitals and are then transferred to a central hospital if their conditions become severe [30]. This leads to delayed diagnosis and delayed initiation of aneurysm repair for aneurysmal SAH, which can, in turn, lead to high complications, poor outcomes, and mortality rates [27, 28, 41]. Thus, to reduce poor outcomes and mortality, improvements are needed in human, medical, and sociological resources at local levels.

In our study, the proportion of patients with aneurysmal SAH who had a WFNS grade of III or more was 44% (74/168), which was higher than the rate reported in a previous study (35.6%; 1910/5362) [40], of which most patients (92.3%; 24/26) who did not receive aneurysm repair had a WFNS grade of III or more (S1 Table in S1 File). In previous studies, several factors were identified as predictors of rebleeding, including longer time to aneurysm treatment and worse neurologic status on admission [16, 42–46]. Additionally, aneurysm treatment is the only effective treatment for the prevention of rebleeding [16]. Therefore, our patients with aneurysmal SAH should have emergency aneurysm repair. In addition to the high risk of early rebleeding after aneurysmal SAH, patients with aneurysmal SAH remain at an elevated risk of rebleeding for 30 days after the initial rupture if the aneurysm is not treated [1, 16]. In our study, a substantial number of patients who did not receive aneurysm repair had a poor WFNS grade on admission and consequently received “discharged to die” decisions (Table 1). In a literature review, the mortality associated with rebleeding was reported to be as high as 70% [47]. However, our study shows that both surgical clipping and endovascular coiling were inversely and independently associated with poor outcomes at both 30 and 90 days of ictus (Table 3). Thus, to reduce the poor outcome and mortality, increasing the number of aneurysm repairs and performing earlier aneurysm treatment with surgical clipping or endovascular coiling are needed.

Our study also shows that the rates of acute hydrocephalus, late rebleeding, DCI, and nosocomial pneumonia were significantly more often observed in patients who had poor outcomes than in patients who had good outcomes (Table 2). However, only late rebleeding and DCI were independently associated with poor outcomes at both 30 and 90 days of ictus (Table 3). Previous studies have also shown that DCI is a frequent complication of SAH; it contributes substantially to morbidity and mortality after SAH [19, 48]. However, the risk of late rebleeding is low but is more common after endovascular coiling (2.9%) than after surgical clipping (0.9%) [24]. Therefore, improvements are needed in both aneurysm treatments and neurocritical care.

Our study has some limitations. Our data are from a selected population of cases that were transferred to the three highest-level public sector hospitals in Vietnam. Therefore, the number of patients with aneurysmal SAH is likely to be considerably higher. Additionally, data were missing for some variables, e.g., in only 83 patients were the data recorded if chronic hydrocephalus was given or not. Moreover, this study only included patients presenting to the participating hospitals within 4 days of ictus and excluded patients for whom admission GCS was unable to score (e.g., the patients intubated before arrival in the central hospital). These factors resulted in incomplete enrollment of patients in the database of the study, which may have introduced selection bias [49]. These limitations might account for some differences in figures reported from other countries.

## Conclusions

This study investigated selected cohort of patients with aneurysmal SAH presenting to central hospitals. Patients with aneurysmal SAH were transferred from local to central hospitals in northern Vietnam with high poor outcomes and mortality rates. At 30 and 90 days of ictus, admission WFNS grades of IV and V and late rebleeding were independently associated with poor outcomes and deaths, and endovascular coiling and surgical clipping were inversely and independently associated with poor outcomes and deaths. DCI was also independently associated with poor outcomes at both 30 and 90 days of ictus. To reduce the poor outcome and mortality in patients with aneurysmal SAH, the management of aneurysmal SAH in Vietnam needs to be enhanced through, for example, increasing the number of aneurysm repairs, performing earlier aneurysm treatment by surgical clipping or endovascular coiling, and improving both aneurysm repairs and neurocritical care.

## Supporting information

S1 Checklist STROBE statement—checklist of items that should be included in reports of *cohort studies*. (DOCX)

S1 File (DOCX)

S2 File (PDF)

S1 Dataset (XLSX)

## References

[1] Lawton MT, Vates GE. Subarachnoid Hemorrhage. The New England journal of medicine. 2017;377(3):257–66. doi: 10.1056/NEJMcp1605827 . 28723321

[2] Macdonald RL, Schweizer TA. Spontaneous subarachnoid haemorrhage. Lancet. 2017;389(10069):655–66. Epub 2016/09/18. doi: 10.1016/S0140-6736(16)30668-7 . 27637674

[3] Hop JW, Rinkel GJ, Algra A, van Gijn J. Case-fatality rates and functional outcome after subarachnoid hemorrhage: a systematic review. Stroke; a journal of cerebral circulation. 1997;28(3):660–4. Epub 1997/03/01. doi: 10.1161/01.str.28.3.660 . 9056628

[4] Stegmayr B, Eriksson M, Asplund K. Declining mortality from subarachnoid hemorrhage: changes in incidence and case fatality from 1985 through 2000. Stroke; a journal of cerebral circulation. 2004;35(9):2059–63. Epub 2004/07/24. doi: 10.1161/01.STR.0000138451.07853.b6 . 15272133

[5] Mackey J, Khoury JC, Alwell K, Moomaw CJ, Kissela BM, Flaherty ML, et al. Stable incidence but declining case-fatality rates of subarachnoid hemorrhage in a population. Neurology. 2016;87(21):2192–7. Epub 2016/10/23. doi: 10.1212/WNL.0000000000003353 ; PubMed Central PMCID: PMC5123555. 27770074 PMC5123555

[6] Huang J, van Gelder JM. The probability of sudden death from rupture of intracranial aneurysms: a meta-analysis. Neurosurgery. 2002;51(5):1101–5; discussion 5–7. Epub 2002/10/18. doi: 10.1097/00006123-200211000-00001 . 12383354

[7] Lindbohm JV, Kaprio J, Jousilahti P, Salomaa V, Korja M. Risk Factors of Sudden Death From Subarachnoid Hemorrhage. Stroke; a journal of cerebral circulation. 2017;48(9):2399–404. Epub 2017/07/26. doi: 10.1161/STROKEAHA.117.018118 . 28739833

[8] Abulhasan YB, Alabdulraheem N, Simoneau G, Angle MR, Teitelbaum J. Mortality after Spontaneous Subarachnoid Hemorrhage: Causality and Validation of a Prediction Model. World neurosurgery. 2018;112:e799–e811. Epub 2018/02/08. doi: 10.1016/j.wneu.2018.01.160 . 29410174

[9] Vergouwen MD, Jong-Tjien-Fa AV, Algra A, Rinkel GJ. Time trends in causes of death after aneurysmal subarachnoid hemorrhage: A hospital-based study. Neurology. 2016;86(1):59–63. Epub 2015/11/22. doi: 10.1212/WNL.0000000000002239 . 26590269

[10] Roos YB, de Haan RJ, Beenen LF, Groen RJ, Albrecht KW, Vermeulen M. Complications and outcome in patients with aneurysmal subarachnoid haemorrhage: a prospective hospital based cohort study in the Netherlands. Journal of neurology, neurosurgery, and psychiatry. 2000;68(3):337–41. Epub 2000/02/16. doi: 10.1136/jnnp.68.3.337 ; PubMed Central PMCID: PMC1736841. 10675216 PMC1736841

[11] Molyneux AJ, Kerr RS, Birks J, Ramzi N, Yarnold J, Sneade M, et al. Risk of recurrent subarachnoid haemorrhage, death, or dependence and standardised mortality ratios after clipping or coiling of an intracranial aneurysm in the International Subarachnoid Aneurysm Trial (ISAT): long-term follow-up. The Lancet Neurology. 2009;8(5):427–33. Epub 2009/03/31. doi: 10.1016/S1474-4422(09)70080-8 ; PubMed Central PMCID: PMC2669592. 19329361 PMC2669592

[12] Wermer MJ, Greebe P, Algra A, Rinkel GJ. Long-term mortality and vascular event risk after aneurysmal subarachnoid haemorrhage. Journal of neurology, neurosurgery, and psychiatry. 2009;80(12):1399–401. Epub 2009/11/18. doi: 10.1136/jnnp.2008.157586 . 19917822

[13] Nieuwkamp DJ, Algra A, Blomqvist P, Adami J, Buskens E, Koffijberg H, et al. Excess mortality and cardiovascular events in patients surviving subarachnoid hemorrhage: a nationwide study in Sweden. Stroke; a journal of cerebral circulation. 2011;42(4):902–7. Epub 2011/02/19. doi: 10.1161/STROKEAHA.110.602722 . 21330628

[14] Korja M, Silventoinen K, Laatikainen T, Jousilahti P, Salomaa V, Kaprio J. Cause-specific mortality of 1-year survivors of subarachnoid hemorrhage. Neurology. 2013;80(5):481–6. Epub 2013/01/11. doi: 10.1212/WNL.0b013e31827f0fb5 ; PubMed Central PMCID: PMC3590048. 23303843 PMC3590048

[15] Koroknay-Pál P, Laakso A, Lehto H, Seppä K, Kivisaari R, Hernesniemi J, et al. Long-term excess mortality in pediatric patients with cerebral aneurysms. Stroke; a journal of cerebral circulation. 2012;43(8):2091–6. Epub 2012/06/14. doi: 10.1161/strokeaha.112.650077 . 22693125

[16] Connolly ES Jr., Rabinstein AA, Carhuapoma JR, Derdeyn CP, Dion J, Higashida RT, et al. Guidelines for the management of aneurysmal subarachnoid hemorrhage: a guideline for healthcare professionals from the American Heart Association/american Stroke Association. Stroke; a journal of cerebral circulation. 2012;43(6):1711–37. Epub 05/03. doi: 10.1161/STR.0b013e3182587839 . 22556195

[17] Schatlo B, Fung C, Stienen MN, Fathi AR, Fandino J, Smoll NR, et al. Incidence and Outcome of Aneurysmal Subarachnoid Hemorrhage: The Swiss Study on Subarachnoid Hemorrhage (Swiss SOS). Stroke; a journal of cerebral circulation. 2021;52(1):344–7. Epub 2020/12/05. doi: 10.1161/strokeaha.120.029538 . 33272133

[18] Mayer SA, Kreiter KT, Copeland D, Bernardini GL, Bates JE, Peery S, et al. Global and domain-specific cognitive impairment and outcome after subarachnoid hemorrhage. Neurology. 2002;59(11):1750–8. Epub 2002/12/11. doi: 10.1212/01.wnl.0000035748.91128.c2 . 12473764

[19] Rosengart AJ, Schultheiss KE, Tolentino J, Macdonald RL. Prognostic factors for outcome in patients with aneurysmal subarachnoid hemorrhage. Stroke; a journal of cerebral circulation. 2007;38(8):2315–21. Epub 2007/06/16. doi: 10.1161/STROKEAHA.107.484360 . 17569871

[20] Wong GK, Lam SW, Ngai K, Wong A, Siu D, Poon WS, et al. Cognitive domain deficits in patients with aneurysmal subarachnoid haemorrhage at 1 year. Journal of neurology, neurosurgery, and psychiatry. 2013;84(9):1054–8. Epub 2013/04/23. doi: 10.1136/jnnp-2012-304517 ; PubMed Central PMCID: PMC3756437. 23606736 PMC3756437

[21] Nieuwkamp DJ, Setz LE, Algra A, Linn FHH, de Rooij NK, Rinkel GJE. Changes in case fatality of aneurysmal subarachnoid haemorrhage over time, according to age, sex, and region: a meta-analysis. The Lancet Neurology. 2009;8(7):635–42. Epub 06/06. doi: 10.1016/S1474-4422(09)70126-7 . 19501022

[22] Khan AU, Dulhanty L, Vail A, Tyrrell P, Galea J, Patel HC. Impact of specialist neurovascular care in subarachnoid haemorrhage. Clinical neurology and neurosurgery. 2015;133:55–60. Epub 2015/04/04. doi: 10.1016/j.clineuro.2015.03.006 . 25839916

[23] Pickard JD, Murray GD, Illingworth R, Shaw MD, Teasdale GM, Foy PM, et al. Effect of oral nimodipine on cerebral infarction and outcome after subarachnoid haemorrhage: British aneurysm nimodipine trial. Bmj. 1989;298(6674):636–42. Epub 1989/03/11. doi: 10.1136/bmj.298.6674.636 ; PubMed Central PMCID: PMC1835889. 2496789 PMC1835889

[24] Molyneux AJ, Kerr RS, Yu LM, Clarke M, Sneade M, Yarnold JA, et al. International subarachnoid aneurysm trial (ISAT) of neurosurgical clipping versus endovascular coiling in 2143 patients with ruptured intracranial aneurysms: a randomised comparison of effects on survival, dependency, seizures, rebleeding, subgroups, and aneurysm occlusion. Lancet. 2005;366(9488):809–17. Epub 2005/09/06. doi: 10.1016/S0140-6736(05)67214-5 . 16139655

[25] Rivero-Arias O, Gray A, Wolstenholme J. Burden of disease and costs of aneurysmal subarachnoid haemorrhage (aSAH) in the United Kingdom. Cost effectiveness and resource allocation: C/E. 2010;8:6. Epub 2010/04/29. doi: 10.1186/1478-7547-8-6; PubMed Central PMCID: PMC2874525. 20423472 PMC2874525

[26] The World Bank. The World Bank In Vietnam. Washington: The World Bank; 2020 [updated October 6, 2020; cited 2021 March 31]. Available from: https://www.worldbank.org/en/country/vietnam/overview.

[27] Cong NH. Stroke Care in Vietnam. International Journal of Stroke. 2007;2(4):279–80. doi: 10.1111/j.1747-4949.2007.00149.x . 18705929

[28] Nguyen TH, Gall S, Cadilhac DA, Nguyen H, Terry D, Pham BN, et al. Processes of Stroke Unit Care and Outcomes at Discharge in Vietnam: Findings from the Registry of Stroke Care Quality (RES-Q) in a Major Public Hospital. Journal of Stroke Medicine. 2019;2(2):119–27. doi: 10.1177/2516608519869132

[29] Luong QC, Manabe T, Do NS, Nguyen VC, Fujikura Y, Nguyen GB, et al. Clinical epidemiology and mortality on patients with acute respiratory distress syndrome (ARDS) in Vietnam. PloS one. 2019;14(8):e0221114. doi: 10.1371/journal.pone.0221114 31415662 PMC6695190

[30] Takashima K, Wada K, Tra TT, Smith DR. A review of Vietnam’s healthcare reform through the Direction of Healthcare Activities (DOHA). Environmental health and preventive medicine. 2017;22(1):74. Epub 2017/11/23. doi: 10.1186/s12199-017-0682-z; PubMed Central PMCID: PMC5664805. 29165160 PMC5664805

[31] Suarez JI, Sheikh MK, Macdonald RL, Amin-Hanjani S, Brown RD Jr., de Oliveira Manoel AL, et al. Common Data Elements for Unruptured Intracranial Aneurysms and Subarachnoid Hemorrhage Clinical Research: A National Institute for Neurological Disorders and Stroke and National Library of Medicine Project. Neurocritical care. 2019;30(Suppl 1):4–19. Epub 2019/05/16. doi: 10.1007/s12028-019-00723-6 . 31087257

[32] The World Federation of Neurological Surgeons (WFNS) Committee. Report of World Federation of Neurological Surgeons Committee on a Universal Subarachnoid Hemorrhage Grading Scale. Journal of neurosurgery. 1988;68(6):985–6. Epub 1988/06/01. doi: 10.3171/jns.1988.68.6.0985 . 3131498

[33] Hunt WE, Hess RM. Surgical risk as related to time of intervention in the repair of intracranial aneurysms. Journal of neurosurgery. 1968;28(1):14–20. Epub 1968/01/01. doi: 10.3171/jns.1968.28.1.0014 . 5635959

[34] Ralph W, Jack W, Mukesh GH, John WC, Stephen EJ, Jay WP. Primer of Diagnostic Imaging, 4th Edition. Edition t, editor. Philadelphia, PA 19103–2899: Mosby Elsevier; 2006. 1152 pages p.

[35] Luong CQ, Nguyen AD, Nguyen CV, Mai TD, Nguyen TA, Do SN, et al. Effectiveness of Combined External Ventricular Drainage with Intraventricular Fibrinolysis for the Treatment of Intraventricular Haemorrhage with Acute Obstructive Hydrocephalus. Cerebrovascular Diseases Extra. 2019;9(2):77–89. doi: 10.1159/000501530 31408859 PMC6751468

[36] van Swieten JC, Koudstaal PJ, Visser MC, Schouten HJ, van Gijn J. Interobserver agreement for the assessment of handicap in stroke patients. Stroke; a journal of cerebral circulation. 1988;19(5):604–7. Epub 1988/05/01. doi: 10.1161/01.str.19.5.604 . 3363593

[37] Gaberel T, Gakuba C, Fournel F, Le Blanc E, Gaillard C, Peyro-Saint-Paul L, et al. FIVHeMA: Intraventricular fibrinolysis versus external ventricular drainage alone in aneurysmal subarachnoid hemorrhage: A randomized controlled trial. Neuro-Chirurgie. 2019;65(1):14–9. Epub 2019/01/15. doi: 10.1016/j.neuchi.2018.11.004 . 30638547

[38] Øie LR, Solheim O, Majewska P, Nordseth T, Müller TB, Carlsen SM, et al. Incidence and case fatality of aneurysmal subarachnoid hemorrhage admitted to hospital between 2008 and 2014 in Norway. Acta neurochirurgica. 2020;162(9):2251–9. Epub 2020/07/01. doi: 10.1007/s00701-020-04463-x ; PubMed Central PMCID: PMC7415018. 32601806 PMC7415018

[39] Do SN, Luong CQ, Pham DT, Nguyen CV, Ton TT, Pham TTN, et al. Survival after out-of-hospital cardiac arrest, Viet Nam: multicentre prospective cohort study. Bulletin of the World Health Organization. 2021;99(1):50–61. Epub 10/28. doi: 10.2471/BLT.20.269837 PMC7924896. 33658734 PMC7924896

[40] Wan A, Jaja BN, Schweizer TA, Macdonald RL. Clinical characteristics and outcome of aneurysmal subarachnoid hemorrhage with intracerebral hematoma. Journal of neurosurgery. 2016;125(6):1344–51. Epub 2016/02/27. doi: 10.3171/2015.10.JNS151036 . 26918469

[41] Chinh LQ, Manabe T, Son DN, Chi NV, Fujikura Y, Binh NG, et al. Clinical epidemiology and mortality on patients with acute respiratory distress syndrome (ARDS) in Vietnam. PloS one. 2019;14 (8):e0221114–e. doi: 10.1371/journal.pone.0221114 ; PubMed Central PMCID: PMC31415662. 31415662 PMC6695190

[42] Hillman J, Fridriksson S, Nilsson O, Yu Z, Saveland H, Jakobsson KE. Immediate administration of tranexamic acid and reduced incidence of early rebleeding after aneurysmal subarachnoid hemorrhage: a prospective randomized study. Journal of neurosurgery. 2002;97(4):771–8. Epub 2002/10/31. doi: 10.3171/jns.2002.97.4.0771 . 12405362

[43] Naidech AM, Janjua N, Kreiter KT, Ostapkovich ND, Fitzsimmons B-F, Parra A, et al. Predictors and impact of aneurysm rebleeding after subarachnoid hemorrhage. Archives of neurology. 2005;62(3):410–6. doi: 10.1001/archneur.62.3.410 . 15767506

[44] Kassell NF, Torner JC. Aneurysmal rebleeding: a preliminary report from the Cooperative Aneurysm Study. Neurosurgery. 1983;13(5):479–81. doi: 10.1227/00006123-198311000-00001 . 6646375

[45] Tang C, Zhang TS, Zhou LF. Risk factors for rebleeding of aneurysmal subarachnoid hemorrhage: a meta-analysis. PloS one. 2014;9(6):e99536. Epub 2014/06/10. doi: 10.1371/journal.pone.0099536; PubMed Central PMCID: PMC4049799. 24911172 PMC4049799

[46] Darkwah Oppong M, Gümüs M, Pierscianek D, Herten A, Kneist A, Wrede K, et al. Aneurysm rebleeding before therapy: a predictable disaster? Journal of neurosurgery. 2018:1–8. Epub 2018/12/14. doi: 10.3171/2018.7.JNS181119 . 30544356

[47] Taqi MA, Torbey MT. Subarachnoid Hemorrhage. In: Edward M. Manno, editor. Emergency Management in Neurocritical Care. Noida, India: John Wiley & Sons, Ltd.; 2012. p. 37–44.

[48] Macdonald RL, Hunsche E, Schüler R, Wlodarczyk J, Mayer SA. Quality of life and healthcare resource use associated with angiographic vasospasm after aneurysmal subarachnoid hemorrhage. Stroke; a journal of cerebral circulation. 2012;43(4):1082–8. Epub 2012/02/14. doi: 10.1161/STROKEAHA.111.634071 . 22328549

[49] Krumholz HM. Registries and selection bias: the need for accountability. Circulation Cardiovascular quality and outcomes. 2009;2(6):517–8. Epub 2009/12/25. doi: 10.1161/CIRCOUTCOMES.109.916601 . 20031886

