# Supplementary material for: Clinical characteristics and factors relating to poor outcome in patients with aneurysmal subarachnoid hemorrhage in Vietnam: A multicenter prospective cohort study
Source: PLoS One. 2021 Aug 13;16(8):e0256150. doi: 10.1371/journal.pone.0256150 (PMC8362943; doi:10.1371/journal.pone.0256150)
Supplement: S1 File — (DOCX) [file pone.0256150.s002.docx]

**S1 File**

**S1 Fig**. Kaplan-Meier curve demonstrating survival at 90 days after the onset of hemorrhage in the treatment group of patients with aneurysmal subarachnoid hemorrhage. The treatment groups were categorized as the no aneurysm repair, endovascular coiling or surgical clipping. A statistically significant association in survival exists between the curves (p=<0.001with the log-rank test). The blue line represents the no aneurysm repair, the green line represents the endovascular coiling and the yellow line represents the surgical clipping.


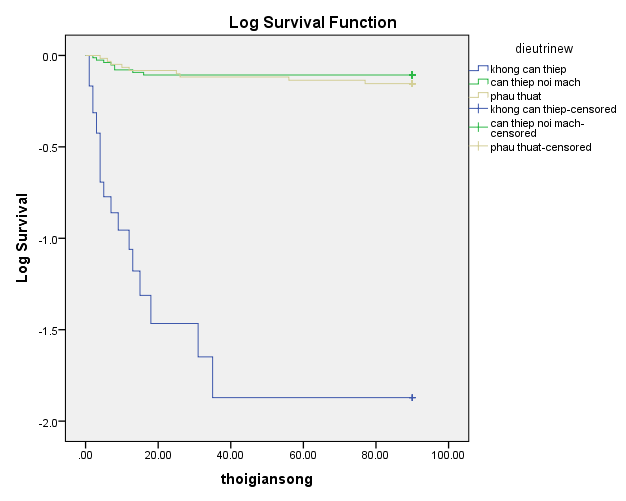


| **Overall Comparisons** | | | |
| --- | --- | --- | --- |
|  | Chi-Square | df | Sig. |
| Log Rank (Mantel-Cox) | 110.634 | 2 | .000 |
| Test of equality of survival distributions for the different levels of dieutrinew. | | | |

**S2 Fig**. Kaplan-Meier curve demonstrating survival at 90 days after the onset of hemorrhage in the treatment subgroup of patients with aneurysmal subarachnoid hemorrhage. The treatment subgroups were categorized as the endovascular coiling or surgical clipping. A statistically nonsignificant difference in survival exists between the curves (p=0.956 with the log-rank test). The green line represents the endovascular coiling and the yellow line represents the surgical clipping.


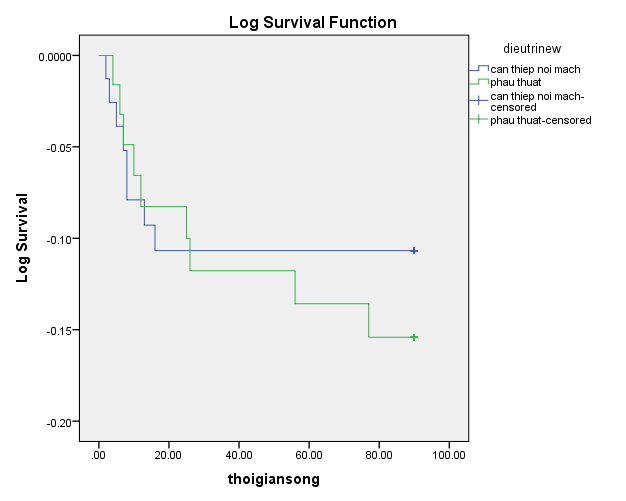


| **Overall Comparisons** | | | |
| --- | --- | --- | --- |
|  | Chi-Square | df | Sig. |
| Log Rank (Mantel-Cox) | .485 | 1 | .486 |
| Test of equality of survival distributions for the different levels of dieutrinew. | | | |

**S1 Table**. Factors associated with poor outcome of patients with aneurysmal subarachnoid hemorrhage at 90 days after the onset of hemorrhage: bivariate regression analyses

|  | Frequency | OR | | 95.0% CI for OR | | p-value |
| --- | --- | --- | --- | --- | --- | --- |
|  |  |  |  | Lower | Upper |  |
| Transferred from local hospitals, n (%) |  |  | |  |  |  |
| Hospital taken to |  |  | |  |  |  |
| Viet Duc | 22 | - | | - | - | - |
| Bach Mai | 129 | 5.926 | | 1.327 | 26.472 | 0.020 |
| Hanoi Medical University | 17 | 7.000 | | 1.222 | 40.089 | 0.029 |
| **Demographics** | | | | | | |
| Age (year) | 168 | 1.054 | | 1.025 | 1.083 | <0.001 |
| Age (years): |  |  | |  |  |  |
| 20 - 39 | 18 | - | | - | - | - |
| 40 - 59 | 74 | 2.963 | | 0.625 | 14.057 | 0.172 |
| ≥ 60 | 76 | 6.829 | | 1.468 | 31.780 | 0.014 |
| Gender (male) | 77 | 0.987 | | 0.520 | 1.873 | 0.967 |
| Resident regions: |  |  | |  |  |  |
| Urban | 68 | - | | - | - | - |
| Rural | 100 | 0.651 | | 0.341 | 1.243 | 0.193 |
| **Socioeconomic status** | | | | | | |
| Health insurance | 152 | 0.359 | | 0.126 | 1.021 | 0.055 |
| Health insurance rates: |  |  | |  |  |  |
| 100% | 30 | - | | - | - | - |
| 95% | 23 | 1.329 | | 0.438 | 4.030 | 0.616 |
| 80% | 94 | 0.696 | | 0.293 | 1.656 | 0.413 |
| 60% | 1 | 0.000 | | 0.000 | - | >0.999 |
| 40% | 4 | 0.000 | | 0.000 | - | 0.999 |
| Occupations: |  |  | |  |  |  |
| Elementary occupations | 46 | - | | - | - | - |
| Managers | 2 | 5815709512 | | 0.000 | - | 0.999 |
| Professionals | 3 | 1.800 | | 0.148 | 21.942 | 0.645 |
| Technicians and associate professionals | 3 | 0.000 | | 0.000 | - | 0.999 |
| Clerical support workers | 4 | 1.200 | | 0.112 | 12.826 | 0.880 |
| Service and sales workers | 15 | 0.900 | | 0.212 | 3.822 | 0.886 |
| Skilled agricultural, forestry and fishery workers | 36 | 2.571 | | 0.980 | 8.745 | 0.055 |
| Plant and machine operators, and assemblers | 6 | 0.720 | | 0.075 | 6.889 | 0.776 |
| Retired | 49 | 3.185 | | 1.298 | 7.814 | 0.011 |
| Student/unemployment | 1 | 5815709512 | | 0.000 | - | >0.999 |
| Other | 3 | 0.000 | | 0.000 | - | 0.999 |
| Highest education levels: |  |  | |  |  |  |
| School | 126 | - | | - | - | - |
| Vocational school | 12 | 1.429 | | 0.428 | 4.771 | 0.562 |
| Junior college | 8 | 0.667 | | 0.129 | 3.446 | 0.629 |
| Senior college | 15 | 1.000 | | 0.321 | 3.113 | >0.999 |
| College/University | 6 | 1.000 | | 0.176 | 5.682 | >0.999 |
| Postgraduate | 1 | 3230949732 | | 0.000 |  | >0.999 |
| Annual income: |  |  | |  |  |  |
| Upper-middle (US $4,036 - US $12,475) | 18 | - | | - | - | - |
| Lower-middle (US $1,026 - US $4,035) | 115 | 1.026 | | 0.358 | 2.943 | 0.961 |
| Low (≤ US $1,025) | 34 | 1.091 | | 0.327 | 3.645 | 0.888 |
| **Risk factors of aneurysmal subarachnoid hemorrhage** | | | | | | |
| Cigarette smoking | 63 | 0.932 | | 0.483 | 1.799 | 0.833 |
| Smoking behaviors: |  |  | |  |  |  |
| Quit | 18 | - | | - | - | - |
| Current | 45 | 1.103 | | 0.348 | 3.501 | 0.867 |
| Hypertension | 64 | 3.548 | | 1.816 | 6.932 | <0.001 |
| Genetic risk | 6 | 0.379 | | 0.043 | 3.320 | 0.381 |
| Alcohol consumption | 81 | 1.057 | | 0.558 | 2.002 | 0.866 |
| Alcohol drinking behaviors: |  |  | |  |  |  |
| Occasionally | 45 | - | | - | - | - |
| Sometimes | 25 | 0.474 | | 0.159 | 1.415 | 0.181 |
| Every day | 11 | 0.857 | | 0.219 | 3.358 | 0.825 |
| Sympathomimetic drugs | 2 | 0.000 | | 0.000 |  | 0.999 |
| Estrogen deficiency | 32 | 1.482 | | 0.603 | 3.645 | 0.391 |
| Antithrombotic therapy | 3 | 4.000 | | 0.355 | 45.082 | 0.262 |
| Elevated total cholesterol | 8 | 1.178 | | 0.271 | 5.114 | 0.827 |
| **Preexisting comorbidities** | | | | | | |
| Cerebrovascular disease | 3 | 0.973 | | 0.086 | 10.966 | 0.982 |
| Chronic cardiac failure | 3 | 4.000 | | 0.355 | 45.082 | 0.262 |
| Coronary artery disease/IM | 3 | 4.000 | | 0.355 | 45.082 | 0.262 |
| Active neoplasm | 3 | 0.973 | | 0.086 | 10.966 | 0.982 |
| Chronic renal failure | 2 | 1.964 | | 0.121 | 31.994 | 0.635 |
| Diabetes mellitus | 15 | 1.802 | | 0.619 | 5.251 | 0.280 |
| Hematological disease | 2 | 1.964 | | 0.121 | 31.994 | 0.635 |
| Others | 17 | 1.850 | | 0.673 | 5.088 | 0.233 |
| **Onset symptoms** | | | | | | |
| Sudden-onset, severe headache | 147 | 0.331 | | 0.130 | 0.841 | 0.020 |
| Vomiting | 102 | 0.672 | | 0.351 | 1.286 | 0.230 |
| Neck pain or stiffness | 66 | 1.070 | | 0.557 | 20.54 | 0.839 |
| Photophobia | 6 | 0.379 | | 0.043 | 3.320 | 0.381 |
| Blurred or double vision | 4 | 0.643 | | 0.065 | 6.323 | 0.705 |
| Brief loss of consciousness | 70 | 4.937 | | 2.484 | 9.814 | <0.001 |
| Seizures | 9 | 0.972 | | 0.234 | 4.039 | 0.969 |
| Other | 132 | 2.066 | | 0.873 | 4.890 | 0.099 |
| **Clinical presentation on admission** | | | | | | |
| GCS score | 168 | 0.682 | | 0.607 | 0.765 | <0.001 |
| GCS score: |  |  | |  |  |  |
| Mild (13 - 15) | 100 | - | | - | - | - |
| Moderate (9 - 12) | 29 | 5.437 | | 2.134 | 13.858 | <0.001 |
| Severe (3 - 8) | 39 | 25.933 | | 9.816 | 68.510 | <0.001 |
| Heart rate (beats/min) | 167 | 1.030 | | 1.009 | 1.051 | 0.005 |
| Heart rate (beats/min): |  |  | |  |  |  |
| ≤ 95 | 126 | - | | - | - | - |
| > 95 | 41 | 2.625 | | 1.272 | 5.415 | 0.009 |
| Respiratory rate (breaths/min) | 159 | 1.076 | | 0.989 | 1.171 | 0.090 |
| Respiratory rate (breaths/min): |  |  | |  |  |  |
| 12 - 25 | 146 | - | | - | - | - |
| > 25 | 13 | 3.962 | | 1.225 | 12.809 | 0.021 |
| Systolic blood pressure (mmHg) | 167 | 1.009 | | 0.997 | 1.020 | 0.145 |
| Systolic blood pressure (mmHg): |  |  | |  |  |  |
| < 140 | 83 | - | | - | - | - |
| ≥ 140 | 84 | 1.774 | | 0.927 | 3.394 | 0.083 |
| Diastolic blood pressure (mmHg) | 167 | 1.027 | | 1.002 | 1.052 | 0.035 |
| Diastolic blood pressure (mmHg): |  |  | |  |  |  |
| < 90 | 117 | - | | - | - | - |
| ≥ 90 | 50 | 3.005 | | 1.507 | 5.993 | 0.002 |
| Body temperature (^o^C) | 167 | 1.792 | | 0.858 | 3.743 | 0.120 |
| Body temperature (^o^C): |  |  | |  |  |  |
| < 38 | 160 | - | | - | - | - |
| ≥ 38 | 7 | 5.192 | | 0.975 | 27.661 | 0.054 |
| Focal neurological deficits | 99 | 1.639 | | 0.843 | 3.190 | 0.146 |
| Focal neurologic signs: |  |  | |  |  |  |
| Third nerve palsy | 4 | 0.523 | | 0.052 | 5.214 | 0.580 |
| Sixth nerve palsy | 1 | 0.000 | | 0.000 |  | >0.999 |
| Hemiparesis | 1 | 1.289 | | 0.549 | 3.027 | 0.559 |
| Aphasia | 6 | 1.6547 | | 0.317 | 8.667 | 0.550 |
| Bilateral leg weakness | 1 | 0.000 | | 0.000 |  | >0.999 |
| Impaired level of consciousness | 49 | 5.333 | | 2.180 | 13.047 | <0.001 |
| Brainstem signs | 3 | 3.333 | | 0.292 | 38.082 | 0.333 |
| Neck stiffness | 52 | 0.505 | | 0.222 | 1.149 | 0.103 |
| **Head imaging findings on admission** | | | | | | |
| Non-contrast head computed tomography (CT) findings | | | | | | |
| Detection of blood within the subarachnoid space | 167 | 937107053.7 | | 0.000 |  | >0.999 |
| Location of blood within the subarachnoid space: |  |  | |  |  |  |
| Basal cistern | 79 | 2.952 | | 1.503 | 5.798 | 0.002 |
| Sylvian fissure | 157 | 1.223 | | 0.304 | 4.921 | 0.777 |
| Interhemispheric fissure | 84 | 1.660 | | 0.866 | 3.183 | 0.127 |
| Interpeduncular fossa | 83 | 3.441 | | 1.734 | 6.830 | <0.001 |
| Suprasellar cistern | 90 | 1.490 | | 0.775 | 2.865 | 0.232 |
| Ambient cistern | 81 | 3.289 | | 1.668 | 6.487 | 0.001 |
| Quadrigeminal cistern | 27 | 4.359 | | 1.837 | 10.345 | 0.001 |
| Maximum thickness of subarachnoid blood (mm) | 165 | 1.074 | | 1.001 | 1.153 | 0.048 |
| IVH | 107 | 3.428 | | 1.608 | 7.035 | 0.001 |
| Location of blood within the ventricular system: |  |  | |  |  |  |
| Right lateral ventricle | 95 | 3.882 | | 0.796 | 18.931 | 0.093 |
| Left lateral ventricle | 99 | 1402110925 | | 0.000 |  | 0.999 |
| Third ventricle | 42 | 9.206 | | 3.748 | 22.614 | <0.001 |
| Fourth ventricle | 43 | 2.364 | | 1.064 | 5.208 | 0.035 |
| Graeb score | 102 | 1.634 | | 1.303 | 2.050 | <0.001 |
| Graeb score: |  |  | |  |  |  |
| Mild (1 - 4) | 69 | - | | - | - | - |
| Moderate (5 - 8) | 24 | 15.294 | | 4.583 | 51.038 | <0.001 |
| Severe (9 - 12) | 9 | 24.471 | | 2.851 | 210.027 | 0.004 |
| ICH | 41 | 2.023 | | 0.983 | 4.163 | 0.056 |
| ICH volume (mL) | 41 | 1.023 | | 0.998 | 1.049 | 0.071 |
| ICH volume (mL): |  |  | |  |  |  |
| < 30 | 26 | - | | - | - | - |
| 30 - 60 | 7 | 1.200 | | 0.221 | 6.521 | 0.833 |
| > 60 | 8 | 4.800 | | 0.806 | 28.598 | 0.085 |
| Subdural hemorrhage | 10 | 2.038 | | 0.565 | 7.355 | 0.277 |
| Hydrocephalus | 72 | 2.840 | | 1.470 | 5.487 | 0.002 |
| Evans' index | 145 | 2379.810 | | 8.353 | 67986.949 | 0.007 |
| Bicaudate index | 145 | 12295.426 | | 42.796 | 3532530.879 | 0.001 |
| Relative bicaudate index | 145 | 5.164 | | 1.788 | 14.910 | 0.002 |
| Hypodense lesions on computed tomography | 8 | 3.000 | | 0.312 | 28.841 | 0.341 |
| Patterns of infarction: | 14 | 4.060 | | 1.291 | 12.769 | 0.017 |
| Single cortical infarcts | 6 | 0.333 | | 0.035 | 3.205 | 0.341 |
| Multiple widespread infarcts | 8 | 3.000 | | 0.312 | 28.841 | 0.341 |
| Multislice computed tomography (MSCT) angiography / Digital subtraction angiography (DSA) findings | | | | | | |
| Number of aneurysm | 168 | 0.799 | | 0.367 | 1.740 | 0.573 |
| Number of aneurysm: |  |  | |  |  |  |
| Single aneurysm | 144 | - | | - | - | - |
| Multiple aneurysms | 24 | 0.608 | | 0.227 | 1.628 | 0.322 |
| Side of aneurysm: |  |  | |  |  |  |
| Right | 58 | - | | - | - | - |
| Left | 49 | 0.705 | | 0.311 | 1.599 | 0.402 |
| Both | 60 | 1.020 | | 0.482 | 2.159 | 0.959 |
| Groups of aneurysm site: |  |  | |  |  |  |
| Anterior circulation aneurysm | 120 | 1.256 | | 0.528 | 2.988 | 0.606 |
| Posterior circulation aneurysm | 33 | 0.670 | | 0.285 | 1.573 | 0.357 |
| Aneurysm site: |  |  | |  |  |  |
| Internal carotid artery (ICA) | 35 | 0.867 | | 0.390 | 1.926 | 0.726 |
| Ophtalmic segment of the ICA (OphIC) | 1 | 0.000 | | 0.000 | 0.000 | >0.999 |
| Cavernous segment of the ICA (cIC) | 7 | 0.771 | | 0.145 | 4.103 | 0.760 |
| Posterior communicating artery (PCoA) | 25 | 1.362 | | 0.569 | 3.260 | 0.488 |
| Anterior cerebral artery (ACA) | 16 | 0.623 | | 0.191 | 2.026 | 0.431 |
| Anterior communicating artery (AcoA) | 49 | 1.350 | | 0.676 | 2.697 | 0.395 |
| Middle cerebral artery (MCA) | 34 | 0.915 | | 0.410 | 2.041 | 0.828 |
| Vertebral artery (VA) | 7 | 1.486 | | 0.321 | 6.879 | 0.612 |
| Posterior inferior cerebellar artery (PICA) | 4 | 0.643 | | 0.065 | 6.232 | 0.705 |
| Basilar artery (BA) | 7 | 0.000 | | 0.000 |  | 0.999 |
| Other | 129 | 1.975 | | 0.865 | 4.512 | 0.106 |
| Maximum aneurysm size (mm): |  |  | |  |  |  |
| Maximum dome width | 166 | 1.008 | | 0.895 | 1.135 | 0.895 |
| Dome height | 163 | 1.073 | | 0.965 | 1.194 | 0.195 |
| Maximum neck width | 158 | 1.259 | | 0.997 | 1.591 | 0.053 |
| Dome-to-neck ratio | 157 | 1.447 | | 0.903 | 2.317 | 0.124 |
| Shape of aneurysm: |  |  | |  |  |  |
| Regular | 18 |  | |  |  |  |
| Irregular | 117 | 2.350 | | 0.729 | 7.580 | 0.153 |
| Multilobular | 11 | 2.000 | | 0.382 | 10.482 | 0.412 |
| Two domes |  |  | |  |  |  |
| Other | 3 | 1.750 | | 0.124 | 24.650 | 0.678 |
| Vasospasm of the parent artery | 14 | 2.803 | | 0.922 | 8.520 | 0.069 |
| **Severity of aneurysmal subarachnoid hemorrhage on admission** | | | | | | |
| PAASH score | 168 | 3.191 | | 2.240 | 4.546 | <0.001 |
| PAASH scale: |  |  | |  |  |  |
| Grade I | 80 | - | | - | - | - |
| Grade II | 31 | 2.301 | | 0.773 | 6.849 | 0.134 |
| Grade III | 28 | 14.200 | | 5.027 | 40.115 | <0.001 |
| Grade IV | 25 | 31.556 | | 9.499 | 104.825 | <0.001 |
| Grade V | 4 | 23.667 | | 2.219 | 252.423 | 0.009 |
| WFNS score | 168 | 2.331 | | 1.796 | 3.026 | <0.001 |
| WFNS scale: |  |  | |  |  |  |
| Grade I | 80 | - | | - | - | - |
| Grade II | 14 | 2.152 | | 0.503 | 9.198 | 0.301 |
| Grade III | 6 | 1.578 | | 0.165 | 15.063 | 0.692 |
| Grade IV | 50 | 10.894 | | 4.463 | 26.592 | <0.001 |
| Grade V | 18 | 37.444 | | 9.531 | 163.251 | <0.001 |
| Modified WFNS score | 168 | 2.373 | | 1.824 | 3.089 | <0.001 |
| Modified WFNS scale: |  |  | |  |  |  |
| Grade I | 80 | - | | - | - | - |
| Grade II | 14 | 1.315 | | 0.253 | 6.845 | 0.745 |
| Grade III | 6 | 3.944 | | 0.630 | 24.677 | 0.142 |
| Grade IV | 50 | 10.894 | | 4.463 | 26.592 | <0.001 |
| Grade V | 18 | 39.444 | | 9.531 | 163.251 | <0.001 |
| Hunt and Hess score | 168 | 2.538 | | 1.904 | 3.385 | <0.001 |
| Hunt and Hess scale: |  |  | |  |  |  |
| Grade 1 | 39 | - | | - | - | - |
| Grade 2 | 45 | 0.850 | | 0.227 | 3.186 | 0.809 |
| Grade 3 | 25 | 2.644 | | 0.734 | 9.530 | 0.137 |
| Grade 4 | 21 | 5.100 | | 1.424 | 18.270 | 0.012 |
| Grade 5 | 38 | 30.114 | | 8.657 | 104.751 | <0.001 |
| Fisher score | 168 | 3.686 | | 1.689 | 8.046 | 0.001 |
| Fisher scale: |  |  | |  |  |  |
| Group 2 | 13 | - | | - | - | - |
| Group 1 | 1 | 0.000 | | 0.000 | - | >0.999 |
| Group 3 | 34 | 2.069 | | 0.218 | 19.629 | 0.527 |
| Group 4 | 120 | 8.870 | | 1.117 | 70.420 | 0.039 |
| Claassen score | 168 | 1.957 | | 1.301 | 2.944 | 0.001 |
| Claassen scale: |  |  | |  |  |  |
| Grade 1 |  |  | |  |  |  |
| Grade 2 | 22 | - | | - | - | - |
| Grade 3 | 11 | 0.450 | | 0.044 | 4.596 | 0.501 |
| Grade 4 | 39 | 0.984 | | 0.253 | 3.825 | 0.982 |
| Grade 5 | 96 | 3.971 | | 1.261 | 12.605 | 0.019 |
| **Laboratory investigations on admission** | | | | | | |
| Complete blood count |  |  | |  |  |  |
| Red blood cells (T/L) | 163 | 1.021 | | 0.673 | 1.550 | 0.922 |
| Hemoglobin (g/L) | 163 | 1.004 | | 0.988 | 1.020 | 0.622 |
| Hematocrit (L/L) | 163 | 590.850 | | 0.288 | 1214.239 | 0.101 |
| Platelets (G/L) | 163 | 1.000 | | 0.996 | 1.004 | 0.913 |
| White blood cells (G/L) | 163 | 1.114 | | 1.034 | 1.200 | 0.004 |
| Percentage of neutrophils (%) | 162 | 1.015 | | 1.001 | 1.029 | 0.036 |
| Coagulation |  |  | |  |  |  |
| Prothrombin time (PT) | 155 | 1.011 | | 0.998 | 1.024 | 0.090 |
| Prothrombin time with INR (PT-INR) | 155 | 6.618 | | 0.162 | 270.705 | 0.318 |
| Activated partial thromboplastin time (APTT) | 153 | 1.002 | | 0.991 | 1.014 | 0.666 |
| rAPTT | 152 | 0.969 | | 0.855 | 1.100 | 0.629 |
| Blood biochemical investigation |  |  | |  |  |  |
| Ure (mmol/L) | 166 | 1.241 | | 1.036 | 1.487 | 0.019 |
| Glucose (mmol/L) | 156 | 1.617 | | 1.339 | 1.953 | <0.001 |
| Glucose (mmol/L): |  |  | |  |  |  |
| ≤ 6.4 | 29 | - | | - | - | - |
| > 6.4 | 127 | 9.360 | | 2.132 | 41.086 | 0.003 |
| Creatinine (µmol/L) | 167 | 1.020 | | 1.005 | 1.036 | 0.011 |
| SGOT (UI/L) | 157 | 1.050 | | 1.024 | 1.076 | <0.001 |
| SGPT (UI/L) | 165 | 1.024 | | 1.006 | 1.043 | <0.001 |
| Na^+^ (mmol/L) | 167 | 1.070 | | 0.999 | 1.147 | 0.053 |
| Na^+^ (mmol/L): |  |  | |  |  |  |
| ≥ 135 | 128 | - | | - | - | - |
| < 135 | 39 | 0.500 | | 0.219 | 1.143 | 0.100 |
| K^+^ (mmol/L) | 167 | 0.747 | | 0.370 | 1.509 | 0.417 |
| Cl^-^ (mmol/L) | 167 | 0.982 | | 0.945 | 1.020 | 0.345 |
| **Aneurysm repairs and other treatments** | | | | | | |
| No aneurysm repair | 26 | 16.814 | | 5.423 | 52.137 | <0.001 |
| Endovascular coiling | 79 | 0.297 | | 0.149 | 0.592 | 0.001 |
| Number of coils | 59 | 0.825 | | 0.612 | 1.113 | 0.207 |
| Stent-assisted coiling | 2 | 0.000 | | 0.000 |  | 0.999 |
| Balloon-assisted coiling | 9 | 0.000 | | 0.000 |  | 0.999 |
| Surgical clipping | 63 | 0.761 | | 0.390 | 1.487 | 0.425 |
| Number of clip attempts | 57 | 1.345 | | 0.942 | 1.919 | 0.103 |
| Temporary vessel occlusion of the parent artery | 7 | 1.550 | | 0.252 | 9.516 | 0.636 |
| Surgical hematoma evacuation or decompressive craniotomy | 7 | 12.941 | | 1.518 | 110.310 | 0.019 |
| External ventricular drainage | 26 | 4.769 | | 1.964 | 11.580 | 0.001 |
| Number of EVD | 26 | 969285189.9 | | 0.000 |  | 0.999 |
| Intraventricular fibrinolysis | 3 | 4.000 | | 0.355 | 45.082 | 0.262 |
| **Airway management and mechanical ventilation** | | | | | | |
| Tracheal intubation | 144 | | 1058412922 | 0.000 |  | 0.999 |
| Mechanical ventilation | 141 | | 1096213712 | 0.000 |  | 0.999 |
| Tracheotomy | 15 | | 15.570 | 3.369 | 71.950 | <0.001 |
| **Medical treatment** | | | | | | |
| Nimodipine for preventing and treating cerebral vasospasm | 158 | 0.238 | | 0.057 | 0.991 | 0.049 |
| Route of administration of nimodipine: |  |  | |  |  |  |
| Oral | 113 | 1.993 | | 0.895 | 4.438 | 0.091 |
| Intravenous | 60 | 0.574 | | 0.281 | 1.173 | 0.128 |
| Deep venous thrombosis prophylaxis | 16 | 3.690 | | 1.259 | 10.821 | 0.017 |
| **Complications** | | | | | | |
| Rebleeding | 10 | 21.800 | | 2.683 | 177.129 | 0.004 |
| Number of rebleeding sites: |  |  | |  |  |  |
| Rebleeding from a single site | 4 | - | | - | - | **-** |
| Rebleeding from multiple sites | 6 | 538491621.5 | | 0.000 |  | 0.999 |
| Rebleeding sites: |  |  | |  |  |  |
| Subarachnoid hemorrhage, (n=9) | 3 | 323094972.9 | | 0.000 |  | 0.999 |
| Subdural hemorrhage | 4 | 0.000 | | 0.000 |  | 0.999 |
| Intraventricular hemorrhage | 6 | 538491621.5 | | 0.000 |  | 0.999 |
| Intracerebral hemorrhage | 7 | 807737432.2 | | 0.000 |  | 0.999 |
| Vasospasm and delayed cerebral ischemia | 17 | 5.749 | | 1.909 | 17.310 | 0.002 |
| Patterns of vasospasm and delayed cerebral ischemia: |  |  | |  |  |  |
| Single cortical infarcts | 9 | - | | - | - | - |
| Multiple widespread infarcts | 7 | 1.250 | | 0.146 | 10.699 | 0.839 |
| Acute hydrocephalus | 76 | 3.415 | | 1.749 | 6.669 | <0.001 |
| Hyponatremia | 34 | 1.729 | | 0.801 | 3.732 | 0.163 |
| Seizures | 44 | 0.565 | | 0.261 | 1.225 | 0.148 |
| Chronic hydrocephalus | 5 | 4.061 | | 0.608 | 27.133 | 0.148 |
| EVD obstruction | 2 | 1076983243 | | 0.000 |  | 0.999 |
| EVD replacement | 2 | 881168107.9 | | 0.000 |  | 0.999 |
| Ventriculitis | 8 | 1.810 | | 0.430 | 7.609 | 0.418 |
| Pneumonia | 29 | 5.050 | | 2.155 | 11.836 | <0.001 |
| Urinary tract infection | 3 | 0.000 | | 0.000 |  | 0.999 |
| **Clinical time course** | | | | | | |
| Ictus to hospital arrival (hours): |  |  | |  |  |  |
| ≤ 24 hours | 69 | - | | - | - | - |
| >24–72 hours | 91 | 0.464 | | 0.240 | 0.899 | 0.023 |
| >72 hours | 1 | 0.000 | | 0.000 |  | >0.999 |
| EVD duration (days) | 25 | 1.065 | | 0.875 | 1.296 | 0.527 |
| EVD duration (days): |  |  | |  |  |  |
| ≤ 7 | 17 | - | | - | - | - |
| > 7 | 8 | 0.909 | | 0.159 | 5.195 | 0.915 |
| Length of hospitalization (days) | 168 | 0.969 | | 0.932 | 1.007 | 0.113 |

**S2 Table**. Factors associated with poor outcome of patients with aneurysmal subarachnoid hemorrhage at 90 days after the onset of hemorrhage: multivariate logistic regression analyses (backward elimination)

| Steps | Factors | Unit | OR | 95% CI for OR | | p value |
| --- | --- | --- | --- | --- | --- | --- |
|  |  |  |  | Lower | Upper |  |
| 1 | **Demographics** | | | | | |
|  | Age (years): |  |  |  |  |  |
|  | 20 - 39 | % | - | - | - | 0.060 |
|  | 40 - 59 | % | 41.866 | 0.579 | 3028.279 | 0.087 |
|  | ≥ 60 | % | 119.151 | 1.747 | 8125.322 | 0.026 |
|  | **Risk factors of aneurysmal subarachnoid hemorrhage** | | | | | |
|  | Hypertension | % | 3.355 | 0.663 | 16.971 | 0.143 |
|  | **Head imaging findings on admission** | | | | | |
|  | Location of blood within the subarachnoid space: |  |  |  |  |  |
|  | Basal cistern | % | 5.001 | 0.904 | 27.675 | 0.065 |
|  | Interpeduncular fossa | % | 0.557 | 0.067 | 4.616 | 0.588 |
|  | Ambient cistern | % | 2.265 | 0.334 | 15.378 | 0.403 |
|  | Quadrigeminal cistern | % | 0.603 | 0.077 | 4.726 | 0.630 |
|  | IVH | % | 0.882 | 0.181 | 4.301 | 0.877 |
|  | ICH | % | 1.606 | 0.264 | 9.765 | 0.607 |
|  | **Severity of aneurysmal subarachnoid hemorrhage on admission** | | | | | |
|  | WFNS scale: |  |  |  |  |  |
|  | Grade I | % | - | - | - | 0.018 |
|  | Grade II | % | 2.733 | 0.198 | 37.706 | 0.453 |
|  | Grade III | % | 1.199 | 0.023 | 63.532 | 0.929 |
|  | Grade IV | % | 14.241 | 2.299 | 88.206 | 0.004 |
|  | Grade V | % | 361.682 | 8.172 | 16008.124 | 0.002 |
|  | **Aneurysm repairs and other treatments** | | | | | |
|  | Aneurysm repairs: |  |  |  |  |  |
|  | No aneurysm repair | % | - | - | - | 0.002 |
|  | Endovascular coiling | % | 0.019 | 0.002 | 0.183 | 0.001 |
|  | Surgical clipping | % | 0.034 | 0.004 | 0.320 | 0.003 |
|  | External ventricular drainage | % | 7.100 | 0.951 | 53.010 | 0.056 |
|  | **Medical treatment** | | | | | |
|  | Nimodipine for preventing and treating cerebral vasospasm | % | 4.343 | 0.116 | 162.147 | 0.427 |
|  | **Complications** | | | | | |
|  | Rebleeding | % | 123.319 | 5.155 | 2950.205 | 0.003 |
|  | Vasospasm and delayed cerebral ischemia | % | 16.827 | 2.243 | 126.236 | 0.006 |
|  | Acute hydrocephalus | % | 0.596 | 0.113 | 3.141 | 0.542 |
|  | Pneumonia | % | 2.684 | 0.541 | 13.321 | 0.227 |
|  | Constant | % | 0.001 |  |  | 0.007 |
| 2 | **Demographics** | | | | | |
|  | Age (years): |  |  |  |  |  |
|  | 20 - 39 | % | - | - | - | 0.061 |
|  | 40 - 59 | % | 38.884 | 0.614 | 2462.199 | 0.084 |
|  | ≥ 60 | % | 113.458 | 1.775 | 7252.294 | 0.026 |
|  | **Risk factors of aneurysmal subarachnoid hemorrhage** | | | | | |
|  | Hypertension | % | 3.264 | 0.669 | 15.927 | 0.143 |
|  | **Head imaging findings on admission** | | | | | |
|  | Location of blood within the subarachnoid space: |  |  |  |  |  |
|  | Basal cistern | % | 4.979 | 0.904 | 27.422 | 0.065 |
|  | Interpeduncular fossa | % | 0.566 | 0.069 | 4.620 | 0.595 |
|  | Ambient cistern | % | 2.251 | 0.332 | 15.262 | 0.406 |
|  | Quadrigeminal cistern | % | 0.608 | 0.078 | 4.738 | 0.635 |
|  | ICH | % | 1.605 | 0.266 | 9.698 | 0.606 |
|  | **Severity of aneurysmal subarachnoid hemorrhage on admission** | | | | | |
|  | WFNS scale: |  |  |  |  |  |
|  | Grade I | % | - | - | - | 0.015 |
|  | Grade II | % | 2.707 | 0.195 | 37.571 | 0.458 |
|  | Grade III | % | 1.249 | 0.026 | 58.923 | 0.910 |
|  | Grade IV | % | 13.682 | 2.382 | 78.599 | 0.003 |
|  | Grade V | % | 353.220 | 8.084 | 15434.064 | 0.002 |
|  | **Aneurysm repairs and other treatments** | | | | | |
|  | Aneurysm repairs: |  |  |  |  |  |
|  | No aneurysm repair | % | - | - | - | 0.002 |
|  | Endovascular coiling | % | 0.020 | 0.002 | 0.184 | 0.001 |
|  | Surgical clipping | % | 0.035 | 0.004 | 0.318 | 0.003 |
|  | External ventricular drainage | % | 7.166 | 0.961 | 53.422 | 0.055 |
|  | **Medical treatment** | | | | | |
|  | Nimodipine for preventing and treating cerebral vasospasm | % | 4.299 | 0.114 | 162.617 | 0.431 |
|  | **Complications** | | | | | |
|  | Rebleeding | % | 116.403 | 5.393 | 2512.272 | 0.002 |
|  | Vasospasm and delayed cerebral ischemia | % | 16.984 | 2.278 | 126.625 | 0.006 |
|  | Acute hydrocephalus | % | 0.591 | 0.112 | 3.113 | 0.535 |
|  | Pneumonia | % | 2.686 | 0.538 | 13.407 | 0.228 |
|  | Constant | % | 0.001 |  |  | 0.007 |
| 3 | **Demographics** | | | | | |
|  | Age (years): |  |  |  |  |  |
|  | 20 - 39 | % | - | - | - | 0.066 |
|  | 40 - 59 | % | 38.384 | 0.574 | 2565.358 | 0.089 |
|  | ≥ 60 | % | 108.749 | 1.621 | 7294.582 | 0.029 |
|  | **Risk factors of aneurysmal subarachnoid hemorrhage** | | | | | |
|  | Hypertension | % | 3.199 | 0.663 | 15.447 | 0.148 |
|  | **Head imaging findings on admission** | | | | | |
|  | Location of blood within the subarachnoid space: |  |  |  |  |  |
|  | Basal cistern | % | 4.572 | 0.860 | 24.306 | 0.075 |
|  | Interpeduncular fossa | % | 0.521 | 0.065 | 4.163 | 0.538 |
|  | Ambient cistern | % | 2.031 | 0.313 | 13.195 | 0.458 |
|  | ICH | % | 1.580 | 0.273 | 9.154 | 0.610 |
|  | **Severity of aneurysmal subarachnoid hemorrhage on admission** | | | | | |
|  | WFNS scale: |  |  |  |  |  |
|  | Grade I | % | - | - | - | 0.015 |
|  | Grade II | % | 2.823 | 0.201 | 39.693 | 0.441 |
|  | Grade III | % | 1.325 | 0.028 | 61.734 | 0.886 |
|  | Grade IV | % | 13.348 | 2.338 | 76.221 | 0.004 |
|  | Grade V | % | 283.518 | 7.681 | 10465.216 | 0.002 |
|  | **Aneurysm repairs and other treatments** | | | | | |
|  | Aneurysm repairs: |  |  |  |  |  |
|  | No aneurysm repair | % | - | - | - | 0.002 |
|  | Endovascular coiling | % | 0.019 | 0.002 | 0.174 | <0.001 |
|  | Surgical clipping | % | 0.037 | 0.004 | 0.325 | 0.003 |
|  | External ventricular drainage | % | 6.931 | 0.960 | 50.068 | 0.055 |
|  | **Medical treatment** | | | | | |
|  | Nimodipine for preventing and treating cerebral vasospasm | % | 3.680 | 0.100 | 135.901 | 0.479 |
|  | **Complications** | | | | | |
|  | Rebleeding | % | 122.450 | 5.661 | 2648.828 | 0.002 |
|  | Vasospasm and delayed cerebral ischemia | % | 16.301 | 2.243 | 118.487 | 0.006 |
|  | Acute hydrocephalus | % | 0.582 | 0.110 | 3.079 | 0.525 |
|  | Pneumonia | % | 2.675 | 0.535 | 13.375 | 0.231 |
|  | Constant | % | 0.001 |  |  | 0.007 |
| 4 | **Demographics** | | | | | |
|  | Age (years): |  |  |  |  |  |
|  | 20 - 39 | % | - | - | - | 0.066 |
|  | 40 - 59 | % | 44.690 | 0.712 | 2806.815 | 0.072 |
|  | ≥ 60 | % | 114.356 | 1.773 | 7377.421 | 0.026 |
|  | **Risk factors of aneurysmal subarachnoid hemorrhage** | | | | | |
|  | Hypertension | % | 3.382 | 0.704 | 16.252 | 0.128 |
|  | **Head imaging findings on admission** | | | | | |
|  | Location of blood within the subarachnoid space: |  |  |  |  |  |
|  | Basal cistern | % | 5.023 | 0.959 | 26.314 | 0.056 |
|  | Interpeduncular fossa | % | 0.443 | 0.061 | 3.225 | 0.422 |
|  | Ambient cistern | % | 1.988 | 0.313 | 12.642 | 0.467 |
|  | **Severity of aneurysmal subarachnoid hemorrhage on admission** | | | | | |
|  | WFNS scale: |  |  |  |  |  |
|  | Grade I | % | - | - | - | 0.010 |
|  | Grade II | % | 3.182 | 0.240 | 42.094 | 0.380 |
|  | Grade III | % | 1.602 | 0.042 | 61.595 | 0.800 |
|  | Grade IV | % | 14.631 | 2.576 | 83.108 | 0.002 |
|  | Grade V | % | 355.984 | 10.390 | 12196.780 | 0.001 |
|  | **Aneurysm repairs and other treatments** | | | | | |
|  | Aneurysm repairs: |  |  |  |  |  |
|  | No aneurysm repair | % | - | - | - | 0.001 |
|  | Endovascular coiling | % | 0.017 | 0.002 | 0.157 | <0.001 |
|  | Surgical clipping | % | 0.040 | 0.005 | 0.336 | 0.003 |
|  | External ventricular drainage | % | 6.268 | 0.919 | 42.745 | 0.061 |
|  | **Medical treatment** | | | | | |
|  | Nimodipine for preventing and treating cerebral vasospasm | % | 4.012 | 0.115 | 140.550 | 0.444 |
|  | **Complications** | | | | | |
|  | Rebleeding | % | 137.908 | 6.451 | 2948.003 | 0.002 |
|  | Vasospasm and delayed cerebral ischemia | % | 15.816 | 2.183 | 114.572 | 0.006 |
|  | Acute hydrocephalus | % | 0.549 | 0.109 | 2.977 | 0.504 |
|  | Pneumonia | % | 2.649 | 0.537 | 13.082 | 0.232 |
|  | Constant | % | 0.001 |  |  | 0.006 |
| 5 | **Demographics** | | | | | |
|  | Age (years): |  |  |  |  |  |
|  | 20 - 39 | % | - | - | - | 0.070 |
|  | 40 - 59 | % | 35.590 | 0.702 | 1804.963 | 0.075 |
|  | ≥ 60 | % | 84.710 | 1.667 | 4305.548 | 0.027 |
|  | **Risk factors of aneurysmal subarachnoid hemorrhage** | | | | | |
|  | Hypertension | % | 3.429 | 0.720 | 16.343 | 0.122 |
|  | **Head imaging findings on admission** | | | | | |
|  | Location of blood within the subarachnoid space: |  |  |  |  |  |
|  | Basal cistern | % | 4.239 | 0.905 | 19.850 | 0.067 |
|  | Interpeduncular fossa | % | 0.450 | 0.064 | 3.182 | 0.424 |
|  | Ambient cistern | % | 1.894 | 0.309 | 11.592 | 0.490 |
|  | **Severity of aneurysmal subarachnoid hemorrhage on admission** | | | | | |
|  | WFNS scale: |  |  |  |  |  |
|  | Grade I | % | - | - | - | 0.007 |
|  | Grade II | % | 2.924 | 0.235 | 36.330 | 0.404 |
|  | Grade III | % | 1.694 | 0.043 | 67.039 | 0.779 |
|  | Grade IV | % | 12.635 | 2.404 | 66.413 | 0.003 |
|  | Grade V | % | 272.281 | 10.136 | 7314.146 | 0.001 |
|  | **Aneurysm repairs and other treatments** | | | | | |
|  | Aneurysm repairs: |  |  |  |  |  |
|  | No aneurysm repair | % | - | - | - | 0.001 |
|  | Endovascular coiling | % | 0.019 | 0.002 | 0.164 | <0.001 |
|  | Surgical clipping | % | 0.040 | 0.005 | 0.337 | 0.003 |
|  | External ventricular drainage | % | 4.742 | 0.863 | 26.047 | 0.073 |
|  | **Medical treatment** | | | | | |
|  | Nimodipine for preventing and treating cerebral vasospasm | % | 3.404 | 0.110 | 104.921 | 0.484 |
|  | **Complications** | | | | | |
|  | Rebleeding | % | 95.480 | 5.820 | 1566.418 | 0.001 |
|  | Vasospasm and delayed cerebral ischemia | % | 16.774 | 2.365 | 118.961 | 0.005 |
|  | Pneumonia | % | 2.609 | 0.542 | 12.566 | 0.232 |
|  | Constant | % | 0.001 |  |  | 0.006 |
| 6 | **Demographics** | | | | | |
|  | Age (years): |  |  |  |  |  |
|  | 20 - 39 | % | - | - | - | 0.068 |
|  | 40 - 59 | % | 19.848 | 0.698 | 564.509 | 0.080 |
|  | ≥ 60 | % | 48.541 | 1.655 | 1423.514 | 0.024 |
|  | **Risk factors of aneurysmal subarachnoid hemorrhage** | | | | | |
|  | Hypertension | % | 3.729 | 0.799 | 17.401 | 0.094 |
|  | **Head imaging findings on admission** | | | | | |
|  | Location of blood within the subarachnoid space: |  |  |  |  |  |
|  | Basal cistern | % | 4.160 | 0.919 | 18.823 | 0.064 |
|  | Interpeduncular fossa | % | 0.695 | 0.157 | 3.086 | 0.632 |
|  | **Severity of aneurysmal subarachnoid hemorrhage on admission** | | | | | |
|  | WFNS scale: |  |  |  |  |  |
|  | Grade I | % | - | - | - | 0.007 |
|  | Grade II | % | 3.158 | 0.245 | 40.643 |  |
|  | Grade III | % | 1.714 | 0.046 | 64.089 | 0.771 |
|  | Grade IV | % | 12.258 | 2.369 | 63.433 | 0.003 |
|  | Grade V | % | 273.966 | 10.616 | 7070.157 | 0.001 |
|  | **Aneurysm repairs and other treatments** | | | | | |
|  | Aneurysm repairs: |  |  |  |  |  |
|  | No aneurysm repair | % | - | - | - | 0.001 |
|  | Endovascular coiling | % | 0.021 | 0.003 | 0.174 | <0.001 |
|  | Surgical clipping | % | 0.043 | 0.005 | 0.348 | 0.003 |
|  | External ventricular drainage | % | 4.471 | 0.831 | 24.047 | 0.081 |
|  | **Medical treatment** | | | | | |
|  | Nimodipine for preventing and treating cerebral vasospasm | % | 4.074 | 0.115 | 143.960 | 0.440 |
|  | **Complications** | | | | | |
|  | Rebleeding | % | 80.008 | 5.335 | 1199.884 | 0.002 |
|  | Vasospasm and delayed cerebral ischemia | % | 14.353 | 2.216 | 92.975 | 0.005 |
|  | Pneumonia | % | 2.822 | 0.594 | 13.415 | 0.192 |
|  | Constant | % | 0.001 |  |  | 0.008 |
| 7 | **Demographics** | | | | | |
|  | Age (years): |  |  |  |  |  |
|  | 20 - 39 | % | - | - | - | 0.064 |
|  | 40 - 59 | % | 16.251 | 0.699 | 377.606 | 0.082 |
|  | ≥ 60 | % | 42.892 | 1.682 | 1093.989 | 0.023 |
|  | **Risk factors of aneurysmal subarachnoid hemorrhage** | | | | | |
|  | Hypertension | % | 3.452 | 0.769 | 15.493 | 0.106 |
|  | **Head imaging findings on admission** | | | | | |
|  | Location of blood within the subarachnoid space: |  |  |  |  |  |
|  | Basal cistern | % | 3.518 | 0.924 | 13.385 | 0.065 |
|  | **Severity of aneurysmal subarachnoid hemorrhage on admission** | | | | | |
|  | WFNS scale: |  |  |  |  |  |
|  | Grade I | % | - | - | - | 0.006 |
|  | Grade II | % | 3.533 | 0.289 | 43.242 | 0.323 |
|  | Grade III | % | 1.961 | 0.057 | 67.514 | 0.709 |
|  | Grade IV | % | 11.904 | 2.296 | 61.729 | 0.003 |
|  | Grade V | % | 231.935 | 10.206 | 5270.722 | 0.001 |
|  | **Aneurysm repairs and other treatments** | | | | | |
|  | Aneurysm repairs: |  |  |  |  |  |
|  | No aneurysm repair | % | - | - | - | 0.002 |
|  | Endovascular coiling | % | 0.023 | 0.003 | 0.183 | <0.001 |
|  | Surgical clipping | % | 0.041 | 0.005 | 0.334 | 0.003 |
|  | External ventricular drainage | % | 4.573 | 0.866 | 24.154 | 0.073 |
|  | **Medical treatment** | | | | | |
|  | Nimodipine for preventing and treating cerebral vasospasm | % | 3.888 | 0.108 | 139.906 | 0.458 |
|  | **Complications** | | | | | |
|  | Rebleeding | % | 76.335 | 4.993 | 1167.079 | 0.002 |
|  | Vasospasm and delayed cerebral ischemia | % | 14.262 | 2.216 | 91.794 | 0.005 |
|  | Pneumonia | % | 2.752 | 0.588 | 12.879 | 0.199 |
|  | Constant | % | 0.002 |  |  | 0.008 |
| 8 | **Demographics** | | | | | |
|  | Age (years): |  |  |  |  |  |
|  | 20 - 39 | % | - | - | - | 0.050 |
|  | 40 - 59 | % | 21.081 | 0.921 | 482.471 | 0.056 |
|  | ≥ 60 | % | 53.153 | 2.081 | 1357.480 | 0.016 |
|  | **Risk factors of aneurysmal subarachnoid hemorrhage** | | | | | |
|  | Hypertension | % | 3.281 | 0.746 | 14.441 | 0.116 |
|  | **Head imaging findings on admission** | | | | | |
|  | Location of blood within the subarachnoid space: |  |  |  |  |  |
|  | Basal cistern | % | 3.506 | 0.933 | 13.174 | 0.063 |
|  | **Severity of aneurysmal subarachnoid hemorrhage on admission** | | | | | |
|  | WFNS scale: |  |  |  |  |  |
|  | Grade I | % | - | - | - | 0.006 |
|  | Grade II | % | 3.556 | 0.298 | 42.468 | 0.316 |
|  | Grade III | % | 1.820 | 0.050 | 66.303 | 0.744 |
|  | Grade IV | % | 11.664 | 2.277 | 59.753 | 0.003 |
|  | Grade V | % | 167.047 | 8.804 | 3169.617 | 0.001 |
|  | **Aneurysm repairs and other treatments** | | | | | |
|  | Aneurysm repairs: |  |  |  |  |  |
|  | No aneurysm repair | % | - | - | - | 0.002 |
|  | Endovascular coiling | % | 0.027 | 0.004 | 0.200 | <0.001 |
|  | Surgical clipping | % | 0.044 | 0.006 | 0.354 | 0.003 |
|  | External ventricular drainage |  | 4.368 | 0.830 | 22.977 | 0.082 |
|  | **Complications** | | | | | |
|  | Rebleeding | % | 81.159 | 5.198 | 1267.215 | 0.002 |
|  | Vasospasm and delayed cerebral ischemia | % | 14.667 | 2.289 | 93.962 | 0.005 |
|  | Pneumonia | % | 2.661 | 0.578 | 12.253 | 0.209 |
|  | Constant | % | 0.005 |  |  | 0.003 |
| 9 | **Demographics** | | | | | |
|  | Age (years): |  |  |  |  |  |
|  | 20 - 39 | % | - | - | - | 0.052 |
|  | 40 - 59 | % | 16.272 | 0.858 | 308.485 | 0.063 |
|  | ≥ 60 | % | 39.045 | 1.938 | 786.699 | 0.017 |
|  | **Risk factors of aneurysmal subarachnoid hemorrhage** | | | | | |
|  | Hypertension | % | 3.842 | 0.874 | 16.881 | 0.075 |
|  | **Head imaging findings on admission** | | | | | |
|  | Location of blood within the subarachnoid space: |  |  |  |  |  |
|  | Basal cistern | % | 4.062 | 1.102 | 14.981 | 0.035 |
|  | **Severity of aneurysmal subarachnoid hemorrhage on admission** | | | | | |
|  | WFNS scale: |  |  |  |  |  |
|  | Grade I | % | - | - | - | 0.002 |
|  | Grade II | % | 3.744 | 0.338 | 41.441 | 0.282 |
|  | Grade III | % | 1.480 | 0.036 | 60.288 | 0.836 |
|  | Grade IV | % | 15.285 | 3.096 | 75.466 | 0.001 |
|  | Grade V | % | 162.965 | 9.975 | 2662.318 | <0.001 |
|  | **Aneurysm repairs and other treatments** | | | | | |
|  | Aneurysm repairs: |  |  |  |  |  |
|  | No aneurysm repair | % | - | - | - | 0.003 |
|  | Endovascular coiling | % | 0.033 | 0.005 | 0.235 | 0.001 |
|  | Surgical clipping | % | 0.046 | 0.006 | 0.370 | 0.004 |
|  | External ventricular drainage |  | 5.016 | 1.000 | 25.158 | 0.050 |
|  | **Complications** | | | | | |
|  | Rebleeding | % | 97.624 | 5.653 | 1686.010 | 0.002 |
|  | Vasospasm and delayed cerebral ischemia | % | 15.209 | 2.321 | 99.673 | 0.005 |
|  | Constant | % | 0.005 |  |  | 0.002 |

**S3 Table**. Demographic and baseline characteristics, management and outcomes of patients with aneurysmal subarachnoid hemorrhage according to survivability within 90 days after the onset of hemorrhage

|  | All cases  (n=168) | Survived  (n=129) | Died  (n=39) | p-value |
| --- | --- | --- | --- | --- |
| Transferred from local hospitals, n (%) |  |  |  |  |
| Hospital taken to, n (%) |  |  |  | 0.014 |
| Viet Duc | 22 (13.1) | 22 (17.1) | 0 |  |
| Bach Mai | 129 (76.8) | 93 (72.1) | 36 (92.3) |  |
| Hanoi Medical University | 17 (10.1) | 14 (10.9) | 3 (7.7) |  |
| **Demographics** | | | | |
| Age (year), median (IQR) | 57 (48-67) | 56 (46-66.5) | 62 (53-70) | 0.064 |
| Age (years), n (%) |  |  |  | 0.299 |
| 20 - 39 | 18 (10.7) | 16 (12.6) | 2 (5.1) |  |
| 40 - 59 | 74 (44.0) | 58 (45.0) | 16 (41.0) |  |
| ≥ 60 | 76 (45.2) | 55 (42.6) | 21 (53.8) |  |
| Gender (male), n (%) | 77 (45.8) | 57 (44.2) | 20 (51.3) | 0.436 |
| Resident regions, n (%) |  |  |  | 0.410 |
| Urban | 68 (40.5) | 50 (38.8) | 18 (46.2) |  |
| Rural | 100 (59.5) | 79 (61.2) | 21 (53.8) |  |
| **Socioeconomic status** | | | | |
| Health insurance, n (%) | 152 (90.5) | 120 (93.0) | 32 (82.1) | 0.059 |
| Health insurance rates, n (%) |  |  |  | 0.874 |
| 100% | 30 (19.7) | 24 (20.0) | 6 (18.8) |  |
| 95% | 23 (15.1) | 17 (14.2) | 6 (18.8) |  |
| 80% | 94 (61.8) | 74 (61.7) | 20 (62.5) |  |
| 60% | 1 (0.7) | 1 (0.8) | 0 (0.0) |  |
| 40% | 42.6v | 4 (3.3) | 0 (0.0) |  |
| Occupations, n (%) |  |  |  | 0.127 |
| Managers | 2 (1.2) | 1 (0.8) | 1 (2.6) |  |
| Professionals | 3 (1.8) | 2 (1.6) | 1 (2.6) |  |
| Technicians and associate professionals | 3 (1.8) | 3 (2.3) | 0 (0.0) |  |
| Clerical support workers | 4 (2.4) | 3 (2.3) | 1 (2.6) |  |
| Service and sales workers | 15 (8.9) | 12 (9.3) | 3 (7.7) |  |
| Skilled agricultural, forestry and fishery workers | 36 (3.6) | 23 (17.8) | 13 (33.3) |  |
| Craft and related trades workers | 0 | 0 | 0 |  |
| Plant and machine operators, and assemblers | 6 (3.6) | 5 (3.9) | 1 (2.6) |  |
| Elementary occupations | 46 (27.4) | 41 (31.8) | 5 (12.8) |  |
| Armed forces occupations | 0 | 0 | 0 |  |
| Retired | 49 (29.2) | 36 (27.9) | 13 (33.3) |  |
| Student/unemployment | 1 (0.6) | 0 (0.0) | 1 (2.6) |  |
| Other | 3 (1.8) | 3 (2.3) | 0 (0.0) |  |
| Highest education levels, n (%) |  |  |  | 0.660 |
| School | 126 (75.0) | 97 (75.2) | 29 (74.4) |  |
| Vocational school | 12 (7.1) | 10 (7.8) | 2 (5.1) |  |
| Junior college | 8 (4.8) | 6 (4.7) | 2 (5.1) |  |
| Senior college | 15 (8.9) | 11 (8.5) | 4 (10.3) |  |
| College/University | 6 (3.6) | 5 (3.9) | 1 (2.6) |  |
| Postgraduate | 1 (0.6) | 0 (0.0) | 1 (2.6) |  |
| Annual income, n (%) |  |  |  | 0.398 |
| Upper-middle (US $4,036 - US $12,475) | 18 (10.8) | 13 (10.2) | 5 (12.8) |  |
| Lower-middle (US $1,026 - US $4,035) | 115 (68.9) | 86 (67.2) | 29 (74.4) |  |
| Low (≤ US $1,025) | 34 (20.4) | 29 (22.7) | 5 (12.8) |  |
| **Risk factors of aneurysmal subarachnoid hemorrhage** | | | | |
| Cigarette smoking, n (%) | 63 (37.5) | 46 (35.7) | 17 (43.6) | 0.370 |
| Smoking behaviors, n (%) |  |  |  | 0.350 |
| Quit | 18 (28.6) | 15 (32.6) | 3 (17.6) |  |
| Current | 45 (71.4) | 31 (67.4) | 14 (82.4) |  |
| Hypertension, n (%) | 64 (38.1) | 43 (33.3) | 21 (53.8) | 0.021 |
| Genetic risk, n (%) | 6 (3.6) | 5 (3.9) | 1 (2.6) | 0.999 |
| Inherited conditions, n (%) |  |  |  | 0.999 |
| Polycystic kidney disease | 1 (16.7) | 1 (20.0) | 0 |  |
| A family history of subar-achnoid hemorrhage/unru-ptured aneurysms | 5 (83.3) | 4 (80.0) | 1 (100) |  |
| Alcohol consumption, n (%) | 81 (48.2) | 60 (46.5) | 21 (53.8) | 0.422 |
| Alcohol drinking behaviors, n (%) |  |  |  | 0.382 |
| Occasionally | 45 (55.6) | 31 (51.7) | 14 (66.7) |  |
| Sometimes | 25 (30.9) | 21 (35.0) | 4 (19.0) |  |
| Every day | 11 (13.6) | 8 (13.3) | 3 (14.3) |  |
| Sympathomimetic drugs, n (%) | 2/167 (1.2) | 2/128 (1.6) | 0/39 (0.0) | 0.432 |
| Estrogen deficiency, n (%) | 32/89 (36.0) | 26/70 (37.1) | 6/19 (31.6) | 0.654 |
| Antithrombotic therapy, n (%) | 3 (1.8) | 2 (1.6) | 1 (2.6) | 0.550 |
| Elevated total cholesterol, n (%) | 8 (4.8) | 6 (4.7) | 2 (5.1) | 0.999 |
| **Preexisting comorbidities** | | | | |
| Cerebrovascular disease, n (%) | 3 (1.8) | 2 (1.6) | 1 (2.6) | 0.550 |
| Chronic cardiac failure, n (%) | 3 (1.8) | 2 (1.6) | 1 (2.6) | 0.550 |
| Coronary artery disease/IM, n (%) | 3 (1.8) | 2 (1.6) | 1 (2.6) | 0.550 |
| COPD /Asthma, n (%) | 0 | 0 | 0 | - |
| Chronic pulmonary disease, n (%) | 0 | 0 | 0 | - |
| Tuberculosis, n (%) | 0 | 0 | 0 | - |
| Active neoplasm, n (%) | 3 (1.8) | 3 (2.3) | 0 (0.0) | 0.999 |
| Chronic renal failure, n (%) | 2 (1.2) | 1 (0.8) | 1 (2.6) | 0.411 |
| Ulcer disease, n (%) | 0 | 0 | 0 | - |
| Diabetes mellitus, n (%) | 15 (8.9) | 11 (8.5) | 4 (10.3) | 0.752 |
| Immunoincompetence, n (%) | 0 | 0 | 0 | - |
| Hematological disease, n (%) | 2 (1.2) | 2 (1.6) | 0 (0.0) | 0.999 |
| Others, n (%) | 17 (10.1) | 14 (10.9) | 3 (7.7) | 0.765 |
| **Onset symptoms** | | | | |
| Sudden-onset, severe headache, n (%) | 147 (87.5) | 115 (89.1) | 32 (82.1) | 0.271 |
| Vomiting, n (%) | 102 (60.7) | 81 (62.8) | 21 (53.8) | 0.316 |
| Neck pain or stiffness, n (%) | 66 (39.3) | 52 (40.3) | 14 (35.9) | 0.621 |
| Photophobia, n (%) | 6 (3.6) | 5 (3.9) | 1 (2.6) | 0.999 |
| Blurred or double vision, n (%) | 4 (2.4) | 3 (2.3) | 1 (2.6) | 0.999 |
| Brief loss of consciousness, n (%) | 70 (41.7) | 43 (33.3) | 27 (69.2) | <0.001 |
| Seizures, n (%) | 9 (5.4) | 7 (5.4) | 2 (5.1) | 0.999 |
| Other, n (%) | 132 (78.6) | 96 (74.4) | 36 (92.3) | 0.017 |
| **Clinical presentation on admission** | | | | |
| GCS score, median (IQR) | 14 (9-15) | 15 (12-15) | 7 (6-10) | <0.001 |
| GCS score, n (%) |  |  |  | <0.001 |
| Mild (13 - 15) | 10 (59.5) | 94 (72.9) | 6 (15.4) |  |
| Moderate (9 - 12) | 29 (17.3) | 20 (15.5) | 9 (23.1) |  |
| Severe (3 - 8) | 39 (23.2) | 15 (11.6) | 24 (61.5) |  |
| Heart rate (beats/min), median (IQR), n=167 | 85 (76-95) | 84.5 (75-90) | 90 (80-100) | 0.011 |
| Heart rate (beats/min), n (%) | n=167 | n=128 | n=39 | 0.006 |
| ≤ 95 | 126 (75.4) | 103 (80.5) | 23 (59.0) |  |
| > 95 | 41 (24.6) | 25 (19.5) | 16 (41.0) |  |
| Respiratory rate (breaths/min), median (IQR), (n=159) | 20 (18-21) | 20 (18-20.5) | 20 (18-25) | 0.812 |
| Respiratory rate (breaths/min), n (%) | n=159 | n=125 | n=34 | 0.034 |
| < 12 | 0 | 0 | 0 |  |
| 12 - 25 | 146 (91.8) | 118 (94.4) | 28 (82.4) |  |
| > 25 | 13 (9.2) | 7 (5.6) | 6 (17.6) |  |
| Systolic blood pressure (mmHg), mean (SD), n=167 | 137.40 (28.07) | 135.98 (22.05) | 142.03 (42.29) | 0.330 |
| Systolic blood pressure (mmHg), n (%) | n=167 | n=128 | n=39 | 0.383 |
| < 140 | 83 (49.7) | 66 (51.6) | 17 (43.6) |  |
| ≥ 140 | 84 (50.3) | 62 (48.4) | 22 (56.4) |  |
| Diastolic blood pressure (mmHg), mean (SD), n=167 | 79.90 (13.73) | 78.68 (11.78) | 83.90 (18.37) | 0.078 |
| Diastolic blood pressure (mmHg), n (%) | n=167 | n=128 | n=39 | 0.012 |
| < 90 | 117 (70.1) | 96 (75.0) | 21 (53.8) |  |
| ≥ 90 | 50 (29.9) | 32 (25.0) | 18 (46.2) |  |
| Body temperature (^o^C), mean (SD), n=167 | 36.93 (0.45) | 36.92 (0.48) | 36.96 (0.32) | 0.429 |
| Body temperature (^o^C), n (%) | n=167 | n=128 | n=39 | 0.666 |
| < 38 | 160 (95.8) | 123 (96.1) | 37 (94.9) |  |
| ≥ 38 | 7 (4.2) | 5 (3.9) | 2 (5.1) |  |
| Focal neurological deficits, n (%) | 99 (58.9) | 71 (55.0) | 28 (71.8) | 0.062 |
| Focal neurologic signs, n (%): |  |  |  |  |
| Third nerve palsy | 4 (4.0) | 5 (5.6) | 0 (0.0) | 0.575 |
| Sixth nerve palsy | 1 (1.0) | 1 (1.4) | 0 (0.0) | 0.999 |
| Hemiparesis | 33 (33.3) | 22 (31.0) | 11 (39.3) | 0.430 |
| Aphasia | 6 (6.1) | 4 (5.6) | 2 (7.1) | 0.999 |
| Bilateral leg weakness | 1 (1.0) | 1 (1.4) | 0 (0.0) | 0.999 |
| Ophthalmoplegia | 0 | 0 | 0 | - |
| Unilateral visual loss or bitemporal hemianopia | 0 | 0 | 0 | - |
| Impaired level of consciousness | 49 (49.5) | 27 (38.0) | 22 (78.6) | <0.001 |
| Brainstem signs | 3 (3.0) | 1 (1.4) | 2 (7.1) | 0.192 |
| Neck stiffness | 52 (52.5) | 40 (56.3) | 12 (42.9) | 0.226 |
| Retinal and subhyaloid hemorrhages | 0 | 0 | 0 | - |
| Preretinal hemorrhages (Terson syndrome) | 0 | 0 | 0 | - |
| Other | 0 | 0 | 0 | - |
| **Head imaging findings on admission** | | | | |
| Non-contrast head computed tomography (CT) findings | | | | |
| Detection of blood within the subarachnoid space, n (%) | 167 (99.4) | 128 (99.2) | 39 (100.0) | 0.999 |
| Location of blood within the subarachnoid space, n (%) |  |  |  |  |
| Basal cistern | 79/165 (47.9) | 55/128 (43.0) | 24/37 (64.9) | 0.019 |
| Sylvian fissure | 157 (94.0) | 121 (94.5) | 36 (92.3) | 0.700 |
| Interhemispheric fissure | 84/166 (50.6) | 59/128 (46.1) | 25/38 (65.8) | 0.033 |
| Interpeduncular fossa | 83/166 (50.0) | 56/128 (43.8) | 27/38 (71.1) | 0.003 |
| Suprasellar cistern | 90/166 (54.2) | 66/128 (51.6) | 24/38 (63.2) | 0.208 |
| Ambient cistern | 81/166 (48.8) | 54/128 (42.2) | 27/38 (71.1) | 0.002 |
| Quadrigeminal cistern | 27/166 (16.3) | 13/128 (10.2) | 14/38 (36.8) | <0.001 |
| Maximum thickness of subarachnoid blood (mm), mean (SD), n=165 | 6.30 (4.56) | 5.90 (4.60) | 7.63 (4.21) | 0.014 |
| IVH, n (%) | 107 (63.7) | 75 (58.1) | 32 (82.1) | 0.007 |
| Location of blood within the ventricular system, n (%) |  |  |  |  |
| Right lateral ventricle | 95/106 (89.6) | 64/74 (86.5) | 31/32 (96.9) | 0.167 |
| Left lateral ventricle | 99/106 (93.4) | 67/74 (90.5) | 32/32 (100.0) | 0.099 |
| Third ventricle | 42/106 (39.6) | 18/74 (24.3) | 24/32 (75.0) | <0.001 |
| Fourth ventricle | 43/106 (40.6) | 24/74 (32.4) | 19/32 (59.4) | 0.010 |
| Graeb score, median (IQR), n=102 | 3 (2-5.25) | 3 (2-4) | 5 (3-7) | <0.001 |
| Graeb score, n (%) | n=102 | n=71 | n=31 | <0.001 |
| Mild (1 - 4) | 69 (67.6) | 57 (80.3) | 12 (38.7) |  |
| Moderate (5 - 8) | 24 (23.5) | 9 (12.7) | 15 (48.4) |  |
| Severe (9 - 12) | 9 (8.8) | 5 (7.0) | 4 (12.9) |  |
| ICH, n (%) | 41 (24.4) | 26 (20.2) | 16 (38.5) | 0.020 |
| ICH volume (mL), mean (SD), n=41 | 29.29 (28.21) | 21.70 (22.56) | 42.44 (32.75) | 0.035 |
| ICH volume (mL), n (%) | n=41 | n=26 | n=16 | 0.166 |
| < 30 | 26 (63.4) | 19 (73.1) | 7 (46.7) |  |
| 30 - 60 | 7 (17.1) | 4 (15.4) | 3 (20.0) |  |
| > 60 | 8 (19.5) | 3 (11.3) | 5 (33.3) |  |
| Subdural hemorrhage, n (%) | 10 (6.0) | 6 (4.7) | 4 (10.3) | 0.243 |
| Hydrocephalus, n (%) | 72 (42.9) | 48 (37.2) | 24 (61.5) | 0.007 |
| Evans' index, mean (SD), n=145 | 0.30 (0.06) | 0.30 (0.06) | 0.31 (0.07) | 0.247 |
| Bicaudate index, mean (SD), n=145 | 0.20 (0.06) | 0.19 (0.06) | 0.22 (0.08) | 0.022 |
| Relative bicaudate index, mean (SD), n=145 | 1.07 (0.34) | 1.02 (0.30) | 1.19 (0.41) | .03 |
| Hypodense lesions on computed tomography, n (%) | 14/167 (8.4) | 10/129 (7.8) | 4/38 (10.5) | 0.525 |
| Patterns of infarction, n (%) | n=14 | n=10 | n=4 |  |
| Single cortical infarcts | 6 (42.9) | 5 (50.0) | 1 (25.0) | 0.580 |
| Multiple widespread infarcts | 8 (57.1) | 5 (50.0) | 3 (75.0) | 0.580 |
| Multislice computed tomography (MSCT) angiography / Digital subtraction angiography (DSA) findings | | | | |
| Detection of intracranial aneurysm, n (%) | 168 (100) | 129 (100) | 39 (100) |  |
| Number of aneurysm, median (IQR) | 1 (1-1)/167 | 1 (1-1)/128 | 1 (1-1)/39 | 0.165 |
| Number of aneurysm, n (%) |  |  |  | 0.170 |
| Single aneurysm | 144 (85.7) | 108 (83.7) | 36 (92.3) |  |
| Multiple aneurysms | 24 (14.3) | 21 (16.3) | 3 (7.7) |  |
| Side of aneurysm, n (%) |  |  |  | 0.532 |
| Right | 58 (34.7) | 42 (32.8) | 16 (41.0) |  |
| Left | 49 (29.3) | 40 (31.2) | 9 (23.1) |  |
| Both | 60 (35.9) | 46 (35.9) | 14 (35.9) |  |
| Groups of aneurysm site, n (%) |  |  |  |  |
| Anterior circulation aneurysm | 120/150 (80.0) | 89/116 (76.7) | 31/34 (91.2) | 0.064 |
| Posterior circulation aneurysm | 33/150 (22.0) | 30/116 (25.9) | 3/34 (8.8) | 0.035 |
| Aneurysm site, n (%) | n=168 | n=129 | n=39 |  |
| Internal carotid artery (ICA) | 35 (20.8) | 27 (20.9) | 8 (20.5) | 0.955 |
| Ophtalmic segment of the ICA (OphIC) | 1 (0.6) | 1 (0.8) | 0 (0.0) | 0.999 |
| Cavernous segment of the ICA (cIC) | 7 (4.2) | 5 (3.9) | 2 (5.1) | 0.664 |
| Anterior choroidal artery segment of the ICA (AchIC) | 0 | 0 | 0 | - |
| Posterior communicating artery (PCoA) | 25 (14.9) | 21 (16.3) | 4 (10.3) | 0.354 |
| Anterior cerebral artery (ACA) | 16 (9.5) | 13 (10.1) | 3 (7.7) | 0.999 |
| Anterior communicating artery (AcoA) | 49 (29.2) | 37 (28.7) | 12 (30.8) | 0.802 |
| Middle cerebral artery (MCA) | 34 (20.2) | 26 (20.2) | 8 (20.5) | 0.961 |
| Posterior cerebral artery (PCA) | 0 | 0 | 0 | - |
| Vertebral artery (VA) | 7 (4.2) | 5 (3.9) | 2 (5.1) | 0.664 |
| Superior cerebellar artery (SCA) | 0 | 0 | 0 | - |
| Posterior inferior cerebellar artery (PICA) | 4 (2.4) | 3 (2.3) | 1 (2.6) | 0.999 |
| Anterior inferior cerebellar artery (AICA) | 0 | 0 | 0 | - |
| Basilar artery (BA) | 7 (4.2) | 7 (5.4) | 0 (0.0) | 0.203 |
| Other | 129 (76.8) | 93 (72.1) | 36 (92.3) | 0.009 |
| Maximum aneurysm size (mm), mean (SD), |  |  |  |  |
| Maximum dome width, n=166 | 4.75 (2.70) | 4.67 (2.53) | 5.02 (3.21) | 0.589 |
| Dome height, n=163 | 5.50 (5.79) | 5.40 (6.38) | 5.84 (3.20) | 0.252 |
| Maximum neck width, n=158 | 3.35 (2.83) | 3.28 (3.08) | 3.58 (1.85) | 0.138 |
| Dome-to-neck ratio, n=157 | 1.65 (3.71) | 1.71 (4.24) | 1.46 (0.75) | 0.335 |
| Shape of aneurysm, n (%) | n=149 | n=110 | n=39 | 0.758 |
| Regular | 18 (12.1) | 15 (13.6) | 3 (7.7) |  |
| Irregular | 117 (78.5) | 85 (77.3) | 32 (82.1) |  |
| Multilobular | 11 (7.4) | 8 (7.3) | 3 (7.7) |  |
| Two domes |  |  |  |  |
| Other | 3 (2.0) | 2 (1.8) | 1 (2.6) |  |
| Vasospasm of the parent artery, n (%), n=166 | 14 (8.4) | 7 (5.5) | 7 (17.9) | 0.022 |
| **Severity of aneurysmal subarachnoid hemorrhage on admission** | | | | |
| PAASH score, median (IQR) | 2 (1-3) | 1 (1-2) | 4 (3-4) | <0.001 |
| PAASH scale, n (%) |  |  |  | <0.001 |
| Grade I | 80 (47.6) | 77 (59.7) | 3 (7.7) |  |
| Grade II | 31 (18.5) | 26 (20.2) | 5 (12.8) |  |
| Grade III | 28 (16.7) | 17 (13.2) | 11 (28.2) |  |
| Grade IV | 25 (14.9) | 7 (5.4) | 18 (46.2) |  |
| Grade V | 4 (2.4) | 2 (1.6) | 2 (5.1) |  |
| WFNS score, median (IQR) | 2 (1-4) | 1 (1-4) | 4 (4-5) | <0.001 |
| WFNS scale, n (%) |  |  |  | <0.001 |
| Grade I | 80 (47.6) | 77 (59.7) | 3 (7.7) |  |
| Grade II | 14 (8.3) | 12 (9.3) | 2 (5.1) |  |
| Grade III | 6 (3.6) | 5 (3.9) | 1 (2.6) |  |
| Grade IV | 50 (29.8) | 30 (23.3) | 20 (51.3) |  |
| Grade V | 18 (10.7) | 5 (3.9) | 13 (33.3) |  |
| Modified WFNS score, median (IQR) | 2 (1-4) | 1 (1-4) | 4 (4-5) | <0.001 |
| Modified WFNS scale, n (%) |  |  |  | <0.001 |
| Grade I | 80 (47.6) | 77 (59.7) | 3 (7.7) |  |
| Grade II | 14 (8.3) | 12 (9.3) | 2 (5.1) |  |
| Grade III | 6 (3.6) | 5 (3.9) | 1 (2.6) |  |
| Grade IV | 50 (29.8) | 30 (23.3) | 20 (51.3) |  |
| Grade V | 18 (10.7) | 5 (3.9) | 13 (33.3) |  |
| Hunt and Hess score, median (IQR) | 2.5 (2-4) | 2 (1-3) | 5 (4-5) | <0.001 |
| Hunt and Hess scale, n (%) |  |  |  | <0.001 |
| Grade 1 | 39 (23.2) | 38 (29.5) | 1 (2.6) |  |
| Grade 2 | 45 (26.8) | 43 (33.3) | 2 (5.1) |  |
| Grade 3 | 25 (14.9) | 20 (15.5) | 5 (12.8) |  |
| Grade 4 | 21 (12.5) | 14 (10.9) | 7 (17.9) |  |
| Grade 5 | 38 (22.6) | 14 (10.9) | 24 (61.5) |  |
| Fisher score, median (IQR) | 4 (3-4) | 4 (3-4) | 4 (4-4) | 0.003 |
| Fisher scale, n (%) |  |  |  | 0.015 |
| Group 1 | 1 (0.6) | 1 (0.8) | 0 (0.0) |  |
| Group 2 | 13 (7.7) | 13 (10.1) | 0 (0.0) |  |
| Group 3 | 34 (20.2) | 30 (23.3) | 4 (10.3) |  |
| Group 4 | 120 (71.4) | 85 (65.9) | 35 (89.7) |  |
| Claassen score, median (IQR) | 5 (4-5) | 4 (4-5) | 5 (5-5) | <0.001 |
| Claassen scale, n (%) |  |  |  | 0.003 |
| Grade 1 |  |  |  |  |
| Grade 2 | 22 (13.1) | 20 (15.5) | 2 (5.1) |  |
| Grade 3 | 11 (6.5) | 11 (8.5) | 0 (0.0) |  |
| Grade 4 | 39 (23.2) | 34 (26.4) | 5 (12.8) |  |
| Grade 5 | 96 (57.1) | 64 (49.6) | 32 (82.1) |  |
| **Laboratory investigations on admission** | | | | |
| Complete blood count |  |  |  |  |
| Red blood cells (T/L), mean (SD) | 4.46 (0.78) | 4.43 (0.83) | 4.53 (0.58) | 0.311 |
| Hemoglobin (g/L), mean (SD) | 132.07 (21.09) | 131.12 )19.76) | 135.21 (25.03) | 0.075 |
| Hematocrit (L/L), mean (SD) | 0.40 (0.04) | 0.39 (0.05) | 0.41 (0.04) | 0.039 |
| Platelets (G/L), mean (SD) | 247.21 (76.12) | 246.48 (77.02) | 249.63 (74.04) | 0.972 |
| White blood cells (G/L), mean (SD) | 19.30 (54.87) | 14.02 (8.65) | 36.67 (111.93) | <0.001 |
| Percentage of neutrophils (%), mean (SD), (n=165) | 70.58 (28.72) | 67.98 (29.99) | 79.07 (22.41) | 0.003 |
| Coagulation |  |  |  |  |
| Prothrombin time (PT), mean (SD) | 91.95 (32.59) | 89.80 (35.84) | 79.07 (22.41) | 0.519 |
| Prothrombin time with INR (PT-INR), mean (SD) | 1.00 (0.09) | 1.00 (0.09) | 1.02 (0.10) | 0.733 |
| Activated partial thromboplastin time (APTT), mean (SD), (n=155) | 44.21 (200.54) | 28.05 (4.42) | 98.67 (419.24) | 0.884 |
| rAPTT, mean (SD), (n=154) | 1.55 (6.82) | 1.69 (7.77) | 1.06 (0.49) | 0.835 |
| Blood biochemical investigation |  |  |  |  |
| Ure (mmol/L), mean (SD), (n=169) | 5.39 (1.80) | 5.27 (1.82) | 5.77 (1.71) | 0.084 |
| Glucose (mmol/L), mean (SD), (n=159) | 8.21 (2.42) | 7.63 (2.12) | 10.06 (2.42) | <0.001 |
| Glucose (mmol/L), n (%) |  |  |  | 0.001 |
| ≤ 6.4 | 29 (18.6) | 29 (24.4) | 0 (0.0) |  |
| > 6.4 | 127 (81.4) | 90 (75.6) | 37 (100.0) |  |
| Creatinine (µmol/L), mean (SD) | 67.40 (25.40) | 64.67 (25.24) | 76.38 (24.09) | 0.001 |
| SGOT (UI/L), mean (SD), (n=160) | 34.30 (26.01) | 27.95 (12.40) | 53.52 (42.42) | <0.001 |
| SGPT (UI/L), mean (SD), (n=168) | 28.77 (24.01) | 24.64 (14.80) | 42.09 (39.11) | <0.001 |
| Na^+^ (mmol/L), mean (SD) | 136.62 (10.76) | 135.87 (11.82) | 139.05 (5.62) | 0.022 |
| Na^+^ (mmol/L), n (%) |  |  |  | 0.086 |
| ≥ 135 | 128 (76.6) | 94 (73.4) | 34 (87.2) |  |
| < 135 | 39 (23.4) | 34 (26.6) | 5 (12.8) |  |
| K^+^ (mmol/L), mean (SD) | 3.56 (0.46) | 3.57 (0.44) | 3.55 (0.54) | 0.835 |
| Cl^-^ (mmol/L), mean (SD) | 99.95 (8.89) | 99.65 (9.55) | 100.94 (6.25) | 0.959 |
| **Aneurysm repairs and other treatments** | | | | |
| No aneurysm repair, n (%) | 26 (15.5) | 4 (3.1) | 22 (56.4) | <0.001 |
| Endovascular coiling, n (%) | 79 (47.0) | 71 (55.0) | 8 (20.5) | <0.001 |
| Number of coils, median (IQR) | 5 (3-7) | 5 (4-7) | 3 (3-5) | 0.064 |
| Stent-assisted coiling, n (%) | 2 (2.6) | 2 (2.9) | 0 (0.0) | 0.999 |
| Balloon-assisted coiling, n (%) | 9 (11.7) | 9 (13.0) | 0 (0.0) | 0.585 |
| Surgical clipping, n (%) | 63 (37.5) | 54 (41.9) | 9 (23.1) | 0.034 |
| Number of clip attempts, median (IQR) | 4 (2-5) | 3.5 (1.75-5) | 4 (3-4) | 0.674 |
| Temporary vessel occlusion of the parent artery, n (%) | 7 (15.2) | 6 (15.0) | 1 (16.7) | 0.999 |
| Surgical hematoma evacuation or decompressive craniotomy, n (%) | 7 (4.2) | 3 (2.3) | 4 (10.3) | 0.051 |
| External ventricular drainage, n (%) | 26 (15.6) | 18 (14.1) | 8 (20.5) | 0.331 |
| Number of EVD, median (IQR) | 1 (1-1) | 1 (1-1) | 1 (1-1) | 0.336 |
| Intraventricular fibrinolysis, n (%) | 3 (1.8) | 2 (1.6) | 1 (2.6) | 0.550 |
| **Airway management and mechanical ventilation** | | | | |
| Tracheal intubation, n (%) | 144/151 (95.4) | 105/112 (93.8) | 39/39 (100.0) | 0.191 |
| Mechanical ventilation, n (%) | 141/149 (94.6) | 102/110 (92.7) | 39/39 (100.0) | 0.112 |
| Tracheotomy, n (%) | 15/161 (9.3) | 11/123 (8.9) | 38 (10.5) | 0.754 |
| **Medical treatment** | | | | |
| Nimodipine for preventing and treating cerebral vasospasm, n (%) | 158/167 (94.6) | 124/128 (96.9) | 34/39 (87.2) | 0.033 |
| Route of administration of nimodipine, n (%) |  |  |  |  |
| Oral | 113 (71.5) | 84 (67.7) | 29 (85.3) | 0.045 |
| Intravenous | 60 (38.0) | 52 (41.9) | 8 (23.5) | 0.050 |
| Deep venous thrombosis prophylaxis, n (%) | 16/151 (10.6) | 12/115 (10.4) | 4/36 (11.1) | 0.999 |
| **Complications** | | | | |
| Rebleeding, n (%) | 10/164 (6.1) | 5/128 (3.9) | 5/36 (13.9) | 0.042 |
| Number of rebleeding sites, n (%): |  |  |  | 0.524 |
| Rebleeding from a single site | 4 (40.0) | 1 (20.0) | 3 (60.0) |  |
| Rebleeding from multiple sites | 6 (60.0) | 4 (80.0) | 2 (40.0) |  |
| Rebleeding sites, n (%) |  |  |  |  |
| Subarachnoid hemorrhage, (n=9) | 3 (33.3) | 1 (25.0) | 2 (40.0) | 0.999 |
| Subdural hemorrhage | 4 (40.0) | 4 (80.0) | 0 (0.0) | 0.048 |
| Intraventricular hemorrhage | 6 (60.0) | 3 (60.0) | 3 (60.0) | 0.999 |
| Intracerebral hemorrhage | 7 (70.0) | 4 (80.0) | 3 (60.0) | 0.999 |
| Vasospasm and delayed cerebral ischemia, n (%) | 17 (10.4) | 11 (8.8) | 6 (15.8) | 0.232 |
| Acute hydrocephalus, n (%) | 76 (45.2) | 51 (39.5) | 25 (64.1) | 0.007 |
| Hyponatremia, n (%) | 34 (20.2) | 26 (20.2) | 8 (20.5) | 0.961 |
| Seizures, n (%) | 44 (26.2) | 40 (31.0) | 4 (10.3) | 0.010 |
| Chronic hydrocephalus, n (%) | 5/83 (6.0) | 4/77 (5.2) | 1/6 (16.7) | 0.320 |
| EVD obstruction, n (%) | 2/22 (9.1) | 2/14 (14.3) | 0/8 | 0.515 |
| EVD replacement, n (%) | 2/19 (10.5) | 2/13 (15.4) | 0/6 | >0.999 |
| Ventriculitis, n (%) | 8/126 (6.3) | 7/92 (7.6) | 1/34 (2.9) | 0.681 |
| Pneumonia, n (%) | 29 (17.3) | 21 (16.3) | 8 (20.5) | 0.540 |
| Urinary tract infection, n (%) | 3 (1.8) | 3 (2.3) | 0 (0.0) |  |
| **Clinical time course** | | | | |
| Ictus to hospital arrival (days), mean (SD) | 1.03 (2.57) | 1.20 (2.90) | 0.50 (0.56) | 0.026 |
| Ictus to hospital arrival (days) |  |  |  | 0.384 |
| ≤ 24 hours | 69 (42.9) | 49 (39.8) | 20 (52.6) |  |
| >24–72 hours | 91 (56.5) | 73 (59.3) | 18 (47.4) |  |
| >72 hours | 1 (0.6) | 1 (0.8) | 0 (0.0) |  |
| EVD duration (days), mean (SD), n=25 | 6.88 (4.60) | 7.56 (4.66) | 5.14 (4.30) | 0.135 |
| EVD duration (days), n (%) |  |  |  | 0.362 |
| ≤ 7 | 17 (68.0) | 11 (61.1) | 6 (85.7) |  |
| > 7 | 8 (32.0) | 7 (38.9) | 1 (14.3) |  |
| Length of hospitalization (days), mean (SD) | 10.85 (9.40) | 12.00 (9.13) | 7.05 (9.40) | <0.001 |
| **Clinical outcomes** | | | | |
| Hospital discharge, n (%) | 52 (31.0) | 52 (40.3) | 0 (0.0) | <0.001 |
| Transferred to another hospital, n (%) | 91 (54.2) | 72 (55.8) | 19 (48.7) | 0.436 |
| Discharged to die, n (%) | 19 (11.3) | 54 (3.9) | 14 (35.9) | <0.001 |
| Died in hospital, n (%) | 6 (3.6) | 0 (0.0) | 6 (15.4) | <0.001 |
| Died within 30 days of ictus, n (%) | 35 (20.8) | 0 (0.0) | 35 (89.7) | <0.001 |
| **Neurological function** | | | | |
| mRS score at hospital discharge, median (IQR) | 1 (1-5) | 1 (1-2) | 5 (5-5) | <0.001 |
| mRS at hospital discharge, n (%) |  |  |  | <0.001 |
| Good (mRS of 0 to 3) | 109 (64.9) | 108 (83.7) | 1 (2.6) |  |
| Poor (mRS of 4 to 6) | 59 (35.1) | 21 (16.3) | 38 (97.4) |  |
| mRS score at 30 days of ictus, median (IQR) | 1 (0-5) | 1 (0-2) | 6 (6-6) | <0.001 |
| mRS at 30 days after the onset of hemorrhage, n (%) |  |  |  | <0.001 |
| Good (mRS of 0 to 3) | 110 (65.5) | 110 (85.3) | 0 (0.0) |  |
| Poor (mRS of 4 to 6) | 58 (34.5) | 19 (14.7) | 39 (100.0) |  |
| GOS score at hospital discharge, median (IQR) | 4 (2-5) | 5 (4-5) | 2 (2-2) | <0.001 |
| GOS at hospital discharge, n (%) |  |  |  | <0.001 |
| Good (GOS of 3 to 5) | 123 (73.2) | 119 (92.2) | 4 (10.3) |  |
| Poor (GOS of 1 to 2) | 45 (26.8) | 10 (7.8) | 35 (89.7) |  |
| GOS score at 30 days after the onset of hemorrhage, median (IQR) | 5 (2-5) | 5 (4-5) | 1 (1-1) | <0.001 |
| GOS at 30 days after the onset of hemorrhage, n (%) |  |  |  | <0.001 |
| Good (GOS of 3 to 5) | 120 (71.4) | 119 (92.2) | 1 (2.6) |  |
| Poor (GOS of 1 to 2) | 48 (28.6) | 10 (7.8) | 38 (97.4) |  |

**S4 Table**. Factors associated with death at 90 days after the onset of hemorrhage in patients with aneurysmal subarachnoid hemorrhage: bivariate regression analyses

|  | Frequency | OR | 95.0% CI for OR | | p-value |
| --- | --- | --- | --- | --- | --- |
|  |  |  | Lower | Upper |  |
| Transferred from local hospitals, n (%) |  |  |  |  |  |
| Hospital taken to, n (%) |  |  |  |  |  |
| Viet Duc | 22 | - | - | - | - |
| Bach Mai | 129 | 625345162.2 | 0.000 | - | 0.998 |
| Hanoi Medical University | 17 | 346173214.8 | 0.000 | - | 0.998 |
| **Demographics** | | | | | |
| Age (year) | 168 | 1.026 | 0.998 | 1.055 | 0.072 |
| Age (years): |  |  |  |  |  |
| 20 - 39 | 18 | - | - | - | - |
| 40 - 59 | 74 | 2.207 | 0.459 | 10.615 | 0.323 |
| ≥ 60 | 76 | 3.055 | 0.646 | 14.443 | 0.159 |
| Gender (male) | 77 | 1.330 | 0.649 | 2.725 | 0.436 |
| Resident regions: |  |  |  |  |  |
| Urban | 68 | - | - | - | - |
| Rural | 100 | 0.738 | 0.359 | 1.521 | 0.411 |
| **Socioeconomic status** | | | | | |
| Health insurance | 152 | 0.343 | 0.119 | 0.992 | 0.048 |
| Health insurance rates: |  |  |  |  |  |
| 100% | 30 | - | - | - | - |
| 95% | 23 | 1.412 | 0.388 | 5.133 | 0.601 |
| 80% | 94 | 1.081 | 0.389 | 3.004 | 0.881 |
| 60% | 1 | 0.000 | 0.000 |  | >0.999 |
| 40% | 4 | 0.000 | 0.000 |  | 0.999 |
| Highest education levels: |  |  |  |  |  |
| School | 126 | - | - | - | - |
| Vocational school | 12 | 0.669 | 0.139 | 3.228 | 0.617 |
| Junior college | 8 | 1.115 | 0.213 | 5.824 | 0.897 |
| Senior college | 15 | 1.216 | 0.360 | 4.108 | 0.753 |
| College/University | 6 | 0.669 | 0.075 | 5.958 | 0.719 |
| Postgraduate | 1 | 5403484893 | 0.000 |  | >0.999 |
| Annual income: |  |  |  |  |  |
| Upper-middle (US $4,036 - US $12,475) | 18 | - | - | - | - |
| Lower-middle (US $1,026 - US $4,035) | 115 | 0.877 | 0.288 | 2.671 | 0.817 |
| Low (≤ US $1,025) | 34 | 0.448 | 0.110 | 1.821 | 0.262 |
| **Risk factors for aneurysmal subarachnoid hemorrhage** | | | | | |
| Cigarette smoking | 63 | 1.394 | 0.673 | 2.888 | 0.371 |
| Smoking behaviors: |  |  |  |  |  |
| Quit | 18 | - | - | - | - |
| Current | 45 | 2.258 | 0.5662 | 9.075 | 0.251 |
| Hypertension | 64 | 2.333 | 1.126 | 4.833 | 0.023 |
| Genetic risk | 6 | 0.653 | 0.074 | 5.759 | 0.701 |
| Alcohol consumption | 81 | 1.342 | 0.654 | 2.752 | 0.423 |
| Alcohol drinking behaviors: |  |  |  |  |  |
| Occasionally | 45 | - | - | - | - |
| Sometimes | 25 | 0.422 | 0.122 | 1.460 | 0.173 |
| Every day | 11 | 0.830 | 0.191 | 3.609 | 0.804 |
| Sympathomimetic drugs | 2 | 0.000 | 0.000 |  | 0.999 |
| Estrogen deficiency | 32 | 0.781 | 0.265 | 2.305 | 0.654 |
| Antithrombotic therapy | 3 | 1.671 | 0.147 | 18.937 | 0.678 |
| Elevated total cholesterol | 8 | 1.108 | 0.215 | 5.724 | 0.902 |
| **Comorbidities** | | | | | |
| Cerebrovascular disease | 3 | 1.671 | 0.147 | 18.937 | 0.678 |
| Chronic cardiac failure | 3 | 1.671 | 0.147 | 18.937 | 0.678 |
| Coronary artery disease/IM | 3 | 1.671 | 0.147 | 18.937 | 0.678 |
| Active neoplasm | 3 | 0.000 | 0.000 |  | 0.999 |
| Chronic renal failure | 2 | 3.368 | 0.206 | 55.136 | 0.394 |
| Diabetes mellitus | 15 | 1.226 | 0.367 | 4.091 | 0.740 |
| Hematological disease | 2 | 0.000 | 0.000 |  | 0.999 |
| Others | 17 | 0.685 | 0.186 | 2.517 | 0.568 |
| **Onset symptoms** | | | | | |
| Sudden-onset, severe headache | 147 | 0.557 | 0.207 | 1.495 | 0.245 |
| Vomiting | 102 | 0.691 | 0.335 | 1.426 | 0.318 |
| Neck pain or stiffness | 66 | 0.829 | 0.395 | 1.743 | 0.621 |
| Photophobia | 6 | 0.653 | 0.074 | 5.759 | 0.701 |
| Blurred or double vision | 4 | 1.105 | 0.112 | 10.937 | 0.932 |
| Brief loss of consciousness | 70 | 4.500 | 2.079 | 9.741 | <0.001 |
| Seizures | 9 | 0.942 | 0.188 | 4.732 | 0.942 |
| Other | 132 | 4.125 | 1.191 | 14.289 | 0.025 |
| **Clinical presentation on admission** | | | | | |
| GCS score | 168 | 0.688 | 0.610 | 0.776 | <0.001 |
| GCS score: |  |  |  |  |  |
| Mild (13 - 15) | 100 | - | - | - | - |
| Moderate (9 - 12) | 29 | 7.050 | 2.254 | 22.048 | 0.001 |
| Severe (3 - 8) | 39 | 25.067 | 8.794 | 71.453 | <0.001 |
| Heart rate (beats/min) | 167 | 1.026 | 1.004 | 1.048 | 0.021 |
| Heart rate (beats/min): |  |  |  |  |  |
| ≤ 95 | 126 | - | - | - | - |
| > 95 | 41 | 2.866 | 1.323 | 6.211 | 0.008 |
| Respiratory rate (breaths/min) | 159 | 1.056 | 0.962 | 1.158 | 0.252 |
| Respiratory rate (breaths/min): |  |  |  |  |  |
| 12 - 25 | 146 | - | - | - | - |
| > 25 | 13 | 3.612 | 1.126 | 11.588 | 0.031 |
| Systolic blood pressure (mmHg) | 167 | 1.008 | 0.995 | 1.020 | 0.241 |
| Systolic blood pressure (mmHg): |  |  |  |  |  |
| < 140 | 83 | - | - | - | - |
| ≥ 140 | 84 | 1.378 | 0.669 | 2.835 | 0.384 |
| Diastolic blood pressure (mmHg) | 167 | 1.028 | 1.001 | 1.056 | 0.042 |
| Diastolic blood pressure (mmHg): |  |  |  |  |  |
| < 90 | 117 | - | - | - | - |
| ≥ 90 | 50 | 2.571 | 1.220 | 5.422 | 0.013 |
| Body temperature (^o^C) | 167 | 1.265 | 0.576 | 2.781 | 0.558 |
| Body temperature (^o^C): |  |  |  |  |  |
| < 38 | 160 | - | - | - | - |
| ≥ 38 | 7 | 1.330 | 0.248 | 7.138 | 0.740 |
| Focal neurological deficits | 99 | 2.079 | 0954 | 4.531 | 0.065 |
| Focal neurologic signs: |  |  |  |  |  |
| Third nerve palsy | 4 | 0.000 | 0.000 |  | 0.999 |
| Sixth nerve palsy | 1 | 0.000 | 0.000 |  | >0.999 |
| Hemiparesis | 33 | 1.441 | 0.580 | 3.581 | 0.431 |
| Aphasia | 6 | 1.288 | 0.222 | 7.465 | 0.777 |
| Bilateral leg weakness | 1 | 0.000 | 0.000 |  | >0.999 |
| Impaired level of consciousness | 49 | 5.975 | 2.150 | 16.603 | 0.001 |
| Brainstem signs | 3 | 5.385 | 0.468 | 61.920 | 0.177 |
| Neck stiffness | 52 | 0.581 | 0.240 | 1.406 | 0.229 |
| **Head imaging findings on admission** | | | | | |
| Non-contrast head computed tomography (CT) findings | | | | | |
| Detection of blood within the subarachnoid space | 167 | 492214819.4 | 0.000 |  | >0.999 |
| Location of blood within the subarachnoid space: |  |  |  |  |  |
| Basal cistern | 79 | 2.450 | 1.146 | 5.241 | 0.210 |
| Sylvian fissure | 157 | 0.694 | 0.171 | 2.833 | 0.610 |
| Interhemispheric fissure | 84 | 2.249 | 1.057 | 4.785 | 0.035 |
| Interpeduncular fossa | 83 | 3.156 | 1.442 | 6.907 | 0.004 |
| Suprasellar cistern | 90 | 1.610 | 0.765 | 3.391 | 0.210 |
| Ambient cistern | 81 | 3.364 | 1.536 | 7.367 | 0.002 |
| Quadrigeminal cistern | 27 | 5.160 | 2.154 | 12.363 | <0.001 |
| Maximum thickness of subarachnoid blood (mm) | 165 | 1.084 | 1.003 | 1.172 | 0.042 |
| IVH | 107 | 3.291 | 1.352 | 8.011 | 0.009 |
| Location of blood within the ventricular system: |  |  |  |  |  |
| Right lateral ventricle | 95 | 4.844 | 0.593 | 39.551 | 0.141 |
| Left lateral ventricle | 99 | 771570288.3 | 0.000 |  | 0.999 |
| Third ventricle | 42 | 9.333 | 3.572 | 24.384 | <0.001 |
| Fourth ventricle | 43 | 3.045 | 1.292 | 7.174 | 0.011 |
| Graeb score | 102 | 1.288 | 1.092 | 1.519 | 0.003 |
| Graeb score: |  |  |  |  |  |
| Mild (1 - 4) | 69 | - | - | - | - |
| Moderate (5 - 8) | 24 | 7.917 | 2.813 | 22.278 | <0.001 |
| Severe (9 - 12) | 9 | 3.800 | 0.887 | 16.276 | 0.072 |
| ICH | 41 | 2.476 | 1.140 | 5.376 | 0.022 |
| ICH volume (mL) | 41 | 1.028 | 1.002 | 1.055 | 0.032 |
| ICH volume (mL): |  |  |  |  |  |
| < 30 | 26 | - | - | - | - |
| 30 - 60 | 7 | 2.036 | 0.361 | 11.479 | 0.421 |
| > 60 | 8 | 4.524 | 0.849 | 24.109 | 0.077 |
| Subdural hemorrhage | 10 | 2.343 | 0.626 | 8.768 | 0.206 |
| Hydrocephalus | 72 | 2.700 | 1.292 | 5.644 | 0.008 |
| Evans' index | 145 | 55.345 | 0.174 | 17593.32 | 0.172 |
| Bicaudate index | 145 | 3379.058 | 8.878 | 1286100 | 0.007 |
| Relative bicaudate index | 145 | 4.346 | 1.411 | 13.383 | 0.010 |
| Hypodense lesions on computed tomography | 8 | 3.000 | 0.227 | 39.608 | 0.404 |
| Patterns of infarction: |  |  |  |  |  |
| Single cortical infarcts | 14 | 1.400 | 0.413 | 4.745 | 0.589 |
| Multiple widespread infarcts | 6 | 0.333 | 0.025 | 4.401 | 0.404 |
| Multislice computed tomography (MSCT) angiography / Digital subtraction angiography (DSA) findings | | | | | |
| Number of aneurysm | 168 | 0.431 | 0.135 | 1.374 | 0.155 |
| Number of aneurysm: |  |  |  |  |  |
| Single aneurysm | 144 | - | - | - | - |
| Multiple aneurysms | 24 | 0.429 | 0.121 | 1.522 | 0.190 |
| Side of aneurysm: |  |  |  |  |  |
| Right | 58 | - | - | - | - |
| Left | 49 | 0.591 | 0.234 | 1.489 | 0.264 |
| Both | 60 | 0.799 | 0.348 | 1.833 | 0.596 |
| Groups of aneurysm site: |  |  |  |  |  |
| Anterior circulation aneurysm | 120 | 3.135 | 0.888 | 11.061 | 0.076 |
| Posterior circulation aneurysm | 33 | 0.277 | 0.079 | 0.974 | 0.045 |
| Aneurysm site: |  |  |  |  |  |
| Internal carotid artery (ICA) | 35 | 0.975 | 0.402 | 2.363 | 0.955 |
| Ophtalmic segment of the ICA (OphIC) | 1 | 0.000 | 0.000 |  | >0.999 |
| Cavernous segment of the ICA (cIC) | 7 | 1.341 | 0.250 | 7.196 | 0.732 |
| Posterior communicating artery (PCoA) | 25 | 0.588 | 0.189 | 1.829 | 0.359 |
| Anterior cerebral artery (ACA) | 16 | 0.744 | 0.201 | 2.756 | 0.658 |
| Anterior communicating artery (AcoA) | 49 | 1.105 | 0.507 | 2.410 | 0.802 |
| Middle cerebral artery (MCA) | 34 | 1.022 | 0.421 | 2.485 | 0.961 |
| Vertebral artery (VA) | 7 | 1.341 | 0.250 | 7.196 | 0.732 |
| Posterior inferior cerebellar artery (PICA) | 4 | 1.105 | 0.112 | 10.937 | 0.932 |
| Basilar artery (BA) | 7 | 0.000 | 0.000 |  | 0.999 |
| Other | 129 | 4.645 | 1.346 | 16.036 | 0.015 |
| Maximum aneurysm size (mm): |  |  |  |  |  |
| Maximum dome width | 166 | 1.047 | 0.922 | 1.188 | 0.483 |
| Dome height | 163 | 1.012 | 0.957 | 1.070 | 0.682 |
| Maximum neck width | 158 | 1.033 | 0.922 | 1.159 | 0.574 |
| Dome-to-neck ratio | 157 | 0.975 | 0.846 | 1.125 | 0.731 |
| Shape of aneurysm: |  |  |  |  |  |
| Regular | 18 | - | - | - | - |
| Irregular | 117 | 1.882 | 0.511 | 6.938 | 0.342 |
| Multilobular | 11 | 1.875 | 0.305 | 11.524 | 0.497 |
| Two domes |  |  |  |  |  |
| Other | 3 | 2.500 | 0.168 | 37.260 | 0.506 |
| Vasospasm of the parent artery | 14 | 3.750 | 1.226 | 11.469 | 0.020 |
| **Severity of aneurysmal subarachnoid hemorrhage on admission** | | | | | |
| PAASH score | 167 | 3.358 | 2.272 | 4.962 | <0.001 |
| PAASH scale: |  |  |  |  |  |
| Grade I | 80 | - | - | - | - |
| Grade II | 31 | 4.936 | 1.103 | 22.095 | 0.037 |
| Grade III | 28 | 16.608 | 4.176 | 66.042 | <0.001 |
| Grade IV | 25 | 66.000 | 15.535 | 280.503 | <0.001 |
| Grade V | 4 | 25.667 | 2.641 | 249.475 | 0.005 |
| WFNS score | 168 | 2.672 | 1.898 | 3.763 | <0.001 |
| WFNS scale: |  |  |  |  |  |
| Grade I | 80 | - | - | - | - |
| Grade II | 14 | 4.278 | 0.646 | 28.310 | 0.132 |
| Grade III | 6 | 5.133 | 0.449 | 58.732 | 0.188 |
| Grade IV | 50 | 17.111 | 4.735 | 61.833 | <0.001 |
| Grade V | 18 | 66.733 | 14.202 | 313.567 | <0.001 |
| Modified WFNS score | 168 | 2.672 | 1.898 | 3.763 | <0.001 |
| Modified WFNS scale: |  |  |  |  |  |
| Grade I | 80 | - | - | - | - |
| Grade II | 14 | 4.278 | 0.646 | 28.310 | 0.132 |
| Grade III | 6 | 5.133 | 0.449 | 58.732 | 0.188 |
| Grade IV | 50 | 17.111 | 4.735 | 61.833 | <0.001 |
| Grade V | 18 | 66.733 | 14.202 | 313.567 | <0.001 |
| Hunt and Hess score | 168 | 2.978 | 20.75 | 4.274 | <0.001 |
| Hunt and Hess scale: |  |  |  |  |  |
| Grade 1 | 39 | - | - | - | - |
| Grade 2 | 45 | 1.767 | 0.154 | 20.274 | 0.647 |
| Grade 3 | 25 | 9.500 | 1.038 | 86.968 | 0.046 |
| Grade 4 | 21 | 19.000 | 2.141 | 168.594 | 0.008 |
| Grade 5 | 38 | 65.143 | 8.040 | 527.797 | <0.001 |
| Fisher score | 168 | 3.905 | 1.447 | 10.529 | 0.007 |
| Fisher scale: |  |  |  |  |  |
| Group 2 | 13 | - | - | - | - |
| Group 1 | 1 | 1.000 | 0.000 | - | >0.999 |
| Group 3 | 34 | 215396752.4 | 0.000 | - | 0.999 |
| Group 4 | 120 | 665195853.1 | 0.000 | - | 0.999 |
| Claassen score | 168 | 2.275 | 1.316 | 3.930 | 0.003 |
| Claassen scale: |  |  |  |  |  |
| Grade 1 |  |  |  |  |  |
| Grade 2 | 22 | - | - | - | - |
| Grade 3 | 11 | 0.000 | 0.000 |  | 0.999 |
| Grade 4 | 39 | 1.471 | 0.261 | 8.298 | 0.662 |
| Grade 5 | 96 | 5.000 | 1.100 | 22.729 | 0.037 |
| **Laboratory investigations on admission** | | | | | |
| Complete blood count |  |  |  |  |  |
| Red blood cells (T/L) | 163 | 1.161 | 0.731 | 1.845 | 0.527 |
| Hemoglobin (g/L) | 163 | 1.011 | 0.991 | 1.032 | 0.296 |
| Hematocrit (L/L) | 163 | 9693.238 | 1.455 | 64597546.05 | 0.041 |
| Platelets (G/L) | 163 | 1.001 | 0.996 | 1.005 | 0.823 |
| White blood cells (G/L) | 163 | 1.105 | 1.022 | 1.194 | 0.012 |
| Percentage of neutrophils (%) | 162 | 1.019 | 1.000 | 1.037 | 0.049 |
| Coagulation |  |  |  |  |  |
| Prothrombin time (PT) | 155 | 1.011 | 0.996 | 1.026 | 0.137 |
| Prothrombin time with INR (PT-INR) | 155 | 10.704 | 0.179 | 640.278 | 0.256 |
| Activated partial thromboplastin time (APTT) | 153 | 1.003 | 0.989 | 1.017 | 0.693 |
| rAPTT | 152 | 0.973 | 0.846 | 1.119 | 0.697 |
| Blood biochemical investigation |  |  |  |  |  |
| Ure (mmol/L) | 166 | 1.156 | 0.954 | 1.401 | 0.139 |
| Glucose (mmol/L) | 156 | 1.521 | 1.274 | 1.817 | <0.001 |
| Glucose (mmol/L): |  |  |  |  |  |
| ≤ 6.4 | 29 | - | - | - | - |
| > 6.4 | 127 | 664139864.7 | 0.000 |  | 0.998 |
| Creatinine (µmol/L) | 167 | 1.017 | 1.002 | 1.023 | 0.025 |
| SGOT (UI/L) | 157 | 1.058 | 1.031 | 1.086 | <0.001 |
| SGPT (UI/L) | 165 | 1.034 | 1.014 | 1.055 | 0.001 |
| Na^+^ (mmol/L) | 167 | 1.097 | 1.013 | 1.188 | 0.022 |
| Na^+^ (mmol/L): |  |  |  |  |  |
| ≥ 135 | 128 | - | - | - | - |
| < 135 | 39 | 0.407 | 0.147 | 1.125 | 0.083 |
| K^+^ (mmol/L) | 167 | 0.920 | 0.421 | 2.010 | 0.834 |
| Cl^-^ (mmol/L) | 167 | 1.023 | 0.967 | 1.083 | 0.426 |
| **Aneurysm repairs and other treatments** | | | | | |
| No aneurysm repair | 26 | 40.441 | 112.431 | 131.570 | <0.001 |
| Endovascular coiling | 79 | 0.211 | 0.090 | 0.494 | <0.001 |
| Number of coils | 59 | 0.687 | 0.435 | 1.086 | 0.108 |
| Stent-assisted coiling | 2 | 0.999 | 0.000 | 0.000 | 0.999 |
| Balloon-assisted coiling | 9 | 0.000 | 0.000 |  | 0.999 |
| Surgical clipping | 63 | 0.417 | 0.183 | 0.949 | 0.037 |
| Number of clip attempts | 57 | 1.097 | 0.691 | 1.742 | 0.695 |
| Temporary vessel occlusion of the parent artery | 7 | 1.133 | 0.112 | 11.484 | 0.916 |
| Surgical hematoma evacuation or decompressive craniotomy | 7 | 4.800 | 1.026 | 22.459 | 0.046 |
| External ventricular drainage | 26 | 1.577 | 626 | 3.970 | 0.334 |
| Number of EVD | 26 | 0.000 | 0.000 |  | 0.999 |
| Intraventricular fibrinolysis | 3 | 1.671 | 0.147 | 18.937 | 0.678 |
| **Airway management and mechanical ventilation** | | | | | |
| Tracheal intubation | 144 | 600033778.5 | 0.000 |  | 0.999 |
| Mechanical ventilation | 141 | 617682023.6 | 0.000 |  | 0.999 |
| Tracheotomy | 15 | 1.198 | 0.358 | 4.005 | 0.769 |
| **Medical treatment** | | | | | |
| Nimodipine for preventing and treating cerebral vasospasm | 158 | 0.219 | 0.056 | 0.862 | 0.030 |
| Route of administration of nimodipine: |  |  |  |  |  |
| Oral | 113 | 2.762 | 0.995 | 7.667 | 0.051 |
| Intravenous | 60 | 0.426 | 0.179 | 1.016 | 0.054 |
| Deep venous thrombosis prophylaxis | 16 | 1.073 | 0.323 | 3.559 | 0.908 |
| **Complications** | | | | | |
| Rebleeding | 10 | 3.968 | 1.081 | 14.569 | 0.038 |
| Number of rebleeding sites: |  |  |  |  |  |
| Rebleeding from a single site | 4 | - | - | - | - |
| Rebleeding from multiple sites | 6 | 0.167 | 0.010 | 2.821 | 0.214 |
| Rebleeding sites: |  |  |  |  |  |
| Subarachnoid hemorrhage, (n=9) | 3 | 2.000 | 0.112 | 35.807 | 0.638 |
| Subdural hemorrhage | 4 | 0.000 | 0.000 |  | 0.999 |
| Intraventricular hemorrhage | 6 | 1.000 | 0.080 | 12.557 | >0.999 |
| Intracerebral hemorrhage | 7 | 0.375 | 0.022 | 6.348 | 0.497 |
| Vasospasm and delayed cerebral ischemia | 17 | 1.943 | 0.667 | 5.661 | 0.223 |
| Patterns of vasospasm and delayed cerebral ischemia: |  |  |  |  |  |
| Single cortical infarcts | 9 | - | - | - | - |
| Multiple widespread infarcts | 7 | 2.625 | 0.300 | 22.998 | 0.383 |
| Acute hydrocephalus | 76 | 2.731 | 1.299 | 5.744 | 0.008 |
| Hyponatremia | 34 | 1.022 | 0.421 | 2.486 | 0.961 |
| Seizures | 44 | 0.254 | 0.085 | 0.764 | 0.015 |
| Chronic hydrocephalus | 5 | 3.650 | 0.341 | 39.094 | 0.285 |
| EVD obstruction | 2 | 0.000 | 0.000 |  | 0.999 |
| EVD replacement | 2 | 0.000 | 0.000 |  | 0.999 |
| Ventriculitis | 8 | 0.368 | 0.044 | 3.107 | 0.358 |
| Pneumonia | 29 | 1.327 | 0.536 | 3.287 | 0.541 |
| Urinary tract infection | 3 | 0.000 | 0.000 |  | 0.999 |
| **Clinical time course** | | | | | |
| Ictus to hospital arrival (hours): |  |  |  |  |  |
| ≤ 24 hours | 69 | - | - | - | - |
| >24–72 hours | 91 | 0.604 | 0.290 | 1.257 | 0.177 |
| >72 hours | 1 | 0.000 | 0.000 |  | >0.999 |
| EVD duration (days) | 25 | 0.857 | 0.657 | 1.116 | 0.252 |
| EVD duration (days): |  |  |  |  |  |
| ≤ 7 | 17 | - | - | - | - |
| > 7 | 8 | 0.262 | 0.026 | 2.664 | 0.258 |
| Length of hospitalization (days) | 168 | 0.918 | 0.866 | 0.974 | 0.005 |

**S5 Table**. Factors associated with death within 90 days after the onset of hemorrhage in patients with aneurysmal subarachnoid hemorrhage: multivariate logistic regression analyses (backward elimination)

| Steps | Factors | Unit | OR | 95% CI for OR | | p value |
| --- | --- | --- | --- | --- | --- | --- |
|  |  |  |  | Lower | Upper |  |
| 1 | **Demographics** | | | | | |
|  | Age (years): |  |  |  |  |  |
|  | 20 - 39 | % | - | - | - | 0.301 |
|  | 40 - 59 | % | 7.133 | 0.350 | 145.309 | 0.201 |
|  | ≥ 60 | % | 2.357 | 0.136 | 40.974 | 0.556 |
|  | **Risk factors of aneurysmal subarachnoid hemorrhage** | | | | | |
|  | Hypertension | % | 5.531 | 0.984 | 31.102 | 0.052 |
|  | **Head imaging findings on admission** | | | | | |
|  | Location of blood within the subarachnoid space: |  |  |  |  |  |
|  | Interhemispheric fissure | % | 1.168 | 0.250 | 5.446 | 0.843 |
|  | Interpeduncular fossa | % | 1.006 | 0.129 | 7.861 | 0.995 |
|  | Ambient cistern | % | 0.943 | 0.142 | 6.253 | 0.952 |
|  | Quadrigeminal cistern | % | 3.152 | 0.362 | 27.421 | 0.298 |
|  | IVH | % | 0.352 | 0.058 | 2.154 | 0.259 |
|  | ICH | % | 0.852 | 0.133 | 5.440 | 0.866 |
|  | **Severity of aneurysmal subarachnoid hemorrhage on admission** | | | | | |
|  | WFNS scale: |  |  |  |  |  |
|  | Grade I | % | - | - | - | 0.073 |
|  | Grade II | % | 3.905 | 0.211 | 72.230 | 0.360 |
|  | Grade III | % | 15.458 | 0.426 | 560.484 | 0.135 |
|  | Grade IV | % | 19.703 | 2.246 | 172.846 | 0.007 |
|  | Grade V | % | 37.908 | 1.922 | 747.665 | 0.017 |
|  | **Aneurysm repairs and other treatments** | | | | | |
|  | Aneurysm repairs: |  |  |  |  |  |
|  | No aneurysm repair | % | - | - | - | <0.001 |
|  | Endovascular coiling | % | 0.010 | 0.001 | 0.094 | <0.001 |
|  | Surgical clipping | % | 0.012 | 0.001 | 0.118 | <0.001 |
|  | External ventricular drainage | % | 0.992 | 0.157 | 6.284 | 0.993 |
|  | **Medical treatment** | | | | | |
|  | Nimodipine for preventing and treating cerebral vasospasm | % | 4.152 | 0.150 | 115.026 | 0.401 |
|  | **Complications** | | | | | |
|  | Rebleeding | % | 15.249 | 1.739 | 133.702 | 0.014 |
|  | Vasospasm and delayed cerebral ischemia | % | 2.510 | 0.367 | 17.167 | 0.348 |
|  | Acute hydrocephalus | % | 2.415 | 0.421 | 13.862 | 0.323 |
|  | Constant | % | 0.022 |  |  | 0.076 |
| 2 | **Demographics** | | | | | |
|  | Age (years): |  |  |  |  |  |
|  | 20 - 39 | % | - | - | - | 0.294 |
|  | 40 - 59 | % | 7.143 | 0.364 | 140.235 | 0.196 |
|  | ≥ 60 | % | 2.360 | 0.139 | 40.055 | 0.552 |
|  | **Risk factors of aneurysmal subarachnoid hemorrhage** | | | | | |
|  | Hypertension | % | 5.529 | 0.986 | 31.003 | 0.052 |
|  | **Head imaging findings on admission** | | | | | |
|  | Location of blood within the subarachnoid space: |  |  |  |  |  |
|  | Interhemispheric fissure | % | 1.169 | 0.257 | 5.322 | 0.840 |
|  | Ambient cistern | % | 0.947 | 0.208 | 4.299 | 0.943 |
|  | Quadrigeminal cistern | % | 3.156 | 0.380 | 26.249 | 0.288 |
|  | IVH | % | 0.352 | 0.060 | 2.078 | 0.249 |
|  | ICH | % | 0.851 | 0.136 | 5.327 | 0.863 |
|  | **Severity of aneurysmal subarachnoid hemorrhage on admission** | | | | | |
|  | WFNS scale: |  |  |  |  |  |
|  | Grade I | % | - | - | - | 0.073 |
|  | Grade II | % | 3.903 | 0.212 | 71.820 | 0.359 |
|  | Grade III | % | 15.452 | 0.427 | 558.734 | 0.135 |
|  | Grade IV | % | 19.707 | 2.248 | 172.744 | 0.007 |
|  | Grade V | % | 37.903 | 1.923 | 747.250 | 0.017 |
|  | **Aneurysm repairs and other treatments** | | | | | |
|  | Aneurysm repairs: |  |  |  |  |  |
|  | No aneurysm repair | % | - | - | - | <0.001 |
|  | Endovascular coiling | % | 0.010 | 0.001 | 0.094 | <0.001 |
|  | Surgical clipping | % | 0.012 | 0.001 | 0.115 | <0.001 |
|  | External ventricular drainage | % | 0.992 | 0.157 | 6.262 | 0.993 |
|  | **Medical treatment** | | | | | |
|  | Nimodipine for preventing and treating cerebral vasospasm | % | 4.152 | 0.150 | 115.044 | 0.401 |
|  | **Complications** | | | | | |
|  | Rebleeding | % | 15.269 | 1.812 | 128.629 | 0.012 |
|  | Vasospasm and delayed cerebral ischemia | % | 2.512 | 0.372 | 16.956 | 0.345 |
|  | Acute hydrocephalus | % | 2.414 | 0.422 | 13.807 | 0.322 |
|  | Constant | % | 0.022 |  |  | 0.075 |
| 3 | **Demographics** | | | | | |
|  | Age (years): |  |  |  |  |  |
|  | 20 - 39 | % | - | - | - | 0.260 |
|  | 40 - 59 | % | 7.123 | 0.388 | 130.874 | 0.186 |
|  | ≥ 60 | % | 2.359 | 0.139 | 39.940 | 0.552 |
|  | **Risk factors of aneurysmal subarachnoid hemorrhage** | | | | | |
|  | Hypertension | % | 5.515 | 1.088 | 27.967 | 0.039 |
|  | **Head imaging findings on admission** | | | | | |
|  | Location of blood within the subarachnoid space: |  |  |  |  |  |
|  | Interhemispheric fissure | % | 1.170 | 0.259 | 5.291 | 0.839 |
|  | Ambient cistern | % | 0.945 | 0.213 | 4.195 | 0.941 |
|  | Quadrigeminal cistern | % | 3.162 | 0.394 | 25.389 | 0.279 |
|  | IVH | % | 0.353 | 0.061 | 2.036 | 0.244 |
|  | ICH | % | 0.852 | 0.139 | 5.229 | 0.863 |
|  | **Severity of aneurysmal subarachnoid hemorrhage on admission** | | | | | |
|  | WFNS scale: |  |  |  |  |  |
|  | Grade I | % | - | - | - | 0.070 |
|  | Grade II | % | 3.906 | 0.213 | 71.616 | 0.359 |
|  | Grade III | % | 15.477 | 0.437 | 548.564 | 0.132 |
|  | Grade IV | % | 19.691 | 2.263 | 171.347 | 0.007 |
|  | Grade V | % | 37.846 | 1.957 | 731.908 | 0.016 |
|  | **Aneurysm repairs and other treatments** | | | | | |
|  | Aneurysm repairs: |  |  |  |  |  |
|  | No aneurysm repair | % | - | - | - | <0.001 |
|  | Endovascular coiling | % | 0.010 | 0.001 | 0.092 | <0.001 |
|  | Surgical clipping | % | 0.012 | 0.001 | 0.115 | <0.001 |
|  | **Medical treatment** | | | | | |
|  | Nimodipine for preventing and treating cerebral vasospasm | % | 4.152 | 0.150 | 114.912 | 0.401 |
|  | **Complications** | | | | | |
|  | Rebleeding | % | 15.229 | 1.965 | 117.998 | 0.009 |
|  | Vasospasm and delayed cerebral ischemia | % | 2.514 | 0.375 | 16.840 | 0.342 |
|  | Acute hydrocephalus | % | 2.408 | 0.461 | 12.570 | 0.297 |
|  | Constant | % | 0.022 |  |  | 0.074 |
| 4 | **Demographics** | | | | | |
|  | Age (years): |  |  |  |  |  |
|  | 20 - 39 | % | - | - | - | 0.256 |
|  | 40 - 59 | % | 7.233 | 0.403 | 129.815 | 0.179 |
|  | ≥ 60 | % | 2.398 | 0.146 | 39.447 | 0.541 |
|  | **Risk factors of aneurysmal subarachnoid hemorrhage** | | | | | |
|  | Hypertension | % | 5.477 | 1.092 | 27.478 | 0.039 |
|  | **Head imaging findings on admission** | | | | | |
|  | Location of blood within the subarachnoid space: |  |  |  |  |  |
|  | Interhemispheric fissure | % | 1.179 | 0.264 | 5.257 | 0.829 |
|  | Quadrigeminal cistern | % | 3.065 | 0.455 | 20.632 | 0.250 |
|  | IVH | % | 0.351 | 0.061 | 2.021 | 0.241 |
|  | ICH | % | 0.861 | 0.143 | 5.186 | 0.870 |
|  | **Severity of aneurysmal subarachnoid hemorrhage on admission** | | | | | |
|  | WFNS scale: |  |  |  |  |  |
|  | Grade I | % | - | - | - | 0.071 |
|  | Grade II | % | 3.940 | 0.218 | 71.299 | 0.353 |
|  | Grade III | % | 15.379 | 0.434 | 544.425 | 0.133 |
|  | Grade IV | % | 19.666 | 2.251 | 171.834 | 0.007 |
|  | Grade V | % | 37.669 | 1.940 | 731.498 | 0.016 |
|  | **Aneurysm repairs and other treatments** | | | | | |
|  | Aneurysm repairs: |  |  |  |  |  |
|  | No aneurysm repair | % | - | - | - | <0.001 |
|  | Endovascular coiling | % | 0.010 | 0.001 | 0.092 | <0.001 |
|  | Surgical clipping | % | 0.012 | 0.001 | 0.115 | <0.001 |
|  | **Medical treatment** | | | | | |
|  | Nimodipine for preventing and treating cerebral vasospasm | % | 4.145 | 0.149 | 115.437 | 0.402 |
|  | **Complications** | | | | | |
|  | Rebleeding | % | 15.160 | 1.960 | 117.286 | 0.009 |
|  | Vasospasm and delayed cerebral ischemia | % | 2.513 | 0.374 | 16.892 | 0.343 |
|  | Acute hydrocephalus | % | 2.396 | 0.461 | 12.440 | 0.299 |
|  | Constant | % | 0.021 |  |  | 0.064 |
| 5 | **Demographics** | | | | | |
|  | Age (years): |  |  |  |  |  |
|  | 20 - 39 | % | - | - | - | 0.253 |
|  | 40 - 59 | % | 6.820 | 0.425 | 109.451 | 0.175 |
|  | ≥ 60 | % | 2.297 | 0.150 | 35.059 | 0.550 |
|  | **Risk factors of aneurysmal subarachnoid hemorrhage** | | | | | |
|  | Hypertension | % | 5.366 | 1.097 | 26.243 | 0.038 |
|  | **Head imaging findings on admission** | | | | | |
|  | Location of blood within the subarachnoid space: |  |  |  |  |  |
|  | Interhemispheric fissure | % | 1.201 | 0.274 | 5.274 | 0.808 |
|  | Quadrigeminal cistern | % | 3.098 | 0.464 | 20.705 | 0.243 |
|  | IVH | % | 0.360 | 0.065 | 2.004 | 0.243 |
|  | **Severity of aneurysmal subarachnoid hemorrhage on admission** | | | | | |
|  | WFNS scale: |  |  |  |  |  |
|  | Grade I | % | - | - | - | 0.062 |
|  | Grade II | % | 3.788 | 0.218 | 65.794 | 0.361 |
|  | Grade III | % | 13.686 | 0.509 | 368.333 | 0.119 |
|  | Grade IV | % | 19.063 | 2.277 | 159.568 | 0.007 |
|  | Grade V | % | 35.786 | 1.998 | 641.063 | 0.015 |
|  | **Aneurysm repairs and other treatments** | | | | | |
|  | Aneurysm repairs: |  |  |  |  |  |
|  | No aneurysm repair | % | - | - | - | <0.001 |
|  | Endovascular coiling | % | 0.010 | 0.001 | 0.092 | <0.001 |
|  | Surgical clipping | % | 0.012 | 0.001 | 0.115 | <0.001 |
|  | **Medical treatment** | | | | | |
|  | Nimodipine for preventing and treating cerebral vasospasm | % | 4.171 | 0.147 | 117.926 | 0.402 |
|  | **Complications** | | | | | |
|  | Rebleeding | % | 15.192 | 1.962 | 117.623 | 0.009 |
|  | Vasospasm and delayed cerebral ischemia | % | 2.531 | 0.380 | 16.849 | 0.337 |
|  | Acute hydrocephalus | % | 2.384 | 0.463 | 12.284 | 0.299 |
|  | Constant | % | 0.021 |  |  | 0.064 |
| 6 | **Demographics** | | | | | |
|  | Age (years): |  |  |  |  |  |
|  | 20 - 39 | % | - | - | - | 0.256 |
|  | 40 - 59 | % | 6.732 | 0.421 | 107.548 | 0.177 |
|  | ≥ 60 | % | 2.305 | 0.150 | 35.452 | 0.549 |
|  | **Risk factors of aneurysmal subarachnoid hemorrhage** | | | | | |
|  | Hypertension | % | 5.316 | 1.090 | 25.932 | 0.039 |
|  | **Head imaging findings on admission** | | | | | |
|  | Location of blood within the subarachnoid space: |  |  |  |  |  |
|  | Quadrigeminal cistern | % | 3.329 | 0.548 | 20.215 | 0.191 |
|  | IVH | % | 0.364 | 0.066 | 2.009 | 0.246 |
|  | **Severity of aneurysmal subarachnoid hemorrhage on admission** | | | | | |
|  | WFNS scale: |  |  |  |  |  |
|  | Grade I | % | - | - | - | 0.060 |
|  | Grade II | % | 4.033 | 0.242 | 67.328 | 0.332 |
|  | Grade III | % | 15.322 | 0.637 | 368.809 | 0.093 |
|  | Grade IV | % | 19.684 | 2.362 | 164.060 | 0.006 |
|  | Grade V | % | 35.219 | 1.993 | 622.505 | 0.015 |
|  | **Aneurysm repairs and other treatments** | | | | | |
|  | Aneurysm repairs: |  |  |  |  |  |
|  | No aneurysm repair | % | - | - | - | <0.001 |
|  | Endovascular coiling | % | 0.010 | 0.001 | 0.092 | <0.001 |
|  | Surgical clipping | % | 0.012 | 0.001 | 0.112 | <0.001 |
|  | **Medical treatment** | | | | | |
|  | Nimodipine for preventing and treating cerebral vasospasm | % | 3.937 | 0.147 | 105.119 | 0.413 |
|  | **Complications** | | | | | |
|  | Rebleeding | % | 15.201 | 1.967 | 117.490 | 0.009 |
|  | Vasospasm and delayed cerebral ischemia | % | 2.593 | 0.391 | 17.189 | 0.324 |
|  | Acute hydrocephalus | % | 2.505 | 0.511 | 12.274 | 0.258 |
|  | Constant | % | 0.023 |  |  | 0.065 |
| 7 | **Demographics** | | | | | |
|  | Age (years): |  |  |  |  |  |
|  | 20 - 39 | % | - | - | - | 0.234 |
|  | 40 - 59 | % | 7.356 | 0.449 | 120.578 | 0.162 |
|  | ≥ 60 | % | 2.478 | 0.156 | 39.383 | 0.520 |
|  | **Risk factors of aneurysmal subarachnoid hemorrhage** | | | | | |
|  | Hypertension | % | 4.643 | 0.996 | 21.641 | 0.051 |
|  | **Head imaging findings on admission** | | | | | |
|  | Location of blood within the subarachnoid space: |  |  |  |  |  |
|  | Quadrigeminal cistern | % | 3.396 | 0.568 | 20.306 | 0.180 |
|  | IVH | % | 0.404 | 0.074 | 2.192 | 0.293 |
|  | **Severity of aneurysmal subarachnoid hemorrhage on admission** | | | | | |
|  | WFNS scale: |  |  |  |  |  |
|  | Grade I | % | - | - | - | 0.071 |
|  | Grade II | % | 4.156 | 0.257 | 67.278 | 0.316 |
|  | Grade III | % | 14.280 | 0.596 | 342.059 | 0.101 |
|  | Grade IV | % | 18.753 | 2.304 | 152.621 | 0.006 |
|  | Grade V | % | 21.768 | 1.619 | 292.676 | 0.020 |
|  | **Aneurysm repairs and other treatments** | | | | | |
|  | Aneurysm repairs: |  |  |  |  |  |
|  | No aneurysm repair | % | - | - | - | <0.001 |
|  | Endovascular coiling | % | 0.013 | 0.002 | 0.100 | <0.001 |
|  | Surgical clipping | % | 0.015 | 0.002 | 0.120 | <0.001 |
|  | **Complications** | | | | | |
|  | Rebleeding | % | 16.395 | 2.131 | 126.109 | 0.007 |
|  | Vasospasm and delayed cerebral ischemia | % | 2.719 | 0.414 | 17.849 | 0.297 |
|  | Acute hydrocephalus | % | 2.436 | 0.505 | 11.745 | 0.267 |
|  | Constant | % | 0.069 |  |  | 0.083 |
| 8 | **Demographics** | | | | | |
|  | Age (years): |  |  |  |  |  |
|  | 20 - 39 | % | - | - | - | 0.208 |
|  | 40 - 59 | % | 8.545 | 0.505 | 144.440 | 0.137 |
|  | ≥ 60 | % | 2.832 | 0.178 | 45.138 | 0.461 |
|  | **Risk factors of aneurysmal subarachnoid hemorrhage** | | | | | |
|  | Hypertension | % | 5.382 | 1.197 | 24.202 | 0.028 |
|  | **Head imaging findings on admission** | | | | | |
|  | Location of blood within the subarachnoid space: |  |  |  |  |  |
|  | Quadrigeminal cistern | % | 3.440 | 0.569 | 20.815 | 0.179 |
|  | IVH | % | 0.343 | 0.065 | 1.808 | 0.207 |
|  | **Severity of aneurysmal subarachnoid hemorrhage on admission** | | | | | |
|  | WFNS scale: |  |  |  |  |  |
|  | Grade I | % | - | - | - | 0.061 |
|  | Grade II | % | 3.445 | 0.211 | 56.154 | 0.385 |
|  | Grade III | % | 10.598 | 0.504 | 222.946 | 0.129 |
|  | Grade IV | % | 19.147 | 2.509 | 146.108 | 0.004 |
|  | Grade V | % | 18.963 | 1.451 | 247.853 | 0.025 |
|  | **Aneurysm repairs and other treatments** | | | | | |
|  | Aneurysm repairs: |  |  |  |  |  |
|  | No aneurysm repair | % | - | - | - | <0.001 |
|  | Endovascular coiling | % | 0.013 | 0.002 | 0.097 | <0.001 |
|  | Surgical clipping | % | 0.016 | 0.002 | 0.125 | <0.001 |
|  | **Complications** | | | | | |
|  | Rebleeding | % | 18.892 | 2.622 | 136.102 | 0.004 |
|  | Acute hydrocephalus | % | 2.130 | 0.452 | 10.024 | 0.339 |
|  | Constant | % | 0.080 |  |  | 0.098 |
| 9 | **Demographics** | | | | | |
|  | Age (years): |  |  |  |  |  |
|  | 20 - 39 | % | - | - | - | 0.208 |
|  | 40 - 59 | % | 10.007 | 0.591 | 169.518 | 0.111 |
|  | ≥ 60 | % | 3.901 | 0.260 | 58.454 | 0.324 |
|  | **Risk factors of aneurysmal subarachnoid hemorrhage** | | | | | |
|  | Hypertension | % | 5.529 | 1.241 | 24.638 | 0.025 |
|  | **Head imaging findings on admission** | | | | | |
|  | Location of blood within the subarachnoid space: |  |  |  |  |  |
|  | Quadrigeminal cistern | % | 4.028 | 0.681 | 23.820 | 0.124 |
|  | IVH | % | 0.397 | 0.080 | 1.968 | 0.258 |
|  | **Severity of aneurysmal subarachnoid hemorrhage on admission** | | | | | |
|  | WFNS scale: |  |  |  |  |  |
|  | Grade I | % | - | - | - | 0.046 |
|  | Grade II | % | 3.712 | 0.221 | 62.236 | 0.362 |
|  | Grade III | % | 9.251 | 0.498 | 171.875 | 0.136 |
|  | Grade IV | % | 20.269 | 2.872 | 143.024 | 0.003 |
|  | Grade V | % | 18.527 | 1.439 | 238.571 | 0.025 |
|  | **Aneurysm repairs and other treatments** | | | | | |
|  | Aneurysm repairs: |  |  |  |  |  |
|  | No aneurysm repair | % | - | - | - | <0.001 |
|  | Endovascular coiling | % | 0.015 | 0.002 | 0.106 | <0.001 |
|  | Surgical clipping | % | 0.016 | 0.002 | 0.129 | <0.001 |
|  | **Complications** | | | | | |
|  | Rebleeding | % | 24.981 | 3.619 | 172.428 | 0.001 |
|  | Constant | % | 0.075 |  |  | 0.088 |
| 10 | **Demographics** | | | | | |
|  | Age (years): |  |  |  |  |  |
|  | 20 - 39 | % | - | - | - | 0.320 |
|  | 40 - 59 | % | 5.684 | 0.457 | 70.743 | 0.177 |
|  | ≥ 60 | % | 2.816 | 0.224 | 35.450 | 0.423 |
|  | **Risk factors of aneurysmal subarachnoid hemorrhage** | | | | | |
|  | Hypertension | % | 4.932 | 1.146 | 21.226 | 0.032 |
|  | **Head imaging findings on admission** | | | | | |
|  | Location of blood within the subarachnoid space: |  |  |  |  |  |
|  | Quadrigeminal cistern | % | 3.606 | 0.622 | 20.901 | 0.153 |
|  | **Severity of aneurysmal subarachnoid hemorrhage on admission** | | | | | |
|  | WFNS scale: |  |  |  |  |  |
|  | Grade I | % | - | - | - | 0.066 |
|  | Grade II | % | 3.939 | 0.252 | 61.528 | 0.328 |
|  | Grade III | % | 9.797 | 0.563 | 170.387 | 0.117 |
|  | Grade IV | % | 14.973 | 2.350 | 95.390 | 0.004 |
|  | Grade V | % | 15.731 | 1.314 | 188.363 | 0.030 |
|  | **Aneurysm repairs and other treatments** | | | | | |
|  | Aneurysm repairs: |  |  |  |  |  |
|  | No aneurysm repair | % | - | - | - | <0.001 |
|  | Endovascular coiling | % | 0.018 | 0.003 | 0.115 | <0.001 |
|  | Surgical clipping | % | 0.019 | 0.003 | 0.140 | <0.001 |
|  | **Complications** | | | | | |
|  | Rebleeding | % | 24.362 | 3.574 | 166.075 | 0.001 |
|  | Constant | % | 0.066 |  |  | 0.063 |
| 11 | **Risk factors of aneurysmal subarachnoid hemorrhage** | | | | | |
|  | Hypertension | % | 4.707 | 1.224 | 18.107 | 0.024 |
|  | **Head imaging findings on admission** | | | | | |
|  | Location of blood within the subarachnoid space: |  |  |  |  |  |
|  | Quadrigeminal cistern | % | 4.279 | 0.760 | 24.086 | 0.099 |
|  | **Severity of aneurysmal subarachnoid hemorrhage on admission** | | | | | |
|  | WFNS scale: |  |  |  |  |  |
|  | Grade I | % | - | - | - | 0.068 |
|  | Grade II | % | 4.632 | 0.317 | 67.657 | 0.263 |
|  | Grade III | % | 10.140 | 0.570 | 180.475 | 0.115 |
|  | Grade IV | % | 14.038 | 2.294 | 85.911 | 0.004 |
|  | Grade V | % | 14.021 | 1.299 | 151.332 | 0.030 |
|  | **Aneurysm repairs and other treatments** | | | | | |
|  | Aneurysm repairs: |  |  |  |  |  |
|  | No aneurysm repair | % | - | - | - | <0.001 |
|  | Endovascular coiling | % | 0.022 | 0.004 | 0.128 | <0.001 |
|  | Surgical clipping | % | 0.024 | 0.004 | 0.155 | <0.001 |
|  | **Complications** | | | | | |
|  | Rebleeding | % | 22.588 | 3.619 | 141.000 | 0.001 |
|  | Constant | % | 0.197 |  |  | 0.143 |

**S6 Table**. Demographic and baseline characteristics, management and outcomes of patients with aneurysmal subarachnoid hemorrhage according to neurologic function at 30 days after the onset of hemorrhage

|  | All cases  (n=168) | mRS of 0 to 3  (n=110) | mRS of 4 to 6  (n=58) | p-value |
| --- | --- | --- | --- | --- |
| Transferred from local hospitals, n (%) |  |  |  |  |
| Hospital taken to, n (%) |  |  |  | 0.082 |
| Viet Duc | 22 (13.1) | 19 (17.3) | 3 (5.2) |  |
| Bach Mai | 129 (76.8) | 81 (73.6) | 48 (82.8) |  |
| Hanoi Medical University | 17 (10.1) | 10 (9.1) | 7 (12.1) |  |
| **Demographics** | | | | |
| Age (year), median (IQR) | 57 (48-67) | 56 (46-65) | 63.5 (53-71.25) | <0.001 |
| Age (years), n (%) |  |  |  | 0.012 |
| 20 - 39 | 18 (10.7) | 15 (13.6) | 3 (5.2) |  |
| 40 - 59 | 74 (44.0) | 54 (49.1) | 20 (34.5) |  |
| ≥ 60 | 76 (45.2) | 41 (37.3) | 35 (60.3) |  |
| Gender (male), n (%) | 77 (45.8) | 50 (45.5) | 27 (46.6) | 0.892 |
| Resident regions, n (%) |  |  |  | 0.244 |
| Urban | 68 (40.5) | 41 (37.3) | 27 (46.6) |  |
| Rural | 100 (59.5) | 69 (62.7) | 31 (53.4) |  |
| **Socioeconomic status** | | | | |
| Health insurance, n (%) | 152 (90.5) | 103 (93.6) | 49 (84.5) | 0.055 |
| Health insurance rates, n (%) |  |  |  | 0.426 |
| 100% | 30 (19.7) | 19 (18.4) | 11 (22.4) |  |
| 95% | 23 (15.1) | 13 (12.6) | 10 (20.4) |  |
| 80% | 94 (61.8) | 66 (64.1) | 28 (57.1) |  |
| 60% | 1 (0.7) | 1 (1.0) | 0 (0.0) |  |
| 40% | 4 (2.6) | 4 (3.9) | 0 (0.0) |  |
| Occupations, n (%) |  |  |  | 0.056 |
| Managers | 2 (1.2) | 0 (0.0) | 2 (3.4) |  |
| Professionals | 3 (1.8) | 2 (1.8) | 1 (1.7) |  |
| Technicians and associate professionals | 3 (1.8) | 3 (2.9) | 0 (0.0) |  |
| Clerical support workers | 4 (2.4) | 3 (2.7) | 1 (1.7) |  |
| Service and sales workers | 15 (8.9) | 12 (10.9) | 3 (5.2) |  |
| Skilled agricultural, forestry and fishery workers | 36 (21.4) | 21 (19.1) | 15 (25.9) |  |
| Craft and related trades workers |  |  |  |  |
| Plant and machine operators, and assemblers | 6 (3.6) | 5 (4.5) | 1 (1.7) |  |
| Elementary occupations | 46 (27.4) | 35 (31.8) | 11 (19.0) |  |
| Armed forces occupations |  |  |  |  |
| Retired | 49 (29.2) | 26 (23.6) | 23 (39.7) |  |
| Student/unemployment | 1 (0.6) | 0 (0.0) | 1 (1.7) |  |
| Other | 3 (1.8) | 3 (2.7) | 0 (0.0) |  |
| Highest education levels, n (%) |  |  |  | 0.824 |
| School | 126 (75.0) | 83 (75.5) | 43 (74.1) |  |
| Vocational school | 12 (7.1) | 7 (6.4) | 5 (8.6) |  |
| Junior college | 8 (4.8) | 6 (5.5) | 2 (3.4) |  |
| Senior college | 15 (8.9) | 10 (9.1) | 5 (8.6) |  |
| College/University | 6 (3.6) | 4 (3.6) | 2 (3.4) |  |
| Postgraduate | 1 (0.6) | 0 (0.0) | 1 (1.7) |  |
| Annual income, n (%) |  |  |  | 0.990 |
| Upper-middle (US $4,036 - US $12,475) | 18 (10.8) | 12 (11.0) | 6 (10.3) |  |
| Lower-middle (US $1,026 - US $4,035) | 115 (68.9) | 75 (68.8) | 40 (69.0) |  |
| Low (≤ US $1,025) | 34 (20.4) | 22 (20.2) | 12 (20.7) |  |
| **Risk factors for aneurysmal subarachnoid hemorrhage** | | | | |
| Cigarette smoking, n (%) | 63 (37.5) | 41 (37.3) | 22 (37.9) | 0.933 |
| Smoking behaviors, n (%) |  |  |  | 0.867 |
| Quit | 18 (28.6) | 12 (29.3) | 6 (27.3) |  |
| Current | 45 (71.4) | 29 (70.7) | 16 (72.7) |  |
| Hypertension, n (%) | 64 (38.1) | 31 (28.2) | 33 (56.9) | 0.005 |
| Genetic risk, n (%) | 6 (3.6) | 5 (4.5) | 1 (1.7) | 0.666 |
| Inherited conditions, n (%) |  |  |  | 0.999 |
| Polycystic kidney disease | 1 (16.7) | 1 (20.0) | 0 (0.0) |  |
| A family history of subar-achnoid hemorrhage/unru-ptured aneurysms | 5 (83.3) | 4 (80.0) | 1 (100.0) |  |
| Alcohol consumption, n (%) | 81 (48.2) | 53 (48.2) | 28 (48.3) | 0.991 |
| Alcohol drinking behaviors, n (%) |  |  |  | 0.390 |
| Occasionally | 45 (55.6) | 27 (50.9) | 18 (64.3) |  |
| Sometimes | 25 (30.9) | 19 (35.8) | 6 (21.4) |  |
| Every day | 11 (13.6) | 7 (13.2) | 4 (14.3) |  |
| Sympathomimetic drugs, n (%) | 2 (1.2) | 2 (1.8) | 0 (0.0) | 0.544 |
| Estrogen deficiency, n (%) | 32/89 (36.0) | 19/58 (32.8) | 13/31 (41.9) | 0.390 |
| Antithrombotic therapy, n (%) | 3 (1.8) | 1 (0.9) | 2 (3.4) | 0.274 |
| Elevated total cholesterol, n (%) | 8 (4.8) | 5 (4.5) | 3 (5.2) | 0.999 |
| **Comorbidities** | | | | |
| Cerebrovascular disease, n (%) | 3 (1.8) | 2 (1.8) | 1 (1.7) | 0.999 |
| Chronic cardiac failure, n (%) | 3 (1.8) | 1 (0.9) | 2 (3.4) | 0.274 |
| Coronary artery disease/IM, n (%) | 3 (1.8) | 1 (0.9) | 2 (3.4) | 0.274 |
| COPD /Asthma, n (%) | 0 | 0 | 0 | - |
| Chronic pulmonary disease, n (%) | 0 | 0 | 0 | - |
| Tuberculosis, n (%) | 0 | 0 | 0 | - |
| Active neoplasm, n (%) | 3 (1.8) | 2 (1.8) | 1 (1.7) | 0.999 |
| Chronic renal failure, n (%) | 2 (1.2) | 1 (0.9) | 1 (1.7) | 0.999 |
| Ulcer disease, n (%) | 0 | 0 | 0 | - |
| Diabetes mellitus, n (%) | 15 (8.9) | 8 (7.3) | 7 (12.1) | 0.394 |
| Immunoincompetence, n (%) | 0 | 0 | 0 | - |
| Hematological disease, n (%) | 2 (1.2) | 1 (0.9) | 1 (1.7) | 0.999 |
| Others, n (%) | 17 (10.1) | 9 (8.2) | 8 (13.8) | 0.252 |
| **Onset symptoms** | | | | |
| Sudden-onset, severe headache, n (%) | 147 (87.5) | 102 (92.7) | 45 (77.6) | 0.005 |
| Vomiting, n (%) | 102 (60.7) | 71 (64.5) | 31 (53.4) | 0.161 |
| Neck pain or stiffness, n (%) | 66 (39.3) | 43 (39.1) | 23 (39.7) | 0.943 |
| Photophobia, n (%) | 6 (3.6) | 5 (4.5) | 1 (1.7) | 0.666 |
| Blurred or double vision, n (%) | 4 (2.4) | 3 (2.7) | 1 (1.7) | 0.999 |
| Brief loss of consciousness, n (%) | 70 (41.7) | 31 (28.2) | 39 (67.2) | <0.001 |
| Seizures, n (%) | 9 (5.4) | 6 (5.5) | 3 (5.2) | 0.999 |
| Other, n (%) | 132 (78.6) | 83 (75.5) | 49 (84.5) | 0.175 |
| **Clinical presentation on admission** | | | | |
| GCS score, median (IQR) | 14 (9-15) | 15 (13.75-15) | 8 (6.75-12) | <0.001 |
| GCS score, n (%) |  |  |  | <0.001 |
| Mild (13 - 15) | 100 (59.5) | 87 (79.1) | 13 (22.4) |  |
| Moderate (9 - 12) | 29 (17.3) | 16 (14.5) | 13 (22.4) |  |
| Severe (3 - 8) | 39 (23.2) | 7 (6.4) | 32 (55.2) |  |
| Heart rate (beats/min), median (IQR), n=167 | 85 (76-95) | 84 (76-90) | 90 (80-100) | 0.004 |
| Heart rate (beats/min), n (%) | n=167 | n=109 | n=58 | 0.011 |
| ≤ 95 | 126 (75.4) | 89 (81.7) | 37 (63.8) |  |
| > 95 | 41 (24.6) | 20 (18.3) | 21 (36.2) |  |
| Respiratory rate (breaths/min), median (IQR), (n=159) | 20 (18-21) | 20 (18-20) | 20 (18-25) | 0.371 |
| Respiratory rate (breaths/min), n (%) | n=159 | n=108 | n=51 | 0.028 |
| < 12 | 0 | 0 | 0 |  |
| 12 - 25 | 146 (91.8) | 103 (95.4) | 43 (84.3) |  |
| > 25 | 13 (8.2) | 5 (4.6) | 8 (15.7) |  |
| Systolic blood pressure (mmHg), mean (SD), n=167 | 137.40 (28.07) | 135.24 (21.37) | 141.45 (37.47) | 0.138 |
| Systolic blood pressure (mmHg), n (%) | n=167 | n=109 | n=58 | 0.117 |
| < 140 | 83 (49.7) | 59 (54.1) | 24 (41.4) |  |
| ≥ 140 | 84 (50.3) | 50 (45.9) | 34 (58.6) |  |
| Diastolic blood pressure (mmHg), mean (SD), n=167 | 79.90 (13.73) | 78.33 (10.91) | 82.84 (17.60) | 0.037 |
| Diastolic blood pressure (mmHg), n (%) | n=167 | n=109 | n=58 | 0.002 |
| < 90 | 117 (70.1) | 85 (78.0) | 32 (55.2) |  |
| ≥ 90 | 50 (29.9) | 24 (22.0) | 26 (44.8) |  |
| Body temperature (^o^C), mean (SD) | 36.93 (0.45) | 36.89 (0.42) | 37.00 (0.48) | 0.194 |
| Body temperature (^o^C), n (%) | n=167 | n=109 | n=58 | 0.050 |
| < 38 | 160 (95.8) | 107 (98.2) | 53 (91.4) |  |
| ≥ 38 | 7 (4.2) | 2 (1.8) | 5 (8.6) |  |
| Focal neurological deficits, n (%) | 99 (58.9) | 60 (54.5) | 39 (67.2) | 0.112 |
| Focal neurologic signs, n (%): |  |  |  |  |
| Third nerve palsy | 4 (4.0) | 3 (5.0) | 1 (2.6) | 0.999 |
| Sixth nerve palsy | 1 (1.0) | 1 (1.7) | 0 (0.0) | 0.999 |
| Hemiparesis | 33 (33.3) | 19 (31.7) | 14 (35.9) | 0.663 |
| Aphasia | 6 (6.1) | 3 (5.0) | 3 (7.7) | 0.678 |
| Bilateral leg weakness | 1 (1.0) | 1 (1.7) | 0 (0.0) | 0.999 |
| Ophthalmoplegia | 0 | 0 | 0 | - |
| Unilateral visual loss or bitemporal hemianopia | 0 | 0 | 0 | - |
| Impaired level of consciousness | 49 (49.5) | 20 (33.3) | 29 (74.4) | <0.001 |
| Brainstem signs | 3 (3.0) | 1 (1.7) | 2 (5.1) | 0.560 |
| Neck stiffness | 52 (52.5) | 36 (60.0) | 16 (41.0) | 0.065 |
| Retinal and subhyaloid hemorrhages | 0 | 0 | 0 | - |
| Preretinal hemorrhages (Terson syndrome) | 0 | 0 | 0 | - |
| Other | 0 | 0 | 0 | - |
| **Head imaging findings on admission** | | | | |
| Non-contrast head computed tomography (CT) findings | | | | |
| Detection of blood within the subarachnoid space, n (%) | 167 (99.4) | 109 (99.1) | 58 (100.0) | 0.999 |
| Location of blood within the subarachnoid space, n (%) | 79 (47.9) | 43 (39.4) | 36 (64.3) | 0.002 |
| Basal cistern | 157 (94.0) | 102 (93.6) | 55 (94.8) | 0.999 |
| Sylvian fissure | 84 (50.6) | 51 (46.8) | 33 (57.9) | 0.174 |
| Interhemispheric fissure | 83 (50.0) | 43 (39.4) | 40 (70.2) | <0.001 |
| Interpeduncular fossa | 90 (54.2) | 55 (50.5) | 35 (61.4) | 0.179 |
| Suprasellar cistern | 81 (48.8) | 42 (38.5) | 39 (68.4) | <0.001 |
| Ambient cistern | 27 (16.3) | 10 (9.2) | 17 (29.8) | 0.001 |
| Quadrigeminal cistern |  |  |  |  |
| Maximum thickness of subarachnoid blood (mm), mean (SD), n=165 | 6.30 (4.56) | 5.84 (4.52) | 7.19 (4.55) | 0.045 |
| IVH, n (%) | 107 (63.7) | 60 (54.5) | 47 (81.0) | 0.001 |
| Location of blood within the ventricular system, n (%) |  |  |  |  |
| Right lateral ventricle | 95 (89.6) | 50 (84.7) | 45 (95.7) | 0.107 |
| Left lateral ventricle | 99 (93.4) | 52 (88.1) | 47 (100.0) | 0.017 |
| Third ventricle | 42 (39.6) | 11 (18.6) | 31 (66.0) | <0.001 |
| Fourth ventricle | 43 (40.6) | 18 (30.5) | 25 (53.2) | 0.018 |
| Graeb score, median (IQR), n=102 | 3 (2-5.25) | 2 (2-4) | 5 (3-7.25) | <0.001 |
| Graeb score, n (%) |  |  |  | <0.001 |
| Mild (1 - 4) | 69 (67.7) | 51 (91.1) | 18 (39.1) |  |
| Moderate (5 - 8) | 24 (23.5) | 4 (7.1) | 20 (43.5) |  |
| Severe (9 - 12) | 9 (8.8) | 1 (1.8) | 8 (17.4) |  |
| ICH, n (%) | 41 (24.4) | 22 (20.0) | 19 (32.8) | 0.067 |
| ICH volume (mL), mean (SD) | 29.29 (28.21) | 21.62 (21.95) | 38.17 (32.43) | 0.117 |
| ICH volume (mL), n (%) |  |  |  | 0.233 |
| < 30 | 26 (63.4) | 16 (72.7) | 10 (52.6) |  |
| 30 - 60 | 7 (17.1) | 4 (18.2) | 3 (15.8) |  |
| > 60 | 8 (19.5) | 2 (9.1) | 6 (31.6) |  |
| Subdural hemorrhage, n (%) | 10 (6.0) | 5 (4.5) | 5 (8.6) | 0.316 |
| Hydrocephalus, n (%) | 72 (42.9) | 38 (34.5) | 34 (58.6) | 0.003 |
| Evans' index, mean (SD), n=145 | 0.30 (0.06) | 0.29 (0.05) | 0.32 (0.08) | 0.018 |
| Bicaudate index, mean (SD), n=145 | 0.20 (0.06) | 0.19 (0.05) | 0.22 (0.08) | 0.004 |
| Relative bicaudate index, mean (SD), n=145 | 1.07 (0.34) | 1.00 (0.27) | 1.18 (0.41) | 0.007 |
| Hypodense lesions on computed tomography, n (%), | 14 (8.4) | 5 (4.5) | 9 (15.8) | 0.018 |
| Patterns of infarction, n (%) |  |  |  |  |
| Single cortical infarcts | 6 (42.9) | 3 (60.0) | 3 (33.3) | 0.580 |
| Multiple widespread infarcts | 8 (57.1) | 2 (40.0) | 6 (66.7) | 0.580 |
| Multislice computed tomography (MSCT) angiography / Digital subtraction angiography (DSA) findings | | | | |
| Detection of intracranial aneurysm, n (%) | 168 (100) | 110 (100) | 58 (100) | - |
| Number of aneurysm, median (IQR) | 1 (1-1) | 1 (1-1) | 1 (1-1) | 0.323 |
| Number of aneurysm, n (%): |  |  |  | 0.289 |
| Single aneurysm | 144 (85.7) | 92 (83.6) | 52 (89.7) |  |
| Multiple aneurysms | 24 (14.3) | 18 (16.4) | 6 (10.3) |  |
| Side of aneurysm, n (%) |  |  |  | 0.554 |
| Right | 58 (34.7) | 36 (33.0) | 22 (37.9) |  |
| Left | 49 (29.3) | 35 (32.1) | 14 (24.1) |  |
| Both | 60 (35.9) | 38 (34.9) | 22 (37.9) |  |
| Groups of aneurysm site, n (%) |  |  |  |  |
| Anterior circulation aneurysm | 120 (80.0) | 77 (78.6) | 43 (82.7) | 0.548 |
| Posterior circulation aneurysm | 33 (22.0) | 24 (24.5) | 9 (17.3) | 0.312 |
| Aneurysm site, n (%) |  |  |  |  |
| Internal carotid artery (ICA) | 35 (20.8) | 23 (20.9) | 12 (20.7) | 0.973 |
| Ophtalmic segment of the ICA (OphIC) | 1 (0.6) | 1 (0.9) | 0 (0.0) | 0.999 |
| Cavernous segment of the ICA (cIC) | 7 (4.2) | 5 (4.5) | 2 (3.4) | >0.999 |
| Anterior choroidal artery segment of the ICA (AchIC) | 0 | 0 | 0 | - |
| Posterior communicating artery (PCoA) | 25 (14.9) | 15 (13.6) | 10 (17.2) | 0.532 |
| Anterior cerebral artery (ACA) | 16 (9.5) | 12 (10.9) | 4 (6.9) | 0.582 |
| Anterior communicating artery (AcoA) | 49 (29.2) | 30 (27.3) | 19 (32.8) | 0.457 |
| Middle cerebral artery (MCA) | 34 (20.2) | 23 (20.9) | 11 (19.0) | 0.766 |
| Posterior cerebral artery (PCA) | 0 | 0 | 0 | - |
| Vertebral artery (VA) | 7 (4.2) | 4 (3.6) | 3 (5.2) | 0.694 |
| Superior cerebellar artery (SCA) | 0 | 0 | 0 | - |
| Posterior inferior cerebellar artery (PICA) | 4 (2.4) | 3 (2.7) | 1 (1.7) | 0.999 |
| Anterior inferior cerebellar artery (AICA) | 0 | 0 | 0 | - |
| Basilar artery (BA) | 7 (4.2) | 7 (6.4) | 0 (0.0) | 0.097 |
| Other | 129 (76.8) | 81 (73.6) | 48 (82.8) | 0.249 |
| Maximum aneurysm size (mm), mean (SD) |  |  |  |  |
| Maximum dome width, n=166 | 4.75 (2.70) | 4.72 (2.65) | 4.81 (2.81) | 0.763 |
| Dome height, n=163 | 5.50 (5.79) | 4.94 (2.47) | 6.58 (9.23) | 0.553 |
| Maximum neck width, n=158 | 3.35 (2.83) | 3.00 (1.26) | 4.03 (4.46) | 0.030 |
| Dome-to-neck ration n=157 | 1.65 (3.71) | 1.30 (0.69) | 2.33 (6.23) | 0.067 |
| Shape of aneurysm, n (%) | n=149 | n=93 | n=56 | 0.505 |
| Regular | 18 (12.1) | 14 (15.1) | 4 (7.1) |  |
| Irregular | 117 (78.5) | 70 (75.3) | 47 (83.9) |  |
| Multilobular | 11 (7.4) | 7 (7.5) | 4 (7.1) |  |
| Other | 3 (2.0) | 2 (2.2) | 1 (1.8) |  |
| Vasospasm of the parent artery, n (%), n=166 | 14 (8.4) | 6 (5.5) | 8 (14.0) | 0.078 |
| **Severity of aneurysmal subarachnoid hemorrhage on admission** | | | | |
| PAASH score, median (IQR) | 2 (1-3) | 1 (1-2) | 3 (2-4) | <0.001 |
| PAASH scale, n (%) |  |  |  | <0.001 |
| Grade I | 80 (47.6) | 771 (64.5) | 9 (15.5) |  |
| Grade II | 31 (18.5) | 24 (21.8) | 7 (12.1) |  |
| Grade III | 28 (16.7) | 10 (9.1) | 18 (31.0) |  |
| Grade IV | 25 (14.9) | 4 (3.6) | 21 (36.2) |  |
| Grade V | 4 (2.4) | 1 (0.9) | 3 (5.2) |  |
| WFNS score, median (IQR) | 2 (1-4) | 1 (1-3) | 4 (4-5) | <0.001 |
| WFNS scale, n (%) |  |  |  | <0.001 |
| Grade I | 80 (47.6) | 71 (64.5) | 9 (15.5) |  |
| Grade II | 14 (8.3) | 11 (10.0) | 3 (5.2) |  |
| Grade III | 6 (3.6) | 5 (4.5) | 1 (1.7) |  |
| Grade IV | 50 (29.8) | 20 (18.2) | 30 (51.7) |  |
| Grade V | 18 (10.7) | 1 (2.7) | 15 (25.9) |  |
| Modified WFNS score, median (IQR) | 2 (1-4) | 1 (1-2.25) | 4 (4-5) | <0.001 |
| Modified WFNS scale, n (%) |  |  |  | <0.001 |
| Grade I | 80 (47.6) | 71 (64.5) | 9 (15.5) |  |
| Grade II | 14 (8.3) | 12 (10.9) | 2 (3.4) |  |
| Grade III | 6 (3.6) | 4 (3.6) | 2 (3.4) |  |
| Grade IV | 50 (29.8) | 20 (18.2) | 30 (51.7) |  |
| Grade V | 18 (10.7) | 3 (2.7) | 15 (25.9) |  |
| Hunt and Hess score, median (IQR) | 2.5 (2-4) | 2 (1-3) | 5 (3-5) | <0.001 |
| Hunt and Hess scale, n (%) |  |  |  | <0.001 |
| Grade 1 | 39 (23.2) | 34 (30.9) | 5 (8.6) |  |
| Grade 2 | 45 (26.8) | 40 (36.4) | 5 (8.6) |  |
| Grade 3 | 25 (14.9) | 18 (16.4) | 7 (12.1) |  |
| Grade 4 | 21 (12.5) | 12 (10.9) | 9 (15.5) |  |
| Grade 5 | 38 (22.6) | 6 (5.5) | 32 (55.2) |  |
| Fisher score, median (IQR) | 4 (3-4) | 4 (3-4) | 4 (4-4) | <0.001 |
| Fisher scale, n (%) |  |  |  | <0.001 |
| Group 1 | 1 (0.6) | 1 (0.9) | 0 (0.0) |  |
| Group 2 | 13 (7.7) | 12 (10.9) | 1 (1.7) |  |
| Group 3 | 34 (20.2) | 29 (26.4) | 5 (8.6) |  |
| Group 4 | 120 (71.4) | 68 (61.8) | 52 (89.7) |  |
| Claassen score, median (IQR) | 5 (4-5) | 4 (3.75-5) | 5 (5-5) | <0.001 |
| Claassen scale, n (%) |  |  |  | 0.002 |
| Grade 1 |  |  |  |  |
| Grade 2 | 22 (13.1) | 18 (16.4) | 4 (6.9) |  |
| Grade 3 | 11 (6.5) | 9 (8.2) | 2 (3.4) |  |
| Grade 4 | 39 (23.2) | 32 (29.1) | 7 (12.1) |  |
| Grade 5 | 96 (57.1) | 51 (46.4) | 45 (77.6) |  |
| **Laboratory investigations on admission** | | | | |
| Complete blood count |  |  |  |  |
| Red blood cells (T/L), mean (SD) | 4.46 (0.78) | 4.46 (0.83) | 4.46 (0.68) | 0.695 |
| Hemoglobin (g/L), mean (SD) | 132.07 (21.09) | 131.58 (19.09) | 132.98 (24.55) | 0.285 |
| Hematocrit (L/L), mean (SD) | 0.40 (0.04) | 0.39 (0.04) | 0.40 (0.05) | 0.138 |
| Platelets (G/L), mean (SD) | 247.21 (76.12) | 247.88 (72.73) | 245.98 (82.71) | 0.996 |
| White blood cells (G/L), mean (SD) | 19.30 (54.87) | 13.71 (9.06) | 29.70 (91.58) | <0.001 |
| Percentage of neutrophils (%), mean (SD) | 70.58 (28.72) | 67.66 (29.82) | 75.97 (25.96) | 0.007 |
| Coagulation |  |  |  |  |
| Prothrombin time (PT), mean (SD) | 91.95 (32.59) | 89.65 (35.52) | 96.62 (25.30) | 0.467 |
| Prothrombin time with INR (PT-INR), mean (SD) | 1.00 (0.09) | 1.00 (0.08) | 1.02 (0.11) | 0.886 |
| Activated partial thromboplastin time (APTT), mean (SD) | 44.21 (200.54) | 28.08 (4.50 ) | 76.45 (347.31) | 0.963 |
| rAPTT, mean (SD) | 1.55 (6.82) | 1.80 (8.36) | 1.05 (0.46) | 0.589 |
| Blood biochemical investigation |  |  |  |  |
| Ure (mmol/L), mean (SD) | 5.39 (1.80) | 5.12 (1.77) | 5.55 (1.77) | 0.004 |
| Glucose (mmol/L), mean (SD) | 8.21 (2.42) | 7.40 (1.84) | 9.68 (2.66) | <0.001 |
| Glucose (mmol/L), n (%) |  |  |  | <0.001 |
| ≤ 6.4 | 29 (18.6) | 27 (26.7) | 2 (3.6) |  |
| > 6.4 | 127 (81.4) | 74 (73.3) | 53 (96.4) |  |
| Creatinine (µmol/L), mean (SD) | 67.40 (25.40) | 63.44 (26.48) | 74.85 (21.53) | <0.001 |
| SGOT (UI/L), mean (SD) | 34.30 (26.01) | 27.10 (11.32) | 46.59 (37.25) | <0.001 |
| SGPT (UI/L), mean (SD) | 28.77 (24.01) | 24.24 (13.79) | 37.11 (34.59) | 0.007 |
| Na^+^ (mmol/L), mean (SD) | 136.62 (10.76) | 135.80 (12.61) | 138.14 (5.72) | 0.191 |
| Na^+^ (mmol/L), n (%) |  |  |  | 0.173 |
| ≥ 135 | 128 (76.6) | 80 (73.4) | 48 (82.8) |  |
| < 135 | 39 (23.4) | 29 (26.6) | 10 (17.2) |  |
| K^+^ (mmol/L), mean (SD) | 3.56 (0.46) | 3.58 (0.41) | 3.53 (0.56) | 0.461 |
| Cl^-^ (mmol/L), mean (SD) | 99.95 (8.89) | 100.50 (5.15) | 98.91 (13.36) | 0.292 |
| **Aneurysm repairs and other treatments** | | | | |
| No aneurysm repair, n (%) | 26 (15.5) | 3 (2.7) | 23 (39.7) | <0.001 |
| Endovascular coiling, n (%) | 79 (47.0) | 63 (57.3) | 16 (27.6) | <0.001 |
| Number of coils, median (IQR) | 5 (3-7) | 5 (4-7) | 4 (3-6) | 0.171 |
| Stent-assisted coiling, n (%) | 2 (2.6) | 2 (3.2) | 0 (0.0) | 0.999 |
| Balloon-assisted coiling, n (%) | 9 (11.7) | 9 (14.8) | 0 (0.0) | 0.191 |
| Surgical clipping, n (%) | 63 (37.5) | 44 (40.0) | 19 (32.8) | 0.357 |
| Number of clip attempts, median (IQR) | 4 (2-5) | 3 (1-4) | 4 (3-5) | 0.078 |
| Temporary vessel occlusion of the parent artery, n (%) | 7 (15.2) | 5 (13.9) | 2 (20.0) | 0.636 |
| Surgical hematoma evacuation or decompressive craniotomy, n (%) | 7 (4.2) | 1 (0.9) | 6 (10.3) | 0.007 |
| External ventricular drainage, n (%) | 26 (15.6) | 9 (8.3) | 17 (29.3) | <0.001 |
| Number of EVD, median (IQR) | 1 (1-1) | 1 (1-1) | 1 (1-1) | 0.294 |
| Intraventricular fibrinolysis, n (%) | 3 (1.8) | 1 (0.9) | 2 (3.4) | 0.274 |
| **Airway management and mechanical ventilation** | | | | |
| Tracheal intubation, n (%) | 144 (95.4) | 86 (92.5) | 58 (100.0) | 0.044 |
| Mechanical ventilation, n (%) | 141 (94.6) | 83 (91.2) | 58 (100.0) | 0.023 |
| Tracheotomy, n (%) | 15 (9.3) | 1 (1.0) | 14 (24.6) | <0.001 |
| **Medical treatment** | | | | |
| Nimodipine for preventing and treating cerebral vasospasm, n (%) | 158 (94.6) | 106 (97.2) | 52 (89.7) | 0.066 |
| Route of administration of nimodipine, n (%) |  |  |  |  |
| Oral | 113 (71.5) | 72 (67.9) | 41 (78.8) | 0.153 |
| Intravenous | 60 (38.0) | 44 (41.5) | 16 (30.8) | 0.191 |
| Deep venous thrombosis prophylaxis, n (%) | 16 (10.6) | 6 (6.1) | 10 (18.9) | 0.015 |
| **Complications** | | | | |
| Rebleeding, n (%) | 10 (6.1) | 1 (0.9) | 9 (16.4) | <0.001 |
| Number of rebleeding sites, n (%): |  |  |  | 0.400 |
| Rebleeding from a single site | 4 (40.0) | 1 (100) | 3 (33.3) |  |
| Rebleeding from multiple sites | 6 (60.0) | 0 | 66 (66.7) |  |
| Rebleeding sites, n (%) |  |  |  |  |
| Subarachnoid hemorrhage, (n=9) | 3 (33.3) | 0 (0.0) | 3 (37.5) | 0.999 |
| Subdural hemorrhage | 4 (40.0) | 1 (100.0) | 3 (33.3) | 0.400 |
| Intraventricular hemorrhage | 6 (60.0) | 0 (0.0) | 6 (66.7) | 0.400 |
| Intracerebral hemorrhage | 7 (70.0) | 0 (0.0) | 7 (77.8) | 0.300 |
| Catheter-induced hemorrhage |  |  |  |  |
| Vasospasm and delayed cerebral ischemia, n (%) | 17 (10.4) | 5 (4.7) | 12 (21.4) | 0.002 |
| Acute hydrocephalus, n (%) | 76 (45.2) | 39 (35.5) | 37 (63.8) | <0.001 |
| Hyponatremia, n (%) | 34 (20.2) | 19 (17.3) | 15 (25.9) | 0.189 |
| Seizures, n (%) | 44 (26.2) | 32 (29.1) | 12 (20.7) | 0.239 |
| Chronic hydrocephalus, n (%) | 5 (6.0) | 3 (4.3) | 2 (14.3) | 0.196 |
| EVD obstruction, n (%) | 2/22 (9.1) | 0/8 | 2/14 (14.3) | 0.515 |
| EVD replacement, n (%) | 2/19 (10.5) | 0/6 | 2/13 (15.4) | >0.999 |
| Ventriculitis, n (%) | 8 (6.3) | 4 (4.0) | 4 (8.7) | 0.462 |
| Pneumonia, n (%) | 29 (17.3) | 10 (9.1) | 19 (32.8) | <0.001 |
| Urinary tract infection, n (%) | 3 (1.8) | 3 (2.7) | 0 (0.0) | 0.552 |
| **Clinical time course** | | | | |
| Ictus to hospital arrival (days), mean (SD) | 1.03 (2.57) | 1.27 (3.10) | 0.59 (0.80) | 0.010 |
| Ictus to hospital arrival (hours), n (%) |  |  |  | 0.034 |
| ≤ 24 hours | 69 (42.9) | 38 (36.2) | 31 (55.4) |  |
| >24–72 hours | 91 (56.5) | 66 (62.9) | 25 (44.6) |  |
| >72 hours | 1 (0.6) | 1 (1.0) | 0 (0.0) |  |
| EVD duration (days), mean (SD) | 6.88 (4.60) | 6.11 (3.26) | 7.31 (5.26) | 0.842 |
| EVD duration (days), n (%) |  |  |  | 0.999 |
| ≤ 7 | 17 (68.0) | 6 (66.7) | 11 (68.8) |  |
| > 7 | 8 (32.0) | 3 (33.3) | 5 (31.2) |  |
| Length of hospitalization (days), mean (SD) | 10.85 (9.40) | 11.46 (9.17) | 9.69 (9.81) | 0.036 |
| **Clinical outcomes** | | | | |
| Hospital discharge, n (%) | 52 (31.0) | 49 (44.5) | 3 (5.2) | <0.001 |
| Transferred to another hospital, n (%) | 91 (54.2) | 60 (54.5) | 31 (53.4) | 0.892 |
| Discharged to die, n (%) | 19 (11.3) | 1 (0.9) | 18 (31.0) | <0.001 |
| Died in hospital, n (%) | 6 (3.6) | 0 (0.0) | 6 (10.3) | 0.001 |
| Died within 30 days of ictus, n (%) | 35 (20.8) | 0 | 35 (60.3) | <0.001 |
| **Neurological function** | | | | |
| mRS score at hospital discharge, median (IQR) | 1 (1-5) | 1 (1-1) | 5 (5-5) | <0.001 |
| mRS at hospital discharge, n (%) |  |  |  | <0.001 |
| Good (mRS of 0 to 3) | 109 (64.9) | 108 (98.2) | 1 (1.7) |  |
| Poor (mRS of 4 to 6) | 59 (35.1) | 2 (1.8) | 57 (98.3) |  |
| GOS score at hospital discharge, median (IQR) | 4 (2-5) | 5 (4-5) | 2 (2-2.25) | <0.001 |
| GOS at hospital discharge, n (%) |  |  |  | <0.001 |
| Good (GOS of 3 to 5) | 123 (73.2) | 109 (99.1) | 14 (24.1) |  |
| Poor (GOS of 1 to 2) | 45 (26.8) | 1 (0.9) | 44 (75.9) |  |

**S7 Table**. Factors associated with poor outcome of patients with aneurysmal subarachnoid hemorrhage at 30 days after the onset of hemorrhage: bivariate regression analyses

|  | Frequency | OR | 95.0% CI for OR | | p-value |
| --- | --- | --- | --- | --- | --- |
|  |  |  | Lower | Upper |  |
| Transferred from local hospitals, n (%) |  |  |  |  |  |
| Hospital taken to, n (%) |  |  |  |  |  |
| Viet Duc | 22 | - | - | - | - |
| Bach Mai | 129 | 3.753 | 1.055 | 13.350 | 0.041 |
| Hanoi Medical University | 17 | 4.433 | 0.937 | 20.976 | 0.060 |
| **Demographics** | | | | | |
| Age (year) | 168 | 1.048 | 1.020 | 1.076 | 0.001 |
| Age (years): |  |  |  |  |  |
| 20 - 39 | 18 | - | - | - | - |
| 40 - 59 | 74 | 1.852 | 0.484 | 7.083 | 0.368 |
| ≥ 60 | 76 | 4.268 | 1.141 | 15.964 | 0.031 |
| Gender (male) | 91 | 1.045 | 0.552 | 1.978 | 0.892 |
| Resident regions: |  |  |  |  |  |
| Urban | 68 | - | - | - | - |
| Rural | 100 | 0.682 | 0.358 | 1.300 | 0.245 |
| **Socioeconomic status** | | | | | |
| Health insurance | 152 | 0.370 | 0.130 | 1.052 | 0.062 |
| Health insurance rates: |  |  |  |  |  |
| 100% | 30 | - | - | - | - |
| 95% | 23 | 0.616 | 1.329 | 0.438 | 4.030 |
| 80% | 94 | 0.481 | 0.733 | 0.309 | 1.739 |
| 60% | 1 | 0.000 | 0.000 |  | 1.000 |
| 40% | 4 | 0.000 | 0.000 |  | 0.999 |
| Elementary occupations | 46 | - | - | - | - |
| Managers | 2 | 5140147296 | 0.000 | - | 0.999 |
| Professionals | 3 | 1.591 | 0.131 | 19.270 | 0.715 |
| Technicians and associate professionals | 3 | 0.000 | 0.000 | - | 0.999 |
| Clerical support workers | 4 | 1.061 | 0.100 | 11.260 | 0.961 |
| Service and sales workers | 15 | 0.795 | 0.189 | 3.341 | 0.755 |
| Skilled agricultural, forestry and fishery workers | 36 | 2.273 | 0.881 | 5.863 | 0.090 |
| Plant and machine operators, and assemblers | 6 | 0.636 | 0.067 | 6.046 | 0.694 |
| Retired | 49 | 2.815 | 1.168 | 6.783 | 0.021 |
| Student/unemployment | 1 | 5140147296 | 0.000 | - | <0.999 |
| Other | 3 | 0.000 | 0.000 | - | 0.999 |
| Highest education levels: |  |  |  |  |  |
| School | 126 | - | - | - | - |
| Vocational school | 12 | 1.379 | 0.413 | 4.602 | 0.601 |
| Junior college | 8 | 0.643 | 0.125 | 3.324 | 0.599 |
| Senior college | 15 | .965 | 0.310 | 3.002 | 0.951 |
| College/University | 6 | 0.965 | 0.170 | 5.481 | 0.968 |
| Postgraduate | 1 | 3118242181 | 0.000 |  | >0.999 |
| Annual income: |  |  |  |  |  |
| Upper-middle (US $4,036 - US $12,475) | 18 | - | - | - | - |
| Lower-middle (US $1,026 - US $4,035) | 115 | 1.067 | 0.372 | 3.056 | 0.904 |
| Low (≤ US $1,025) | 34 | 1.091 | 0.327 | 3.645 | 0.888 |
| **Risk factors for aneurysmal subarachnoid hemorrhage** | | | | | |
| Cigarette smoking | 63 | 1.028 | 0.534 | 1.982 | 0.933 |
| Smoking behaviors: |  |  |  |  |  |
| Quit | 18 | - | - | - | - |
| Current | 45 | 1.103 | 0.348 | 3.501 | 0.867 |
| Hypertension | 64 | 3.364 | 1.729 | 6.543 | <0.001 |
| Genetic risk | 6 | 0.368 | 0.042 | 3.230 | 0.367 |
| Alcohol consumption | 81 | 1.004 | 0.531 | 1.897 | 0.991 |
| Alcohol drinking behaviors: |  |  |  |  |  |
| Occasionally | 45 | - | - | - | - |
| Sometimes | 25 | 0.474 | 0.159 | 1.415 | 0.181 |
| Every day | 11 | 0.857 | 0.219 | 3.358 | 0.825 |
| Sympathomimetic drugs | 2 | 0.000 | 0.000 |  | 0.999 |
| Estrogen deficiency | 32 | 1.482 | 0.603 | 3.645 | 0.391 |
| Antithrombotic therapy | 3 | 3.893 | 0.345 | 43.866 | 0.271 |
| Elevated total cholesterol | 8 | 1.145 | 0.264 | 4.972 | 0.856 |
| **Comorbidities** | | | | | |
| Cerebrovascular disease | 3 | 0.947 | 0.084 | 10.673 | 0.965 |
| Chronic cardiac failure | 3 | 3.893 | 0.345 | 43.866 | 0.271 |
| Coronary artery disease/IM | 3 | 3.893 | 0.345 | 43.866 | 0.271 |
| Active neoplasm | 3 | 0.947 | 0.084 | 10.673 | 0.965 |
| Chronic renal failure | 2 | 1.912 | 0.117 | 31.142 | 0.649 |
| Diabetes mellitus | 15 | 1.750 | 0.601 | 5.095 | 0.305 |
| Hematological disease | 2 | 1.912 | 0.117 | 31.142 | 0.649 |
| Others | 17 | 1.796 | 0.653 | 4.934 | 0.256 |
| **Onset symptoms** | | | | | |
| Sudden-onset, severe headache | 147 | 0.271 | 0.105 | 0.701 | 0.007 |
| Vomiting | 102 | 0.631 | 0.330 | 1.205 | 0.163 |
| Neck pain or stiffness | 66 | 1.024 | 0.534 | 1.963 | 0.943 |
| Photophobia | 6 | 0.368 | 0.042 | 3.230 | 0.367 |
| Blurred or double vision | 4 | 0.626 | 0.064 | 6.154 | 0.688 |
| Brief loss of consciousness | 70 | 5.231 | 2.639 | 10.407 | <0.001 |
| Seizures | 9 | 0.945 | 0.228 | 3.927 | 0.938 |
| Other | 132 | 1.771 | 0.770 | 4.074 | 0.179 |
| **Clinical presentation on admission** | | | | | |
| GCS score | 168 | 0.671 | 0.596 | 0.755 | <0.001 |
| GCS score: |  |  |  |  |  |
| Mild (13 - 15) | 100 | - | - | - | - |
| Moderate (9 - 12) | 29 | 5.437 | 2.134 | 13.858 | <0.001 |
| Severe (3 - 8) | 39 | 30.593 | 11.207 | 83.514 | <0.001 |
| Heart rate (beats/min) | 167 | 1.030 | 1.009 | 1.051 | 0.005 |
| Heart rate (beats/min): |  |  |  |  |  |
| ≤ 95 | 126 | - | - | - | - |
| > 95 | 41 | 2.526 | 1.226 | 5.202 | 0.012 |
| Respiratory rate (breaths/min) | 159 | 1.0720 | 0.986 | 1.167 | 0.104 |
| Respiratory rate (breaths/min): |  |  |  |  |  |
| 12 - 25 | 146 | - | - | - | - |
| > 25 | 13 | 3.833 | 1.186 | 12.381 | 0.025 |
| Systolic blood pressure (mmHg) | 167 | 1.008 | 0.996 | 1.020 | 0.176 |
| Systolic blood pressure (mmHg): |  |  |  |  |  |
| < 140 | 83 | - | - | - | - |
| ≥ 140 | 84 | 1.672 | 0.878 | 3.184 | 0.118 |
| Diastolic blood pressure (mmHg) | 167 | 1.025 | 1.000 | 1.050 | 0.047 |
| Diastolic blood pressure (mmHg): |  |  |  |  |  |
| < 90 | 117 | - | - | - | - |
| ≥ 90 | 50 | 2.878 | 1.447 | 5.724 | 0.003 |
| Body temperature (^o^C) | 167 | 1.804 | 0.864 | 3.764 | 0.116 |
| Body temperature (^o^C): |  |  |  |  |  |
| < 38 | 160 | - | - | - | - |
| ≥ 38 | 7 | 5.047 | 0.948 | 26.880 | 0.058 |
| Focal neurological deficits | 99 | 1.711 | 0.880 | 3.324 | 0.113 |
| Focal neurologic signs: |  |  |  |  |  |
| Third nerve palsy | 4 | 0.500 | 0.050 | 4.988 | 0.555 |
| Sixth nerve palsy | 1 | 0.000 | 0.000 |  | >0.999 |
| Hemiparesis | 33 | 1.208 | 0.516 | 2.830 | 0.663 |
| Aphasia | 6 | 1.583 | 0.303 | 8.276 | 0.586 |
| Bilateral leg weakness | 1 | 0.000 | 0.000 |  | >0.999 |
| Impaired level of consciousness | 49 | 5.800 | 2.365 | 14.224 | <0.001 |
| Brainstem signs | 3 | 3.189 | 0.279 | 36.421 | 0.351 |
| Neck stiffness | 52 | 0.464 | 0.204 | 1.054 | 0.067 |
| **Head imaging findings on admission** | | | | | |
| Non-contrast head computed tomography (CT) findings | | | | | |
| Location of blood within the subarachnoid space: |  |  |  |  |  |
| Basal cistern | 79 | 2.763 | 1.416 | 5.389 | 0.003 |
| Sylvian fissure | 157 | 1.258 | 0.313 | 5.060 | 0.746 |
| Interhemispheric fissure | 84 | 1.564 | 0.819 | 2.985 | 0.175 |
| Interpeduncular fossa | 83 | 3.611 | 1.820 | 7.166 | <0.001 |
| Suprasellar cistern | 90 | 1.562 | 0.814 | 2.999 | 0.180 |
| Ambient cistern | 81 | 3.456 | 1.753 | 6.814 | <0.001 |
| Quadrigeminal cistern | 27 | 4.207 | 1.775 | 9.974 | 0.001 |
| Maximum thickness of subarachnoid blood (mm) | 165 | 1.066 | 0.994 | 1.144 | 0.074 |
| IVH | 107 | 3.561 | 1.672 | 7.585 | 0.001 |
| Location of blood within the ventricular system: |  |  |  |  |  |
| Right lateral ventricle | 95 | 4.050 | 0.831 | 19.746 | 0.084 |
| Left lateral ventricle | 99 | 1460141033 | 0.000 | - | 0.999 |
| Third ventricle | 42 | 8.455 | 3.470 | 20.601 | <0.001 |
| Fourth ventricle | 43 | 2.588 | 1.166 | 5.744 | 0.019 |
| Graeb score | 102 | 1.620 | 1.293 | 2.030 | <0.001 |
| Graeb score: |  |  |  |  |  |
| Mild (1 - 4) | 69 | - | - | - | - |
| Moderate (5 - 8) | 24 | 14.167 | 4.265 | 47.058 | <0.001 |
| Severe (9 - 12) | 9 | 22.667 | 2.648 | 194.042 | 0.004 |
| ICH | 41 | 1.949 | 0.948 | 4.005 | 0.069 |
| ICH volume (mL) | 41 | 1.023 | 0.998 | 1.049 | 0.071 |
| ICH volume (mL): |  |  |  |  |  |
| < 30 | 26 | - | - | - | - |
| 30 - 60 | 7 | 1.200 | 0.221 | 6.521 | 0.833 |
| > 60 | 8 | 4.800 | 0.806 | 28.598 | 0.085 |
| Subdural hemorrhage | 10 | 1.981 | 0.549 | 7.146 | 0.296 |
| Hydrocephalus | 72 | 2.684 | 1.396 | 5.161 | 0.003 |
| Evans' index | 145 | 2379.810 | 8.353 | 677986.9 | 0.007 |
| Bicaudate index | 145 | 12295.43 | 42.796 | 3532531 | 0.001 |
| Relative bicaudate index | 145 | 5.164 | 1.788 | 14.910 | 0.002 |
| Hypodense lesions on computed tomography | 14 | 3.937 | 1.253 | 12.377 | 0.019 |
| Patterns of infarction: |  |  |  |  |  |
| Single cortical infarcts | 6 | 0.333 | 0.035 | 3.205 | 0.341 |
| Multiple widespread infarcts | 8 | 3.000 | 0.312 | 28.841 | 0.341 |
| Multislice computed tomography (MSCT) angiography / Digital subtraction angiography (DSA) findings | | | | | |
| Number of aneurysm | 168 | 0.781 | 0.359 | 1.700 | 0.533 |
| Number of aneurysm: |  |  |  |  |  |
| Single aneurysm | 144 | - | - | - | - |
| Multiple aneurysms | 24 | 0.590 | 0.220 | 1.578 | 0.293 |
| Side of aneurysm: |  |  |  |  |  |
| Right | 58 | - | - | - | - |
| Left | 49 | 0.655 | 0.290 | 1.480 | 0.309 |
| Both | 60 | 0.947 | 0.449 | 1.998 | 0.887 |
| Groups of aneurysm site: |  |  |  |  |  |
| Anterior circulation aneurysm | 120 | 1.303 | 0.548 | 3.096 | 0.549 |
| Posterior circulation aneurysm | 33 | 0.645 | 0.275 | 1.515 | 0.314 |
| Aneurysm site: |  |  |  |  |  |
| Internal carotid artery (ICA) | 35 | 0.987 | 0.450 | 2.161 | 0.973 |
| Ophtalmic segment of the ICA (OphIC) | 1 | 0.000 | 0.000 |  | >0.999 |
| Cavernous segment of the ICA (cIC) | 7 | 0.750 | 0.141 | 3.990 | 0.736 |
| Posterior communicating artery (PCoA) | 25 | 1.319 | 0.552 | 3.156 | 0.533 |
| Anterior cerebral artery (ACA) | 16 | 0.605 | 0.186 | 1.967 | 0.404 |
| Anterior communicating artery (AcoA) | 49 | 1.299 | 0.651 | 2.591 | 0.458 |
| Middle cerebral artery (MCA) | 34 | 0.885 | 0.397 | 1.973 | 0.766 |
| Vertebral artery (VA) | 7 | 1.445 | 0.312 | 6.689 | 0.637 |
| Posterior inferior cerebellar artery (PICA) | 4 | 0.626 | 0.064 | 6.154 | 0.688 |
| Basilar artery (BA) | 7 | 0.000 | 0.000 |  | 0.999 |
| Other | 129 | 1.719 | 0.770 | 3.834 | 0.186 |
| Maximum aneurysm size (mm): |  |  |  |  |  |
| Maximum dome width | 166 | 1.013 | 0.900 | 1.139 | 0.836 |
| Dome height | 163 | 1.073 | 0.965 | 1.194 | 0.195 |
| Maximum neck width | 158 | 1.259 | 0.997 | 1.591 | 0.053 |
| Dome-to-neck ratio | 157 | 1.447 | 0.903 | 2.317 | 0.124 |
| Shape of aneurysm: |  |  |  |  |  |
| Regular | 18 | - | - | - | - |
| Irregular | 117 | 2.350 | 0.729 | 7.580 | 0.153 |
| Multilobular | 11 | 2.000 | 0.382 | 10.482 | 0.412 |
| Two domes |  |  |  |  |  |
| Other | 3 | 1.750 | 0.124 | 24.650 | 0.678 |
| Vasospasm of the parent artery | 14 | 2.803 | 0.922 | 8.520 | 0.069 |
| **Severity of aneurysmal subarachnoid hemorrhage on admission** | | | | | |
| PAASH score | 168 | 3.378 | 2.348 | 4.860 | <0.001 |
| PAASH scale: |  |  |  |  |  |
| Grade I | 80 | - | - | - | - |
| Grade II | 31 | 2.301 | 0.773 | 6.849 | 0.134 |
| Grade III | 28 | 14.200 | 5.027 | 40.115 | <0.001 |
| Grade IV | 25 | 41.417 | 11.579 | 148.137 | <0.001 |
| Grade V | 4 | 23.667 | 2.219 | 252.423 | <0.001 |
| WFNS score | 168 | 2.371 | 1.825 | 3.081 | <0.001 |
| WFNS scale: |  |  |  |  |  |
| Grade I | 80 | - | - | - | - |
| Grade II | 14 | 2.152 | 0.503 | 9.198 | 0.301 |
| Grade III | 6 | 1.578 | 0.165 | 15.063 | 0.692 |
| Grade IV | 50 | 11.833 | 4.835 | 28.961 | <0.001 |
| Grade V | 18 | 39.444 | 9.531 | 163.251 | <0.001 |
| Modified WFNS score | 168 | 2.415 | 1.853 | 3.146 | <0.001 |
| Modified WFNS scale: |  |  |  |  |  |
| Grade I | 80 | - | - | - | - |
| Grade II | 14 | 1.315 | 0.253 | 6.845 | 0.745 |
| Grade III | 6 | 3.944 | 0.630 | 24.677 | 0.142 |
| Grade IV | 50 | 11.833 | 4.835 | 28.961 | <0.001 |
| Grade V | 18 | 39.444 | 9.531 | 163.251 | <0.001 |
| Hunt and Hess score | 168 | 2.63301.964 | 3.528 | <0.001 |  |
| Hunt and Hess scale: |  |  |  |  |  |
| Grade 1 | 39 | - | - | - | - |
| Grade 2 | 45 | 0.850 | 0.227 | 3.186 | 0.809 |
| Grade 3 | 25 | 2.644 | 0.734 | 9.530 | 0.137 |
| Grade 4 | 21 | 5.100 | 1.424 | 18.270 | 0.012 |
| Grade 5 | 38 | 36.267 | 10.071 | 130.599 | <0.001 |
| Fisher score | 168 | 3.789 | 1.733 | 8.285 | 0.001 |
| Fisher scale: |  |  |  |  |  |
| Group 2 | 13 | - | - | - | - |
| Group 1 | 1 | 0.000 | 0.000 | - | >0.999 |
| Group 3 | 34 | 2.069 | 0.218 | 19.629 | 0.527 |
| Group 4 | 120 | 9.176 | 1.156 | 72.846 | 0.036 |
| Claassen score | 168 | 1.839 | 1.245 | 2.716 | 0.002 |
| Claassen scale: |  |  |  |  |  |
| Grade 1 |  | - | - | - | - |
| Grade 2 | 22 | - | - | - | - |
| Grade 3 | 11 | 1.000 | 0.153 | 6.531 | >0.999 |
| Grade 4 | 39 | 0.984 | 0.253 | 3.825 | 0.982 |
| Grade 5 | 96 | 3.971 | 1.251 | 12.605 | 0.019 |
| **Laboratory investigations on admission** | | | | | |
| Complete blood count |  |  |  |  |  |
| Red blood cells (T/L) | 163 | 1.002 | 0.661 | 1.519 | 0.992 |
| Hemoglobin (g/L) | 163 | 1.003 | 0.998 | 1.019 | 0.686 |
| Hematocrit (L/L) | 163 | 296.020 | 0.157 | 557399.6 | 0.139 |
| Platelets (G/L) | 163 | 1.000 | 0.995 | 1.004 | 0.879 |
| White blood cells (G/L) | 163 | 1.120 | 1.039 | 1.207 | 0.003 |
| Percentage of neutrophils (%) | 162 | 1.011 | 0.998 | 1.025 | 0.085 |
| Coagulation |  |  |  |  |  |
| Prothrombin time (PT) | 155 | 1.007 | 0.996 | 1.019 | 0.215 |
| Prothrombin time with INR (PT-INR) | 155 | 20.743 | 0.449 | 959.297 | 0.121 |
| Activated partial thromboplastin time (APTT) | 153 | 1.003 | 0.990 | 1.015 | 0.689 |
| rAPTT | 152 | 0.969 | 0.855 | 1.099 | 0.625 |
| Blood biochemical investigation |  |  |  |  |  |
| Ure (mmol/L) | 166 | 1.263 | 1.053 | 1.515 | 0.012 |
| Glucose (mmol/L) | 156 | 1.593 | 1.323 | 1.918 | <0.001 |
| Glucose (mmol/L): |  |  |  |  |  |
| ≤ 6.4 | 29 | - | - | - | - |
| > 6.4 | 127 | 9.669 | 2.203 | 42.432 | 0.003 |
| Creatinine (µmol/L) | 167 | 1.020 | 1.004 | 1.036 | 0.012 |
| SGOT (UI/L) | 157 | 1.054 | 1.028 | 1.081 | <0.001 |
| SGPT (UI/L) | 165 | 1.029 | 1.010 | 1.049 | 0.003 |
| Na^+^ (mmol/L) | 167 | 1.051 | 0.984 | 1.122 | 0.139 |
| Na^+^ (mmol/L): |  |  |  |  |  |
| ≥ 135 | 128 | - | - | - | - |
| < 135 | 39 | 0.575 | 0.257 | 1.283 | 0.176 |
| K^+^ (mmol/L) | 167 | 0.768 | 0.382 | 1.545 | 0.459 |
| Cl^-^ (mmol/L) | 167 | 0.980 | 0.943 | 1.019 | 0.310 |
| **Aneurysm repairs and other treatments** | | | | | |
| No aneurysm repair | 26 | 23.438 | 6.634 | 82.812 | <0.001 |
| Endovascular coiling | 79 | 0.284 | 0.143 | 0.566 | <0.001 |
| Number of coils | 59 | 0.825 | 0.612 | 1.113 | 0.207 |
| Stent-assisted coiling | 2 | 0.000 | 0.000 |  | 0.999 |
| Balloon-assisted coiling | 9 | 0.000 | 0.000 |  | 0.999 |
| Surgical clipping | 63 | 0.731 | 0.375 | 1.425 | 0.357 |
| Number of clip attempts | 57 | 1.345 | 0.942 | 1.919 | 0.103 |
| Temporary vessel occlusion of the parent artery | 7 | 1.550 | 0.252 | 9.516 | 0.636 |
| Surgical hematoma evacuation or decompressive craniotomy | 7 | 12.577 | 1.476 | 107.177 | 0.021 |
| External ventricular drainage | 26 | 4.607 | 1.900 | 11.173 | 0.001 |
| Number of EVD | 26 | 969284759.1 | 0.000 |  | 0.999 |
| Intraventricular fibrinolysis | 3 | 3.893 | 0.345 | 43.866 | 0.271 |
| Ventriculoperitoneal shunt placement |  |  |  |  |  |
| **Airway management and mechanical ventilation** | | | | | |
| Tracheal intubation | 144 | 1089505260 | 0.000 |  | 0.999 |
| Mechanical ventilation | 141 | 1128886957 | 0.000 |  | 0.999 |
| Tracheotomy | 15 | 33.535 | 4.275 | 263.042 | 0.001 |
| **Medical treatment** | | | | | |
| Nimodipine for preventing and treating cerebral vasospasm | 158 | 0.245 | 0.059 | 1.020 | 0.053 |
| Route of administration of nimodipine: |  |  |  |  |  |
| Oral | 113 | 1.760 | 0.806 | 3.842 | 0.156 |
| Intravenous | 60 | 0.626 | 0.310 | 1.267 | 0.193 |
| Deep venous thrombosis prophylaxis | 16 | 3.566 | 1.217 | 10.477 | 0.020 |
| **Complications** | | | | | |
| Rebleeding | 10 | 21.130 | 2.602 | 171.626 | 0.004 |
| Number of rebleeding sites: |  |  |  |  |  |
| Rebleeding from a single site | 4 | - | - | - | - |
| Rebleeding from multiple sites | 6 | 538491621.5 | 0.000 |  | 0.999 |
| Rebleeding patterns: |  |  |  |  |  |
| Subarachnoid hemorrhage, (n=9) | 3 | 323094972.9 | 0.000 |  | 0.999 |
| Subdural hemorrhage | 4 | 0.000 | 0.000 |  | 0.999 |
| Intraventricular hemorrhage | 6 | 538491621.5 | 0.000 |  | 0.999 |
| Intracerebral hemorrhage | 7 | 807737432.2 | 0.000 |  | 0.999 |
| Vasospasm and delayed cerebral ischemia | 17 | 5.564 | 1.849 | 16.740 | 0.002 |
| Patterns of vasospasm and delayed cerebral ischemia: |  |  |  |  |  |
| Single cortical infarcts | 9 | - | - | - | - |
| Multiple widespread infarcts | 7 | 1.250 | 0.146 | 10.699 | 0.839 |
| Acute hydrocephalus | 76 | 3.208 | 1.653 | 6.224 | 0.001 |
| Hyponatremia | 34 | 1.671 | 0.775 | 3.601 | 0.190 |
| Seizures | 44 | 0.636 | 0.298 | 1.355 | 0.241 |
| Chronic hydrocephalus | 5 | 3.667 | 0.553 | 24.319 | 0.178 |
| EVD obstruction | 2 | 1076983243 | 0.000 |  | 0.999 |
| EVD replacement | 2 | 881168107.9 | 0.000 |  | 0.999 |
| Ventriculitis | 8 | 1.810 | 0.430 | 7.609 | 0.418 |
| Pneumonia | 29 | 4.872 | 2.081 | 11.403 | <0.001 |
| Urinary tract infection | 3 | 0.000 | 0.000 |  | 0.999 |
| **Clinical time course** | | | | | |
| Ictus to hospital arrival (hours): |  |  |  |  |  |
| ≤ 24 hours | 69 | - | - | - | - |
| >24–72 hours | 91 | 0.464 | 0.240 | 0.899 | 0.023 |
| >72 hours | 1 | 0.000 | 0.000 |  | >0.999 |
| EVD duration (days) | 25 | 1.065 | 0.875 | 1.296 | 0.527 |
| EVD duration (days): |  |  |  |  |  |
| ≤ 7 | 17 | - | - | - | - |
| > 7 | 8 | 0.909 | 0.159 | 5.195 | 0.915 |
| Length of hospitalization (days) | 168 | 0.978 | 0.943 | 1.015 | 0.248 |

**S8 Table**. Factors associated with poor outcome of patients with aneurysmal subarachnoid hemorrhage at 30 days after the onset of hemorrhage: multivariate logistic regression analyses (backward elimination)

| Steps | Factors | Unit | OR | 95% CI for OR | | p value |
| --- | --- | --- | --- | --- | --- | --- |
|  |  |  |  | Lower | Upper |  |
| 1 | **Demographics** | | | | | |
|  | Age (years): |  |  |  |  |  |
|  | 20 - 39 | % | - | - | - | 0.152 |
|  | 40 - 59 | % | 4.205 | 0.205 | 86.176 | 0.351 |
|  | ≥ 60 | % | 14.696 | 0.651 | 331.695 | 0.091 |
|  | **Risk factors of aneurysmal subarachnoid hemorrhage** | | | | | |
|  | Hypertension | % | 2.928 | 0.598 | 14.302 | 0.185 |
|  | **Head imaging findings on admission** | | | | | |
|  | Location of blood within the subarachnoid space: |  |  |  |  |  |
|  | Basal cistern | % | 3.299 | 0.664 | 16.405 | 0.145 |
|  | Interpeduncular fossa | % | 1.207 | 0.175 | 8.331 | 0.849 |
|  | Ambient cistern | % | 1.776 | 0.275 | 11.465 | 0.546 |
|  | Quadrigeminal cistern | % | 0.549 | 0.072 | 4.207 | 0.564 |
|  | IVH | % | 1.113 | 0.242 | 5.128 | 0.890 |
|  | ICH | % | 1.842 | 0.319 | 10.621 | 0.494 |
|  | **Severity of aneurysmal subarachnoid hemorrhage on admission** | | | | | |
|  | WFNS scale: |  |  |  |  |  |
|  | Grade I | % | - | - | - | 0.024 |
|  | Grade II | % | 2.873 | 0.209 | 39.442 | 0.430 |
|  | Grade III | % | 1.716 | 0.046 | 63.723 | 0.770 |
|  | Grade IV | % | 14.032 | 2.389 | 82.438 | 0.003 |
|  | Grade V | % | 139.084 | 4.496 | 4303.014 | 0.005 |
|  | **Aneurysm repairs and other treatments** | | | | | |
|  | Aneurysm repairs: |  |  |  |  |  |
|  | No aneurysm repair | % | - | - | - | 0.001 |
|  | Endovascular coiling | % | 0.017 | 0.002 | 0.161 | <0.001 |
|  | Surgical clipping | % | 0.023 | 0.002 | 0.220 | 0.001 |
|  | External ventricular drainage |  | 8.660 | 1.191 | 62.982 | 0.033 |
|  | **Medical treatment** | | | | | |
|  | Nimodipine for preventing and treating cerebral vasospasm | % | 7.166 | 0.226 | 227.640 | 0.264 |
|  | **Complications** | | | | | |
|  | Rebleeding | % | 71.836 | 3.631 | 1421.183 | 0.005 |
|  | Vasospasm and delayed cerebral ischemia | % | 15.024 | 2.008 | 112.396 | 0.008 |
|  | Acute hydrocephalus | % | 0.567 | 0.108 | 2.969 | 0.502 |
|  | Pneumonia | % | 2.354 | 0.477 | 11.617 | 0.293 |
|  | Constant | % | 0.004 |  |  | 0.015 |
| 2 | **Demographics** | | | | | |
|  | Age (years): |  |  |  |  |  |
|  | 20 - 39 | % | - | - | - | 0.151 |
|  | 40 - 59 | % | 4.333 | 0.218 | 86.045 | 0.336 |
|  | ≥ 60 | % | 14.913 | 0.672 | 331.122 | 0.088 |
|  | **Risk factors of aneurysmal subarachnoid hemorrhage** | | | | | |
|  | Hypertension | % | 2.989 | 0.632 | 14.126 | 0.167 |
|  | **Head imaging findings on admission** | | | | | |
|  | Location of blood within the subarachnoid space: |  |  |  |  |  |
|  | Basal cistern | % | 3.313 | 0.666 | 16.489 | 0.143 |
|  | Interpeduncular fossa | % | 1.208 | 0.175 | .8.318 | 0.848 |
|  | Ambient cistern | % | 1.771 | 0.275 | 11.385 | 0.547 |
|  | Quadrigeminal cistern | % | 0.547 | 0.071 | 4.195 | 0.562 |
|  | ICH | % | 1.841 | 0.318 | 10.668 | 0.496 |
|  | **Severity of aneurysmal subarachnoid hemorrhage on admission** | | | | | |
|  | WFNS scale: |  |  |  |  |  |
|  | Grade I | % | - | - | - | 0.017 |
|  | Grade II | % | 2.922 | 0.216 | 39.445 | 0.419 |
|  | Grade III | % | 1.668 | 0.044 | 63.337 | 0.783 |
|  | Grade IV | % | 14.510 | 2.632 | 80.002 | 0.002 |
|  | Grade V | % | 1041.839 | 4.649 | 4327.090 | 0.004 |
|  | **Aneurysm repairs and other treatments** | | | | | |
|  | Aneurysm repairs: |  |  |  |  |  |
|  | No aneurysm repair | % | - | - | - | 0.001 |
|  | Endovascular coiling | % | 0.017 | 0.002 | 0.158 | <0.001 |
|  | Surgical clipping | % | 0.022 | 0.002 | 0.212 | 0.001 |
|  | External ventricular drainage | % | 8.636 | 1.189 | 62.715 | 0.033 |
|  | **Medical treatment** | | | | | |
|  | Nimodipine for preventing and treating cerebral vasospasm | % | 7.340 | 0.237 | 227.484 | 0.255 |
|  | **Complications** | | | | | |
|  | Rebleeding | % | 74.209 | 3.875 | 1421.250 | 0.004 |
|  | Vasospasm and delayed cerebral ischemia | % | 14.824 | 2.013 | 107.686 | 0.008 |
|  | Acute hydrocephalus | % | 0.567 | 0.109 | 2.965 | 0.502 |
|  | Pneumonia | % | 2.345 | 0.479 | 11.487 | 0.293 |
|  | Constant | % | 0.004 |  |  | 0..015 |
| 3 | **Demographics** | | | | | |
|  | Age (years): |  |  |  |  |  |
|  | 20 - 39 | % | - | - | - | 0.153 |
|  | 40 - 59 | % | 4.702 | 0.260 | 84.882 | 0.294 |
|  | ≥ 60 | % | 15.550 | 0.706 | 342.404 | 0.082 |
|  | **Risk factors of aneurysmal subarachnoid hemorrhage** | | | | | |
|  | Hypertension | % | 3.015 | 0.639 | 14.224 | 0.163 |
|  | **Head imaging findings on admission** | | | | | |
|  | Location of blood within the subarachnoid space: |  |  |  |  |  |
|  | Basal cistern | % | 3.460 | 0.737 | 16.251 | 0.116 |
|  | Ambient cistern | % | 1.945 | 0.395 | 9.571 | 0.413 |
|  | Quadrigeminal cistern | % | 0.562 | 0.075 | 4.223 | 0.576 |
|  | ICH | % | 1.761 | 0.321 | 9.670 | 0.515 |
|  | **Severity of aneurysmal subarachnoid hemorrhage on admission** | | | | | |
|  | WFNS scale: |  |  |  |  |  |
|  | Grade I | % | - | - | - | 0.016 |
|  | Grade II | % | 2.802 | 0.217 | 36.226 | 0.430 |
|  | Grade III | % | 1.626 | 0.042 | 62.317 | 0.794 |
|  | Grade IV | % | 14.634 | 2.652 | 80.759 | 0.002 |
|  | Grade V | % | 141.981 | 4.678 | 4309.578 | 0.004 |
|  | **Aneurysm repairs and other treatments** | | | | | |
|  | Aneurysm repairs: |  |  |  |  |  |
|  | No aneurysm repair | % | - | - | - | 0.001 |
|  | Endovascular coiling | % | 0.016 | 0.002 | 0.149 | <0.001 |
|  | Surgical clipping | % | 0.023 | 0.002 | 0.212 | 0.001 |
|  | External ventricular drainage | % | 8.555 | 1.182 | 61.923 | 0.034 |
|  | **Medical treatment** | | | | | |
|  | Nimodipine for preventing and treating cerebral vasospasm | % | 7.205 | 0.241 | 215.770 | 0.255 |
|  | **Complications** | | | | | |
|  | Rebleeding | % | 78.295 | 4.260 | 1439.100 | 0.003 |
|  | Vasospasm and delayed cerebral ischemia | % | 14.885 | 2.034 | 108.950 | 0.008 |
|  | Acute hydrocephalus | % | 0.569 | 0.109 | 2.967 | 0.503 |
|  | Pneumonia | % | 2.335 | 0.475 | 11.469 | 0.296 |
|  | Constant | % | 0.004 |  |  | 0.015 |
| 4 | **Demographics** | | | | | |
|  | Age (years): |  |  |  |  |  |
|  | 20 - 39 | % | - | - | - | 0.168 |
|  | 40 - 59 | % | 4.147 | 0.242 | 70.963 | 0.326 |
|  | ≥ 60 | % | 13.355 | 0.648 | 275.240 | 0.093 |
|  | **Risk factors of aneurysmal subarachnoid hemorrhage** | | | | | |
|  | Hypertension | % | 2.978 | 0.634 | 13.995 | 0.167 |
|  | **Head imaging findings on admission** | | | | | |
|  | Location of blood within the subarachnoid space: |  |  |  |  |  |
|  | Basal cistern | % | 3.049 | 0.695 | 13.368 | 0.139 |
|  | Ambient cistern | % | 1.620 | 0.379 | 6.917 | 0.515 |
|  | ICH | % | 1.738 | 0.331 | 9.135 | 0.514 |
|  | **Severity of aneurysmal subarachnoid hemorrhage on admission** | | | | | |
|  | WFNS scale: |  |  |  |  |  |
|  | Grade I | % | - | - | - | 0.018 |
|  | Grade II | % | 2.981 | 0.228 | 38.961 | 0.405 |
|  | Grade III | % | 1.793 | 0.049 | 65.356 | 0.750 |
|  | Grade IV | % | 14.187 | 2.586 | 77.832 | 0.002 |
|  | Grade V | % | 115.634 | 4.201 | 3182.509 | 0.005 |
|  | **Aneurysm repairs and other treatments** | | | | | |
|  | Aneurysm repairs: |  |  |  |  |  |
|  | No aneurysm repair | % | - | - | - | 0.001 |
|  | Endovascular coiling | % | 0.016 | 0.002 | 0.145 | <0.001 |
|  | Surgical clipping | % | 0.025 | 0.003 | 0.219 | 0.001 |
|  | External ventricular drainage | % | 8.232 | 1.177 | 57.570 | 0.034 |
|  | **Medical treatment** | | | | | |
|  | Nimodipine for preventing and treating cerebral vasospasm | % | 6.585 | 0.215 | 201.209 | 0.280 |
|  | **Complications** | | | | | |
|  | Rebleeding | % | 80.165 | 4.382 | 1466.691 | 0.003 |
|  | Vasospasm and delayed cerebral ischemia | % | 13.896 | 2.003 | 96.429 | 0.008 |
|  | Acute hydrocephalus | % | 0.559 | 0.107 | 2.929 | 0.491 |
|  | Pneumonia | % | 2.332 | 0.472 | 11.516 | 0.299 |
|  | Constant | % | 0.005 |  |  | 0.016 |
| 5 | **Demographics** | | | | | |
|  | Age (years): |  |  |  |  |  |
|  | 20 - 39 | % | - | - | - | 0.185 |
|  | 40 - 59 | % | 3.364 | 0.243 | 46.557 | 0.365 |
|  | ≥ 60 | % | 9.948 | 0.643 | 153.980 | 0.100 |
|  | **Risk factors of aneurysmal subarachnoid hemorrhage** | | | | | |
|  | Hypertension | % | 3.486 | 0.788 | 15.429 | 0.100 |
|  | **Head imaging findings on admission** | | | | | |
|  | Location of blood within the subarachnoid space: |  |  |  |  |  |
|  | Basal cistern | % | 3.523 | 0.849 | 14.619 | 0.083 |
|  | ICH | % | 1.514 | 0.302 | 7.586 | 0.614 |
|  | **Severity of aneurysmal subarachnoid hemorrhage on admission** | | | | | |
|  | WFNS scale: |  |  |  |  |  |
|  | Grade I | % | - | - | - | 0.015 |
|  | Grade II | % | 2.873 | 0.214 | 38.548 | 0.426 |
|  | Grade III | % | 1.757 | 0.051 | 60.976 | 0.755 |
|  | Grade IV | % | 14.207 | 2.597 | 77.729 | 0.002 |
|  | Grade V | % | 142.340 | 5.238 | 3867.977 | 0.003 |
|  | **Aneurysm repairs and other treatments** | | | | | |
|  | Aneurysm repairs: |  |  |  |  |  |
|  | No aneurysm repair | % | - | - | - | 0.001 |
|  | Endovascular coiling | % | 0.016 | 0.002 | 0.141 | <0.001 |
|  | Surgical clipping | % | 0.027 | 0.003 | 0.228 | 0.001 |
|  | External ventricular drainage | % | 7.200 | 1.113 | 46.596 | 0.038 |
|  | **Medical treatment** | | | | | |
|  | Nimodipine for preventing and treating cerebral vasospasm | % | 7.906 | 0.254 | 245.779 | 0.238 |
|  | **Complications** | | | | | |
|  | Rebleeding | % | 73.499 | 4.293 | 1258.259 | 0.003 |
|  | Vasospasm and delayed cerebral ischemia | % | 12.347 | 1.885 | 80.876 | 0.009 |
|  | Acute hydrocephalus | % | 0.576 | 0.113 | 2.945 | 0.507 |
|  | Pneumonia | % | 2.529 | 0.520 | 12.300 | 0.250 |
|  | Constant | % | 0.006 |  |  | 0.020 |
| 6 | **Demographics** | | | | | |
|  | Age (years): |  |  |  |  |  |
|  | 20 - 39 | % | - | - | - | 0.175 |
|  | 40 - 59 | % | 3.800 | 0.306 | 47.152 | 0.299 |
|  | ≥ 60 | % | 10.804 | 0.738 | 158.183 | 0.082 |
|  | **Risk factors of aneurysmal subarachnoid hemorrhage** | | | | | |
|  | Hypertension | % | 3.515 | 0.789 | 15.663 | 0.099 |
|  | **Head imaging findings on admission** | | | | | |
|  | Location of blood within the subarachnoid space: |  |  |  |  |  |
|  | Basal cistern | % | 3.553 | 0.845 | 14.937 | 0.084 |
|  | **Severity of aneurysmal subarachnoid hemorrhage on admission** | | | | | |
|  | WFNS scale: |  |  |  |  |  |
|  | Grade I | % | - | - | - | 0.011 |
|  | Grade II | % | 3.334 | 0.271 | 40.960 | 0.347 |
|  | Grade III | % | 2.175 | 0.078 | 60.944 | 0.648 |
|  | Grade IV | % | 15.315 | 2.806 | 83.578 | 0.002 |
|  | Grade V | % | 172.351 | 6.554 | 4532.307 | 0.002 |
|  | **Aneurysm repairs and other treatments** | | | | | |
|  | Aneurysm repairs: |  |  |  |  |  |
|  | No aneurysm repair | % | - | - | - | 0.001 |
|  | Endovascular coiling | % | 0.015 | 0.002 | 0.133 | <0.001 |
|  | Surgical clipping | % | 0.028 | 0.003 | 0.232 | 0.001 |
|  | External ventricular drainage | % | 6.831 | 1.088 | 42.878 | 0.040 |
|  | **Medical treatment** | | | | | |
|  | Nimodipine for preventing and treating cerebral vasospasm | % | 8.400 | 0.272 | 259.557 | 0.224 |
|  | **Complications** | | | | | |
|  | Rebleeding | % | 80.714 | 4.663 | 1397.119 | 0.003 |
|  | Vasospasm and delayed cerebral ischemia | % | 11.931 | 1.837 | 77.489 | 0.009 |
|  | Acute hydrocephalus | % | 0.551 | 0.109 | 2.785 | 0.471 |
|  | Pneumonia | % | 2.450 | 0.518 | 11.603 | 0.259 |
|  | Constant | % | 0.006 |  |  | 0.018 |
| 7 | **Demographics** | | | | | |
|  | Age (years): |  |  |  |  |  |
|  | 20 - 39 | % | - | - | - | 0.211 |
|  | 40 - 59 | % | 3.387 | 0.277 | 41.446 | 0.340 |
|  | ≥ 60 | % | 8.842 | 0.642 | 121.792 | 0.103 |
|  | **Risk factors of aneurysmal subarachnoid hemorrhage** | | | | | |
|  | Hypertension | % | 3.442 | 0.770 | 15.387 | 0.106 |
|  | **Head imaging findings on admission** | | | | | |
|  | Location of blood within the subarachnoid space: |  |  |  |  |  |
|  | Basal cistern | % | 2.975 | 0.783 | 11.298 | 0.109 |
|  | **Severity of aneurysmal subarachnoid hemorrhage on admission** | | | | | |
|  | WFNS scale: |  |  |  |  |  |
|  | Grade I | % | - | - | - | 0.008 |
|  | Grade II | % | 3.108 | 0.265 | 36.399 | 0.366 |
|  | Grade III | % | 2.277 | 0.075 | 66.333 | 0.632 |
|  | Grade IV | % | 13.153 | 2.598 | 66.597 | 0.002 |
|  | Grade V | % | 124.588 | 6.612 | 2347.655 | 0.001 |
|  | **Aneurysm repairs and other treatments** | | | | | |
|  | Aneurysm repairs: |  |  |  |  |  |
|  | No aneurysm repair | % | - | - | - | 0.001 |
|  | Endovascular coiling | % | 0.016 | 0.002 | 0.139 | <0.001 |
|  | Surgical clipping | % | 0.027 | 0.003 | 0.233 | 0.001 |
|  | External ventricular drainage | % | 5.102 | 0.997 | 26.115 | 0.050 |
|  | **Medical treatment** | | | | | |
|  | Nimodipine for preventing and treating cerebral vasospasm | % | 6.500 | 0.251 | 168.349 | 0.260 |
|  | **Complications** | | | | | |
|  | Rebleeding | % | 56.693 | 4.049 | 793.832 | 0.003 |
|  | Vasospasm and delayed cerebral ischemia | % | 12.894 | 2.033 | 81.778 | 0.007 |
|  | Pneumonia | % | 2.422 | 0.522 | 11.233 | 0.258 |
|  | Constant | % | 0.008 |  |  | 0.019 |
| 8 | **Demographics** | | | | | |
|  | Age (years): |  |  |  |  |  |
|  | 20 - 39 | % | - | - | - | 0.187- |
|  | 40 - 59 | % | 4.251 | 0.329 | 54.920 | 0.268 |
|  | ≥ 60 | % | 10.400 | 0.728 | 148.587 | 0.084 |
|  | **Risk factors of aneurysmal subarachnoid hemorrhage** | | | | | |
|  | Hypertension | % | 3.075 | 0.725 | 13.049 | 0.128 |
|  | **Head imaging findings on admission** | | | | | |
|  | Location of blood within the subarachnoid space: |  |  |  |  |  |
|  | Basal cistern | % | 2.788 | 0.772 | 10.073 | 0.118 |
|  | **Severity of aneurysmal subarachnoid hemorrhage on admission** | | | | | |
|  | WFNS scale: |  |  |  |  |  |
|  | Grade I | % | - | - | - | 0.008 |
|  | Grade II | % | 3.175 | 0.287 | 35.160 | 0.346 |
|  | Grade III | % | 2.063 | 0.068 | 63.025 | 0.678 |
|  | Grade IV | % | 12.596 | 2.578 | 61.539 | 0.002 |
|  | Grade V | % | 66.951 | 5.100 | 878.850 | 0.001 |
|  | **Aneurysm repairs and other treatments** | | | | | |
|  | Aneurysm repairs: |  |  |  |  |  |
|  | No aneurysm repair | % | - | - | - | 0.001 |
|  | Endovascular coiling | % | 0.023 | 0.003 | 0.166 | <0.001 |
|  | Surgical clipping | % | 0.033 | 0.004 | 0.264 | 0.001 |
|  | External ventricular drainage | % | 4.799 | 0.945 | 24.372 | 0.059 |
|  | **Complications** | | | | | |
|  | Rebleeding | % | 57.636 | 4.170 | 796.613 | 0.002 |
|  | Vasospasm and delayed cerebral ischemia | % | 13.119 | 2.108 | 81.649 | 0.006 |
|  | Pneumonia | % | 2.279 | 0.512 | 10.146 | 0.280 |
|  | Constant | % | 0.038 |  |  | 0.028 |
| 9 | **Demographics** | | | | | |
|  | Age (years): |  |  |  |  |  |
|  | 20 - 39 | % | - | - | - | 0.175 |
|  | 40 - 59 | % | 4.135 | 0.360 | 47.524 | 0.254 |
|  | ≥ 60 | % | 9.921 | 0.795 | 123.832 | 0.075 |
|  | **Risk factors of aneurysmal subarachnoid hemorrhage** | | | | | |
|  | Hypertension | % | 3.496 | 0.825 | 14.806 | 0.089 |
|  | **Head imaging findings on admission** | | | | | |
|  | Location of blood within the subarachnoid space: |  |  |  |  |  |
|  | Basal cistern | % | 3.265 | 0.924 | 11.541 | 0.066 |
|  | **Severity of aneurysmal subarachnoid hemorrhage on admission** | | | | | |
|  | WFNS scale: |  |  |  |  |  |
|  | Grade I | % | - | - | - | 0.003 |
|  | Grade II | % | 3.352 | 0.326 | 34.504 | 0.309 |
|  | Grade III | % | 1.767 | 0.054 | 58.286 | 0.750 |
|  | Grade IV | % | 15.941 | 3.375 | 75.292 | <0.001 |
|  | Grade V | % | 75.307 | 5.980 | 948.419 | 0.001 |
|  | **Aneurysm repairs and other treatments** | | | | | |
|  | Aneurysm repairs: |  |  |  |  |  |
|  | No aneurysm repair | % | - | - | - | 0.001 |
|  | Endovascular coiling | % | 0.026 | 0.004 | 0.186 | <0.001 |
|  | Surgical clipping | % | 0.034 | 0.004 | 0.268 | 0.001 |
|  | External ventricular drainage | % | 5.356 | 1.103 | 26.000 | 0.037 |
|  | **Complications** | | | | | |
|  | Rebleeding | % | 65.718 | 4.654 | 927.996 | 0.002 |
|  | Vasospasm and delayed cerebral ischemia | % | 13.736 | 2.167 | 87.061 | 0.005 |
|  | Constant | % | 0.034 |  |  | 0.019 |
| 10 | **Risk factors of aneurysmal subarachnoid hemorrhage** | | | | | |
|  | Hypertension | % | 5.822 | 1.588 | 21.342 | 0.008 |
|  | **Head imaging findings on admission** | | | | | |
|  | Location of blood within the subarachnoid space: |  |  |  |  |  |
|  | Basal cistern | % | 3.736 | 1.082 | 12.899 | 0.037 |
|  | **Severity of aneurysmal subarachnoid hemorrhage on admission** | | | | | |
|  | WFNS scale: |  |  |  |  |  |
|  | Grade I | % | - | - | - | 0.003 |
|  | Grade II | % | 1.842 | 0.209 | 16.256 | 0.583 |
|  | Grade III | % | 1.740 | 0.073 | 41.440 | 0.732 |
|  | Grade IV | % | 14.367 | 3.155 | 65.429 | 0.001 |
|  | Grade V | % | 54.391 | 4.831 | 612.362 | 0.001 |
|  | **Aneurysm repairs and other treatments** | | | | | |
|  | Aneurysm repairs: |  |  |  |  |  |
|  | No aneurysm repair | % | - | - | - | 0.001 |
|  | Endovascular coiling | % | 0.032 | 0.005 | 0.194 | <0.001 |
|  | Surgical clipping | % | 0.044 | 0.007 | 0.291 | 0.001 |
|  | External ventricular drainage |  | 4.202 | 1.006 | 17.556 | 0.049 |
|  | **Complications** | | | | | |
|  | Rebleeding | % | 71.142 | 4.915 | 1029.672 | 0.002 |
|  | Vasospasm and delayed cerebral ischemia | % | 11.581 | 1.897 | 70.698 | 0.008 |
|  | Constant | % | 0.143 |  |  | 0.070 |

**S9 Table**. Demographic and baseline characteristics, management and outcomes of patients with aneurysmal subarachnoid hemorrhage according to survivability within 30 days after the onset of hemorrhage

|  | All cases  (n=168) | Survived  (n=133) | Died  (n=35) | p-value |
| --- | --- | --- | --- | --- |
| Transferred from local hospitals, n (%) |  |  |  |  |
| Hospital taken to, n (%) |  |  |  | 0.012 |
| Viet Duc | 22 (13.1) | 22 (16.5) | 0 |  |
| Bach Mai | 129 (76.8) | 97 (72.9) | 32 (91.4) |  |
| Hanoi Medical University | 17 (10.1) | 14 (10.5) | 3 (8.6) |  |
| **Demographics** | | | | |
| Age (year), median (IQR) | 57 (48-67) | 56 (46-66) | 62 (53-71) | 0.031 |
| Age (years), n (%) |  |  |  | 0.276 |
| 20 - 39 | 18 (10.7) | 16 (12.0) | 2 (5.7) |  |
| 40 - 59 | 74 (44.0) | 16 (45.9) | 13 (37.1) |  |
| ≥ 60 | 76 (45.2) | 56 (42.1) | 20 (57.1) |  |
| Gender (male), n (%) | 77 (45.8) | 61 (45.9) | 16 (45.7) | >0.999 |
| Resident regions, n (%) |  |  |  | 0.562 |
| Urban | 68 (40.5) | 52 (39.1) | 16 (45.7) |  |
| Rural | 100 (59.5) | 81 (60.9) | 19 (54.3) |  |
| **Socioeconomic status** | | | | |
| Health insurance, n (%) | 152 (90.5) | 124 (93.2) | 28 (80.0) | 0.045 |
| Health insurance rates, n (%) |  |  |  | 0.723 |
| 100% | 30 (19.7) | 25 (20.2) | 5 (17.9) |  |
| 95% | 23 (15.1) | 17 (13.7) | 6 (21.4) |  |
| 80% | 94 (61.8) | 77 (62.1) | 17 (60.7) |  |
| 60% | 1 (0.7) | 1 (0.8) | 0 (0.0) |  |
| 40% | 4 (2.6) | 4 (3.2) | 0 (0.0) |  |
| Occupations, n (%) |  |  |  | 0.031 |
| Managers | 2 (1.2) | 1 (0.8) | 1 (2.9) |  |
| Professionals | 3 (1.8) | 2 (1.5) | 1 (2.9) |  |
| Technicians and associate professionals | 3 (1.8) | 3 (2.3) | 1 (2.9) |  |
| Clerical support workers | 4 (2.4) | 3 (2.3) | 1 (2.9) |  |
| Service and sales workers | 15 (8.9) | 13 (9.8) | 2 (5.7) |  |
| Skilled agricultural, forestry and fishery workers | 36 (3.6) | 24 (18.0) | 12 (34.3) |  |
| Craft and related trades workers | 0 | 0 | 0 |  |
| Plant and machine operators, and assemblers | 6 (3.6) | 5 (3.8) | 1 (2.9) |  |
| Elementary occupations | 46 (27.4) | 43 (32.3) | 3 (8.6) |  |
| Armed forces occupations | 0 | 0 | 0 |  |
| Retired | 49 (29.2) | 36 (27.1) | 13 (37.1) |  |
| Student/unemployment | 1 (0.6) | 0 (0.0) | 1 (2.9) |  |
| Other | 3 (1.8) | 3 (2.3) | 0 (0.0) |  |
| Highest education levels, n (%) |  |  |  | 0.631 |
| School | 126 (75.0) | 100 (75.2) | 26 (74.3) |  |
| Vocational school | 12 (7.1) | 10 (7.5) | 2 (5.7) |  |
| Junior college | 8 (4.8) | 6 (4.5) | 2 (5.7) |  |
| Senior college | 15 (8.9) | 12 (9.0) | 3 (8.6) |  |
| College/University | 6 (3.6) | 5 (3.8) | 1 (2.9) |  |
| Postgraduate | 1 (0.6) | 0 (0.0) | 1 (2.9) |  |
| Annual income, n (%) |  |  |  | 0.501 |
| Upper-middle (US $4,036 - US $12,475) | 18 (10.8) | 13 (9.8) | 5 (14.3) |  |
| Lower-middle (US $1,026 - US $4,035) | 115 (68.9) | 90 (68.2) | 25 (71.4) |  |
| Low (≤ US $1,025) | 34 (20.4) | 29 (22.0) | 5 (14.3) |  |
| **Risk factors of aneurysmal subarachnoid hemorrhage** | | | | |
| Cigarette smoking, n (%) | 63 (37.5) | 50 (37.6) | 13 (37.1) | >0.999 |
| Smoking behaviors, n (%) |  |  |  | 0.316 |
| Quit | 18 (28.6) | 16 (32.0) | 2 (15.4) |  |
| Current | 45 (71.4) | 34 (38.0) | 11 (84.6) |  |
| Hypertension, n (%) | 64 (38.1) | 46 (34.6) | 18 (51.4) | 0.080 |
| Genetic risk, n (%) | 6 (3.6) | 5 (3.8) | 1 (2.9) | >0.999 |
| Inherited conditions, n (%) |  |  |  | >0.999 |
| Polycystic kidney disease | 1 (16.7) | 1 (20.0) | 0 (0.0) |  |
| A family history of subar-achnoid hemorrhage/unru-ptured aneurysms | 5 (83.3) | 4 (80.0) | 1 (100.0) |  |
| Alcohol consumption, n (%) | 81 (48.2) | 64 (48.1) | 17 (48.6) | >0.999 |
| Alcohol drinking behaviors, n (%) |  |  |  | 0.437 |
| Occasionally | 45 (55.6) | 34 (53.1) | 11 (64.7) |  |
| Sometimes | 25 (30.9) | 22 (34.4) | 3 (17.6) |  |
| Every day | 11 (13.6) | 8 (12.5) | 3 (17.6) |  |
| Sympathomimetic drugs, n (%) | 2/167 (1.2) | 2/132 (1.5) | 0/35 (0.0) | >0.999 |
| Estrogen deficiency, n (%) | 32/89 (36.0) | 26/70 (37.1) | 6/19 (31.6) | 0.790 |
| Antithrombotic therapy, n (%) | 3 (1.8) | 2 (1.5) | 1 (2.9) | 0.506 |
| Elevated total cholesterol, n (%) | 8 (4.8) | 6 (4.5) | 2 (5.7) | 0.672 |
| **Preexisting comorbidities** | | | | |
| Cerebrovascular disease, n (%) | 3 (1.8) | 2 (1.5) | 1 (2.9) | 0.506 |
| Chronic cardiac failure, n (%) | 3 (1.8) | 2 (1.5) | 1 (2.9) | 0.506 |
| Coronary artery disease/IM, n (%) | 3 (1.8) | 2 (1.5) | 1 (2.9) | 0.506 |
| COPD /Asthma, n (%) | 0 | 0 | 0 | - |
| Chronic pulmonary disease, n (%) | 0 | 0 | 0 | - |
| Tuberculosis, n (%) | 0 | 0 | 0 | - |
| Active neoplasm, n (%) | 3 (1.8) | 2 (2.3) | 0 (0.0) | >0.999 |
| Chronic renal failure, n (%) | 2 (1.2) | 1 (0.8) | 1 (2.9) |  |
| Ulcer disease, n (%) | 0 |  |  |  |
| Diabetes mellitus, n (%) | 15 (8.9) | 11 (8.3) | 4 (11.4) | 0.519 |
| Immunoincompetence, n (%) | 0 |  |  |  |
| Hematological disease, n (%) | 2 (1.2) | 2 (1.5) | 0 (0.0) | >0.999 |
| Others, n (%) | 17 (10.1) | 14 (10.5) | 3 (8.6) | >0.999 |
| **Onset symptoms** | | | | |
| Sudden-onset, severe headache, n (%) | 147 (87.5) | 119 (89.5) | 28 (80.0) | 0.152 |
| Vomiting, n (%) | 102 (60.7) | 83 (62.4) | 19 (54.3) | 0.438 |
| Neck pain or stiffness, n (%) | 66 (39.3) | 56 (42.1) | 10 (28.6) | 0.175 |
| Photophobia, n (%) | 6 (3.6) | 5 (3.8) | 1 (2.9) | >0.999 |
| Blurred or double vision, n (%) | 4 (2.4) | 3 (2.3) | 1 (2.9) | >0.999 |
| Brief loss of consciousness, n (%) | 70 (41.7) | 46 (34.6) | 24 (68.6) | <0.001 |
| Seizures, n (%) | 9 (5.4) | 7 (5.3) | 2 (5.7) | >0.999 |
| Other, n (%) | 132 (78.6) | 100 (75.2) | 32 (91.4) | 0.039 |
| **Clinical presentation on admission** | | | | |
| GCS score, median (IQR) | 14 (9-15) | 15 (12-15) | 7 (6-10) | <0.001 |
| GCS score, n (%) |  |  |  | <0.001 |
| Mild (13 - 15) | 100 (59.5) | 95 (71.4) | 5 (14.3) |  |
| Moderate (9 - 12) | 29 (17.3) | 22 (16.5) | 7 (20.0) |  |
| Severe (3 - 8) | 39 (23.2) | 16 (12.0) | 23 (65.7) |  |
| Heart rate (beats/min), median (IQR), n=167 | 85 (76-95) | 85 (75-90) | 90 (80-100) | 0.031 |
| Heart rate (beats/min), n (%) | n=167 | n=132 | n=35 | 0.075 |
| ≤ 95 | 126 (75.4) | 104 (78.8) | 22 (62.9) |  |
| > 95 | 41 (24.6) | 28 (21.2) | 13 (37.1) |  |
| Respiratory rate (breaths/min), median (IQR), (n=159) | 20 (18-21) | 20 (18-21) | 20 (18-21.25) | 0.491 |
| Respiratory rate (breaths/min), n (%) | n=159 | n=129 | n=30 |  |
| < 12 | 0 | 0 | 0 |  |
| 12 - 25 | 146 (91.8) | 120 (93.0) | 25 (86.7) |  |
| > 25 | 13 (9.2) | 9 (9.0) | 4 (13.3) |  |
| Systolic blood pressure (mmHg), mean (SD), n=167 | 137.40 (28.07) | 136.71 (23.35) | 139.97 (41.70) | 0.477 |
| Systolic blood pressure (mmHg), n (%) | n=167 | n=132 | n=35 | 0.448 |
| < 140 | 83 (49.7) | 68 (51.5) | 15 (42.9) |  |
| ≥ 140 | 84 (50.3) | 64 (48.5) | 20 (57.1) |  |
| Diastolic blood pressure (mmHg), mean (SD), n=167 | 79.90 (13.73) | 78.87 (11.95) | 93.77 (18.72) | 0.105 |
| Diastolic blood pressure (mmHg), n (%) | n=167 | n=132 | n=35 | 0.036 |
| < 90 | 117 (70.1) | 98 (74.2) | 19 (54.3) |  |
| ≥ 90 | 50 (29.9) | 34 (25.8) | 16 (45.7) |  |
| Body temperature (^o^C), mean (SD), n=167 | 36.93 (0.45) | 36.92 (0.47) | 36.97 (0.33) | 0.397 |
| Body temperature (^o^C), n (%) | n=167 | n=132 | n=35 | 0.638 |
| < 38 | 160 (95.8) | 127 (96.2) | 33 (94.3) |  |
| ≥ 38 | 7 (4.2) | 5 (3.8) | 2 (5.7) |  |
| Focal neurological deficits, n (%) | 99 (58.9) | 74 (55.6) | 25 (71.4) | 0.122 |
| Focal neurologic signs, n (%): |  |  |  |  |
| Third nerve palsy | 4 (4.0) | 4 (5.4) | 0 (0.0) | 0.569 |
| Sixth nerve palsy | 1 (1.0) | 1 (1.4) | 0 (0.0) | >0.999 |
| Hemiparesis | 33 (33.3) | 24 (32.4) | 9 (36.0) | 0.808 |
| Aphasia | 6 (6.1) | 4 (5.4) | 2 (8.0) | 0.641 |
| Bilateral leg weakness | 1 (1.0) | 1 (1.4) | 0 (0.0) | >0.999 |
| Ophthalmoplegia | 0 | 0 | 0 | - |
| Unilateral visual loss or bitemporal hemianopia | 0 | 0 | 0 | - |
| Impaired level of consciousness | 49 (49.5) | 29 (39.2) | 20 (80.0) | <0.001 |
| Brainstem signs | 3 (3.0) | 1 (1.4) | 2 (8.0) | 0.156 |
| Neck stiffness | 52 (52.5) | 43 (58.1) | 9 (36.0) | 0.067 |
| Retinal and subhyaloid hemorrhages | 0 | 0 | 0 | - |
| Preretinal hemorrhages (Terson syndrome) | 0 | 0 | 0 | - |
| Other | 0 | 0 | 0 | - |
| **Head imaging findings on admission** | | | | |
| Non-contrast head computed tomography (CT) findings | | | | |
| Detection of blood within the subarachnoid space, n (%) | 167 (99.4) | 132 (99.2) | 35 (100.0) | >0.999 |
| Location of blood within the subarachnoid space, n (%) |  |  |  |  |
| Basal cistern | 79/165 (47.9) | 57/132 (43.2) | 22/33 (66.7) | 0.019 |
| Sylvian fissure | 157 (94.0) | 125 (94.7) | 32 (91.4) | 0.438 |
| Interhemispheric fissure | 84/166 (50.6) | 61/132 (46.2) | 23/34 (67.6) | 0.034 |
| Interpeduncular fossa | 83/166 (50.0) | 59/132 (44.7) | 24/34 (70.6) | 0.012 |
| Suprasellar cistern | 90/166 (54.2) | 68/132 (51.5) | 22/34 (64.7) | 0.182 |
| Ambient cistern | 81/166 (48.8) | 57/132 (43.2) | 24/34 (70.6) | 0.007 |
| Quadrigeminal cistern | 27/166 (16.3) | 14/132 (10.6) | 13/34 (38.2) | <0.001 |
| Maximum thickness of subarachnoid blood (mm), mean (SD), n=165 | 6.30 (4.56) | 5.98 (4.58) | 7.53 (4.31) | 0.034 |
| IVH, n (%) | 107 (63.7) | 79 (59.4) | 28 (80.0) | 0.029 |
| Location of blood within the ventricular system, n (%) |  |  |  |  |
| Right lateral ventricle | 95/106 (89.6) | 67/78 (85.9) | 28/28 (100.0) | 0.035 |
| Left lateral ventricle | 99/106 (93.4) | 71/78 (91.0) | 28/28 (100.0) | 0.186 |
| Third ventricle | 42/106 (39.6) | 20/78 (25.6) | 22/28 (78.6) | <0.001 |
| Fourth ventricle | 43/106 (40.6) | 25/78 (32.1) | 18/28 (64.3) | 0.004 |
| Graeb score, median (IQR), n=102 | 3 (2-5.25) | 3(2-4) | 5 (3-7) | <0.001 |
| Graeb score, n (%) | n=102 | n=75 | n=27 | <0.001 |
| Mild (1 - 4) | 69 (67.6) | 60 (80.0) | 9 (33.3) |  |
| Moderate (5 - 8) | 24 (23.5) | 9 (12.0) | 15 (55.6) |  |
| Severe (9 - 12) | 9 (8.8) | 6 (8.0) | 3 (11.1) |  |
| ICH, n (%) | 41 (24.4) | 28 (21.1) | 13 (37.1) | 0.075 |
| ICH volume (mL), mean (SD), n=41 | 29.29 (28.21) | 23.69 (23.46) | 41.35 (34.40) | 0.117 |
| ICH volume (mL), n (%) | n=41 | n=28 | n=13 | 0.503 |
| < 30 | 26 (63.4) | 19 (67.9) | 7 (53.8) |  |
| 30 - 60 | 7 (17.1) | 5 (17.9) | 2 (15.4) |  |
| > 60 | 8 (19.5) | 4 (14.3) | 4 (30.8) |  |
| Subdural hemorrhage, n (%) | 10 (6.0) | 6 (4.5) | 4 (11.4) | 0.219 |
| Hydrocephalus, n (%) | 72 (42.9) | 51 (38.3) | 21 (60.0) | 0.034 |
| Evans' index, mean (SD), n=145 | 0.30 (0.06) | 0.30 (0.06) | 0.31 (0.07) | 0.244 |
| Bicaudate index, mean (SD), n=145 | 0.20 (0.06) | 0.19 (0.06) | 0.22 (0.08) | 0.062 |
| Relative bicaudate index, mean (SD), n=145 | 1.07 (0.34) | 1.03 (0.30) | 1.18 (0.42) | 0.074 |
| Hypodense lesions on computed tomography, n (%) | 14/167 (8.4) | 10 (7.5) | 4 (11.8) | 0.487 |
| Patterns of infarction, n (%) | n=14 | n=10 | n=4 |  |
| Single cortical infarcts | 6 (42.9) | 5 (50.0) | 5 (25.0) | 0.580 |
| Multiple widespread infarcts | 8 (57.1) | 5 (50.0) | 3 (75.0) | 0.580 |
| Multislice computed tomography (MSCT) angiography / Digital subtraction angiography (DSA) findings | | | | |
| Detection of intracranial aneurysm, n (%) | 168 (100) | 133 (100) | 35 (100) | - |
| Number of aneurysm, median (IQR) | 1 (1-1) | 1 (1-1) | 1 (1-1) | 0.263 |
| Number of aneurysm, n (%) |  |  |  | 0.278 |
| Single aneurysm | 144 (85.7) | 112 (84.2) | 32 (91.4) |  |
| Multiple aneurysms | 24 (14.3) | 21 (15.8) | 3 (8.6) |  |
| Side of aneurysm, n (%) |  |  |  | 0.749 |
| Right | 58 (34.7) | 44 (33.3) | 14 (40.0) |  |
| Left | 49 (29.3) | 41 (31.1) | 8 (22.9) |  |
| Both | 60 (35.9) | 47 (35.6) | 13 (37.1) |  |
| Groups of aneurysm site, n (%) |  |  |  |  |
| Anterior circulation aneurysm | 120/150 (80.0) | 92/119 (77.3) | 28/31 (90.3) | 0.134 |
| Posterior circulation aneurysm | 33/150 (22.0) | 30/119 (25.2) | 3/31 (9.7) | 0.087 |
| Aneurysm site, n (%) | n=168 | n=133 | n=35 |  |
| Internal carotid artery (ICA) | 35 (20.8) | 29 (21.8) | 6 (17.1) | 0.645 |
| Ophtalmic segment of the ICA (OphIC) | 1 (0.6) | 1 (0.8) | 0 (00.0) | >0.999 |
| Cavernous segment of the ICA (cIC) | 7 (4.2) | 6 (4.5) | 1 (2.9) | >0.999 |
| Anterior choroidal artery segment of the ICA (AchIC) | 0 | 0 | 0 | - |
| Posterior communicating artery (PCoA) | 25 (14.9) | 21 (15.8) | 4 (11.4) | 0.605 |
| Anterior cerebral artery (ACA) | 16 (9.5) | 13 (9.8) | 3 (8.6) | >0.999 |
| Anterior communicating artery (AcoA) | 49 (29.2) | 38 (28.6) | 11 (31.4) | 0.835 |
| Middle cerebral artery (MCA) | 34 (20.2) | 26 (19.5) | 8 (22.9) | 0.643 |
| Posterior cerebral artery (PCA) | 0 | 0 | 0 | - |
| Vertebral artery (VA) | 7 (4.2) | 5 (3.8) | 2 (5.7) | 0.637 |
| Superior cerebellar artery (SCA) | 0 |  |  |  |
| Posterior inferior cerebellar artery (PICA) | 4 (2.4) | 3 (2.3) | 1 (2.9) | >0.999 |
| Anterior inferior cerebellar artery (AICA) | 0 |  |  |  |
| Basilar artery (BA) | 7 (4.2) | 7 (5.3) | 0 (0.0) | 0.347 |
| Other | 129 (76.8) | 97 (72.9) | 32 (91.4) | 0.024 |
| Maximum aneurysm size (mm), mean (SD), |  |  |  |  |
| Maximum dome width, n=166 | 4.75 (2.70) | 4.73 (2.67) | 4.84 (2.87) | 0.568 |
| Dome height, n=163 | 5.50 (5.79) | 5.45 (6.34) | 5.71 (2.95) | 0.282 |
| Maximum neck width, n=158 | 3.35 (2.83) | 3.26 (3.03) | 3.67 (1.91) | 0.083 |
| Dome-to-neck ratio, n=157 | 1.65 (3.71) | 1.73 (4.18) | 1.38 (0.53) | 0.582 |
| Shape of aneurysm, n (%) | n=149 | n=114 | n=35 |  |
| Regular | 18 (12.1) | 16 (14.0) | 2 (5.7) |  |
| Irregular | 117 (78.5) | 87 (76.3) | 30 (85.7) |  |
| Multilobular | 11 (7.4) | 9 (7.9) | 2 (5.7) |  |
| Two domes | 0 | 0 | 0 |  |
| Other | 3 (2.0) | 2 (1.8) | 1 (2.9) |  |
| Vasospasm of the parent artery, n (%), n=166 | 14 (8.4) | 7 (5.3) | 7 (20.0) | 0.012 |
| **Severity of aneurysmal subarachnoid hemorrhage on admission** | | | | |
| PAASH score, median (IQR) | 2 (1-3) | 1 (1-2) | 4 (3-4) | <0.001 |
| PAASH scale, n (%) |  |  |  | <0.002 |
| Grade I | 80 (47.6) | 78 (5.6) | 2 (5.7) |  |
| Grade II | 31 (18.5) | 27 (20.3) | 4 (11.4) |  |
| Grade III | 28 (16.7) | 18 (13.5) | 10 (28.6) |  |
| Grade IV | 25 (14.9) | 8 (6.0) | 17 (48.6) |  |
| Grade V | 4 (2.4) | 2 (1.5) | 2 (5.7) |  |
| WFNS score, median (IQR) | 2 (1-4) | 1 (1-4) |  |  |
| WFNS scale, n (%) |  |  |  | <0.001 |
| Grade I | 80 (47.6) | 78 (5.6) | 2 (5.7) |  |
| Grade II | 14 (8.3) | 12 (9.0) | 2 (5.7) |  |
| Grade III | 6 (3.6) | 5 (3.8) | 1 (2.9) |  |
| Grade IV | 50 (29.8) | 32 (24.1) | 18 (51.4) |  |
| Grade V | 18 (10.7) | 6 (4.5) | 12 (34.4) |  |
| Modified WFNS score, median (IQR) | 2 (1-4) | 1 (1-4) | 4 (4-5) | <0.001 |
| Modified WFNS scale, n (%) |  |  |  | <0.001 |
| Grade I | 80 (47.6) | 78 (58.6) | 2 (5.7) |  |
| Grade II | 14 (8.3) | 12 (9.0) | 2 (5.7) |  |
| Grade III | 6 (3.6) | 5 (3.8) | 1 (2.9) |  |
| Grade IV | 50 (29.8) | 32 (24.1) | 18 (51.4) |  |
| Grade V | 18 (10.7) | 6 (4.5) | 12 (34.3) |  |
| Hunt and Hess score, median (IQR) | 2.5 (2-4) | 2 (1-3) | 5 (4-5) | <0.001 |
| Hunt and Hess scale, n (%) |  |  |  | <0.001 |
| Grade 1 | 39 (23.2) | 38 (28.6) | 1 (2.9) |  |
| Grade 2 | 45 (26.8) | 44 (33.1) | 1 (2.9) |  |
| Grade 3 | 25 (14.9) | 20 (15.0) | 5 (14.3) |  |
| Grade 4 | 21 (12.5) | 16 (12.0) | 5 (14.3) |  |
| Grade 5 | 38 (22.6) | 15 (11.3) | 23 (65.7) |  |
| Fisher score, median (IQR) | 4 (3-4) | 4 (3-4) | 4 (4-4) | 0.009 |
| Fisher scale, n (%) |  |  |  | 0.045 |
| Group 1 | 1 (0.6) | 1 (0.8) | 0 (0.0) |  |
| Group 2 | 13 (7.7) | 13 (9.8) | 0 (0.0) |  |
| Group 3 | 34 (20.2) | 30 (22.6) | 4 (11.4) |  |
| Group 4 | 120 (71.4) | 89 (66.9) | 31 (88.6) |  |
| Claassen score, median (IQR) | 5 (4-5) | 5 (4-5) | 5 (5-5) | 0.002 |
| Claassen scale, n (%) |  |  |  |  |
| Grade 1 |  |  |  | 0.018 |
| Grade 2 | 22 (13.1) | 20 (15.0) | 2 (5.7) |  |
| Grade 3 | 11 (6.5) | 11 (8.3) | 0 (0.0) |  |
| Grade 4 | 39 (23.2) | 35 (25.6) | 5 (14.3) |  |
| Grade 5 | 96 (57.1) | 68 (51.1) | 28 (80.0) |  |
| **Laboratory investigations on admission** | | | | |
| Complete blood count |  |  |  |  |
| Red blood cells (T/L), mean (SD) | 4.46 (0.78) | 4.45 (0.82) | 4.49 (0.58) | 0.570 |
| Hemoglobin (g/L), mean (SD) | 132.07 (21.09) | 131.78 (19.91) | 133.18 (25.40) | 0.335 |
| Hematocrit (L/L), mean (SD) | 0.40 (0.04) | 0.39 (0.05) | 0.40 (0.04) | 0.188 |
| Platelets (G/L), mean (SD) | 247.21 (76.12) | 246.60 (76.47) | 249.53 (75.87) | 0.987 |
| White blood cells (G/L), mean (SD) | 19.30 (54.87) | 14.16 (8.56) | 38.78 (118.34) | <0.001 |
| Percentage of neutrophils (%), mean (SD), (n=165) | 70.58 (28.72) | 68.44 (29.69) | 78.65 (23.36) | 0.008 |
| Coagulation |  |  |  |  |
| Prothrombin time (PT), mean (SD) | 91.95 (32.59) | 90.06 (35.45) | 99.18 (16.22) | 0.558 |
| Prothrombin time with INR (PT-INR), mean (SD) | 1.00 (0.09) | 1.00 (0.09) | 1.02 (0.11) | 0.716 |
| Activated partial thromboplastin time (APTT), mean (SD), (n=155) | 44.21 (200.54) | 27.98 (4.39) | 105.55 (438.41) | 0.554 |
| rAPTT, mean (SD), (n=154) | 1.55 (6.82) | 1.70 (7.67) | 0.99 (0.28) | 0.550 |
| Blood biochemical investigation |  |  |  |  |
| Ure (mmol/L), mean (SD), (n=169) | 5.39 (1.80) | 5.26 (1.80) | 5.87 (1.77) | 0.053 |
| Glucose (mmol/L), mean (SD), (n=159) | 8.21 (2.42) | 7.66 (2.11) | 10.18 (2.44) | <0.001 |
| Glucose (mmol/L), n (%) |  |  |  | 0.001 |
| ≤ 6.4 | 29 (18.6) | 29 (23.8) | 0 (0.0) |  |
| > 6.4 | 127 (81.4) | 93 (76.2) | 34 (100) |  |
| Creatinine (µmol/L), mean (SD) | 67.40 (25.40) | 65.09 (25.06) | 76.11 (25.11) | 0.006 |
| SGOT (UI/L), mean (SD), (n=160) | 34.30 (26.01) | 29.55 (20.79) | 50.83 (34.68) | <0.001 |
| SGPT (UI/L), mean (SD), (n=168) | 28.77 (24.01) | 26.10 (23.00) | 38.66 (25.44) | <0.001 |
| Na^+^ (mmol/L), mean (SD) | 136.62 (10.76) | 135.98 (11.66) | 139.00 (5.90) | 0.061 |
| Na^+^ (mmol/L), n (%) |  |  |  | 0.183 |
| ≥ 135 | 128 (76.6) | 98 (74.2) | 30 (85.7) |  |
| < 135 | 39 (23.4) | 34 (25.8) | 5 (14.3) |  |
| K^+^ (mmol/L), mean (SD) | 3.56 (0.46) | 3.56 (0.44) | 3.57 (0.55) | 0.888 |
| Cl^-^ (mmol/L), mean (SD) | 99.95 (8.89) | 99.67 (9.41) | 101.01 (6.59) | 0.914 |
| **Aneurysm repairs and other treatments** | | | | |
| No aneurysm repair, n (%) | 26 (15.5) | 6 (4.5) | 20 (57.1) | <0.001 |
| Endovascular coiling, n (%) | 79 (47.0) | 71 (53.4) | 8 (22.9) | 0.001 |
| Number of coils, median (IQR) | 5 (3-7) | 5 (4-7) | 3 (3-5) | 0.064 |
| Stent-assisted coiling, n (%) | 2 (2.6) | 2 (1.9) | 0 (0.0) | >0.999 |
| Balloon-assisted coiling, n (%) | 9 (11.7) | 9 (13.0) | 0 (0.0) | 0.585 |
| Surgical clipping, n (%) | 63 (37.5) | 56 (42.1) | 7 (20.0) | 0.018 |
| Number of clip attempts, median (IQR) | 4 (2-5) | 3.5 (2-5) | 4 (2.5-4.5) | 0.677 |
| Temporary vessel occlusion of the parent artery, n (%) | 7 (15.2) | 6 (14.6) | 1 (20.0) | >0.999 |
| Surgical hematoma evacuation or decompressive craniotomy, n (%) | 7 (4.2) | 3 (2.3) | 4 (11.4) | 0.035 |
| External ventricular drainage, n (%) | 26 (15.6) | 19 (14.4) | 7 (20.0) | 0.416 |
| Number of EVD, median (IQR) | 1 (1-1) | 1 (1-1) | 1 (1-) | 0.381 |
| Intraventricular fibrinolysis, n (%) | 3 (1.8) | 2 (1.5) | 1 (2.9) | 0.506 |
| **Airway management and mechanical ventilation** | | | | |
| Tracheal intubation, n (%) | 144/151 (95.4) | 109/116 (94.0) | 35/35 (100.0) | 0.202 |
| Mechanical ventilation, n (%) | 141/149 (94.6) | 106/114 (93.0) | 35/35 (100.0) | 0.199 |
| Tracheotomy, n (%) | 15/161 (9.3) | 12/127 (9.4) | 3/34 (8.8) | >0.999 |
| **Medical treatment** | | | | |
| Nimodipine for preventing and treating cerebral vasospasm, n (%) | 158/167 (94.6) | 128/132 (97.0) | 30/35 (85.7) | 0.021 |
| Route of administration of nimodipine, n (%) |  |  |  |  |
| Oral | 113 (71.5) | 88 (68.8) | 25 (83.3) | 0.123 |
| Intravenous | 60 (38.0) | 54 (42.2) | 6 (20.0) | 0.035 |
| Deep venous thrombosis prophylaxis, n (%) | 16/151 (10.6) | 12/119 (10.1) | 4/32 (12.5) | 0.747 |
| **Complications** | | | | |
| Rebleeding, n (%) | 10/164 (6.1) | 6/131 (4.6) | 4/33 (12.1) | 0.116 |
| Number of rebleeding sites, n (%): |  |  |  | 0.190 |
| Rebleeding from a single site | 4 (40.0) | 1 (16.7) | 3 (75.0) |  |
| Rebleeding from multiple sites | 6 (60.0) | 5 (83.3) | 1 (25.0) |  |
| Rebleeding sites, n (%) |  |  |  |  |
| Subarachnoid hemorrhage, (n=9) | 3 (33.3) | 2 (40.0) | 1 (25.0) | >0.999 |
| Subdural hemorrhage | 4 (40.0) | 4 (66.7) | 0 (0.0) | 0.076 |
| Intraventricular hemorrhage | 6 (60.0) | 4 (66.7) | 2 (50.0) | >0.999 |
| Intracerebral hemorrhage | 7 (70.0) | 5 (83.3) | 2 (50.0) | 0.500 |
| Vasospasm and delayed cerebral ischemia, n (%) | 17 (10.4) | 11 (8.6) | 6 (17.1) | 0.207 |
| Acute hydrocephalus, n (%) | 76 (45.2) | 54 (40.6) | 22 (62.9) | 0.022 |
| Hyponatremia, n (%) | 34 (20.2) | 28 (21.1) | 6 (17.1) | 0.608 |
| Seizures, n (%) | 44 (26.2) | 41 (30.8) | 3 (8.6) | 0.009 |
| Chronic hydrocephalus, n (%) | 5/83 (6.0) | 4/78 (5.1) | 1/5 (20.0) | 0.273 |
| EVD obstruction, n (%) | 2/22 (9.1) | 2/15 (13.3) | 0/7 (0.0) | >0.999 |
| EVD replacement, n (%) | 2/19 (10.5) | 2/14 (14.3) | 0/5 (0.0) | >0.999 |
| Ventriculitis, n (%) | 8/126 (6.3) | 8/96 (8.3) | 0/30 (0.0) | 0.197 |
| Pneumonia, n (%) | 29 (17.3) | 22 (16.5) | 7 (20.0) | 0.621 |
| Urinary tract infection, n (%) | 3 (1.8) | 3 (2.3) | 0 (0.0) | >0.999 |
| **Clinical time course** | | | | |
| Ictus to hospital arrival (days), mean (SD) | 1.03 (2.57) | 1.16 (2.87) | 0.56 (0.56) | 0.133 |
| Ictus to hospital arrival (hours), n (%) | n=161 | n=127 | n=34 | 0.761 |
| ≤ 24 hours | 69 (42.9) | 53 (41.7) | 16 (47.1) |  |
| >24–72 hours | 91 (56.5) | 73 (57.5) | 18 (52.9) |  |
| >72 hours | 1 (0.6) | 1 (0.8) | 0 (0.0) |  |
| EVD duration (days), mean (SD), n=25 | 6.88 (4.60) | 7.21 (4.77) | 5.83 (4.26) | 0.422 |
| EVD duration (days), n (%) |  |  |  | 0.624 |
| ≤ 7 | 17 (68.0) | 12 (63.2) | 5 (83.3) |  |
| > 7 | 8 (32.0) | 7 (36.8) | 1 (16.7) |  |
| Length of hospitalization (days), mean (SD) | 10.85 (9.40) | 12.50 (9.54) | 4.60 (5.52) | <0.001 |
| **Clinical outcomes** | | | | |
| Hospital discharge, n (%) | 52 (31.0) | 52 (39.1) | 0 (0.0) | <0.001 |
| Transferred to another hospital, n (%) | 91 (54.2) | 75 (56.4) | 16 (45.7) | 0.341 |
| Discharged to die, n (%) | 19 (11.3) | 6 (4.5) | 13 (37.1) | <0.001 |
| Died in hospital, n (%) | 6 (3.6) | 0 (0.0) | 6 (17.1) | <0.001 |
| **Neurological function** | | | | |
| mRS score at hospital discharge, median (IQR) | 1 (1-5) | 1 (1-3) | 5 (5-5) | <0.001 |
| mRS at hospital discharge, n (%) |  |  |  | <0.001 |
| Good (mRS of 0 to 3) | 109 (64.9) | 108 (81.2) | 1 (2.9) |  |
| Poor (mRS of 4 to 6) | 59 (35.1) | 25 (18.8) | 34 (97.1) |  |
| GOS score at hospital discharge, median (IQR) | 4 (2-5) | 5 (4-5) | 2 (2-2) | <0.001 |
| GOS at hospital discharge, n (%) |  |  |  | <0.001 |
| Good (GOS of 3 to 5) | 123 (73.2) | 121 (91.0) | 2 (5.7) |  |
| Poor (GOS of 1 to 2) | 45 (26.8) | 12 (9.0) | 33 (94.3) |  |

**S10 Table**. Factors associated with death within 30 days after the onset of hemorrhage in patients with aneurysmal subarachnoid hemorrhage: bivariate regression analyses

|  | Frequency | OR | 95.0% CI for OR | | p-value |
| --- | --- | --- | --- | --- | --- |
|  |  |  | Lower | Upper |  |
| Transferred from local hospitals, n (%) |  |  |  |  |  |
| Hospital taken to, n (%) |  |  |  |  |  |
| Viet Duc | 22 | - | - | - | - |
| Bach Mai | 129 | 532939132.1 | 0.000 | - | 0.998 |
| Hanoi Medical University | 17 | 346172516.6 | 0.000 | - | 0.998 |
| **Demographics** |  |  |  |  |  |
| Age (year) | 168 | 1.031 | 1.001 | 1.061 | 0.044 |
| Age (years): |  |  |  |  |  |
| 20 - 39 | 18 | - | - | - | - |
| 40 - 59 | 74 | 1.705 | 0.349 | 8.337 | 0.510 |
| ≥ 60 | 76 | 2.857 | 0.603 | 13.544 | 0.186 |
| Gender (male) | 77 | 0.994 | 0.471 | 2.099 | 0.987 |
| Resident regions: |  |  |  |  |  |
| Urban | 68 | - | - | - | - |
| Rural | 100 | 0.762 | 0.360 | 1.615 | 0.479 |
| **Socioeconomic status** | | | | | |
| Health insurance | 152 | 0.290 | 0.100 | 0.846 | 0.023 |
| Health insurance rates: |  |  |  |  |  |
| 100% | 30 | - | - | - | - |
| 95% | 23 | 1.765 | 0.463 | 6.721 | 0.405 |
| 80% | 94 | 1.104 | 0.370 | 3.298 | 0.859 |
| 60% | 1 | 0 | 0 |  | >0.999 |
| 40% | 4 | 0 | 0 |  | 0.999 |
| Highest education levels: |  |  |  |  |  |
| School | 126 | - | - | - | - |
| Vocational school | 12 | 0.769 | 0.159 | 3.728 | 0.745 |
| Junior college | 8 | 1.282 | 0.244 | 6.726 | 0.769 |
| Senior college | 15 | 0.962 | 0.253 | 3.660 | 0.954 |
| College/University | 6 | 0.769 | 0.086 | 6.873 | 0.814 |
| Postgraduate | 1 | 6213364864 | 0 |  | >0.999 |
| Annual income: |  |  |  |  |  |
| Upper-middle (US $4,036 - US $12,475) | 18 | - | - | - | - |
| Lower-middle (US $1,026 - US $4,035) | 115 | 0.722 | 0.235 | 2.219 | 0.570 |
| Low (≤ US $1,025) | 34 | 0.448 | 0.110 | 1.821 | 0.262 |
| **Risk factors for aneurysmal subarachnoid hemorrhage** | | | | | |
| Cigarette smoking | 63 | 0.981 | 0.454 | 2.119 | 0.961 |
| Smoking behaviors: |  |  |  |  |  |
| Quit | 18 | - | - | - | - |
| Current | 45 | 2.588 | 0.512 | 13.0373 | 0.250 |
| Hypertension | 64 | 2.003 | 0.943 | 4.252 | 0.071 |
| Genetic risk | 6 | 0.753 | 0.085 | 6.661 | 0.799 |
| Alcohol consumption | 81 | 1.018 | 0.483 | 2.145 | 0.962 |
| Alcohol drinking behaviors: |  |  |  |  |  |
| Occasionally | 45 | - | - | - | - |
| Sometimes | 25 | 0.421 | 0.106 | 1.683 | 0.221 |
| Every day | 11 | 1.159 | 0.261 | 5.148 | 0.846 |
| Sympathomimetic drugs | 2 | 0 | 0 |  | 0.999 |
| Estrogen deficiency | 32 | 0.781 | 0.265 | 2.305 | 0.654 |
| Antithrombotic therapy | 3 | 1.926 | 0.170 | 21.881 | 0.597 |
| Elevated total cholesterol | 8 | 1.283 | 0.247 | 6.650 | 0.767 |
| **Comorbidities** | | | | | |
| Cerebrovascular disease | 3 | 1.926 | 0.170 | 21.881 | 0.597 |
| Chronic cardiac failure | 3 | 1.926 | 0.170 | 21.881 | 0.597 |
| Coronary artery disease/IM | 3 | 1.926 | 0.170 | 21.881 | 0.597 |
| Active neoplasm | 3 | 0 | 0 |  | 0.999 |
| Chronic renal failure | 2 | 3.882 | 0.237 | 63.673 | 0.342 |
| Diabetes mellitus | 15 | 1.431 | 0.427 | 4.801 | 0.562 |
| Hematological disease | 2 | 0 | 0 |  | 0.999 |
| Others | 17 | 0.797 | 0.216 | 2.943 | 0.733 |
| **Onset symptoms** | | | | | |
| Sudden-onset, severe headache | 147 | 0.471 | 0.174 | 1.274 | 0.138 |
| Vomiting | 102 | 0.715 | 0.337 | 1.517 | 0.383 |
| Neck pain or stiffness | 66 | 0.550 | 0.245 | 1.236 | 0.148 |
| Photophobia | 6 | 0.753 | 0.085 | 6.661 | 0.799 |
| Blurred or double vision | 4 | 1.275 | 0.128 | 12.642 | 0.836 |
| Brief loss of consciousness | 70 | 4.126 | 1.858 | 9.166 | <0.001 |
| Seizures | 9 | 1.091 | 0.216 | 5.499 | 0.916 |
| Other | 132 | 3.520 | 1.011 | 12.251 | 0.048 |
| **Clinical presentation on admission** | | | | | |
| GCS score | 168 | 0.679 | 0.598 | 0.771 | <0.001 |
| GCS score: |  |  |  |  |  |
| Mild (13 - 15) | 100 | - | - | - | - |
| Moderate (9 - 12) | 29 | 6.045 | 1.753 | 20.845 | 0.004 |
| Severe (3 - 8) | 39 | 27.312 | 9.067 | 82.269 | <0.001 |
| Heart rate (beats/min) | 167 | 1.021 | 0.999 | 1.044 | 0.057 |
| Heart rate (beats/min): |  |  |  |  |  |
| ≤ 95 | 126 | - | - | - | - |
| > 95 | 41 | 2.195 | 0.984 | 4.898 | 0.055 |
| Respiratory rate (breaths/min) | 159 | 1.008 | 0.911 | 1.115 | 0.877 |
| Respiratory rate (breaths/min): |  |  |  |  |  |
| 12 - 25 | 146 | - | - | - | - |
| > 25 | 13 | 2.051 | 0.587 | 7.172 | 0.261 |
| Systolic blood pressure (mmHg) | 167 | 1.004 | 0.991 | 1.017 | 0.541 |
| Systolic blood pressure (mmHg): |  |  |  |  |  |
| < 140 | 83 | - | - | - | - |
| ≥ 140 | 84 | 1.417 | 0.668 | 3.003 | 0.364 |
| Diastolic blood pressure (mmHg) | 167 | 1.026 | 0.998 | 1.054 | 0.065 |
| Diastolic blood pressure (mmHg): |  |  |  |  |  |
| < 90 | 117 | - | - | - | - |
| ≥ 90 | 50 | 2.427 | 1.123 | 5.248 | 0.024 |
| Body temperature (^o^C) | 167 | 1.312 | 0.582 | 2.958 | 0.512 |
| Body temperature (^o^C): |  |  |  |  |  |
| < 38 | 160 | - | - | - | - |
| ≥ 38 | 7 | 1.539 | 0.286 | 8.292 | 0.616 |
| Focal neurological deficits | 99 | 1.993 | 0.887 | 4.477 | 0.095 |
| Focal neurologic signs: |  |  |  |  |  |
| Third nerve palsy | 4 | 0 | 0 |  | 0.999 |
| Sixth nerve palsy | 1 | 0 | 0 |  | >0.999 |
| Hemiparesis | 33 | 1.172 | 0.453 | 3.032 | 0.744 |
| Aphasia | 6 | 1.522 | 0.261 | 8.859 | 0.640 |
| Bilateral leg weakness | 1 | 0 | 0 |  | >0.999 |
| Impaired level of consciousness | 49 | 6.207 | 2.096 | 18.377 | 0.001 |
| Brainstem signs | 3 | 6.348 | 0.550 | 73.251 | 0.139 |
| Neck stiffness | 52 | 0.406 | 0.159 | 1.036 | 0.059 |
| **Head imaging findings on admission** | | | | | |
| Non-contrast head computed tomography (CT) findings | | | | | |
| Detection of blood within the subarachnoid space | 167 | 4.28E+8 | 0 |  | >0.999 |
| Location of blood within the subarachnoid space: |  |  |  |  |  |
| Basal cistern | 79 | 2.632 | 1.181 | 5.866 | 0.018 |
| Sylvian fissure | 157 | 0.597 | 0.146 | 2.440 | 0.473 |
| Interhemispheric fissure | 84 | 2.434 | 1.098 | 5.394 | 0.028 |
| Interpeduncular fossa | 83 | 2.969 | 1.316 | 6.699 | 0.009 |
| Suprasellar cistern | 90 | 1.725 | 0.790 | 3.771 | 0.171 |
| Ambient cistern | 81 | 3.158 | 1.399 | 7.128 | 0.006 |
| Quadrigeminal cistern | 27 | 5.218 | 2.151 | 12.658 | <0.001 |
| Maximum thickness of subarachnoid blood (mm) | 165 | 1.075 | 0.992 | 1.164 | 0.079 |
| IVH | 107 | 2.734 | 1.114 | 6.709 | 0.028 |
| Location of blood within the ventricular system: |  |  |  |  |  |
| Right lateral ventricle | 95 | 0 | 0 |  | 0.999 |
| Left lateral ventricle | 99 | 6.37E+8 | 0 |  | 0.999 |
| Third ventricle | 42 | 10.633 | 3.774 | 29.962 | <0.001 |
| Fourth ventricle | 43 | 3.816 | 1.540 | 9.457 | 0.004 |
| Graeb score | 102 | 1.308 | 1.105 | 1.548 | 0.002 |
| Graeb score: |  |  |  |  |  |
| Mild (1 - 4) | 69 | - | - | - | - |
| Moderate (5 - 8) | 24 | 11.111 | 3.760 | 32.830 | <0.001 |
| Severe (9 - 12) | 9 | 3.333 | 0.705 | 15.751 | 0.129 |
| ICH | 41 | 2.216 | 0.993 | 4.944 | 0.052 |
| ICH volume (mL) | 41 | 1.023 | 0.998 | 1.048 | 0.073 |
| ICH volume (mL): |  |  |  |  |  |
| < 30 | 26 | - | - | - | - |
| 30 - 60 | 7 | 1.086 | 0.170 | 6.938 | 0.931 |
| > 60 | 8 | 2.714 | 0.529 | 13.916 | 0.231 |
| Subdural hemorrhage | 10 | 2.731 | 0.726 | 10.272 | 0.137 |
| Hydrocephalus | 72 | 2.412 | 1.127 | 5.163 | 0.023 |
| Evans' index | 145 | 49.522 | 0.129 | 18992.44 | 0.199 |
| Bicaudate index | 145 | 1284.682 | 2.998 | 550582.3 | 0.021 |
| Relative bicaudate index | 145 | 3.688 | 1.168 | 11.647 | 0.026 |
| Hypodense lesions on computed tomography | 8 | 3.000 | 0.227 | 39.608 | 0.404 |
| Patterns of infarction: |  |  |  |  |  |
| Single cortical infarcts | 14 | - | - | - | - |
| Multiple widespread infarcts | 6 | 0.333 | 0.025 | 4.401 | 0.404 |
| Multislice computed tomography (MSCT) angiography / Digital subtraction angiography (DSA) findings | | | | | |
| Number of aneurysm | 168 | 0.490 | 0.155 | 1.554 | 0.226 |
| Number of aneurysm: |  |  |  |  |  |
| Single aneurysm | 144 | - | - | - | - |
| Multiple aneurysms | 24 | 0.500 | 0.140 | 1.784 | 0.285 |
| Side of aneurysm: |  |  |  |  |  |
| Right | 58 | - | - | - | - |
| Left | 49 | 0.613 | 0.233 | 1.613 | 0.322 |
| Both | 60 | 0.869 | 0.369 | 2.053 | 0.869 |
| Groups of aneurysm site: |  |  |  |  |  |
| Anterior circulation aneurysm | 120 | 2.739 | 0.773 | 9.711 | 0.119 |
| Posterior circulation aneurysm | 33 | 0.318 | 0.090 | 1.121 | 0.075 |
| Aneurysm site: |  |  |  |  |  |
| Internal carotid artery (ICA) | 35 | 0.742 | 0.281 | 1.959 | 0.547 |
| Ophtalmic segment of the ICA (OphIC) | 1 | 0 | 0 |  | >0.999 |
| Cavernous segment of the ICA (cIC) | 7 | 0.623 | 0.072 | 5.348 | 0.666 |
| Posterior communicating artery (PCoA) | 25 | 0.688 | 0.220 | 2.154 | 0.521 |
| Anterior cerebral artery (ACA) | 16 | 0.865 | 0.232 | 3.222 | 0.829 |
| Anterior communicating artery (AcoA) | 49 | 1.146 | 0.511 | 2.2567 | 0.741 |
| Middle cerebral artery (MCA) | 34 | 1.219 | 0.497 | 2.993 | 0.665 |
| Vertebral artery (VA) | 7 | 1.552 | 0.288 | 8.357 | 0.609 |
| Posterior inferior cerebellar artery (PICA) | 4 | 1.275 | 0.128 | 12.642 | 0.836 |
| Basilar artery (BA) | 7 | 0 | 0 |  | 0.999 |
| Other | 129 | 3.959 | 1.141 | 13.731 | 0.030 |
| Maximum aneurysm size (mm): |  |  |  |  |  |
| Maximum dome width | 166 | 1.016 | 0.886 | 1.165 | 0.820 |
| Dome height | 163 | 1.007 | 0.949 | 1.069 | 0.813 |
| Maximum neck width | 158 | 1.043 | 0.929 | 1.170 | 0.478 |
| Dome-to-neck ratio | 157 | 0.949 | 0.743 | 1.213 | 0.678 |
| Shape of aneurysm: |  |  |  |  |  |
| Regular | 18 | - | - | - | - |
| Irregular | 117 | 2.759 | 0.599 | 12.707 | 0.193 |
| Multilobular | 11 | 1.778 | 0.213 | 14.860 | 0.595 |
| Two domes |  |  |  |  |  |
| Other | 3 | 4.000 | 0.240 | 66.758 | 0.334 |
| Vasospasm of the parent artery | 14 | 4.429 | 1.438 | 13.642 | 0.010 |
| **Severity of aneurysmal subarachnoid hemorrhage on admission** | | | | | |
| PAASH score | 168 | 3.543 | 2.337 | 5.371 | <0.001 |
| PAASH scale: |  |  |  |  |  |
| Grade I | 80 | - | - | - | - |
| Grade II | 31 | 5.778 | 1.001 | 33.345 | 0.050 |
| Grade III | 28 | 21.667 | 4.364 | 107.569 | <0.001 |
| Grade IV | 25 | 82.875 | 16.142 | 425.481 | <0.001 |
| Grade V | 4 | 39.000 | 3.500 | 434.525 | 0.003 |
| WFNS score | 168 | 2.734 | 1.889 | 3.957 | <0.001 |
| WFNS scale: |  |  |  |  |  |
| Grade I | 80 | - | - | - | - |
| Grade II | 14 | 6.500 | 0.835 | 50.594 | 0.074 |
| Grade III | 6 | 7.800 | 0.600 | 101.415 | 0.117 |
| Grade IV | 50 | 21.937 | 4.809 | 100.074 | <0.001 |
| Grade V | 18 | 78.000 | 14.082 | 432.047 | <0.001 |
| Modified WFNS score | 168 | 2.734 | 1.889 | 3.957 | <0.001 |
| Modified WFNS scale: |  |  |  |  |  |
| Grade I | 80 | - | - | - | - |
| Grade II | 14 | 6.500 | 0.835 | 50.594 | 0.074 |
| Grade III | 6 | 7.800 | 0.600 | 101.415 | 0.117 |
| Grade IV | 50 | 21.937 | 4.809 | 100.074 | <0.001 |
| Grade V | 18 | 78.000 | 14.082 | 432.047 | <0.001 |
| Hunt and Hess score | 168 | 3.073 | 2.085 | 4.530 | <0.001 |
| Hunt and Hess scale: |  |  |  |  |  |
| Grade 1 | 39 | - | - | - | - |
| Grade 2 | 45 | 0.864 | 0.052 | 14.282 | 0.918 |
| Grade 3 | 25 | 9.500 | 1.038 | 86.968 | 0.046 |
| Grade 4 | 21 | 11.875 | 1.283 | 109.896 | 0.029 |
| Grade 5 | 38 | 58.267 | 7.211 | 470.807 | <0.001 |
| Fisher score | 168 | 3.408 | 1.273 | 9.119 | 0.015 |
| Fisher scale: |  |  |  |  |  |
| Group 2 | 13 | - | - | - | - |
| Group 1 | 1 | 1.000 | 0.000 | - | >0.999 |
| Group 3 | 34 | 215397078.7 | 0.000 | - | 0.999 |
| Group 4 | 120 | 562694615.8 | 0.000 | - | 0.999 |
| Claassen score | 168 | 2.041 | 1.194 | 3.488 | 0.009 |
| Claassen scale: |  |  |  |  |  |
| Grade 1 |  | - | - | - | - |
| Grade 2 | 22 | - | - | - | - |
| Grade 3 | 11 | 0 | 0 |  | 0.999 |
| Grade 4 | 39 | 1.471 | 0.261 | 8.298 | 0.662 |
| Grade 5 | 96 | 4.118 | 0.902 | 18.803 | 0.068 |
| **Laboratory investigations on admission** | | | | | |
| Complete blood count |  |  |  |  |  |
| Red blood cells (T/L) | 163 | 1.069 | 0.659 | 1.732 | 0.787 |
| Hemoglobin (g/L) | 163 | 1.003 | 0.985 | 1.022 | 0.731 |
| Hematocrit (L/L) | 163 | 384.478 | 0.055 | 2707244 | 0.188 |
| Platelets (G/L) | 163 | 1.001 | 0.996 | 1.006 | 0.841 |
| White blood cells (G/L) | 163 | 1.083 | 1.002 | 1.170 | 0.044 |
| Percentage of neutrophils (%) | 162 | 1.017 | 0.998 | 1.036 | 0.078 |
| Coagulation |  |  |  |  |  |
| Prothrombin time (PT) | 155 | 1.011 | 0.996 | 1.026 | 0.167 |
| Prothrombin time with INR (PT-INR) | 155 | 13.831 | 0.206 | 926.578 | 0.221 |
| Activated partial thromboplastin time (APTT) | 153 | 1.004 | 0.944 | 1.068 | 0.892 |
| rAPTT | 152 | 0.937 | 0.498 | 1.762 | 0.839 |
| Blood biochemical investigation |  |  |  |  |  |
| Ure (mmol/L) | 166 | 1.193 | 0.979 | 1.454 | 0.081 |
| Glucose (mmol/L) | 156 | 1.530 | 1.278 | 1.832 | ,0.001 |
| Glucose (mmol/L): |  |  |  |  |  |
| ≤ 6.4 | 29 | - | - | - | - |
| > 6.4 | 127 | 590603841.9 | 0 |  | 0.998 |
| Creatinine (µmol/L) | 167 | 1.015 | 1.001 | 1.030 | 0.040 |
| SGOT (UI/L) | 157 | 1.034 | 1.013 | 1.055 | 0.001 |
| SGPT (UI/L) | 165 | 1.020 | 1.003 | 1.037 | 0.022 |
| Na^+^ (mmol/L) | 167 | 1.089 | 1.005 | 1.179 | 0.037 |
| Na^+^ (mmol/L): |  |  |  |  |  |
| ≥ 135 | 128 | - | - | - | - |
| < 135 | 39 | 0.480 | 0.173 | 1.338 | 0.161 |
| K^+^ (mmol/L) | 167 | 1.060 | 0.472 | 2.382 | 0.887 |
| Cl^-^ (mmol/L) | 167 | 1.025 | 0.965 | 1.089 | 0.421 |
| **Aneurysm repairs and other treatments** | | | | | |
| No aneurysm repair | 26 | 28.222 | 9.801 | 81.270 | <0.001 |
| Endovascular coiling | 79 | 0.259 | 0.110 | 0.611 | 0.002 |
| Number of coils | 59 | 0.687 | 0.435 | 1.086 | 0.108 |
| Stent-assisted coiling | 2 | 0.000 | 0.000 |  | 0.999 |
| Balloon-assisted coiling | 9 | 0.000 | 0.000 |  | 0.999 |
| Surgical clipping | 63 | 0.344 | 0.140 | 0.843 | 0.020 |
| Number of clip attempts | 57 | 1.105 | 0.646 | 1.890 | 0.716 |
| Temporary vessel occlusion of the parent artery | 7 | 1.458 | 0.138 | 15.387 | 0.754 |
| Surgical hematoma evacuation or decompressive craniotomy | 7 | 5.591 | 1.190 | 26.274 | 0.029 |
| External ventricular drainage | 26 | 1.487 | 0.569 | 3.884 | 0.418 |
| Number of EVD | 26 | 0.000 | 0.000 |  | 0.999 |
| Intraventricular fibrinolysis | 3 | 1.926 | 0.170 | 21.881 | 0.597 |
| **Airway management and mechanical ventilation** | | | | | |
| Tracheal intubation | 144 | 518730428.7 | 0.000 |  | 0.999 |
| Mechanical ventilation | 141 | 533411720.6 | 0.000 |  | 0.999 |
| Tracheotomy | 15 | 0.927 | 0.246 | 3.492 | 0.911 |
| **Medical treatment** | | | | | |
| Nimodipine for preventing and treating cerebral vasospasm | 158 | 0.188 | 0.047 | 0.741 | 0.017 |
| Route of administration of nimodipine: |  |  |  |  |  |
| Oral | 113 | 2.273 | 0.811 | 6.368 | 0.118 |
| Intravenous | 60 | 0.343 | 0.131 | 0.896 | 0.029 |
| Deep venous thrombosis prophylaxis | 16 | 1.274 | 0.382 | 4.253 | 0.694 |
| **Complications** | | | | | |
| Rebleeding | 10 | 2.874 | 0.761 | 10.844 | 0.119 |
| Number of rebleeding sites: |  |  |  |  |  |
| Rebleeding from a single site | 4 | - | - | - | - |
| Rebleeding from multiple sites | 6 | 0.067 | 0.003 | 1.509 | 0.089 |
| Rebleeding sites: |  |  |  |  |  |
| Subarachnoid hemorrhage, (n=9) | 3 | 0.500 | 0.028 | 8.952 | 0.638 |
| Subdural hemorrhage | 4 | 0.000 | 0.000 |  | 0.999 |
| Intraventricular hemorrhage | 6 | 0.500 | 0.037 | 6.683 | 0.600 |
| Intracerebral hemorrhage | 7 | 0.200 | 0.011 | 3.661 | 0.278 |
| Vasospasm and delayed cerebral ischemia | 17 | 2.201 | 0.751 | 6.445 | 0.150 |
| Acute hydrocephalus | 76 | 2.476 | 1.149 | 5.337 | 0.021 |
| Hyponatremia | 34 | 0.776 | 0.293 | 2.053 | 0.609 |
| Seizures | 44 | 0.210 | 0.061 | 0.726 | 0.014 |
| Chronic hydrocephalus | 5 | 4.625 | 0.415 | 51.559 | 0.213 |
| EVD obstruction | 2 | 0.000 | 0.000 |  | 0.999 |
| EVD replacement | 2 | 0.000 | 0.000 |  | 0.999 |
| Ventriculitis | 8 | 0.000 | 0.000 |  | 0.999 |
| Pneumonia | 29 | 1.261 | 0.490 | 3.249 | 0.631 |
| Urinary tract infection | 3 | 0.000 | 0.000 |  | 0.999 |
| **Clinical time course** | | | | | |
| Ictus to hospital arrival (hours): |  |  |  |  |  |
| ≤ 24 hours | 69 | - | - | - | - |
| >24–72 hours | 91 | 0.817 | 0.382 | 1.748 | 0.602 |
| >72 hours | 1 | 0 | 0 |  | >0.999 |
| EVD duration (days) | 25 | 0.926 | 0.731 | 1.172 | 0.521 |
| EVD duration (days): |  |  |  |  |  |
| ≤ 7 | 17 | - | - | - | - |
| > 7 | 8 | 0.343 | 0.033 | 3.562 | 0.370 |
| Length of hospitalization (days) | 168 | 0.809 | 0.735 | 0.890 | <0.001 |

**S11 Table**. Factors associated with death within 30 days after the onset of hemorrhage in patients with aneurysmal subarachnoid hemorrhage: multivariate logistic regression analyses (backward elimination)

| Steps | Factors | Unit | OR | 95% CI for OR | | p value |
| --- | --- | --- | --- | --- | --- | --- |
|  |  |  |  | Lower | Upper |  |
| 1 | **Demographics** | | | | | |
|  | Age (years): |  |  |  |  |  |
|  | 20 - 39 | % | - | - | - | 0.638 |
|  | 40 - 59 | % | 3.566 | 0.176 | 72.261 | 0.408 |
|  | ≥ 60 | % | 4.093 | 0.222 | 75.374 | 0.343 |
|  | **Head imaging findings on admission** | | | | | |
|  | Location of blood within the subarachnoid space: |  |  |  |  |  |
|  | Basal | % | 3.426 | 0.580 | 20.226 | 0.174 |
|  | Interhemispheric fissure | % | 0.682 | 0.135 | 3.451 | 0.644 |
|  | Interpeduncular fossa | % | 1.029 | 0.149 | 7.127 | 0.977 |
|  | Ambient cistern | % | 0.544 | 0.086 | 3.432 | 0.517 |
|  | Quadrigeminal cistern | % | 5.241 | 0.640 | 42.943 | 0.123 |
|  | IVH | % | 0.322 | 0.051 | 2.031 | 0.228 |
|  | ICH | % | 0.555 | 0.094 | 3.270 | 0.515 |
|  | **Severity of aneurysmal subarachnoid hemorrhage on admission** | | | | | |
|  | WFNS scale: |  |  |  |  |  |
|  | Grade I | % | - | - | - | 0.074 |
|  | Grade II | % | 31.784 | 1.277 | 791.145 | 0.035 |
|  | Grade III | % | 41.141 | 0.751 | 2254.995 | 0.069 |
|  | Grade IV | % | 49.372 | 3.417 | 713.291 | 0.004 |
|  | Grade V | % | 74.909 | 2.731 | 2054.644 | 0.011 |
|  | **Aneurysm repairs and other treatments** | | | | | |
|  | Aneurysm repairs: |  |  |  |  |  |
|  | No aneurysm repair | % | - | - | - | 0.001 |
|  | Endovascular coiling | % | 0.017 | 0.002 | 0.158 | <0.001 |
|  | Surgical clipping | % | 0.027 | 0.004 | 0.205 | <0.001 |
|  | External ventricular drainage | % | 1.697 | 0.298 | 9.672 | 0.551 |
|  | **Medical treatment** | | | | | |
|  | Nimodipine for preventing and treating cerebral vasospasm | % | 1.232 | 0.079 | 19.122 | 0.882 |
|  | **Complications** | | | | | |
|  | Rebleeding | % | 6.919 | 0.811 | 59.020 | 0.077 |
|  | Vasospasm and delayed cerebral ischemia | % | 5.265 | 0.771 | 35.936 | 0.090 |
|  | Acute hydrocephalus | % | 1.611 | 0.309 | 8.404 | 0.572 |
|  | Constant | % | 0.030 |  |  | 0.094 |
| 2 | **Demographics** | | | | | |
|  | Age (years): |  |  |  |  |  |
|  | 20 - 39 | % | - | - | - | 0.630 |
|  | 40 - 59 | % | 3.603 | 0.192 | 67.478 | 0.391 |
|  | ≥ 60 | % | 4.117 | 0.230 | 73.804 | 0.337 |
|  | **Head imaging findings on admission** | | | | | |
|  | Location of blood within the subarachnoid space: |  |  |  |  |  |
|  | Basal | % | 3.429 | 0.582 | 20.194 | 0.173 |
|  | Interhemispheric fissure | % | 0.685 | 0.138 | 3.400 | 0.643 |
|  | Ambient cistern | % | 0.552 | 0.116 | 2.631 | 0.456 |
|  | Quadrigeminal cistern | % | 5.264 | 0.656 | 42.235 | 0.118 |
|  | IVH | % | 0.324 | 0.054 | 1.957 | 0.219 |
|  | ICH | % | 0.553 | 0.095 | 3.199 | 0.508 |
|  | **Severity of aneurysmal subarachnoid hemorrhage on admission** | | | | | |
|  | WFNS scale: |  |  |  |  |  |
|  | Grade I | % | - | - | - | 0.074 |
|  | Grade II | % | 31554. | 1.318 | 755.300 | 0.033 |
|  | Grade III | % | 40.941 | 0.758 | 2211.076 | 0.068 |
|  | Grade IV | % | 49.352 | 3.421 | 712.005 | 0.004 |
|  | Grade V | % | 74.650 | 2.752 | 2025.123 | 0.010 |
|  | **Aneurysm repairs and other treatments** | | | | | |
|  | Aneurysm repairs: |  |  |  |  |  |
|  | No aneurysm repair | % | - | - | - | <0.001 |
|  | Endovascular coiling | % | 0.017 | 0.002 | 0.157 | <0.001 |
|  | Surgical clipping | % | 0.027 | 0.004 | 0.203 | <0.001 |
|  | External ventricular drainage | % | 1.694 | 0.299 | 9.609 | 0.552 |
|  | **Medical treatment** | | | | | |
|  | Nimodipine for preventing and treating cerebral vasospasm | % | 1.223 | 0.082 | 18.173 | 0.884 |
|  | **Complications** | | | | | |
|  | Rebleeding | % | 6.960 | 0.846 | 57.273 | 0.071 |
|  | Vasospasm and delayed cerebral ischemia | % | 5.289 | 0.793 | 35.257 | 0.085 |
|  | Acute hydrocephalus | % | 1.609 | 0.309 | 8.381 | 0.572 |
|  | Constant | % | 0.030 |  |  | 0.094 |
| 3 | **Demographics** | | | | | |
|  | Age (years): |  |  |  |  |  |
|  | 20 - 39 | % | - | - | - | 0.621 |
|  | 40 - 59 | % | 3.728 | 0.203 | 68.538 | 0.376 |
|  | ≥ 60 | % | 4.210 | 0.234 | 75.834 | 0.330 |
|  | **Head imaging findings on admission** | | | | | |
|  | Location of blood within the subarachnoid space: |  |  |  |  |  |
|  | Basal | % | 3.374 | 0.584 | 19.483 | 0.174 |
|  | Interhemispheric fissure | % | 0.673 | 0.137 | 3.299 | 0.625 |
|  | Ambient cistern | % | 0.562 | 0.121 | 2.624 | 0.464 |
|  | Quadrigeminal cistern | % | 5.309 | 0.668 | 42.200 | 0.114 |
|  | IVH | % | 0.332 | 0.056 | 1.951 | 0.222 |
|  | ICH | % | 0.549 | 0.095 | 3.174 | 0.503 |
|  | **Severity of aneurysmal subarachnoid hemorrhage on admission** | | | | | |
|  | WFNS scale: |  |  |  |  |  |
|  | Grade I | % | - | - | - | 0.070 |
|  | Grade II | % | 31.622 | 1.325 | 754.908 | 0.033 |
|  | Grade III | % | 40.973 | 0.759 | 2212.311 | 0.068 |
|  | Grade IV | % | 48.517 | 3.414 | 689.513 | 0.004 |
|  | Grade V | % | 69.213 | 3.019 | 1586.522 | 0.008 |
|  | **Aneurysm repairs and other treatments** | | | | | |
|  | Aneurysm repairs: |  |  |  |  |  |
|  | No aneurysm repair | % | - | - | - | <0.001 |
|  | Endovascular coiling | % | 0.018 | 0.002 | 0.155 | <0.001 |
|  | Surgical clipping | % | 0.027 | 0.004 | 0.205 | <0.001 |
|  | External ventricular drainage | % |  |  |  |  |
|  | **Complications** | | | | | |
|  | Rebleeding | % | 7.088 | 0.872 | 57.599 | 0.067 |
|  | Vasospasm and delayed cerebral ischemia | % | 5.341 | 0.804 | 35.479 | 0.083 |
|  | Acute hydrocephalus | % | 1.635 | 0.317 | 8.429 | 0.557 |
|  | Constant | % | 0.035 |  |  | 0.067 |
| 4 | **Demographics** | | | | | |
|  | Age (years): |  |  |  |  |  |
|  | 20 - 39 | % | - | - | - | 0.640 |
|  | 40 - 59 | % | 3.465 | 0.199 | 60.436 | 0.394 |
|  | ≥ 60 | % | 3.890 | 0.231 | 65.387 | 0.345 |
|  | **Head imaging findings on admission** | | | | | |
|  | Location of blood within the subarachnoid space: |  |  |  |  |  |
|  | Basal | % | 2.885 | 0.553 | 15.050 | 0.209 |
|  | Ambient cistern | % | 0.616 | 0.139 | 2.734 | 0.524 |
|  | Quadrigeminal cistern | % | 4.774 | 0.640 | 35.639 | 0.127 |
|  | IVH | % | 0.344 | 0.059 | 1.993 | 0.234 |
|  | ICH | % | 0.631 | 0.120 | 3.318 | 0.587 |
|  | **Severity of aneurysmal subarachnoid hemorrhage on admission** | | | | | |
|  | WFNS scale: |  |  |  |  |  |
|  | Grade I | % | - | - | - | 0.075 |
|  | Grade II | % | 27.707 | 1.226 | 626.081 | 0.037 |
|  | Grade III | % | 32.355 | 0.673 | 1555.355 | 0.078 |
|  | Grade IV | % | 43.592 | 3.186 | 596.444 | 0.005 |
|  | Grade V | % | 58.867 | 2.790 | 1242.048 | 0.009 |
|  | **Aneurysm repairs and other treatments** | | | | | |
|  | Aneurysm repairs: |  |  |  |  |  |
|  | No aneurysm repair | % | - | - | - | <0.001 |
|  | Endovascular coiling | % | 0.020 | 0.003 | 0.160 | <0.001 |
|  | Surgical clipping | % | 0.031 | 0.004 | 0.215 | <0.001 |
|  | External ventricular drainage | % | 1.658 | 0.300 | 9.166 | 0.562 |
|  | **Complications** | | | | | |
|  | Rebleeding | % | 6.961 | 0.853 | 56.810 | 0.070 |
|  | Vasospasm and delayed cerebral ischemia | % | 5.282 | 0.804 | 34.713 | 0.083 |
|  | Acute hydrocephalus | % | 1.438 | 0.302 | 6.836 | 0.648 |
|  | Constant | % | 0.034 |  |  | 0.064 |
| 5 | **Demographics** | | | | | |
|  | Age (years): |  |  |  |  |  |
|  | 20 - 39 | % | - | - | - | 0.587 |
|  | 40 - 59 | % | 3.632 | 0.209 | 63.284 | 0.376 |
|  | ≥ 60 | % | 4.366 | 0.264 | 72.237 | 0.303 |
|  | **Head imaging findings on admission** | | | | | |
|  | Location of blood within the subarachnoid space: |  |  |  |  |  |
|  | Basal | % | 3.177 | 0.638 | 15.816 | 0.158 |
|  | Ambient cistern | % | 0.625 | 0.141 | 2.771 | 0.537 |
|  | Quadrigeminal cistern | % | 4.965 | 0.671 | 36.718 | 0.116 |
|  | IVH | % | 0.346 | 0.060 | 2.002 | 0.236 |
|  | ICH | % | 0.640 | 0.124 | 3.301 | 0.594 |
|  | **Severity of aneurysmal subarachnoid hemorrhage on admission** | | | | | |
|  | WFNS scale: |  |  |  |  |  |
|  | Grade I | % | - | - | - | 0.069 |
|  | Grade II | % | 27.200 | 1.228 | 602.380 | 0.037 |
|  | Grade III | % | 30.370 | 0.688 | 1340.062 | 0.077 |
|  | Grade IV | % | 43.910 | 3.333 | 578.437 | 0.004 |
|  | Grade V | % | 53.056 | 2.752 | 1022.707 | 0.009 |
|  | **Aneurysm repairs and other treatments** | | | | | |
|  | Aneurysm repairs: |  |  |  |  |  |
|  | No aneurysm repair | % | - | - | - | <0.001 |
|  | Endovascular coiling | % | 0.019 | 0.002 | 0.157 | <0.001 |
|  | Surgical clipping | % | 0.030 | 0.004 | 0.207 | <0.001 |
|  | External ventricular drainage | % | 1.847 | 0.351 | 9.727 | 0.469 |
|  | **Complications** | | | | | |
|  | Rebleeding | % | 7.699 | 0.978 | 60.584 | 0.052 |
|  | Vasospasm and delayed cerebral ischemia | % | 4.831 | 0.772 | 30.218 | 0.092 |
|  | Constant | % | 0.037 |  |  | 0.066 |
| 6 | **Demographics** | | | | | |
|  | Age (years): |  |  |  |  |  |
|  | 20 - 39 | % | - | - | - | 0.624 |
|  | 40 - 59 | % | 2.898 | 0.201 | 41.846 | 0.435 |
|  | ≥ 60 | % | 3.683 | 0.259 | 52.415 | 0.336 |
|  | **Head imaging findings on admission** | | | | | |
|  | Location of blood within the subarachnoid space: |  |  |  |  |  |
|  | Basal | % | 2.943 | 0.618 | 14.014 | 0.175 |
|  | Ambient cistern | % | 0.652 | 0.147 | 2.886 | 0.573 |
|  | Quadrigeminal cistern | % | 5.332 | 0.737 | 38.563 | 0.097 |
|  | IVH | % | 0.374 | 0.068 | 2.058 | 0.258 |
|  | **Severity of aneurysmal subarachnoid hemorrhage on admission** | | | | | |
|  | WFNS scale: |  |  |  |  |  |
|  | Grade I | % | - | - | - | 0.074 |
|  | Grade II | % | 23.423 | 1.141 | 480.950 | 0.041 |
|  | Grade III | % | 25.128 | 0.581 | 1085.991 | 0.093 |
|  | Grade IV | % | 39.702 | 3.151 | 500.249 | 0.004 |
|  | Grade V | % | 45.329 | 2.537 | 809.964 | 0.010 |
|  | **Aneurysm repairs and other treatments** | | | | | |
|  | Aneurysm repairs: |  |  |  |  |  |
|  | No aneurysm repair | % | - | - | - | <0.001 |
|  | Endovascular coiling | % | 0.023 | 0.003 | 0.164 | <0.001 |
|  | Surgical clipping | % | 0.032 | 0.005 | 0.212 | <0.001 |
|  | External ventricular drainage | % | 1.971 | 0.379 | 10.261 | 0.420 |
|  | **Complications** | | | | | |
|  | Rebleeding | % | 7.396 | 0.939 | 58.281 | 0.057 |
|  | Vasospasm and delayed cerebral ischemia | % | 5.075 | 0.828 | 31.091 | 0.079 |
|  | Constant | % | 0.037 |  |  | 0.065 |
| 7 | **Head imaging findings on admission** | | | | | |
|  | Location of blood within the subarachnoid space: |  |  |  |  |  |
|  | Basal | % | 3.237 | 0.689 | 15.205 | 0.137 |
|  | Ambient cistern | % | 0.544 | 0.128 | 2.311 | 0.410 |
|  | Quadrigeminal cistern | % | 6.456 | 0.911 | 45.742 | 0.062 |
|  | IVH | % | 0.404 | 0.078 | 2.084 | 0.279 |
|  | **Severity of aneurysmal subarachnoid hemorrhage on admission** | | | | | |
|  | WFNS scale: |  |  |  |  |  |
|  | Grade I | % | - | - | - | 0.071 |
|  | Grade II | % | 21.503 | 1.102 | 419.754 | 0.043 |
|  | Grade III | % | 27.804 | 0.666 | 1161.296 | 0.081 |
|  | Grade IV | % | 40.325 | 3.260 | 498.798 | 0.004 |
|  | Grade V | % | 41.456 | 2.419 | 710.575 | 0.010 |
|  | **Aneurysm repairs and other treatments** | | | | | |
|  | Aneurysm repairs: |  |  |  |  |  |
|  | No aneurysm repair | % | - | - | - | <0.001 |
|  | Endovascular coiling | % | 0.023 | 0.003 | 0.159 | <0.001 |
|  | Surgical clipping | % | 0.032 | 0.005 | 0.205 | <0.001 |
|  | External ventricular drainage | % | 2.091 | 0.412 | 10.624 | 0.374 |
|  | **Complications** | | | | | |
|  | Rebleeding | % | 8.480 | 1.133 | 63.481 | 0.037 |
|  | Vasospasm and delayed cerebral ischemia | % | 5.437 | 0.887 | 33.312 | 0.067 |
|  | Constant | % | 0.102 |  |  | 0.104 |
| 8 | **Head imaging findings on admission** | | | | | |
|  | Location of blood within the subarachnoid space: |  |  |  |  |  |
|  | Basal | % | 2.831 | 0.623 | 12.856 | 0.178 |
|  | Quadrigeminal cistern | % | 4.810 | 0.786 | 29.431 | 0.089 |
|  | IVH | % | 0.417 | 0.083 | 2.104 | 0.290 |
|  | **Severity of aneurysmal subarachnoid hemorrhage on admission** | | | | | |
|  | WFNS scale: |  |  |  |  |  |
|  | Grade I | % | - | - | - | 0.084 |
|  | Grade II | % | 23.366 | 1.214 | 449.705 | 0.037 |
|  | Grade III | % | 30.438 | 0.777 | 1193.106 | 0.068 |
|  | Grade IV | % | 38.792 | 3.024 | 497.693 | 0.005 |
|  | Grade V | % | 39.818 | 2.262 | 700.934 | 0.012 |
|  | **Aneurysm repairs and other treatments** | | | | | |
|  | Aneurysm repairs: |  |  |  |  |  |
|  | No aneurysm repair | % | - | - | - | <0.001 |
|  | Endovascular coiling | % | 0.025 | 0.004 | 0.170 | <0.001 |
|  | Surgical clipping | % | 0.034 | 0.005 | 0.214 | <0.001 |
|  | External ventricular drainage | % | 1.887 | 0.377 | 9.449 | 0.440 |
|  | **Complications** | | | | | |
|  | Rebleeding | % | 7.955 | 1.075 | 58.884 | 0.042 |
|  | Vasospasm and delayed cerebral ischemia | % | 5.501 | 0.868 | 34.857 | 0.070 |
|  | Constant | % | 0.081 |  |  | 0.074 |
| 9 | **Head imaging findings on admission** | | | | | |
|  | Location of blood within the subarachnoid space: |  |  |  |  |  |
|  | Basal | % | 3.116 | 0.692 | 14.024 | 0.139 |
|  | Quadrigeminal cistern | % | 4.494 | 0.753 | 26.835 | 0.099 |
|  | IVH | % | 0.414 | 0.083 | 2.068 | 0.283 |
|  | **Severity of aneurysmal subarachnoid hemorrhage on admission** | | | | | |
|  | WFNS scale: |  |  |  |  |  |
|  | Grade I | % | - | - | - | 0.071 |
|  | Grade II | % | 24.011 | 1.315 | 438.394 | 0.032 |
|  | Grade III | % | 22.466 | 0.628 | 803.372 | 0.088 |
|  | Grade IV | % | 39.886 | 3.276 | 485.675 | 0.004 |
|  | Grade V | % | 41.668 | 2.438 | 712.094 | 0.010 |
|  | **Aneurysm repairs and other treatments** | | | | | |
|  | Aneurysm repairs: |  |  |  |  |  |
|  | No aneurysm repair | % | - | - | - | <0.001 |
|  | Endovascular coiling | % | 0.031 | 0.005 | 0.189 | <0.001 |
|  | Surgical clipping | % | 0.040 | 0.007 | 0.238 | <0.001 |
|  | **Complications** | | | | | |
|  | Rebleeding | % | 9.525 | 1.353 | 67..063 | 0.024 |
|  | Vasospasm and delayed cerebral ischemia | % | 5.134 | 0.837 | 31.496 | 0.077 |
|  | Constant | % | 0.077 |  |  | 0.068 |
| 10 | **Head imaging findings on admission** | | | | | |
|  | Location of blood within the subarachnoid space: |  |  |  |  |  |
|  | Basal | % | 2.597 | 0.618 | 10.906 | 0.192 |
|  | Quadrigeminal cistern | % | 4.040 | 0.683 | 23.898 | 0.124 |
|  | **Severity of aneurysmal subarachnoid hemorrhage on admission** | | | | | |
|  | WFNS scale: |  |  |  |  |  |
|  | Grade I | % | - | - | - | 0.094 |
|  | Grade II | % | 21.638 | 1.268 | 369.247 | 0.034 |
|  | Grade III | % | 24.897 | 0.818 | 758.037 | 0.065 |
|  | Grade IV | % | 27.468 | 2.596 | 290.605 | 0.006 |
|  | Grade V | % | 32.860 | 2.117 | 510.064 | 0.013 |
|  | **Aneurysm repairs and other treatments** | | | | | |
|  | Aneurysm repairs: |  |  |  |  |  |
|  | No aneurysm repair | % | - | - | - | <0.001 |
|  | Endovascular coiling | % | 00.37 | 0.007 | 0.208 | <0.001 |
|  | Surgical clipping | % | 0.043 | 0.007 | 0.245 | <0.001 |
|  | **Complications** | | | | | |
|  | Rebleeding | % | 9.316 | 1.374 | 63.192 | 0.022 |
|  | Vasospasm and delayed cerebral ischemia | % | 5.567 | 0.940 | 32.964 | 0.058 |
|  | Constant | % | 0.053 |  |  | 0.029 |
| 11 | **Head imaging findings on admission** | | | | | |
|  | Location of blood within the subarachnoid space: |  |  |  |  |  |
|  | Quadrigeminal cistern | % | 5.958 | 1.155 | 30.730 | 0.033 |
|  | **Severity of aneurysmal subarachnoid hemorrhage on admission** | | | | | |
|  | WFNS scale: |  |  |  |  |  |
|  | Grade I | % | - | - | - | 0.098 |
|  | Grade II | % | 20.290 | 1.218 | 338.093 | 0.036 |
|  | Grade III | % | 30.342 | 1.055 | 872.794 | 0.046 |
|  | Grade IV | % | 26.787 | 2.599 | 276.094 | 0.006 |
|  | Grade V | % | 24.131 | 1.705 | 341.556 | 0.019 |
|  | **Aneurysm repairs and other treatments** | | | | | |
|  | Aneurysm repairs: |  |  |  |  |  |
|  | No aneurysm repair | % | - | - | - | <0.001 |
|  | Endovascular coiling | % | 0.045 | 0.009 | 0.228 | <0.001 |
|  | Surgical clipping | % | 0.038 | 0.007 | 0.227 | <0.001 |
|  | **Complications** | | | | | |
|  | Rebleeding | % | 10.153 | 1.557 | 66.219 | 0.015 |
|  | Vasospasm and delayed cerebral ischemia | % | 6.602 | 1.127 | 38.673 | 0.036 |
|  | Constant | % | 0.082 |  |  | 0.055 |
