# Supplementary material for: Clinical characteristics and factors relating to poor outcome in patients with aneurysmal subarachnoid hemorrhage in Vietnam: A multicenter prospective cohort study
Source: PLoS One. 2021 Aug 13;16(8):e0256150. doi: 10.1371/journal.pone.0256150 (PMC8362943; doi:10.1371/journal.pone.0256150)
Supplement: S2 File — (PDF) [file pone.0256150.s003.pdf]

**MINISTRY OF HEALTH**<br>**VIET DUC HOSPITAL**<br>No: 818/QĐ–VĐ	**SOCIALIST REPUBLIC OF VIETNAM**<br>**Independence - Freedom - Happiness**<br>*Hanoi, May 4, 2020*

DECISION OF DIRECTOR OF VIET DUC HOSPITAL

On: Approval for the implementation of the 2020 institutional-level scientific research projects in the Viet Duc Hospital

DIRECTOR OF VIET DUC HOSPITAL

- Pursuant to Decision No. 1184/QĐ-BYT dated 29 March 2019 by the Minister of Health about issuing the Regulation of Organization and Operation of Viet Duc Hospital;
- Pursuant to the meeting dated 27 March 2020 of Standing Board of the Scientific Committee and Ethics Committee in Viet Duc Hospital;
- Based on request of the Head of Scientific Research Department - Viet Duc Hospital.

IT IS TO DECIDE

Article I: To approve 112 institutional-level scientific research projects in the Viet Duc Hospital, including:

1. 30 scientific research projects regarding neurology, anesthesia and critical care medicine, laboratory, and other fields
2. 33 scientific research projects regarding cardiology, trauma
3. 19 scientific research projects regarding gastroenterology, urology
4. 30 scientific research projects regarding medical nursing

A list of the 2020 institutional-level scientific research projects is attached to this decision.

Article II: The Head of research will be responsible for implementing and submitting for approval of the research on schedule as approved and complying with the state regulations on technology agreements and financial expenditure.

Article III: Heads of Scientific Research Department, Integrated Planning Department, Finance-Accounting Department, Leaders of relevant units and the head of each scientific research project above in Article I take responsibilities to implement this Decision. This Decision takes effect from the signing date./.

DIRECTOR OF VIET DUC HOSPITAL

Recipients:
- *As Article III;*
- *Saved at Scientific Research and Information Technology Department;*
- *Archived at managerial administration (archival division).*

Prof. Tran Binh Giang, MD., PhD.

MINISTRY OF HEALTH
VIET DUC HOSPITAL

THE LIST OF THE 2020 INSTITUTIONAL-LEVEL SCIENTIFIC RESEARCH PROJECTS IN THE VIET DUC HOSPITAL

(Decision No. 818/QĐ–VĐ dated May 4, 2020 by Director of Viet Duc Hospital is attached to this list)

**No.**	**Research code**	**Title**	**Head of scientific research project/ implementing unit**	**Duration of research project**	**Expenditure**
1					
2					
3					
4	KH04.2020	Study on the prognostic value of PAASH grading scale and factors relating to poor outcome on patients with aneurysmal subarachnoid hemorrhage	Ngo Manh Hung/ Department of Neurosurgery II	From September 2019 to September 2020	5,000,000 VND
5					
6					
7					
8					
9					

BỘ Y TẾ
BỆNH VIỆN HỮU NGHỊ VIỆT ĐỨC

Số: 818/QĐ – VĐ

CỘNG HÒA XÃ HỘI CHỦ NGHĨA VIỆT NAM
Độc lập – Tự do – Hạnh phúc

Hà nội, ngày 04 tháng 5 năm 2020

QUYẾT ĐỊNH

Phê duyệt triển khai đề tài khoa học công nghệ cấp cơ sở năm 2020 tại Bệnh viện Hữu nghị Việt Đức

GIÁM ĐỐC BỆNH VIỆN HỮU NGHỊ VIỆT ĐỨC

Căn cứ Quyết định số 1184/QĐ – BYT ngày 29/3/2019 của Bộ trưởng Bộ Y tế về việc ban hành Quy chế tổ chức và hoạt động của Bệnh viện HN Việt Đức trực thuộc Bộ Y tế;

Căn cứ Biên bản họp Hội đồng KHCN xét duyệt đề cương đề tài cơ sở năm 2020 ngày 27/3/2020;

Căn cứ Bản hoàn thiện đề cương đề tài cấp cơ sở năm 2020;

Xét đề nghị của Trưởng phòng Nghiên cứu khoa học

QUYẾT ĐỊNH:

Điều 1: Phê duyệt triển khai 112 đề tài khoa học công nghệ cấp cơ sở năm 2020 tại Bệnh viện Hữu nghị Việt Đức. Gồm:

- 30 đề tài thuộc chuyên ngành Thần kinh, Gây mê hồi sức, Cận lâm sàng và một số lĩnh vực khác.

- 33 đề tài thuộc chuyên ngành Tim mạch, Chấn thương.

- 19 đề tài thuộc chuyên ngành Tiêu hóa, Tiết niệu.

- 30 đề tài thuộc chuyên ngành Điều dưỡng.

Danh sách đề tài KHCN cấp cơ sở thực hiện năm 2020 đính kèm theo Quyết định này.

Điều 2: Chủ nhiệm đề tài và nhóm nghiên cứu có trách nhiệm triển khai nghiên cứu đề tài theo đúng Thuyết minh đề tài đã được Giám đốc phê duyệt; tuân thủ các quy định của Nhà nước, Bộ Y tế và Bệnh viện HN Việt Đức về quản lý, tổ chức, thực hiện đề tài khoa học công nghệ; quyết toán kinh phí phù hợp với nội dung chi và đảm bảo đúng tiến độ.

Điều 3: Các ông (bà) Trưởng phòng NCKH, KHTH, TCKT, Lãnh đạo đơn vị thực hiện nghiên cứu đề tài, Chủ nhiệm đề tài chịu trách nhiệm thi hành Quyết định này.

Nơi nhận:
- Như điều 3;
- Lưu VT, NCKH.

GIÁM ĐỐC

BỆNH VIỆN HỮU NGHỊ VIỆT ĐỨC

Trần Bình Giang

BỘ Y TẾ
BỆNH VIỆN HN VIỆT ĐỨC

DANH SÁCH ĐỀ TÀI KHCN CẤP CƠ SỞ THỰC HIỆN TẠI BỆNH VIỆN HỮU NGHỊ VIỆT ĐỨC NĂM 2020

(Kèm theo Quyết định số 818/QĐ-VĐ ngày 04/5/2020 của Giám đốc bệnh viện)

STT	Mã số đề tài	Tên đề tài	Chủ nhiệm đề tài/ Đơn vị thực hiện	Thời gian thực hiện	Kinh phí từ nguồn bệnh viện
**CHUYÊN NGÀNH THẦN KINH, GMHS, CẬN LÂM SÀNG VÀ MỘT SỐ LĨNH VỰC KHÁC**					
1	KH01.2020	Đánh giá kết quả phẫu thuật đặt điện cực kích thích não sâu điều trị bệnh lý Parkinson vô căn tại Bệnh viện Việt Đức	Đồng Văn Hệ, Trần Đình Văn/ Khoa PT Thần kinh 1	T4/2020 – T4/2021	**05 triệu đồng**
2	KH02.2020	Đánh giá kết quả xa sau điều trị vi phẫu thuật giải ép mạch máu thần kinh trong chứng co giật nửa mặt	Dương Đại Hà/ Khoa PT Thần kinh 1	T4/2020 – T4/2021	**05 triệu đồng**
3	KH03.2020	Đánh giá kết quả áp dụng chụp cộng hưởng từ 3.0 Tesla xác định bó tháp trong phẫu thuật u não vùng chức năng tại Bệnh viện Việt Đức	Dương Đại Hà/ Khoa PT Thần kinh 1	T4/2020 – T4/2021	**05 triệu đồng**
4	KH04.2020	Nghiên cứu giá trị tiên lượng của thang điểm PAASH và một số yếu tố liên quan tới kết cục chức năng thần kinh xấu ở bệnh nhân chảy máu dưới nhện do vỡ phình động mạch não	Ngô Mạnh Hùng/ PT Thần kinh II	T09/2019 – T9/2020	**05 triệu đồng**
5	KH05.2020	Đánh giá chất lượng cuộc sống của người bệnh mắc bệnh động kinh tại khoa Nội – Hồi sức Thần kinh, BV HN Việt Đức năm 2020	Nguyễn Anh Tuấn/ Khoa Nội – Hồi sức thần kinh, BV HN Việt Đức	T1/2020 – T12/2020	**05 triệu đồng**
6	KH06.2020	Đặc điểm lâm sàng và điện sinh lý trong tổn thương thần kinh quay	Nguyễn Anh Tuấn/ Khoa Nội – Hồi sức thần kinh, BV HN Việt Đức	T3/2020 – T3/2021	**05 triệu đồng**
7	KH07.2020	Đánh giá sự hiểu biết của người bệnh ngoại khoa về chuyên ngành gây mê hồi sức	Nguyễn Thị Thúy Ngân/ Khoa Gây mê 1, Trung tâm GMHS	T4/2020 – T4/2021	**05 triệu đồng**
8	KH08.2020	Gây mê cho ghép hai phổi có ECMO hỗ trợ	Trịnh Kế Điệp, Nguyễn Thị Thúy Ngân/ Trung tâm GMHS	T4/2020 – T4/2021	**05 triệu đồng**
9	KH09.2020	So sánh hiệu quả vô cảm, giảm đau sau mổ của levobupivacain với ropivacain trong phong bế liên tục đám rối thần kinh cánh tay đường nách có siêu âm hướng dẫn	Nguyễn Đắc Thanh/ Khoa Gây mê 1	T4/2020 – T10/2020	**05 triệu đồng**

10	KH10.2020	Đánh giá ảnh hưởng của dung dịch tinh thể cân bằng Ringerfundin lên tình trạng điện giải, toan kiềm và chức năng thận giai đoạn sớm sau ghép thận	Trịnh Thị Thơm,<br>Nguyễn Thị Thu Yến,<br>Nguyễn Thị Nhâm/<br>Khoa GMHS 2,<br>TT Gây mê HS	T2/2020 – T10/2020	**05 triệu đồng**
11	KH11.2020	Đánh giá hiệu quả của Ondansetron liều 8mg trong dự phòng, một số tác dụng không mong muốn của gây tê tủy sống trong phẫu thuật chi dưới	Hoàng Thị Thu Hà/<br>TT Gây mê Hồi sức	T3/2020 – T9/2020	**05 triệu đồng**
12	KH12.2020	Đánh giá hiệu quả giải giãn cơ Sugamadex so với Neostigmin trong phẫu thuật nội soi u tuyến thượng thận	Phạm Thị Vân Anh,<br>Trịnh Xuân Khánh/<br>Khoa GMHS 2,<br>TT Gây mê HS	T7/2019 – T7/2020	**05 triệu đồng**
13	KH13.2020	Tình tình tai nạn giao thông liên quan đến sử dụng rượu bia sau một năm thực hiện Nghị định 100 của Thủ tướng chính phủ	Ngô Xuân Tiệp,<br>Phạm Hải Bằng/<br>Khoa Khám bệnh	T01/2020 – T02/2021	**05 triệu đồng**
14	KH14.2020	Khảo sát thực trạng chuyển viện từ bệnh viện tuyến dưới đến khoa Cấp cứu Bệnh viện HN Việt Đức năm 2020	Ngô Xuân Tiệp,<br>Phạm Hải Bằng/<br>Khoa Khám bệnh	T05/2020 – T11/2020	**05 triệu đồng**
15	KH15.2020	Nghiên cứu đặc điểm hình ảnh cộng hưởng từ cột sống và đánh giá hiệu quả phương pháp phong bế điều trị đau rễ thần kinh thắt lưng cùng	Nguyễn Đình Minh,<br>Dương Anh Dũng/<br>Khoa Chẩn đoán hình ảnh	T3/2020 – T9/2020	**05 triệu đồng**
16	KH16.2020	Nghiên cứu đặc điểm hình ảnh và giá trị của cộng hưởng từ trong chẩn đoán u trong ống sống	Trịnh Anh Tuấn,<br>Nguyễn Đình Minh/<br>Khoa Chẩn đoán hình ảnh	T02/2020 – T10/2020	**05 triệu đồng**
17	KH17.2020	Đánh giá kết quả chụp cộng hưởng từ động học sàn chậu ở người trưởng thành không triệu chứng	Nguyễn Ngọc Ánh,<br>Nguyễn Thị Mến/<br>TT PT ĐTT và TSM	T4/2020 – T7/2020	**05 triệu đồng**
18	KH18.2020	Phân tích tình hình báo cáo phản ứng có hại của thuốc tại Bệnh viện HN Việt Đức giai đoạn 2014 – 2019	Đàm Mai Hương/<br>Khoa Dược	T2/2020 – T2/2021	**05 triệu đồng**
19	KH19.2020	Đánh giá kết quả xây dựng danh mục tương tác thuốc cần chú ý trong thực hành lâm sàng tại bệnh viện HN Việt Đức	Nguyễn Thanh Hiền/<br>Khoa Dược	T1/2020 – T12/2020	**05 triệu đồng**

20	KH20.2020	Phân loại carbapenemase và tìm hiểu kiểu cách đề kháng của các chủng *Klebsiella pneumoniae* kháng carbapenemase tại khoa Hồi sức tích cực – Bệnh viện HN Việt Đức	Trần Hải Yến/<br>Khoa Vi sinh	T4/2020 –<br>T5/2021	**05 triệu đồng**
21	KH21.2020	Nhận xét đặc điểm mô gân đồng loài được bảo quản lạnh sâu tại Ngân hàng Mô – Bệnh viện HN Việt Đức từ tháng 3/2018 đến tháng 3/2020	Trần Thị Hằng,<br>Dương Công Nguyên/<br>Ngân hàng Mô	T4/2020 –<br>T9/2020	**05 triệu đồng**
22	KH22.2020	Đặc điểm mô xương sọ tự thân được bảo quản lạnh sâu tại Ngân hàng Mô – Bệnh viện Hữu nghị Việt Đức từ tháng 3/2018 đến tháng 3/2020	Trần Thị Hằng/<br>Ngân hàng Mô	T3/2020 –<br>T10/2020	**05 triệu đồng**
23	KH23.2020	Tình trạng dinh dưỡng cán bộ công nhân viên Bệnh viện Hữu nghị Việt Đức năm 2019	Đỗ Tất Thành/<br>Khoa Dinh Dưỡng	T10/2019 –<br>T10/2020	**05 triệu đồng**
24	KH24.2020	Nghiên cứu xây dựng giá ghép gan	Trịnh Hồng Sơn,<br>Đỗ Thu Hà/<br>Phòng Tài chính Kế toán	T3/2020 –<br>T6/2020	**10 triệu đồng**
25	KH25.2020	Quy trình giải quyết chế độ BHYT cho người bệnh cấp cứu vào điều trị nội trú	Lê Minh Thu,<br>Đỗ Thu Hà/<br>Phòng Tài chính kế toán	T5/2020 –<br>T5/2021	**10 triệu đồng**
26	KH26.2020	Ứng dụng công nghệ thông tin trong quản lý hồ sơ cán bộ viên chức, người lao động	Nguyễn Mạnh Khánh/<br>Phòng Tổ chức cán bộ	T4/2020 –<br>T3/2021	**10 triệu đồng**
27	KH27.2020	Thực trạng nhiễm khuẩn vết mổ và một số yếu tố liên quan tại các khoa Chấn thương chỉnh hình bệnh viện Việt Đức năm 2020	Lê Tư Hoàng/<br>Phòng Quản lý chất lượng	T7/2020 –<br>T9/2020	**05 triệu đồng**
28	KH28.2020	Khảo sát sự hài lòng và một số yếu tố liên quan của người bệnh nội trú tại bệnh viện Hữu nghị Việt Đức năm 2020	Lê Thị Kim Nhung/<br>Phòng Quản lý chất lượng	T3/2020 –<br>T7/2020	**05 triệu đồng**
29	KH29.2020	Hiệu quả hợp tác y tế với một số bệnh viện Đài Loan 2018 - 2019	Nguyễn Đức Chính/<br>Phòng Hợp tác quốc tế	T3/2020 –<br>T3/2021	**05 triệu đồng**
30	KH30.2020	Nghiên cứu thực trạng và đề xuất giải pháp triển khai công tác hỗ trợ người bệnh khám theo yêu cầu tại Bệnh viện HN Việt Đức	Nguyễn Trọng Sơn/<br>Phòng Công tác xã hội	T4/2020 –<br>T4/2021	**05 triệu đồng**

CHUYÊN NGÀNH TIM MẠCH, CHẤN THƯƠNG

STT	Mã số đề tài	Tên đề tài	Chủ nhiệm đề tài/ Đơn vị thực hiện	Thời gian thực hiện	Kinh phí từ nguồn bệnh viện
1	CT01.2020	Kết quả ứng dụng của siêu âm tim cho bệnh nhân trước và sau ghép tim	Nguyễn Hữu Ước,<br>Khổng Tiến Bình/<br>TT Tim mạch và LN	T01/2020 – T11/2020	**05 triệu đồng**
2	CT02.2020	Ứng dụng kỹ thuật nội soi phế quản trong ghép phổi từ người cho chết não tại Bệnh viện Hữu nghị Việt Đức	Nguyễn Hữu Ước,<br>Vũ Văn Thời/<br>TT Tim mạch và LN	T01/2020 – T12/2020	**05 triệu đồng**
3	CT03.2020	Kết quả ứng dụng phương pháp cố định xương sườn gãy bằng nẹp vis tại bệnh viện Hữu nghị Việt Đức	Nguyễn Hữu Ước,<br>Nguyễn Việt Anh/<br>TT Tim mạch và LN	T01/2020 – T10/2020	**05 triệu đồng**
4	CT04.2020	Kết quả ứng dụng phương pháp Hybrid điều trị bệnh lý động mạch chủ	Nguyễn Hữu Ước,<br>Nguyễn Tùng Sơn/<br>TT Tim mạch và LN	T01/2020 – T10/2020	**10 triệu đồng**
5	CT05.2020	Kết quả bước đầu trong hồi sức tích cực, điều trị và theo dõi sau ghép phổi tại Trung tâm Tim mạch và Lồng ngực, BV HN Việt Đức	Phạm Tiến Quân/<br>Khoa HSTC Tim mạch và LN,<br>TT Tim mạch và LN	T1/2020 – T12/2020	**05 triệu đồng**
6	CT06.2020	Nghiên cứu ứng dụng các khuyến cáo phòng ngừa viêm phổi thở máy ở bệnh nhân sau mổ tim hở tại khoa HSTC Tim mạch và Lồng ngực, TT Tim mạch và Lồng ngực, Bệnh viện HN Việt Đức	Phạm Tiến Quân/<br>Khoa HSTC Tim mạch và LN,<br>TT Tim mạch và Lồng ngực	T1/2020 – T12/2020	**05 triệu đồng**
7	CT07.2020	Kết quả phẫu thuật điều trị u tuyến ức tại Bệnh viện HN Việt Đức giai đoạn 2016 – 2020	Phạm Hữu Lư/<br>TT Tim mạch và Lồng ngực	T4/2020 – T4/2021	**05 triệu đồng**
8	CT08.2020	Kết quả điều trị ung thư phổi không tế bào nhỏ bằng phẫu thuật nội soi tại Bệnh viện Hữu nghị Việt Đức	Phạm Hữu Lư/<br>TT Tim mạch và Lồng ngực	T4/2020 – T4/2021	**05 triệu đồng**
9	CT09.2020	Kết quả phẫu thuật đóng thông liên nhĩ ít xâm lấn qua đường mở ngực phải không ngừng tim tại bệnh viện Hữu nghị Việt Đức	Phùng Duy Hồng Sơn/<br>TT Tim mạch và Lồng ngực	T3/2020 – T12/2020	**05 triệu đồng**
10	CT10.2020	Kết quả điều trị chấn thương động mạnh khoeo tại Bệnh viện Hữu nghị Việt Đức.	Nguyễn Hữu Ước,<br>Dương Ngọc Thắng/<br>Khoa PT Tim mạch và LN	T02/2020 – T10/2020	**05 triệu đồng**

11	CT11.2020	Kết quả ứng dụng phương pháp can thiệp nhiệt nội mạch bằng Laser trong điều trị bệnh lý suy van tĩnh mạch nông chi dưới	Lê Nhật Tiên,<br>Nguyễn Hữu Ước/<br>TT Tim mạch và LN	T01/2020 – T10/2020	**05 triệu đồng**
12	CT12.2020	Đánh giá kết quả nội soi khâu chóp xoay khớp vai bằng kỹ thuật hai hàng.	Nguyễn Mạnh Khánh,<br>Lưu Danh Huy/<br>Khoa PT Chi trên và YHTT	T4/2020 – T4/2021	**05 triệu đồng**
13	CT13.2020	Đánh giá kết quả điều trị trật cùng đòn sử dụng Tightrope.	Nguyễn Mạnh Khánh,<br>Đỗ Văn Hải/<br>Khoa PT Chi trên và YHTT	T4/2020 – T4/2021	**05 triệu đồng**
14	CT14.2020	Kết quả phẫu thuật thay khớp liên đốt gần bàn tay điều trị di chứng sau chấn thương, vết thương bàn tay	Nguyễn Mạnh Khánh,<br>Đỗ Văn Hải/<br>Khoa PT Chi trên và YHTT	T4/2020 – T4/2021	**05 triệu đồng**
15	CT15.2020	Đánh giá kết quả thay khớp háng toàn phần không xi măng hai chuyển động	Nguyễn Mạnh Khánh,<br>Đỗ Vũ Anh/<br>Khoa PT Chi trên và YHTT	T4/2020 – T4/2021	**05 triệu đồng**
16	CT16.2020	Kết quả điều trị trật đài quay trong gãy cũ monteggia trẻ em.	Lưu Danh Huy,<br>Phạm Ngọc Đình/<br>Khoa PT Chi trên và YHTT	T4/2020 – T4/2021	**05 triệu đồng**
17	CT17.2020	Đánh giá kết quả phẫu thuật chuyển gân duỗi riêng ngón II cho duỗi dài ngón I trong tổn thương đến muộn.	Lưu Danh Huy,<br>Nguyễn Mộc Sơn/<br>Khoa PT Chi trên và YHTT	T4/2020 – T4/2021	**05 triệu đồng**
18	CT18.2020	Nghiên cứu ứng dụng phẫu thuật đường bên (XLIF) điều trị vẹo cột sống thắt lưng do thoái hóa	Đinh Ngọc Sơn/<br>Khoa Phẫu thuật Cột sống	T4/2020 – T4/2021	**05 triệu đồng**
19	CT19.2020	Kết quả phẫu thuật nội soi giải ép điều trị bệnh lý hẹp ống sống thắt lưng.	Vũ Văn Cường /<br>Khoa Phẫu thuật Cột sống	T02/2020 – T02/2021	**05 triệu đồng**
20	CT20.2020	Nghiên cứu ứng dụng Robot định vị chính xác hỗ trợ trong phẫu thuật chỉnh vẹo cột sống vô căn thanh thiếu niên.	Nguyễn Hoàng Long/<br>Khoa PT Cột sống	T4/2020 – T10/2020	**05 triệu đồng**
21	CT21.2020	Nghiên cứu ứng dụng phẫu thuật nội soi lỗ liên hợp điều trị thoát vị đĩa đệm cột sống thắt lưng di trú.	Đỗ Mạnh Hùng/<br>Khoa PT Cột sống	T4/2020 – T4/2021	**05 triệu đồng**

22	CT22.2020	Kết quả tạo hình tai do dị tật tai nhỏ bẩm sinh bằng chất liệu nhân tạo Polyethylene.	Nguyễn Hồng Hà/ Khoa PT Hàm mặt – Tạo hình – Thẩm mỹ	T4/2020 – T8/2020	**10 triệu đồng**
23	CT23.2020	Nghiên cứu ứng dụng nẹp vít tự tiêu trong điều trị gãy xương hàm mặt.	Vũ Trung Trực/ Khoa PT Hàm mặt – Tạo hình – Thẩm mỹ	T3/2020 – T4/2021	**05 triệu đồng**
24	CT24.2020	Đánh giá kết quả ứng dụng nội soi hỗ trợ trong phẫu thuật chấn thương hàm mặt.	Đào Văn Giang/ Khoa PT Hàm mặt – Tạo hình – Thẩm mỹ	T3/2020 – T10/2020	**05 triệu đồng**
25	CT25.2020	Đánh giá kết quả chuyển thần kinh XI cho thần kinh trên vai trong điều trị liệt đám rối thần kinh cánh tay người lớn.	Đào Văn Giang/ Khoa PT Hàm mặt – Tạo hình – Thẩm mỹ	T3/2020 – T9/2020	**05 triệu đồng**
26	CT26.2020	Đánh giá kết quả tạo hình vá vỡ xương sàn hốc mắt sớm thì đầu tại Bệnh viện Việt Đức.	Bùi Mai Anh, Vũ Trung Trực/ Khoa PT Hàm mặt – Tạo hình – Thẩm mỹ	T01/2020 – T02/2021	**05 triệu đồng**
27	CT27.2020	Đánh giá kết quả chuyển thần kinh quay cho thần kinh nách trong điều trị liệt đám rối thần kinh cánh tay không hoàn toàn ở người lớn tại khoa PT Hàm mặt – tạo hình – thẩm mỹ - Bệnh viện HN Việt Đức.	Trần Thị Thanh Huyền/ Khoa PT Hàm mặt – Tạo hình – Thẩm mỹ	T3/2020 – T10/2020	**05 triệu đồng**
28	CT28.2020	Kết quả phục hồi chức năng sau thay khớp vai do gãy phức tạp đầu trên xương cánh tay tại Bệnh viện Việt Đức.	Bùi Duy Hiếu/ Khoa Phục hồi chức năng	T01/2020 – T10/2020	**05 triệu đồng**
29	CT29.2020	Chuẩn bị nền vết thương – vai trò quan trọng trong chăm sóc vết thương nhiễm khuẩn	Nguyễn Đức Chính/ Khoa PT Nhiễm khuẩn	T02/2020 – T5/2020	**05 triệu đồng**
30	CT30.2020	Kết quả phẫu thuật nội soi tái tạo dây chằng chéo trước bằng mảnh ghép nửa trước gân cơ mác dài tự thân tại bệnh viện Việt Đức	Lê Mạnh Sơn, Nguyễn Văn Phan/ Khoa PT chấn thương chung	T12/2019 – T12/2020	**0 đồng**
31	CT31.2020	Đánh giá kết quả điều trị sau mổ 5 năm với gãy mâm chày Schatzker V, VI bằng nẹp vít khóa tại bệnh viện Việt Đức	Lê Mạnh Sơn, Đặng Trung Kiên/ Khoa PT CT chung	T5/2020 – T5/2021	**0 đồng**
32	CT32.2020	Kết quả bước đầu chụp cắt lớp trong mổ sử dụng BodyTom hỗ trợ phẫu thuật cột sống ngực thắt lưng cùng	Nguyễn Lê Bảo Tiến, Đinh Ngọc Sơn/ Khoa PT Cột Sống	T02/2020 – T02/2021	**0 đồng**
33	CT33.2020	Kết quả bước đầu phẫu thuật nội soi hỗ trợ cố định cột sống, giải ép, ghép xương liên thân đốt qua lỗ liên hợp điều trị bệnh lý cột sống vùng thắt lưng cùng	Nguyễn Lê Bảo Tiến, Võ Văn Thanh/ Khoa PT Cột sống	T2/2020 – T2/2021	**0 đồng**

CHUYÊN NGÀNH TIÊU HÓA – TIẾT NIỆU

STT	Mã số đề tài	Tên đề tài	Chủ nhiệm đề tài/ Đơn vị thực hiện	Thời gian thực hiện	Kinh phí từ nguồn bệnh viện
1	TH01.2020	Nghiên cứu kết quả phẫu thuật cắt toàn bộ tuyến tiền liệt điều trị ung thư tuyến tiền liệt ở giai đoạn khu trú.	Trần Chí Thanh/<br>Khoa Điều trị Theo yêu cầu	T01/2020 – T12/2020	**05 triệu đồng**
2	TH02.2020	Nghiên cứu đặc điểm tái phát, di căn của ung thư đại trực tràng.	Hoàng Minh Đức/<br>Khoa Điều trị Theo yêu cầu	T3/2020 – T3/2021	**05 triệu đồng**
3	TH03.2020	Khảo sát tình hình mắc rối loạn phát triển giới tính ở người bệnh đến khám và điều trị tại Bệnh viện Hữu nghị Việt Đức	Trần Thị Ngọc Anh,<br>Nguyễn Việt Hoa/<br>Khoa PT Nhi và trẻ sơ sinh	T02/2020 – T11/2020	**10 triệu đồng**
4	TH04.2020	Kết quả nuôi dưỡng người bệnh trước mổ.	Phạm Hoàng Hà/<br>Khoa Phẫu thuật Tiêu hóa	T3/2020 – T3/2021	**05 triệu đồng**
5	TH05.2020	Kết quả chăm sóc vết mổ nhiễm khuẩn tại khoa Phẫu thuật Tiêu hóa – Bệnh viện Hữu nghị Việt Đức	Phạm Hoàng Hà/<br>Khoa Phẫu thuật Tiêu hóa	T3/2020 – T3/2021	**05 triệu đồng**
6	TH06.2020	Đánh giá kết quả sớm điều trị viêm phúc mạc ruột thừa bằng phẫu thuật nội soi tại bệnh viện Việt Đức	Dương Trọng Hiền/<br>Khoa PT Cấp cứu Tiêu hóa	T01/2020 – T12/2020	**05 triệu đồng**
7	TH07.2020	Đánh giá kết quả sớm điều trị thoát vị bẹn tái phát bằng phương pháp đặt lưới qua nội soi ổ bụng tại bệnh viện Hữu nghị Việt Đức	Lê Việt Khánh/<br>Khoa PT Cấp cứu Tiêu hóa	T01/2020 – T12/2020	**05 triệu đồng**
8	TH08.2020	Tư vấn chăm sóc người bệnh nhiễm HIV/AIDS cho người nhà người bệnh tại Bệnh viện Hữu nghị Việt Đức	Nguyễn Đức Chinh,<br>Nguyễn Minh Ky/<br>Khoa PT Nhiễm khuẩn	T02/2020 – T7/2020	**05 triệu đồng**
9	TH09.2020	Bóc u phì đại lành tính tuyến tiền liệt nội soi qua đường niệu đạo bằng laser công suất lớn.	Đỗ Trường Thành/<br>Phẫu thuật Tiết niệu	T4/2020 – T02/2021	**05 triệu đồng**
10	TH10.2020	Đánh giá kết quả sớm phẫu thuật nội soi kết hợp nội soi tán sỏi điện thủy lực điều trị sỏi đường mật ở các bệnh nhân dưới 60 tuổi tại bệnh viện Hữu nghị Việt Đức	Đỗ Tuấn Anh,<br>Mẫn Văn Chung/<br>Khoa PT Gan mật	T5/2020 – T9/2020	**05 triệu đồng**

11	TH11.2020	Nghiên cứu áp dụng cắt gan nội soi có kiểm soát cuống gan toàn bộ tạm thời trong điều trị u gan lành tính tại BV Hữu nghị Việt Đức	Trần Đình Thơ,<br>Mẫn Văn Chung/<br>Khoa PT Gan mật	T3/2020 – T7/2020	**05 triệu đồng**
12	TH12.2020	Nghiên cứu so sánh đánh giá mức độ xơ gan giữa mô bệnh học với máy siêu âm đo độ đàn hồi nhu mô gan: nghiên cứu đầu tiên tại Việt Nam	Phạm Gia Anh,<br>Nguyễn Lan Hương/<br>Khoa Ung bướu	T4/2020 – T4/2021	**05 triệu đồng**
13	TH13.2020	Thực hành và kết quả Lọc màng bụng tại khoa Thận – Lọc máu, bệnh viện Hữu nghị Việt Đức	Nguyễn Thị Thủy /<br>Khoa Thận Lọc máu	T4/2020 – T4/2021	**05 triệu đồng**
14	TH14.2020	Đánh giá kết quả ghép thận từ người cho chết não sau 10 năm 2010 – 2020 tại Bệnh viện Việt Đức.	Lê Nguyên Vũ,<br>Nguyễn Quang Nghĩa/<br>Trung tâm Ghép tạng	T12/2019 – T12/2020	**05 triệu đồng**
15	TH15.2020	Cải tiến cách thức lấy thận nội soi để ghép và ứng dụng sử dụng đoạn tĩnh mạch sinh dục kéo dài tĩnh mạch thận trong ghép thận từ người cho sống tại Bệnh viện Việt Đức giai đoạn 2019 – 2020.	Lê Nguyên Vũ,<br>Ninh Việt Khải/<br>Trung Tâm ghép tạng	T8/2019 – T8/2020	**05 triệu đồng**
16	TH16.2020	Kết quả phẫu thuật bệnh rò hậu môn tái phát tại Bệnh viện Hữu nghị Việt Đức.	Phạm Phúc Khánh,<br>Nguyễn Ngọc Ánh/<br>TT PT Đại trực tràng – TSM	T01/2020 – T10/2020	**05 triệu đồng**
17	TH17.2020	Mô tả đặc điểm lâm sàng, cận lâm sàng và đánh giá kết quả điều trị phẫu thuật bệnh Hirchsprung ở người trưởng thành	Lê Nhật Huy,<br>Nguyễn Xuân Hùng/<br>TT PT Đại trực tràng & TSM	T01/2020 – T01/2021	**05 triệu đồng**
18	TH18.2020	Đánh giá rối loạn tình dục ở bệnh nhân cắt dương vật do ung thư tại Trung tâm Nam học, Bệnh viện Hữu nghị Việt Đức	Nguyễn Quang/<br>Trung tâm Nam học	T3/2020 – T3/2021	**05 triệu đồng**
19	TH19.2020	Đánh giá vai trò của siêu âm Doppler dương vật trong đánh giá tình trạng cương dương vật	Nguyễn Quang/<br>Trung tâm Nam học	T3/2020 – 12/2020	**05 triệu đồng**

CHUYÊN NGÀNH ĐIỀU DƯỠNG					
**STT**	**Mã số đề tài**	**Tên đề tài**	**Chủ nhiệm đề tài/ Đơn vị thực hiện**	**Thời gian thực hiện**	**Kinh phí từ nguồn bệnh viện**
1	ĐD01.2020	Đánh giá hiệu quả chương trình dự phòng và chăm sóc bí tiểu sau phẫu thuật tại Bệnh viện Hữu nghị Việt Đức năm 2020.	Trần Văn Oánh,<br>Nguyễn Thị Chinh/<br>Phòng Điều Dưỡng	T9/2020 – T03/2021	**05 triệu đồng**
2	ĐD02.2020	Mô tả tỷ lệ bí tiểu trên bệnh nhân sau phẫu thuật và tìm hiểu một số yếu tố ảnh hưởng tới tình trạng bí tiểu sau phẫu thuật tại Bệnh viện HN Việt Đức năm 2020.	Bùi Thị Kim Nhung,<br>Nguyễn Thị Chinh/<br>Phòng Điều dưỡng	T03/2020 – T9/2020	**05 triệu đồng**
3	ĐD03.2020	Thời gian phục hồi nhu động ruột sau phẫu thuật đại trực tràng và yếu tố liên quan tại Bệnh viện HN Việt Đức	Chu Văn Long/<br>Phòng Điều dưỡng	T3/2020 – T3/2021	**05 triệu đồng**
4	ĐD04.2020	Đánh giá chất lượng giấc ngủ và các yếu tố ảnh hưởng đến giấc ngủ của người bệnh tại Bệnh viện HN Việt Đức.	Lê Sỹ Thoại/<br>Phòng Điều dưỡng	T02/2020 – T02/2021	**05 triệu đồng**
5	ĐD05.2020	Đánh giá kiến thức và kỹ năng thực hành của điều dưỡng trong sử dụng thang điểm hôn mê Glasgow Coma Scale tại Trung tâm Phẫu thuật Thần kinh, Bệnh viện Hữu nghị Việt Đức	Hoàng Bích Ngọc/<br>Khoa PT Thần kinh 2	T01/2020 – T10/2020	**05 triệu đồng**
6	ĐD06.2020	Nghiên cứu thực trạng công tác điều trị giảm đau qua sử dụng thang CPOT trên người bệnh chấn thương sọ não tại phòng Hồi sức, khoa Nội – Hồi sức Thần kinh, Bệnh viện Việt Đức năm 2020	Dương Minh Đức,<br>Chu Văn Long/<br>Khoa Nội – Hồi sức Thần kinh	T2/2020 – T12/2020	**05 triệu đồng**
7	ĐD07.2020	Đánh giá thực trạng công tác chuẩn bị người bệnh trước ghép tim từ người cho đa tạng chết não của điều dưỡng tại khoa Hồi sức tích cực Tim mạch – lồng ngực, bệnh viện Việt Đức năm 2020.	Nguyễn Thị Thu Hà,<br>Nguyễn Xuân Vinh/<br>Khoa Hồi sức tích cực Tim mạch – lồng ngực	T5/2020 – T5/2021	**05 triệu đồng**
8	ĐD08.2020	Đánh giá hiệu quả áp dụng hướng dẫn xử trí tình trạng bí tiểu trên bệnh nhân sau mổ chi dưới tại khoa Điều trị theo yêu cầu – BV Việt Đức.	Nguyễn Bá Anh/<br>Khoa Điều trị theo YC	T01/2020 – T12/2020	**05 triệu đồng**
9	ĐD09.2020	Đánh giá công tác chăm sóc thông tiểu sau mổ cắt bàng quang toàn bộ tại khoa điều trị 1C – Bệnh viện Việt Đức.	Trần Thị Lụa,<br>Nguyễn Bá Anh/<br>Khoa Điều trị theo YC	T01/2020 – T12/2020	**05 triệu đồng**
10	ĐD10.2020	Đánh giá chất lượng cuộc sống ở những bệnh nhân sau phẫu thuật thay khớp gối bằng bộ câu hỏi SF36 và thang đánh giá đau Womac tại bệnh viện Hữu nghị Việt Đức	Đinh Quang Chung/<br>Khoa PT Chi trên và YHTT	T3/2020 – T11/2020	**05 triệu đồng**

11	ĐD11.2020	Đánh giá kết quả theo dõi điều trị bảo tồn bệnh nhân chấn thương gan tại khoa Phẫu thuật cấp cứu bụng, Bệnh viện Hữu nghị Việt Đức năm 2020	Trần Thu Ngân,<br>Hoàng Thị Lan Anh/<br>Khoa PT cấp cứu bụng	T4/2020 – T4/2021	**10 triệu đồng**
12	ĐD12.2020	Mô tả thực trạng chăm sóc vết thương cho người bệnh phẫu thuật bụng cấp cứu tại khoa Phẫu thuật Cấp cứu bụng, Bệnh viện HN Việt Đức	Ngô Thị Mận,<br>Nguyễn Tiến Hào/<br>Khoa PT Cấp cứu bụng	T9/2019 – T8/2020	**05 triệu đồng**
13	ĐD13.2020	Kết quả chăm sóc người bệnh sau phẫu thuật tán sỏi thận qua da và một số yếu tố liên quan tại khoa Phẫu thuật Tiết niệu	Nguyễn Thị Thúy Huyền/<br>Khoa PT Tiết niệu	T4/2020- T12/2020	**05 triệu đồng**
14	ĐD14.2020	Kiến thức và thái độ về quản lý đau sau phẫu thuật của điều dưỡng tại Trung tâm Gây mê và hồi sức ngoại khoa, Bệnh viện HN Việt Đức	Trần Quang Phúc/<br>Khoa Gây mê 1	T5/2020 – T8/2020	**05 triệu đồng**
15	ĐD15.2020	Đánh giá hiệu quả của thực hiện quy định 5S quản lý tủ thuốc tại khoa Gây mê 2 năm 2020	Trần Văn Thủy,<br>Phạm Thị Ngoan/<br>Khoa Gây mê 2	T4/2020 – T9/2020	**05 triệu đồng**
16	ĐD16.2020	Đánh giá kết quả chăm sóc điều dưỡng và các tác dụng không mong muốn của phương pháp giảm đau ngoài màng cứng đối với người bệnh sau mổ vùng bụng tại Trung tâm Gây mê và Hồi sức ngoại khoa, Bệnh viện Hữu nghị Việt Đức	Vũ Thị Hằng,<br>Nguyễn Thị Thiện/<br>Khoa Gây mê 2	T5/2020 – T5/2021	**05 triệu đồng**
17	ĐD17.2020	Đánh giá kết quả thực hiện cải tiến trong phòng ngừa và chăm sóc loét tỳ đè của điều dưỡng khoa Hồi sức tích cực 1 năm 2020	Lại Thanh Nga,<br>Nguyễn Văn Thanh/<br>Khoa Hồi sức tích cực 1	T3/2020 – T3/2021	**05 triệu đồng**
18	ĐD18.2020	Đánh giá thực trạng chăm sóc bóng chèn ống NKQ, MKQ trên người bệnh thở máy tại khoa Hồi sức tích cực 1	Nguyễn Đức Dưỡng,<br>Nguyễn Đức Tiến/<br>Khoa Hồi sức tích cực 1	T3/2020 – T3/2021	**05 triệu đồng**
19	ĐD19.2020	Thực trạng chăm sóc ống thông dạ dày và một số yếu tố liên quan trên người bệnh nặng tại khoa Hồi sức tích cực 2 năm 2020	Khang Thị Diên,<br>Dương Thị Thanh/<br>Khoa Hồi sức tích cực 2	T4/2020 – T8/2020	**05 triệu đồng**
20	ĐD20.2020	Đánh giá hiệu quả kỹ thuật đặt huyết áp động mạch xâm lấn dưới hướng dẫn siêu âm ở bệnh nhân phù.	Nguyễn Trường Giang/<br>Khoa Hồi sức tích cực 2	T02/2020 – T10/2020	**05 triệu đồng**
21	ĐD21.2020	Đánh giá tình trạng nhiễm khuẩn tiết niệu ở người bệnh được hồi sức thở máy có đặt thông tiểu tại khoa Hồi sức tích cực Bệnh viện HN Việt Đức	Lê Thị Huệ/<br>Khoa Hồi sức tích cực 2	T4/2020 – T12/2020	**05 triệu đồng**
22	ĐD22.2020	Đánh giá hiệu quả dinh dưỡng sớm đường miệng trên bệnh nhân phẫu thuật ung thư đại tràng tại khoa Ung bướu Bệnh viện HN Việt Đức năm 2020.	Trần Thị Ngọc/<br>Khoa Ung bướu	T01/2020 – T12/2020	**10 triệu đồng**

23	ĐD23.2020	Ứng dụng điện thoại thông minh trong theo dõi vạt vi phẫu.	Nguyễn Ngân Giang/<br>Khoa PT Hàm mặt – Tạo hình – Thẩm mỹ	T4/2020-<br>T3/2021	**05 triệu đồng**
24	ĐD24.2020	Thực trạng kiểm soát đau sau mổ trĩ và sự hài lòng của người bệnh tại Trung tâm Phẫu thuật Đại trực tràng – tầng sinh môn	Vũ Thị Hồng,<br>Phạm Thị Thanh Huyền/<br>TT PT Đại trực tràng và TSM	T01/2020 –<br>T10/2020	**05 triệu đồng**
25	ĐD25.2020	Đánh giá chất lượng cuộc sống bệnh nhân sau điều trị phẫu thuật đau nửa mặt và co giật nửa mặt	Nguyễn Thị Ngân,<br>Dương Đại Hà/<br>Khoa PT Thần kinh 1	T4/2020 –<br>T4/2021	**10 triệu đồng**
26	ĐD26.2020	Khảo sát thực trạng chăm sóc và điều trị người bệnh chấn thương sọ não nặng sau khi ra viện	Hồ Thị Quỳnh Nga,<br>Đồng Văn Hệ/<br>Khoa PT Thần kinh 1	T5/2020 –<br>T5/2021	**10 triệu đồng**
27	ĐD27.2020	Gánh nặng chăm sóc của gia đình người bệnh bị chấn thương sọ não tại khoa PT Thần kinh 1, Bệnh viện HN Việt Đức năm 2020 và một số yếu tố liên quan	Phạm Thị Sơn/<br>Khoa PT Thần kinh 1	T4/2020 –<br>T4/2021	**05 triệu đồng**
28	ĐD28.2020	Đánh giá kiến thức của điều dưỡng và thực trạng chăm sóc dẫn lưu vùng cột sống thắt lưng tại khoa Phẫu thuật thần kinh 1, BV Hữu nghị Việt Đức năm 2020	Trần Thị Thúy Ngần/<br>Khoa PT Thần kinh 1	T02/2020 –<br>T02/2021	**05 triệu đồng**
29	ĐD29.2020	Khảo sát lý do người bệnh chọn khám tại khoa Phẫu thuật Thần kinh, bệnh viện Hữu nghị Việt Đức	Nguyễn Thị Xuân Mai,<br>Đồng Văn Hệ/<br>Khoa PT Thần kinh 1	T5/2020 –<br>T5/2021	**05 triệu đồng**
30	ĐD30.2020	Mức độ lo âu, căng thẳng của người bệnh trước mổ u não	Nguyễn Thị Thảo/<br>Khoa PT Thần kinh 1	T3/2020-<br>T9/2020	**05 triệu đồng**

GIÁM ĐỐC

BỆNH VIỆN HỮU NGHỊ VIỆT ĐỨC

Trần Bình Giang
